# Exploring utility of genomic epidemiology to trace origins of highly pathogenic influenza A/H7N9 in Guangdong

**DOI:** 10.1093/ve/veaa097

**Published:** 2020-12-18

**Authors:** Ru Bai, Reina S Sikkema, Bas B Oude Munnink, Cong Rong Li, Jie Wu, Lirong Zou, Yi Jing, Jing Lu, Run Yu Yuan, Ming Liao, Marion Koopmans, Chang-Wen Ke

**Affiliations:** Department of Viroscience, Erasmus University Medical Center, P.O. Box 2040, 3000CA Rotterdam, The Netherlands; Department of Viroscience, Erasmus University Medical Center, P.O. Box 2040, 3000CA Rotterdam, The Netherlands; Biosafety Laboratory, Zhongshan School of Medicine, Sun Yat-sen University, Guangzhou, 510080, China; Department of Pathogenic Microbiolgy, Guangdong Provincial Center for Disease Control and Prevention, Guangzhou, 511430, China; Department of Pathogenic Microbiolgy, Guangdong Provincial Center for Disease Control and Prevention, Guangzhou, 511430, China; School of Public Health, Southern Medical University, Guangzhou, 510515, China; Department of Pathogenic Microbiolgy, Guangdong Provincial Center for Disease Control and Prevention, Guangzhou, 511430, China; Department of Pathogenic Microbiolgy, Guangdong Provincial Center for Disease Control and Prevention, Guangzhou, 511430, China; Biosafety Laboratory, Zhongshan School of Medicine, Sun Yat-sen University, Guangzhou, 510080, China; National and Regional Joint Engineering Laboratory for Medicament of Zoonoses Prevention and Control, College of Veterinary Medicine, South China Agricultural University, Guangzhou, 510642, China; Department of Viroscience, Erasmus University Medical Center, P.O. Box 2040, 3000CA Rotterdam, The Netherlands; Department of Pathogenic Microbiolgy, Guangdong Provincial Center for Disease Control and Prevention, Guangzhou, 511430, China; Biosafety Laboratory, Zhongshan School of Medicine, Sun Yat-sen University, Guangzhou, 510080, China

**Keywords:** highly pathogenic influenza A/H7N9, genomic epidemiology, origins, Guangdong

## Abstract

The first highly pathogenic (HP) influenza A/H7N9 was reported in Guangdong in January 2017. To investigate the emergence and spread of HP A/H7N9 in Guangdong province, we sequenced 297 viruses (58 HP A/H7N9, 19 low pathogenic (LP) A/H7N9, and 220 A/H9N2) during 2016–2017. Our analysis showed that during the fifth wave, three A/H7N9 lineages were co-circulating in Guangdong: the local LP Pearl River Delta (PRD) lineage (13%), the newly imported LP Yangtze River Delta (YRD) lineage (23%), and the HP YRD lineage (64%). Previously circulating YRD-lineage LP during the third wave evolved to the YRD-lineage HP A/H7N9 in Guangdong. All YRD-lineage LP detected during the fifth wave most likely originated from newly imported viruses into Guangdong. Genotype comparison of HP A/H7N9 suggests limited outward spread of HP A/H7N9 to other provinces. The distribution of HP A/H7N9 cleavage site variants on live poultry markets differed from that found in humans, suggesting a V1-type cleavage site may facilitate human infections.

## 1. Introduction

Since the first detection of low pathogenic (LP) A/H7N9 avian influenza in the Yangtze River Delta (YRD) region in China in 2013, five epidemic waves of human and poultry transmission took place in China (Li et al. 2014). An important risk factor for human A/H7N9 infection is a visit to a live poultry market (LPM) (Liu et al. 2014; Yu et al. 2014). Control of the A/H7N9 epizootic has been challenging due to extensive live poultry trading and absence of obvious clinical symptoms in infected birds (Spackman et al. 2015; Zhou et al. 2015). In January 2017, the first highly pathogenic (HP) A/H7N9 virus was identified in a patient with confirmed exposure to dead poultry in Guangdong province, China (Ke et al. 2017). Subsequently, similar viruses were reported in poultry sampled at LPMs in Guangdong (Lu et al. 2018).

In response to the first emergence of HP A/H7N9 in Guangdong province, we used data from our ongoing LPMs environmental surveillance program in the Guangdong province to genetically trace the source of these HP A/H7N9 viruses. In addition, we used genomic data to compare the molecular characteristics of the A/H7N9 viruses in humans, poultry, and the environment. We aim to have a thorough understanding of the origin, spread, and transmission of this novel virus in Guangdong province.

## 2. Material and methods

### 2.1 Influenza a surveillance at the live poultry markets

Influenza A surveillance was conducted at twenty-eight sentinel hospitals and LPMs in twenty-one prefecture-level cities in Guangdong province during the fifth wave of the A/H7N9 outbreak (November 2016–August 2017). In order to trace the origin of HP A/H7 in Guangdong, we retrospectively screened samples of LPMs collected since January 2016. Environmental samples of LPMs include swab specimens collecting from cages, chopping boards, drinking water, and feather removal machines. Additionally, oropharyngeal and cloacal swabs were taken from apparently healthy chickens, ducks, pigeons, francolins, geese, and quails on LPMs. We collected samples at one retail market and one wholesale market in each of twenty-one prefecture-level cities. All samples were placed in viral transport medium and stored at −80 °C until further processing.

### 2.2 Screening and virus isolation

Samples were first tested for the presence of influenza A RNA using real-time reverse transcription-PCR (rRT-PCR) assays targeting the matrix protein, followed by rRT-PCR assays specifically targeting the A/H7 and A/H9 genes in twenty-one local Centers for Disease Control and Prevention (CDC) and were further verified by the Guangdong Provincial CDC as described previously (Ke et al. 2014). HP A/H7N9 was detected with rRT-PCR amplification of a fragment of HA, as described previously (Jia et al. 2017). Samples positive for A/H7 and A/H9 were treated by antibiotics for 30 min and blindly passaged for two to three generations in 9- to 10-day-old embryonated chicken eggs for virus isolation. Allantoic fluid was collected 2 days after inoculation and tested for the presence of virus by a hemagglutination test using 1 per cent guinea pig erythrocytes or 0.5 per cent turkey erythrocytes. Then positive subtypes of H7 and H9 were further confirmed by rRT-PCR and processed for sequencing. In total, 251 HP and 70 LP A/H7N9 rRT-PCR positive samples were inoculated, yielding 83 and 24 isolates, respectively. Of these, fifty-eight and nineteenwere successfully sequenced. For A/H9N2 viruses, 863 positive samples were inoculated, yielding 360 isolates, and 220 were successfully sequenced.

### 2.3 Genome sequencing

Viral RNA was extracted from allantoic fluid samples using the QIAamp Viral RNA Mini Kit (Qiagen, Hilden, Germany). Extracted RNA was subjected to reverse transcription and amplification using the SuperScript^®^ III One-Step RT-PCR system (Thermo Fisher Waltham, USA). Genome sequencing of environmental A/H7 and A/H9 viruses was performed on the next-generation Ion PGM sequencing platform. Sequencing data were analyzed with CLC Genomics Workbench 7.5.1 software. Low-quality reads were trimmed using CLC trimmer with a quality limit set at 0.05. Trimmed reads were then *de novo* assembled in CLC using default parameters. Contigs with a coverage above 10 were extracted and annotated using a BLAST search against the NCBI non-redundant nucleotide database (Benson et al. 2014). The sequence with the highest nucleotide identity was downloaded as reference for read mapping. Consensus sequences for all segments were extracted from the mapping results, with at least 10× coverage depth at each site.

### 2.4 Epidemiological data

Information on live poultry imports from other regions of China to Guangdong province was obtained from the local Ministry of Agriculture. According to the geographic distribution of twennty-nine Chinese provinces and cities, we classified them into six regions. Provinces Hebei, Heilongjiang, Jilin, Liaoning, Inner Mongolia, Shanxi, and Beijing city were grouped as northern regions. Provinces Hubei, Hunan, and Henan were grouped as central regions. Provinces Anhui, Fujian, Jiangsu, Jiangxi, Shandong, Zhejiang, and the city of Shanghai were grouped as eastern region. Hainan is considered the southern region. The north-western region included provinces Gansu, Ningxia, Qinghai, Shaanxi, Xinjiang and the south-western region included provinces of Guangxi, Guizhou, Sichuan, Tibet, Yunnan, and the city of Chongqing.

### 2.5 Phylogenetic analysis

Genome sequences from the present study were combined with all publicly available sequences of influenza A/H7N9 (China) and A/H9N2 (China) virus sequences available in GISAID and part of A/H9N2 (China) from NCBI. The reference sequence accession numbers are summarized in Supplementary Table S3. A total of 15,103 A/H7N9 sequences (HA = 1,989, NA = 2,173, PB2 = 1,779, PB1 = 1,620, PA = 1,793, NP = 1,913, M = 2,027, NS = 1,809) and 6950 A/H9N2 sequences (PB2 = 1,159, PB1 = 1,150, PA = 1,162, NP = 1,151, M = 1,172, NS = 1,140) were downloaded and analyzed. From the first to the fifth wave, a total of 1,371 complete genomes (1,301 of LP A/H7N9 and 70 of HP A/H7N9) were available in GISAID. We only used sequence data with known collection dates and sampling locations in our analyses. Sequences were aligned in MUSCLE v3.5 with default settings (Edgar 2004). The alignment was manually checked and corrected in BioEdit (version 7.0.5.2). The amino acid sequences of 58 HP A/H7N9 and 19 LP A/H7N9 viruses were translated by NCBI ORF Finder. Phylogenetic analysis was performed using IQ-TREE (Nguyen et al. 2015), using maximum-likelihood phylogenies under the GTR+F + I+G4 nucleotide substitution model as the best predicted model. Clade nomenclature of eight segments was assigned as described previously (Lam et al. 2015). We defined a novel genotype of A/H7N9 virus if one of eight segments belonged to a different clade. Clades were further divided into sub-clades (1.1, 1.2, 2.1, 2.2, etc.) if at least three sequences were available and the average intra-sub-clade genetic distance was less than 3 per cent, as described previously (Lam et al. 2015).

We estimated molecular clock phylogenies by using the Bayesian Markov chain Monte Carlo approach implemented in BEAST version 1.8. We computed four independent Markov chain Monte Carlo runs of 1.5 × 10^8^ steps for each alignment and extracted a subset of 2,000 phylogenies from the posterior tree distribution, subsequently used as an empirical tree distribution for phylogeographic analyses. We computed maximum clade credibility trees for each dataset by using TreeAnnotator. We used the discrete phylogeographic method implemented in BEAST to investigate spatial dynamics of H7N9 virus lineages from seven regions in China. The seven regions were central China (Jiangxi, Hunan, and Hubei), southern China (Guangdong), Eastern China (Anhui, Jiangsu, Shandong, and Zhejiang), Southwestern China (Yunnan, Sichuan, Gansu, Guangxi, and Guizhou), Northern China (Hebei, Henan, Liaoning, and Jilin), Southeastern China (Fujian), Northwestern China (Xinjiang, Shanxi, and Ningxia).

### 2.6 Analysis of genetic markers for risk assessment

Protein sequences of fifty-eight HP A/H7N9 viruses in this study were compared with publicly available HP A/H7N9 sequences using BioEdit. Biologically relevant amino acid substitutions on protein HA, NA, NS1, M2, PA, PB1, and PB2 were compared as described in previous studies (Jiao et al. 2008; Ping et al. 2010; Mok et al. 2011, 2014; Tada et al. 2011; Song et al. 2014; Bi et al. 2015; Xiao et al. 2016).

## 3. Results

### 3.1 Influenza a surveillance at the live poultry markets

**Figure 1.**
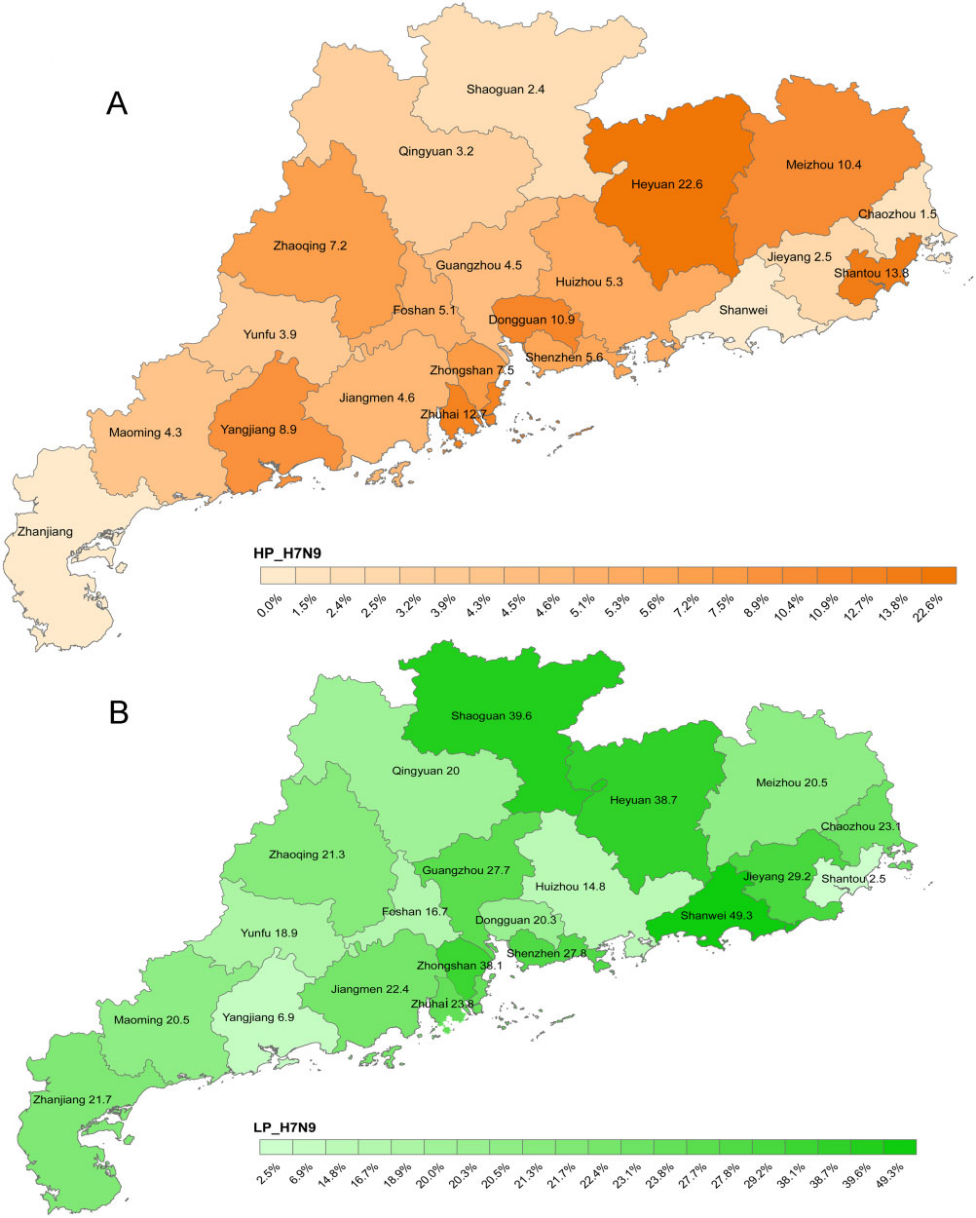
Geographic spread and distribution of HP (A) and LP (B) A/H7N9 viruses on LPMs in twenty-one cities of Guangdong province from November 2016 to August 2017. Percentage represented the positive rate of HP and LP A/H7N9 in total avian influenza viruses by RT-PCR detection from November 2016 to August 2017.

During the study period, 34,106 environmental swabs were tested, ranging from 572 to 3,790 per month during January 2016–August 2017 (Table 1). Surveillance data showed that influenza A virus was detected in 7.4–29.4 per cent of the monthly collected samples. A/H7 accounted for 1.0–36.8 per cent of all avian influenza A viruses (Table 1). HP A/H7 was first detected in environmental samples collected on 7 November 2016 in the city of Heyuan. Between November 2016 and August 2017, HP A/H7N9 viruses spread to LPMs in nineteen cities (Fig. 1A and B). From November 2016 to August 2017, the proportion of HP-positive samples accounted for 0.5–12 per cent of all avian influenza A viruses. Approximately 25 per cent of all A/H7N9-positive swabs were positive for HP A/H7 (Table 1). We sequenced the complete genomes of 58 HP H7N9, 19 LP H7N9, and 220 H9N2 viruses isolated from LPMs and poultry from November 2016 to November 2017. These sequences have been deposited into the GISAID database with accession numbers listed in Supplementary Table S1.

**Table 1.** Environmental surveillance of avian influenza viruses and H7N9 influenza viruses in Guangdong province from January 2016 to August 2017.

		No. (%) of positive samples			
Time	No. of collected samples	FluA^+^/total	H7N9/FluA^+^	HP H7N9/FluA^+^	LP H7N9/FluA^+^
Jan 2016	2,523	743 (29.45)	50 (6.73)	0 (0)	50 (6.73)
Feb 2016	2,186	537 (24.57)	52 (9.68)	0 (0)	52 (9.68)
Mar 2016	2,203	611 (27.73)	84 (13.75)	0 (0)	84 (13.75)
Apr 2016	1,755	342 (19.49)	12 (3.51)	0 (0)	12 (3.51)
May 2016	1,867	430 (23.03)	17 (3.95)	0 (0)	17 (3.95)
June 2016	625	85 (13.6)	3 (3.53)	0 (0)	3 (3.53)
July 2016	596	105 (17.62)	2 (1.9)	0 (0)	2 (1.9)
Aug 2016	750	132 (17.6)	0 (0)	0 (0)	0 (0)
Sept 2016	572	97 (16.96)	1 (1.03)	0 (0)	1 (1.03)
Oct 2016	712	99 (13.9)	4 (4.04)	0 (0)	4 (4.04)
Nov 2016	1,852	421 (22.73)	27 (6.41)	2 (0.48)	25 (5.94)
Dec 2016	2,590	686 (26.49)	206 (30.03)	38 (5.54)	168 (24.49)
Jan 2017	3,790	708 (18.68)	260 (36.72)	55 (7.77)	205 (28.95)
Feb 2017	3,341	672 (20.11)	247 (36.76)	48 (7.14)	199 (29.61)
Mar 2017	3,062	507 (16.56)	131 (25.84)	62 (12.23)	69 (13.61)
Apr 2017	1,910	355 (18.59)	101 (28.45)	32 (9.01)	69 (19.44)
May 2017	1,960	286 (14.59)	38 (13.29)	10 (3.5)	28 (9.79)
June 2017	599	55 (9.18)	12 (21.82)	3 (5.45)	9 (16.36)
July 2017	632	47 (7.44)	6 (12.77)	1 (2.13)	5 (10.64)
Aug 2017	581	90 (15.49)	0 (0)	0 (0)	0 (0)
Total	34,106	7,008 (20.55)	1,253 (17.88)	251 (3.58)	1,002 (14.3)

FluA+, type A avian influenza viruses; LP, low pathogenic; HP, highly pathogenic.

### 3.2 Poultry trade

**Figure 2.**
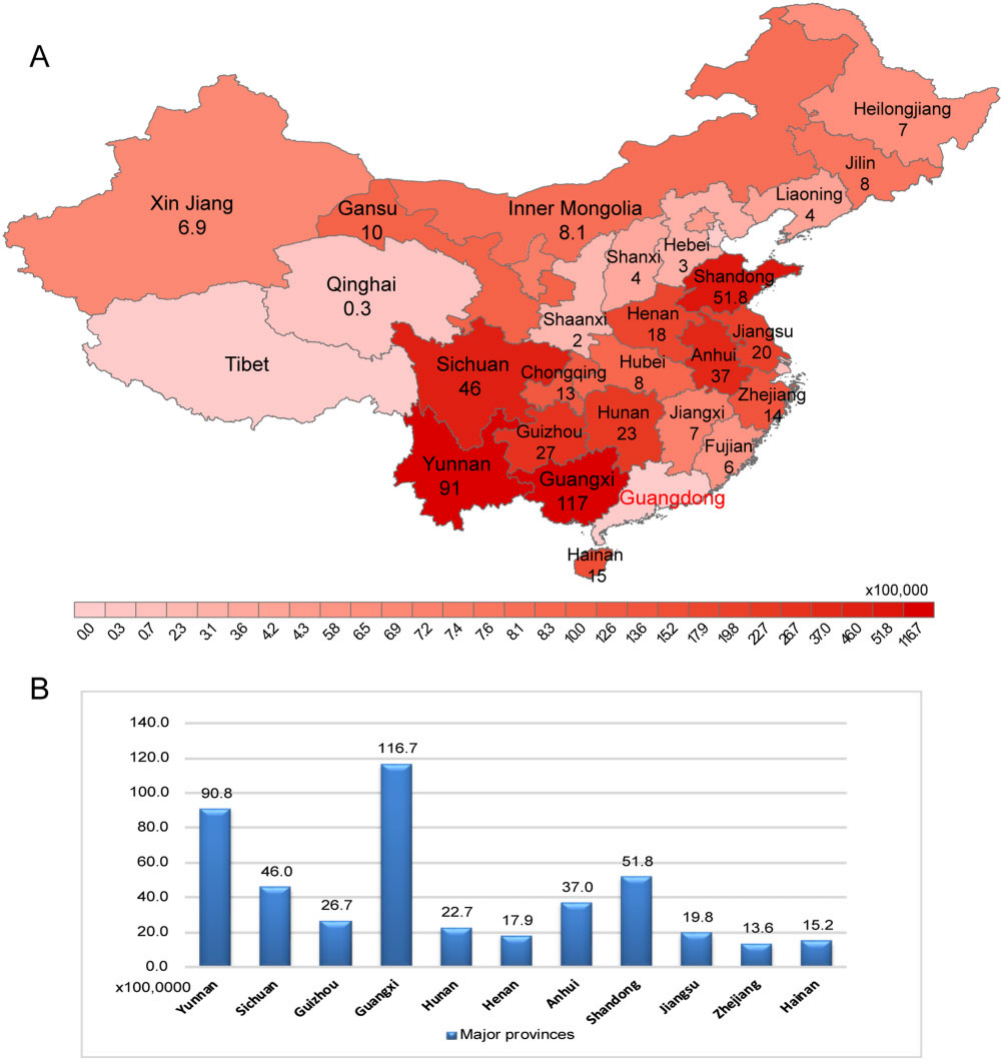
Live poultry import from different areas of China into Guangdong province. (A) Shows numbers (in millions) of live poultry were imported into Guangdong from different areas of China including twenty-nine provinces from the northwestern, southwestern, northern, central, eastern, and southern regions during the period from January 2016 to July 2018. (B) Shows 11 major provinces that accounted for 70 per cent of imported live poultry into Guangdong province from January 2016 to July 2018.

A total of 5.5 billion live birds were imported into Guangdong from different areas of China including twenty-nine provinces from the northwestern, southwestern, northern, central, eastern, and southern regions during the period January 2016–July 2018 (Supplementary Table S2). Specifically, live poultry from the south-west, central, eastern, and south of China accounted for 70 per cent of imported live poultry into Guangdong province. Those regions included eleven provinces, which were Yunnan (south-west), Sichuan (south-west), Guizhou (south-west), Guangxi (south-west), Hunan (central), Henan (central), Anhui (eastern), Shandong (eastern), Jiangsu (eastern), Zhejiang (eastern), and Hainan (south) (Fig. 2A and B).

### 3.3 Analysis of a/H7N9 sequences

We compared the newly generated sequence data with all Chinese A/H7N9 HP and LP full genomes available in public domains. A total of 57 out of 523 LP A/H7N9 sequences (including 19 genomes from this study) were from Guangdong (11%), 260 (50%), 85 (16%), and 121 (23%) were from eastern, central, and other regions of China, respectively. Of the 125 HP sequences (including 58 genomes from this study), 89 (71%) were collected in Guangdong, 2 (2%) from eastern China, 5 (4%) from central China, and 29 (23%) from other regions.

**Figure 3.**
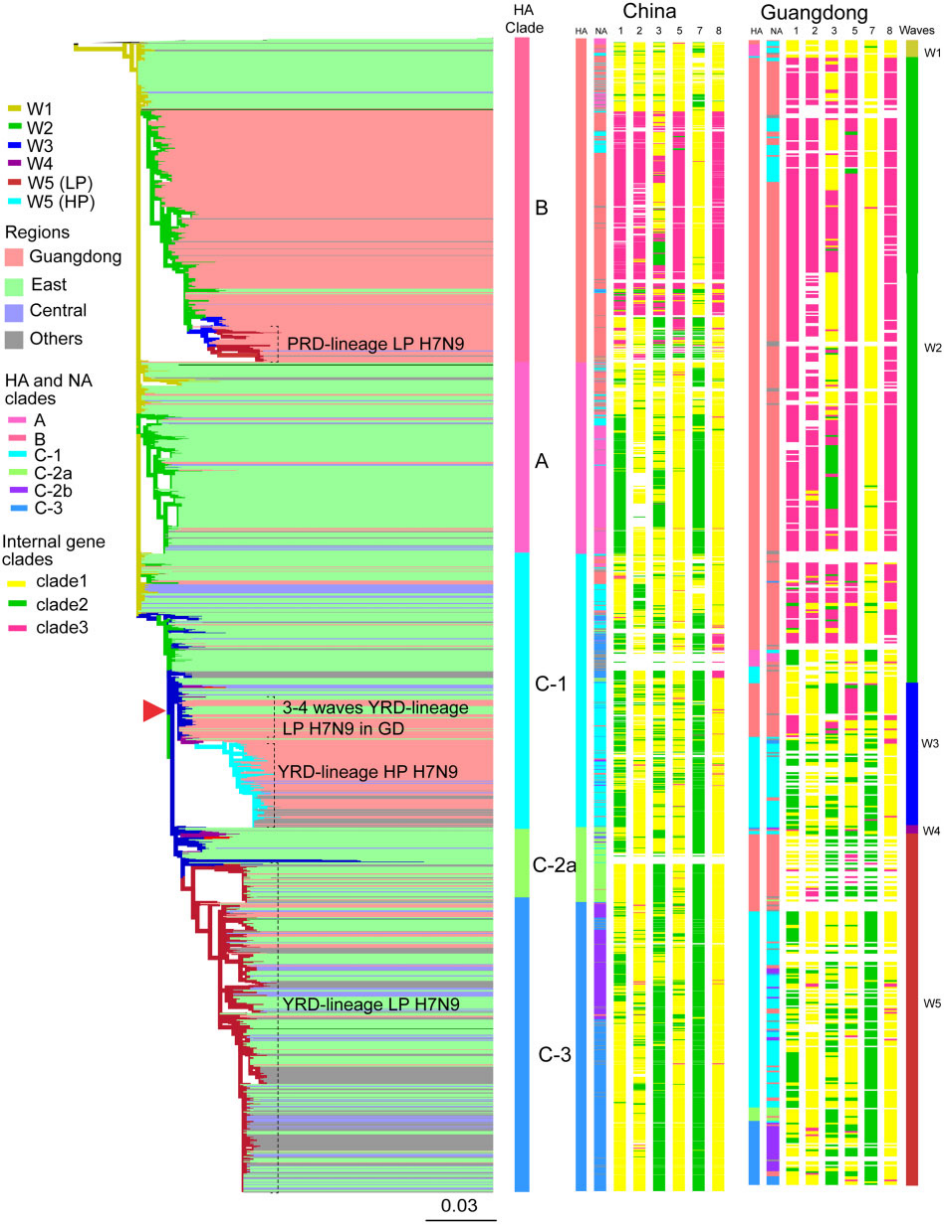
Phylogenetic analysis of the H7N9 influenza viruses. H7N9 viruses are divided into three clades (A, B, and C). Different colors of branches are indicated by different waves of H7N9 viruses. Viruses from different regions are highlighted by different colors. The surface and internal genes of different clade H7N9 influenza viruses are distinguished by different colored bars. All branch lengths are scaled according to the number of substitutions per site. Abbreviations of eight segments are showed on the top of gene constellation bars: HA, haemagglutinin gene; NA, neuraminidase gene; 1, PB2 gene; 2, PB1 gene; 3, PA gene; 5, NP gene; 7, M gene; 8, NS gene. The fifth wave PRD-lineage LP A/H7N9, YRD-lineage HPAI H7N9, and YRD-lineage LP A/H7N9 viruses are highlighted by the black bracketed lines. The previous YRD-lineage LP A/H7N9 in Guangdong province is highlighted by a red arrow. The surface and internal genes of H7N9 viruses from the first wave to the fifth wave inside Guangdong province are listed.

**Figure 4.**
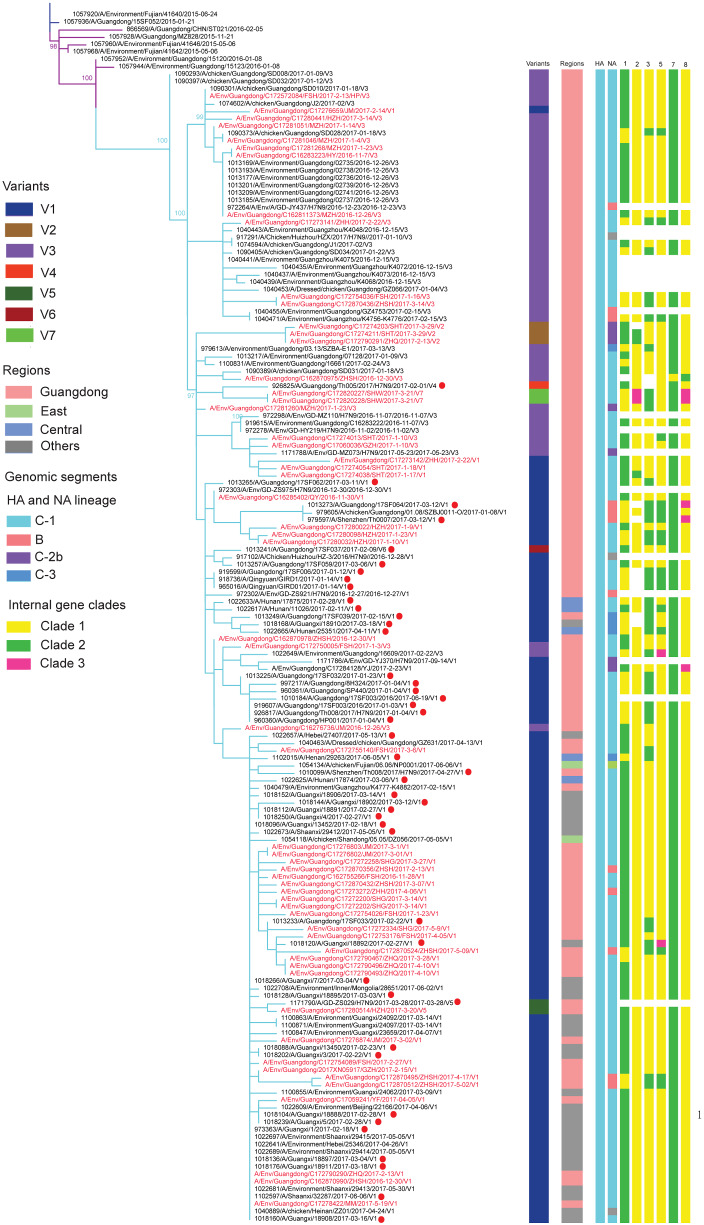
Enlarged branch of phylogenetic analysis of HP A/H7N9 viruses during the fifth wave in Fig. 3. Different variants, regions, and clades of HP A/H7N9 viruses were highlighted by different colors. Human HP A/H7N9 were labeled out by red dot in the tree. Abbreviations of eight segments are showed on the top of gene constellation bars: HA, haemagglutinin gene; NA, neuraminidase gene; 1, PB2 gene; 2, PB1 gene; 3, PA gene; 5, NP gene; 7, M gene; 8, NS gene. All branch lengths are scaled according to the number of substitutions per site.

Based on the evolutionary analysis of H7N9 viruses since first outbreak in 2013, the YRD region and the PRD region were identified as the H7N9 outbreak source in China. A/H7N9 viruses are separated in the YRD-lineage and the Pearl River Delta (PRD)-lineage LP A/H7N9 viruses (Lam et al. 2015). The YRD viruses have expanded and have been further subdivided in clades and sub-clades, following nomenclature as described by Lam et al. (2015) (Fig. 3, Supplementary Fig. S1). The A/H7N9 viruses of the fifth wave clustered into two major clades, designated as Clade B and C. All LP YRD-lineage A/H7N9 from the fifth wave cluster into the C-2a, C-2b, and C-3 clades. HP YRD-lineage A/H7N9 viruses clustered into Clade C-1. The ancestor of the HA gene of YRD-lineage HP A/H7N9 seems to have originated from third wave viruses that spread to Guangdong in early 2015 (Fig. 4, Supplementary Figs S1 and S3). We found that virus isolates from eastern China and Guangdong in the fourth wave were at the root of clade C-1, suggesting that this clade possibly originated from eastern China (Fig. 4, Supplementary Fig. S1).

The comparison of genotypes identified a total of 180 genotypes (G1–G180) from the first wave to the fifth epidemic A/H7N9 wave (Supplementary Fig. S2). A total of ffifty-nine genotypes were detected in the fifth wave in China, of which nineteen genotypes were HP (Supplementary Fig. S2), and forty genotypes were LP, reflecting the widespread evolution of these viruses. Of the nineteen genotypes of HP, 15, 5, 2, and 2 genotypes were found in Guangdong, Eastern China, Central China, and other regions, respectively. The most prevalent genotype G108 (83/125; 66%) was detected across China (Supplementary Fig. S2). Twelve of the fourteen other genotypes found in our Guangdong dataset are unique to Guangdong (Supplementary Fig. S2). Genotypes 113, 114, 119, and 126 were uniquely detected in other regions of China. Most HP A/H7N9 genotypes in Guangdong reassorted with NA and six internal genes of newly introduced YRD-lineage LP, local PRD-lineage LP, and local A/H9N2 viruses (Fig. 4). Our genome analysis confirmed the diversity of A/H7N9 viruses and its continuing diversification by genetic reassortment.

### 3.4 Reassortment history

To clarify the mechanism behind the genotype diversity of LP and HP A/H7N9 in Guangdong, the internal genes of the viruses within each HA clade were further analyzed. During the fifth wave, three H7N9 lineages were co-circulating in Guangdong: the local LP PRD lineage (13%), the newly imported LP YRD lineage (23%), and HP YRD lineage (64%). Strikingly, in addition to the PRD-lineage LP viruses, a diverse range of YRD-lineage LP A/H7N9 viruses were found during the fifth wave (Fig. 3), which strongly suggests that these were newly introduced from other provinces. Moreover, in the fifth wave, the six internal genes of PRD-lineage A/H7N9 viruses in Guangdong seem to be replaced by those of YRD-lineage A/H7N9 viruses (Fig. 3). Most viruses, including PRD-lineage A/H7N9 viruses, contained descendants of internal genes of YRD-lineage A/H7N9, while none of the local LPM and human A/H7N9 viruses inherited all six internal gene segments from PRD-lineage A/H7N9. In the beginning of the fifth wave, HP A/H7N9 viruses acquired PB2 and M gene segments from clade 2, and had PB1, PA, NP, and NS gene segments of clade 1 (Fig. 3, Supplementary Fig. S1). The later spread of this virus to different regions in Guangdong led to occasionally replacement of six internal genes by clade 1 PB2 or by clade 2 PB1, PA, NP, and NS genes, or by clade 3 PB1, NP, and NS genes. Thus, current HP A/H7N9 was a result of extensive reassortment of introduced YRD-lineage A/H7N9, local PRD-lineage A/H7N9, and A/H9N2 viruses (Fig. 3, Supplementary Fig. S1).

### 3.5 Analysis of HP A/H7N9 genomes for biologically relevant mutations

**Figure 5.**
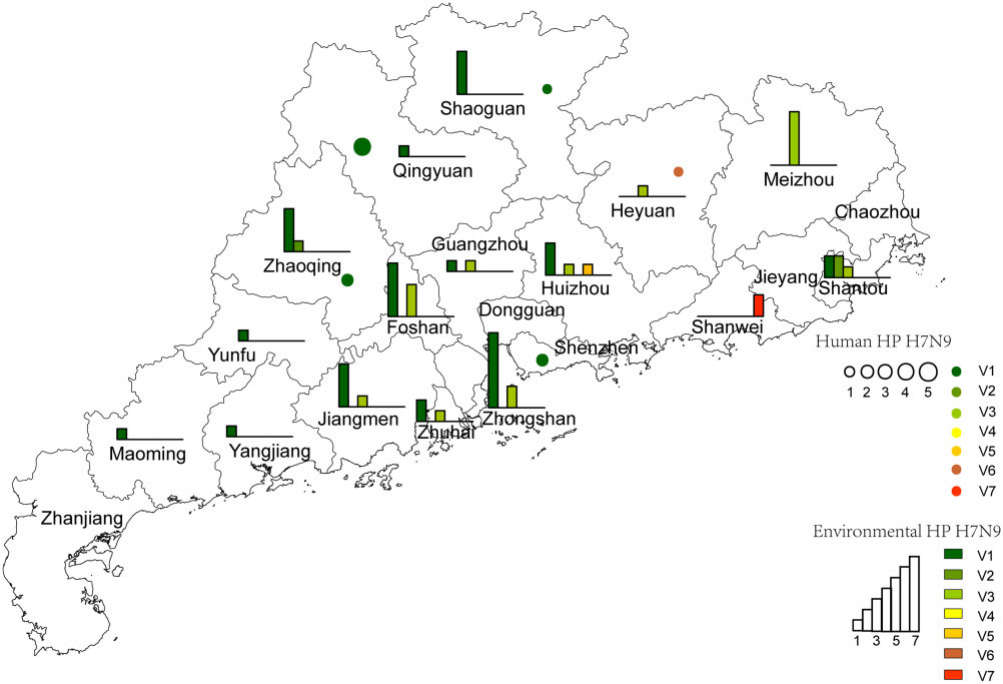
Distribution of the seven different motifs on HA cleavage sites of HP A/H7N9 viruses in Guangdong province for environmental and human isolates. Different motifs were highlighted with different colors. The number of variants detected in human and environment were represented by the scales of circle and column, respectively.

Six HA cleavage site amino acid motifs have been described, including PKR**KRTA**R↓G (V1), PKG**KRTK**R↓G (V2), PKG**KRTA**R↓G (V3), PKG**KRIA**R↓G (V4), PKR**RRTA**R↓G (V5), and PKR**KRAA**R↓G (V6). In the present study, we identified a novel motif ‘PKR**KRIA**R↓G′ and defined it as V7. Moreover, variants 1, 2, 3, and 5 were also found in the present study. Analysis based on all publicly available HP A/H7N9 and those in the present study showed that most human HP A/H7N9 viruses contain the V1 motif. Variant 1 HP A/H7N9 was found in 93.7 per cent (45/48) of human cases, variants 3, 5, and 6 accounted for 2 per cent (1/48) each. In LPMs’ surveillance samples, variants 1 and 3 accounted for 49.6 per cent (57/115) and 43.4 per cent (50/115) of positive samples, and variants 2, 5, and 7 accounted for 3.5 per cent (4/115), 1.7 per cent (2/115), and 1.7 per cent (2/115), respectively. V1 was widely distributed in the north, east, west and center of Guangdong, while V2–V7 only circulated in the center and east of Guangdong (Fig. 5). The human cases were mainly detected in central, northern, and eastern Guangdong (Fig. 5).

Amino acid substitutions of human and environmental HP A/H7N9 were analyzed and compared (H3 numbering), with an emphasis on variants V1 and V3. Many amino acid substitutions that may be relevant for risk assessment were found both in human and environmental HP A/H7N9, which include G186V on the HA gene, K526R, A588V, and K702R on the PB2 gene, V100A, A402S, and S409N on the PA gene, S31N of M2 gene, N205S on the NS1 gene, and T48A on the NS2 gene (Table 2). Moreover, some amino acid substitutions were exclusively found in human HP A/H7N9, including G186I, Q226H, and Q226L on the HA gene, E119V, R292K, and R292X on the NA gene, A199S, T271A, A588I, E627K, E627X, D701N, and D701X on the PB2 gene, I368L on the PB1 gene, V33I on the NP gene, P41A on the M1 gene, and P42S on the NS1 gene (Table 2).

**Table 2.** Amino acid substitutions of HP A/H7N9 viruses from humans and LPMs.

			No. (%) of viral isolates from				
			Human HPAI H7N9		Environmental HPAI H7N9		
Segments	Amino acid substitution		V1	Total	V1	V3	Total
HA^a^	G186V/N/K	I	3 (6.7)	3 (6.3)	0 (0)	0 (0)	0 (0)
	G186V/N/K	V	42 (93.3)	45 (93.8)	36 (100)	16 (100)	58 (100)
	K193T	K	45 (100)	48 (100)	36 (100)	16 (100)	58 (100)
	N224K	N	45 (100)	48 (100)	36 (100)	16 (100)	58 (100)
	Q226L	H	2 (4.4)	2 (4.2)	0 (0)	0 (0)	0 (0)
	Q226L	L	2 (4.4)	2 (4.2)	0 (0)	0 (0)	0 (0)
	Q226L	Q	41 (91.1)	44 (91.7)	36 (100)	16 (100)	58 (100)
	G228S	G	45 (100)	48 (100)	36 (100)	16 (100)	58 (100)
NA^a^	E119V	E	45 (100)	47 (97.9)	36 (100)	16 (100)	58 (100)
	E119V	V	1 (2.2)	1 (2.1)	0 (0)	0 (0)	0 (0)
	A246T	A	45 (100)	48 (100)	36 (100)	16 (100)	58 (100)
	H274Y	H	44 (97.8)	47 (97.9)	36 (100)	16 (100)	58 (100)
	H274Y	Y	1 (2.2)	1 (2.1)	0 (0)	0 (0)	0 (0)
	R292K	R	31 (68.9)	34 (70.8)	36 (100)	16 (100)	58 (100)
	R292K	K	13 (28.9)	13 (27.1)	0 (0)	0 (0)	0 (0)
	R292K	X	1 (2.2)	1 (2.1)	0 (0)	0 (0)	0 (0)
PB2^b^	A199S	A	42 (95.5)	44 (95.7)	36 (100)	16 (100)	58 (100)
	A199S	T	2 (4.5)	2 (4.3)	0 (0)	0 (0)	0 (0)
	T271A	T	43 (97.7)	45 (97.8)	36 (100)	16 (100)	58 (100)
	T271A	A	1 (2.3)	1 (2.2)	0 (0)	0 (0)	0 (0)
	K526R	K	17 (38.6)	17 (37)	5 (13.9)	6 (37.5)	13 (22.4)
	K526R	R	27 (61.4)	29 (63)	31 (86.1)	10 (62.5)	45 (77.5)
	M535L	M	17 (38.6)	17 (37)	5 (13.9)	6 (37.5)	13 (22.4)
	M535L	L	27 (61.4)	29 (63)	31 (86.1)	10 (62.5)	45 (77.6)
	A588V	A	26 (59.1)	27 (58.7)	30 (83.3)	11 (68.8)	45 (77.6)
	A588V	I	2 (4.5)	2 (4.3)	0 (0)	0 (0)	0 (0)
	A588V	T	2 (4.5)	2 (4.3)	1 (2.8)	0 (0)	1 (1.7)
	A588V	V	14 (31.8)	15 (32.6)	5 (13.9)	5 (31.3)	12 (20.7)
	Q591K	Q	46 (104.5)	46 (100)	36 (100)	16 (100)	58 (100)
	Q591K	K	0 (0)	0 (0)	0 (0)	0 (0)	0 (0)
	E627K	E	26 (59.1)	26 (56.5)	36 (100)	16 (100)	58 (100)
	E627K	K	14 (31.8)	16 (34.8)	0 (0)	0 (0)	0 (0)
	E627K	X	4 (9.1)	4 (8.7)	0 (0)	0 (0)	0 (0)
	D701N	D	36 (81.8)	38 (82.6)	36 (100)	16 (100)	58 (100)
	D701N	N	7 (15.9)	7 (15.2)	0 (0)	0 (0)	0 (0)
	D701N	X	1 (2.3)	1 (2.2)	0 (0)	0 (0)	0 (0)
	K702R	K	35 (79.5)	37 (80.4)	31 (86.1)	16 (100)	51 (87.9)
	K702R	R	9 (20.5)	9 (19.6)	5 (13.9)	0 (0)	7 (12.1)
PB1^c^	I368V	I	1 (2.2)	1 (2.1)	0 (0)	0 (0)	1 (1.7)
	I368V	L	1 (2.2)	1 (2.1)	0 (0)	0 (0)	0 (0)
	I368V	V	43 (95.6)	45 (95.7)	36 (100)	16 (100)	56 (96.6)
PA^c^	V100A	A	23 (51.1)	24 (51.1)	28 (77.8)	11 (68.8)	43 (74.1)
	V100A	V	22 (48.9)	23 (48.9)	7 (19.4)	5 (31.3)	14 (24.1)
	V100A	T	0 (0)	0 (0)	1 (2.8)	0 (0)	1 (1.7)
	A404S	A	43 (95.6)	45 (95.7)	35 (97.2)	15 (93.8)	56 (96.6)
	A404S	S	2 (4.4)	2 (4.3)	1 (2.8)	1 (6.3)	2 (3.4)
	S409N	S	3 (6.7)	3 (6.4)	3 (8.3)	3 (18.8)	6 (10.3)
	S409N	N	42 (93.3)	44 (93.6)	33 (91.7)	13 (81.3)	52 (89.7)
NP^c^	V33I	V	43 (95.6)	45 (95.7)	36 (100)	16 (100)	58 (100)
	V33I	I	2 (4.4)	2 (4.3)	0 (0)	0 (0)	0 (0)
	I109V	I	45 (100)	46 (97.9)	36 (100)	16 (100)	56 (96.6)
	I109V	V	0 (0)	1 (2.1)	0 (0)	0 (0)	2 (3.4)
M1^c^	P41A	A	44 (97.8)	46 (97.9)	36 (100)	16 (100)	58 (100)
	P41A	S	1 (2.2)	1 (2.1)	0 (0)	0 (0)	0 (0)
	T215A	A	45 (100)	47 (100)	36 (100)	16 (100)	58 (100)
	V115I	V	44 (97.8)	46 (97.9)	36 (100)	16 (100)	58 (100)
	V115I	I	1 (2.2)	1 (2.1)	0 (0)	0 (0)	0 (0)
M2^c^	S31N	N	45 (100)	47 (100)	36 (100)	15 (93.8)	57 (98.3)
	S31N	S	0 (0)	0 (0)	0 (0)	1 (6.3)	1 (1.7)
NS1 ^c^	P42S	S	44 (97.8)	46 (97.9)	36 (100)	16 (100)	58 (100)
	P42S	A	1 (2.2)	1 (2.1)	0 (0)	0 (0)	0 (0)
	N205S	S	44 (97.8)	46 (97.9)	36 (100)	15 (93.8)	57 (98.3)
	N205S	I	1 (2.2)	1 (2.1)	0 (0)	1 (6.3)	1 (1.7)
NS2^c^	T48A	A	44 (97.8)	46 (97.9)	36 (100)	15 (93.8)	57 (98.3)
	T48A	S	1 (2.2)	1 (2.1)	0 (0)	1 (6.3)	1 (1.7)

a Variant 1, *n* = 45; HP human cases, *n* = 48.

b Variant 1, *n* = 44; HP human cases, *n* = 46

c Variant 1, *n* = 45; HP human cases, *n* = 47.

## 4. Discussion

The fifth epidemic wave of avian influenza A/H7N9 started in September 2016 with a sudden increase of human cases in seven provinces (Su et al. 2017). The first HP A/H7N9 was detected retrospectively on four markets in Heyuan, Guangdong province in November 2016, before the notification of the first case of human HP A/H7N9 infection in December 2016 (Ke et al. 2017). HP A/H7N9 was later also identified in other patients in the PRD Region, as well as the eastern and northern areas of Guangdong.

Since the first detection of HP A/H7N9 in four cities of Guangdong province, it was detected in nineteen cities in Guangdong and eleven other provinces in China. Analysis based on the HA gene suggested that HP A/H7N9 originated from the Guangdong and eastern China. It seems that HP A/H7N9 viruses spread from Guangdong to other regions (Fig. 3). This might have been due to market closure, which may have led to a shift in the market through retailers selling their live poultry in other regions or neighboring provinces (Wu et al. 2016). Genotype comparison demonstrated that, although Guangdong province accounted for 79 per cent of HP genotypes in the fifth wave, most HP genotypes are unique to Guangdong province. This suggests limited outward spread of HP A/H7N9 viruses. The reverse was true for LP, for which genome analysis revealed novel imported H7N9 viruses into Guangdong. Further analysis of six internal genes showed that the internal genes of HP A/H7N9 viruses have multiple origins, with internal gene segments from YRD-lineage H7N9 viruses from fourteen different provinces across the whole wave, which is consistent with the massive poultry import into Guangdong (Li et al. 2018). These imported LP viruses seemed to have an advantage as almost all internal segments of local PRD-lineage LP/H7N9 viruses were replaced by those of YRD-lineage H7N9 viruses. Our study suggests that live poultry trade is a key driver of genetic heterogeneity of HP H7N9 and LP H7N9 in Guangdong.

Seven different cleavage site variants (V1–V7) were identified in Guangdong in the fifth wave outbreak (Qi et al. 2018; Quan et al. 2018; Shi et al. 2018). Comparing these cleavage site motifs between human A/H7 viruses and those detected on LPMs, showed a difference in distribution of variants between LPMs and humans, suggesting V1 cleavage site may increase the replication in poultry. The high viral load makes the virus more readily transmit from birds to humans. It has been described that different cleavage site motifs can contribute to variations in virulence in HP avian influenza viruses in different hosts(Zhang et al. 2012). However, further research is needed to determine their influence on human pathogenicity and virulence. In addition, we found several mutations that have been described as potentially increasing the risk of pathogenicity and transmission in humans. A G186V amino acid substitution in the HA gene was found in 100 per cent of environmental HP A/H7N9 viruses in the present study, implying an increased viral affinity to the human-type receptor (Chen et al. 2016). Although mutations on E627K and D701N in PB2 gene were not found in environmental HP A/H7N9, mutations K526R and M535L were found in 77.5 per cent environmental HP A/H7N9 viruses, which indicated a potential enhanced virulence in mammalians (Chen et al. 2016). Mutations associated with host transmission (avian to human) were also found in the PB2 gene (12%) and the PA gene (74%–90%) in environmental HP A/H7N9. Moreover, 99 per cent of environment HP A/H7N9 acquired S31N on M2 gene, N205S on NS1, T48A on NS2, which reportedly reduces susceptibility to rimantadine and amantadine and alter the antiviral response in the hosts (Chen et al. 2016). The potential consequences of these different mutations of HP A/H7N9 virus need further investigation.

Our study was done retrospectively, but showed potential for early warning for vaccine escape variants or circulation of viruses with molecular markers for human infections or antiviral resistance (Bai 2019). With the development of next-generation sequencing, such public health translation of pathogen genomic data can become a new routine, provided costs of sequencing and analytical processes is developed for such environments (Aarestrup and Koopmans 2016; Grubaugh et al. 2019). This includes infrastructure for sharing of relevant data, even before public release (Amid 2019).

## Supplementary data

Supplementary data are available at *Virus Evolution* online.

## Data availiablility

The data used to support the findings of this study are available from the corresponding author upon request.

## Authors contributors

R.B. is a postdoctoral researcher at the Guangdong Provincial Center for Disease Control and Prevention, Guangzhou, China, whose research focuses on the epidemiology, evolution, and transmission of avian influenza viruses in Guangdong province. R.S.S. is a PhD candidate at the Erasmus Medical Centre, Rotterdam, The Netherlands, whose research focuses on the risk-based surveillance of influenza viruses in the market chain.

## Supplementary Material

veaa097_Supplementary_Data
