# Supplementary material for: Exploring utility of genomic epidemiology to trace origins of highly pathogenic influenza A/H7N9 in Guangdong
Source: Virus Evol. 2020 Dec 18;6(2):veaa097. doi: 10.1093/ve/veaa097 (PMC7758296; doi:10.1093/ve/veaa097)
Supplement: veaa097_Supplementary_Data [file veaa097_supplementary_data.zip › S table 1.docx]

Supplementary table 1. DNA accession No of avian influenza A/H7N9 and A/H9N2 isolates in present study

| **Isolate ID** | **Segments IDs** | **Isolate Name** | **Subtype** |
| --- | --- | --- | --- |
| 343888 | PB2: EPI1379820 / PB1: EPI1379821 / PA: EPI1380225 / HA: EPI1379670 / NP: EPI1380378 / NA: EPI1379745 / MP: EPI1380222 / NS: EPI1380379 | A/Env/Guangdong/C17274211/SHT/2017/3/29 | H7N9 |
| 343889 | PB2: EPI1379968 / PA: EPI1380227 / HA: EPI1379671 / NP: EPI1380380 / NA: EPI1379746 / MP: EPI1380226 / NS: EPI1380381 | A/Env/Guangdong/C17274203/SHT/2017/3/29 | H7N9 |
| 343890 | PB2: EPI1379822 / PB1: EPI1379823 / PA: EPI1380230 / HA: EPI1379672 / NP: EPI1380382 / NA: EPI1379747 / MP: EPI1380229 / NS: EPI1380383 | A/Env/Guangdong/C172790291/ZHQ/2017/2/13 | H7N9 |
| 343891 | PB2: EPI1379824 / PB1: EPI1379825 / PA: EPI1380232 / HA: EPI1379673 / NP: EPI1380384 / NA: EPI1379748 / MP: EPI1380231 / NS: EPI1380385 | A/Env/Guangdong/C17273142/ZHH/2017/2/22 | H7N9 |
| 343892 | PB2: EPI1379826 / PB1: EPI1379827 / PA: EPI1380234 / HA: EPI1379674 / NP: EPI1380386 / NA: EPI1379749 / MP: EPI1380233 / NS: EPI1380387 | A/Env/Guangdong/C17274054/SHT/2017/1/18 | H7N9 |
| 343893 | PB2: EPI1379828 / PB1: EPI1379829 / PA: EPI1380236 / HA: EPI1379675 / NP: EPI1380388 / NA: EPI1379750 / MP: EPI1380235 / NS: EPI1380389 | A/Env/Guangdong/C17060036/GZH/2017/1/10 | H7N9 |
| 343894 | PB2: EPI1379830 / PB1: EPI1379831 / PA: EPI1380238 / HA: EPI1379676 / NP: EPI1380390 / NA: EPI1379751 / MP: EPI1380237 / NS: EPI1380391 | A/Env/Guangdong/C17274013/SHT/2017/1/10 | H7N9 |
| 343895 | PB2: EPI1379832 / PB1: EPI1379833 / PA: EPI1380240 / HA: EPI1379677 / NP: EPI1380392 / NA: EPI1379752 / MP: EPI1380239 / NS: EPI1380393 | A/Env/Guangdong/C17274038/SHT/2017/1/17 | H7N9 |
| 343896 | PB2: EPI1379834 / PB1: EPI1379835 / PA: EPI1380242 / HA: EPI1379678 / NP: EPI1380394 / NA: EPI1379753 / MP: EPI1380241 / NS: EPI1380395 | A/Env/Guangdong/C17280098/HZH/2017/1/23 | H7N9 |
| 343897 | PB2: EPI1379836 / PB1: EPI1379837 / PA: EPI1380244 / HA: EPI1379679 / NP: EPI1380396 / NA: EPI1379754 / MP: EPI1380243 / NS: EPI1380397 | A/Env/Guangdong/C17280032/HZH/2017/1/10 | H7N9 |
| 343898 | PB2: EPI1379838 / PB1: EPI1379839 / PA: EPI1380246 / HA: EPI1379680 / NP: EPI1380398 / NA: EPI1379755 / MP: EPI1380245 / NS: EPI1380399 | A/Env/Guangdong/C17284128/YJ/2017/2/23 | H7N9 |
| 343899 | PB2: EPI1379840 / PB1: EPI1379841 / PA: EPI1380248 / HA: EPI1379681 / NP: EPI1380400 / NA: EPI1379756 / MP: EPI1380247 / NS: EPI1380401 | A/Env/Guangdong/C172754036/FSH/2017/1/16 | H7N9 |
| 343900 | PB2: EPI1379842 / PB1: EPI1379843 / PA: EPI1380250 / HA: EPI1379682 / NP: EPI1380402 / NA: EPI1379757 / MP: EPI1380249 / NS: EPI1380403 | A/Env/Guangdong/C172870436/ZHSH/2017/3/14 | H7N9 |
| 343901 | PB2: EPI1379844 / PB1: EPI1379845 / PA: EPI1380252 / HA: EPI1379683 / NP: EPI1380404 / NA: EPI1379758 / MP: EPI1380251 / NS: EPI1380405 | A/Env/Guangdong/C17273141/ZHH/2017/2/22 | H7N9 |
| 343902 | PB2: EPI1379846 / PB1: EPI1379847 / PA: EPI1380255 / HA: EPI1379684 / NP: EPI1380406 / NA: EPI1379759 / MP: EPI1380254 / NS: EPI1380407 | A/Env/Guangdong/C172820227/SHW/2017/3/21 | H7N9 |
| 343903 | PB2: EPI1379848 / PB1: EPI1379849 / PA: EPI1380257 / HA: EPI1379685 / NP: EPI1380408 / NA: EPI1379760 / MP: EPI1380256 / NS: EPI1380409 | A/Env/Guangdong/C172820228/SHW/2017/3/21 | H7N9 |
| 343904 | PB2: EPI1379850 / PB1: EPI1379851 / PA: EPI1380259 / HA: EPI1379686 / NP: EPI1380410 / NA: EPI1379761 / MP: EPI1380258 / NS: EPI1380411 | A/Env/Guangdong/C17280441/HZH/2017/3/14 | H7N9 |
| 343905 | PB2: EPI1379852 / PB1: EPI1379853 / PA: EPI1380261 / HA: EPI1379687 / NP: EPI1380412 / NA: EPI1379762 / MP: EPI1380260 / NS: EPI1380413 | A/Env/Guangdong/C17272200/SHG/2017/3/14 | H7N9 |
| 343906 | PB2: EPI1379854 / PB1: EPI1379855 / PA: EPI1380263 / HA: EPI1379688 / NP: EPI1380414 / NA: EPI1379763 / MP: EPI1380262 / NS: EPI1380415 | A/Env/Guangdong/C17272202/SHG/2017/3/14 | H7N9 |
| 343907 | PB2: EPI1379856 / PB1: EPI1379857 / PA: EPI1380265 / HA: EPI1379689 / NP: EPI1380416 / NA: EPI1379764 / MP: EPI1380264 / NS: EPI1380417 | A/Env/Guangdong/C172754026/FSH/2017/1/23 | H7N9 |
| 343908 | PB2: EPI1379858 / PB1: EPI1379859 / PA: EPI1380267 / HA: EPI1379690 / NP: EPI1380418 / NA: EPI1379765 / MP: EPI1380266 / NS: EPI1380419 | A/Env/Guangdong/C162755266/FSH/2016/11/28 | H7N9 |
| 343909 | PB2: EPI1379860 / PB1: EPI1379861 / PA: EPI1380269 / HA: EPI1379691 / NP: EPI1380420 / NA: EPI1379766 / MP: EPI1380268 / NS: EPI1380421 | A/Env/Guangdong/C172870495/ZHSH/2017/4/17 | H7N9 |
| 343910 | PB2: EPI1379862 / PB1: EPI1379863 / PA: EPI1380271 / HA: EPI1379692 / NP: EPI1380422 / NA: EPI1379767 / MP: EPI1380270 / NS: EPI1380423 | A/Env/Guangdong/C172870512/ZHSH/2017/5/02 | H7N9 |
| 343911 | PB2: EPI1379864 / PB1: EPI1379865 / PA: EPI1380273 / HA: EPI1379693 / NP: EPI1380424 / NA: EPI1379768 / MP: EPI1380272 / NS: EPI1380425 | A/Env/Guangdong/C172870524/ZHSH/2017/5/09 | H7N9 |
| 343912 | PB2: EPI1379866 / PB1: EPI1379867 / PA: EPI1380275 / HA: EPI1379694 / NP: EPI1380426 / NA: EPI1379769 / MP: EPI1380274 / NS: EPI1380427 | A/Env/Guangdong/C172754089/FSH/2017/2/27 | H7N9 |
| 343913 | PB2: EPI1379868 / PB1: EPI1379869 / PA: EPI1380277 / HA: EPI1379695 / NP: EPI1380428 / NA: EPI1379770 / MP: EPI1380276 / NS: EPI1380429 | A/Env/Guangdong/C172755140/FSH/2017/3/6 | H7N9 |
| 343914 | PB2: EPI1379870 / PB1: EPI1379871 / PA: EPI1380279 / HA: EPI1379696 / NP: EPI1380430 / NA: EPI1379771 / MP: EPI1380278 / NS: EPI1380431 | A/Env/Guangdong/C17272334/SHG/2017/5/9 | H7N9 |
| 343915 | PB2: EPI1379872 / PB1: EPI1379873 / PA: EPI1380281 / HA: EPI1379697 / NP: EPI1380432 / NA: EPI1379772 / MP: EPI1380280 / NS: EPI1380433 | A/Env/Guangdong/C162870990/ZHSH/2016/12/30 | H7N9 |
| 343916 | PB2: EPI1379874 / PB1: EPI1379875 / PA: EPI1380283 / HA: EPI1379698 / NP: EPI1380434 / NA: EPI1379773 / MP: EPI1380282 / NS: EPI1380435 | A/Env/Guangdong/C172790290/ZHQ/2017/2/13 | H7N9 |
| 343917 | PB2: EPI1379876 / PB1: EPI1379877 / PA: EPI1380285 / HA: EPI1379699 / NP: EPI1380436 / NA: EPI1379774 / MP: EPI1380284 / NS: EPI1380437 | A/Env/Guangdong/C172753176/FSH/2017/4/05 | H7N9 |
| 343918 | PB2: EPI1379878 / PB1: EPI1379879 / PA: EPI1380287 / HA: EPI1379700 / NP: EPI1380438 / NA: EPI1379775 / MP: EPI1380286 / NS: EPI1380439 | A/Env/Guangdong/C172790493/ZHQ/2017/4/10 | H7N9 |
| 343919 | PB2: EPI1379880 / PB1: EPI1379881 / PA: EPI1380289 / HA: EPI1379701 / NP: EPI1380440 / NA: EPI1379776 / MP: EPI1380288 / NS: EPI1380441 | A/Env/Guangdong/C172790496/ZHQ/2017/4/10 | H7N9 |
| 343920 | PB2: EPI1379882 / PB1: EPI1379883 / PA: EPI1380374 / HA: EPI1379702 / NP: EPI1380442 / NA: EPI1379777 / MP: EPI1380372 / NS: EPI1380443 | A/Env/Guangdong/C172790467/ZHQ/2017/3/28 | H7N9 |
| 343921 | PB2: EPI1379884 / PB1: EPI1379885 / PA: EPI1380291 / HA: EPI1379703 / NP: EPI1380444 / NA: EPI1379778 / MP: EPI1380290 / NS: EPI1380445 | A/Env/Guangdong/C17059241/YF/2017/04/05 | H7N9 |
| 343922 | PB2: EPI1379886 / PB1: EPI1379887 / PA: EPI1380293 / HA: EPI1379704 / NP: EPI1380446 / NA: EPI1379779 / MP: EPI1380292 / NS: EPI1380447 | A/Env/Guangdong/C17272258/SHG/2017/3/27 | H7N9 |
| 343923 | PB2: EPI1379888 / PB1: EPI1379889 / PA: EPI1380295 / HA: EPI1379705 / NP: EPI1380448 / NA: EPI1379780 / MP: EPI1380294 / NS: EPI1380449 | A/Env/Guangdong/C17276874/JM/2017/3/02 | H7N9 |
| 343924 | PB2: EPI1379890 / PB1: EPI1379891 / PA: EPI1380297 / HA: EPI1379706 / NP: EPI1380450 / NA: EPI1379781 / MP: EPI1380296 / NS: EPI1380451 | A/Env/Guangdong/C172870356/ZHSH/2017/2/13 | H7N9 |
| 343925 | PB2: EPI1379892 / PB1: EPI1379893 / PA: EPI1380299 / HA: EPI1379707 / NP: EPI1380452 / NA: EPI1379782 / MP: EPI1380298 / NS: EPI1380453 | A/Env/Guangdong/C17273272/ZHH/2017/4/06 | H7N9 |
| 343926 | PB2: EPI1379894 / PB1: EPI1379895 / PA: EPI1380301 / HA: EPI1379708 / NP: EPI1380454 / NA: EPI1379783 / MP: EPI1380300 / NS: EPI1380455 | A/Env/Guangdong/C172870432/ZHSH/2017/3/07 | H7N9 |
| 343927 | PB2: EPI1379896 / PB1: EPI1379897 / PA: EPI1380303 / HA: EPI1379709 / NP: EPI1380456 / NA: EPI1379784 / MP: EPI1380302 / NS: EPI1380457 | A/Env/Guangdong/C17280514/HZH/2017/3/20 | H7N9 |
| 343928 | PB2: EPI1379898 / PB1: EPI1379899 / PA: EPI1380305 / HA: EPI1379710 / NP: EPI1380458 / NA: EPI1379785 / MP: EPI1380304 / NS: EPI1380459 | A/Env/Guangdong/C17276803/JM/2017/3/1 | H7N9 |
| 343929 | PB2: EPI1379900 / PB1: EPI1379901 / PA: EPI1380307 / HA: EPI1379711 / NP: EPI1380460 / NA: EPI1379786 / MP: EPI1380306 / NS: EPI1380461 | A/Env/Guangdong/C17276802/JM/2017/3/01 | H7N9 |
| 343930 | PB2: EPI1379902 / PB1: EPI1379903 / PA: EPI1380309 / HA: EPI1379712 / NP: EPI1380462 / NA: EPI1379787 / MP: EPI1380308 / NS: EPI1380463 | A/Env/Guangdong/2017XN05917/GZH/2017/2/15 | H7N9 |
| 343931 | PB2: EPI1379904 / PB1: EPI1379905 / PA: EPI1380311 / HA: EPI1379713 / NP: EPI1380464 / NA: EPI1379788 / MP: EPI1380310 / NS: EPI1380465 | A/Env/Guangdong/C17278422/MM/2017/5/19 | H7N9 |
| 343932 | PB2: EPI1379906 / PB1: EPI1379907 / PA: EPI1380313 / HA: EPI1379714 / NP: EPI1380466 / NA: EPI1379789 / MP: EPI1380312 / NS: EPI1380467 | A/Env/Guangdong/C162870975/ZHSH/2016/12/30 | H7N9 |
| 343933 | PB2: EPI1379908 / PB1: EPI1379909 / PA: EPI1380315 / HA: EPI1379715 / NP: EPI1380468 / NA: EPI1379790 / MP: EPI1380314 / NS: EPI1380469 | A/Env/Guangdong/C17276659/JM/2017/2/14 | H7N9 |
| 343934 | PB2: EPI1379910 / PB1: EPI1379911 / PA: EPI1380317 / HA: EPI1379716 / NP: EPI1380470 / NA: EPI1379791 / MP: EPI1380316 / NS: EPI1380471 | A/Env/Guangdong/C172572084/FSH/2017/2/13 | H7N9 |
| 343935 | PB2: EPI1379912 / PB1: EPI1379913 / PA: EPI1380319 / HA: EPI1379717 / NP: EPI1380472 / NA: EPI1379792 / MP: EPI1380318 / NS: EPI1380473 | A/Env/Guangdong/C17281051/MZH/2017/1/14 | H7N9 |
| 343936 | PB2: EPI1379914 / PB1: EPI1379915 / PA: EPI1380321 / HA: EPI1379718 / NP: EPI1380474 / NA: EPI1379793 / MP: EPI1380320 / NS: EPI1380475 | A/Env/Guangdong/C16283223/HY/2016/11/7 | H7N9 |
| 343937 | PB2: EPI1379916 / PB1: EPI1379917 / PA: EPI1380323 / HA: EPI1379719 / NP: EPI1380476 / NA: EPI1379794 / MP: EPI1380322 / NS: EPI1380477 | A/Env/Guangdong/C17281046/MZH/2017/1/4 | H7N9 |
| 343938 | PB2: EPI1379918 / PB1: EPI1379919 / PA: EPI1380325 / HA: EPI1379720 / NP: EPI1380478 / NA: EPI1379795 / MP: EPI1380324 / NS: EPI1380479 | A/Env/Guangdong/C162811373/MZH/2016/12/26 | H7N9 |
| 343939 | PB2: EPI1379920 / PB1: EPI1379921 / PA: EPI1380327 / HA: EPI1379721 / NP: EPI1380480 / NA: EPI1379796 / MP: EPI1380326 / NS: EPI1380481 | A/Env/Guangdong/C17281268/MZH/2017/1/23 | H7N9 |
| 343940 | PB2: EPI1379922 / PB1: EPI1379923 / PA: EPI1380329 / HA: EPI1379722 / NP: EPI1380482 / NA: EPI1379797 / MP: EPI1380328 / NS: EPI1380483 | A/Env/Guangdong/C17280022/HZH/2017/1/9 | H7N9 |
| 343941 | PB2: EPI1379924 / PB1: EPI1379925 / PA: EPI1380331 / HA: EPI1379723 / NP: EPI1380484 / NA: EPI1379798 / MP: EPI1380330 / NS: EPI1380485 | A/Env/Guangdong/C172750005/FSH/2017/1/3 | H7N9 |
| 343942 | PB2: EPI1379926 / PB1: EPI1379927 / PA: EPI1380333 / HA: EPI1379724 / NP: EPI1380486 / NA: EPI1379799 / MP: EPI1380332 / NS: EPI1380487 | A/Env/Guangdong/C16276736/JM/2016/12/26 | H7N9 |
| 343943 | PB2: EPI1379928 / PB1: EPI1379929 / PA: EPI1380335 / HA: EPI1379725 / NP: EPI1380488 / NA: EPI1379800 / MP: EPI1380334 / NS: EPI1380489 | A/Env/Guangdong/C16285402/QY/2016/11/30 | H7N9 |
| 343944 | PB2: EPI1379930 / PB1: EPI1379931 / PA: EPI1380337 / HA: EPI1379726 / NP: EPI1380490 / NA: EPI1379801 / MP: EPI1380336 / NS: EPI1380491 | A/Env/Guangdong/C17281260/MZH/2017/1/23 | H7N9 |
| 343945 | PB2: EPI1379932 / PB1: EPI1379933 / PA: EPI1380339 / HA: EPI1379727 / NP: EPI1380492 / NA: EPI1379802 / MP: EPI1380338 / NS: EPI1380493 | A/Env/Guangdong/C162870978/ZHSH/2016/12/30 | H7N9 |
| 343946 | PB2: EPI1379934 / PB1: EPI1379935 / PA: EPI1380341 / HA: EPI1379728 / NP: EPI1380494 / NA: EPI1379803 / MP: EPI1380340 / NS: EPI1380495 | A/Env/Guangdong/C17281767/MZH/2017/4/7 | H7N9 |
| 343947 | PB2: EPI1379936 / PB1: EPI1379937 / PA: EPI1380343 / HA: EPI1379729 / NP: EPI1380496 / NA: EPI1379804 / MP: EPI1380342 / NS: EPI1380497 | A/Env/Guangdong/C17281770/MZH/2017/4/7 | H7N9 |
| 343948 | PB2: EPI1379938 / PB1: EPI1379939 / PA: EPI1380345 / HA: EPI1379730 / NP: EPI1380498 / NA: EPI1379805 / MP: EPI1380344 / NS: EPI1380499 | A/Env/Guangdong/C16272296/SHG/2016/12/28 | H7N9 |
| 343949 | PB2: EPI1379940 / PB1: EPI1379941 / PA: EPI1380347 / HA: EPI1379731 / NP: EPI1380500 / NA: EPI1379806 / MP: EPI1380346 / NS: EPI1380501 | A/Env/Guangdong/C162753280/FSH/2016/12/19 | H7N9 |
| 343950 | PB2: EPI1379942 / PB1: EPI1379943 / PA: EPI1380349 / HA: EPI1379732 / NP: EPI1380502 / NA: EPI1379807 / MP: EPI1380348 / NS: EPI1380503 | A/Env/Guangdong/C17289142/MZH/2017/2/24 | H7N9 |
| 343951 | PB2: EPI1379944 / PB1: EPI1379945 / PA: EPI1380351 / HA: EPI1379733 / NP: EPI1380504 / NA: EPI1379808 / MP: EPI1380350 / NS: EPI1380505 | A/Env/Guangdong/C17272045/SHG/2017/1/10 | H7N9 |
| 343952 | PB2: EPI1379946 / PB1: EPI1379947 / PA: EPI1380353 / HA: EPI1379734 / NP: EPI1380506 / NA: EPI1379809 / MP: EPI1380352 / NS: EPI1380507 | A/Env/Guangdong/C17060028/GZH/2017/1/10 | H7N9 |
| 343953 | PB2: EPI1379948 / PB1: EPI1379949 / PA: EPI1380355 / HA: EPI1379735 / NP: EPI1380508 / NA: EPI1379810 / MP: EPI1380354 / NS: EPI1380509 | A/Env/Guangdong/C17060034/GZH/2017/1/10 | H7N9 |
| 343954 | PB2: EPI1379950 / PB1: EPI1379951 / PA: EPI1380376 / HA: EPI1379736 / NP: EPI1380510 / NA: EPI1379811 / MP: EPI1380375 / NS: EPI1380511 | A/Env/Guangdong/EN17281067/MZH/2017/5/1 | H7N9 |
| 343955 | PB2: EPI1379952 / PB1: EPI1379953 / PA: EPI1380357 / HA: EPI1379737 / NP: EPI1380512 / NA: EPI1379812 / MP: EPI1380356 / NS: EPI1380513 | A/Env/Guangdong/C17280745/HZH/2017/5/16 | H7N9 |
| 343956 | PB2: EPI1379954 / PB1: EPI1379955 / PA: EPI1380359 / HA: EPI1379738 / NP: EPI1380514 / NA: EPI1379813 / MP: EPI1380358 / NS: EPI1380515 | A/Env/Guangdong/C17288460/CZH/2017/2/21 | H7N9 |
| 343957 | PB2: EPI1379956 / PB1: EPI1379957 / PA: EPI1380361 / HA: EPI1379739 / NP: EPI1380516 / NA: EPI1379814 / MP: EPI1380360 / NS: EPI1380517 | A/Env/Guangdong/C17288455/CZH/2017/2/21 | H7N9 |
| 343958 | PB2: EPI1379958 / PB1: EPI1379959 / PA: EPI1380363 / HA: EPI1379740 / NP: EPI1380518 / NA: EPI1379815 / MP: EPI1380362 / NS: EPI1380519 | A/Env/Guangdong/C17288457/CZH/2017/2/21 | H7N9 |
| 343959 | PB2: EPI1379960 / PB1: EPI1379961 / PA: EPI1380365 / HA: EPI1379741 / NP: EPI1380520 / NA: EPI1379816 / MP: EPI1380364 / NS: EPI1380521 | A/Env/Guangdong/C17288255/CZH/2017/1/25 | H7N9 |
| 343960 | PB2: EPI1379962 / PB1: EPI1379963 / PA: EPI1380367 / HA: EPI1379742 / NP: EPI1380522 / NA: EPI1379817 / MP: EPI1380366 / NS: EPI1380523 | A/Env/Guangdong/C17060026/GZH/2017/1/10 | H7N9 |
| 343961 | PB2: EPI1379964 / PB1: EPI1379965 / PA: EPI1380369 / HA: EPI1379743 / NP: EPI1380524 / NA: EPI1379818 / MP: EPI1380368 / NS: EPI1380525 | A/Env/Guangdong/C162870921/ZHSH/2016/12/27 | H7N9 |
| 343962 | PB2: EPI1379966 / PB1: EPI1379967 / PA: EPI1380371 / HA: EPI1379744 / NP: EPI1380526 / NA: EPI1379819 / MP: EPI1380370 / NS: EPI1380527 | A/Env/Guangdong/C16276734/JM/2016/12/26 | H7N9 |
| 391418 | PB2: EPI1593857 / PB1: EPI1593858 / PA: EPI1593856 / HA: EPI1593860 / NP: EPI1593853 / NA: EPI1593859 / MP: EPI1593855 / NS: EPI1593854 | A/Env/Guangdong/C16273562/ZHH/2016/12/14 | H7N9 |
| 391419 | PB2: EPI1593865 / PB1: EPI1593866 / PA: EPI1593864 / HA: EPI1593868 / NP: EPI1593861 / NA: EPI1593867 / MP: EPI1593863 / NS: EPI1593862 | A/Env/Guangdong/C16273563/ZHH/2016/12/14 | H7N9 |
| 393304 | PB2: EPI1599922 / PB1: EPI1599474 / PA: EPI1599921 / HA: EPI1599476 / NP: EPI1600591 / NA: EPI1599475 / MP: EPI1600152 / NS: EPI1600592 | A/Env/Guangdong/EN17288141/CZH/2017/4/19 | H9N2 |
| 393303 | PB2: EPI1599920 / PB1: EPI1599471 / PA: EPI1599919 / HA: EPI1599473 / NP: EPI1600589 / NA: EPI1599472 / MP: EPI1600151 / NS: EPI1600590 | A/Env/Guangdong/EN17288135/CZH/2017/4/19 | H9N2 |
| 393302 | PB2: EPI1599918 / PB1: EPI1599468 / PA: EPI1599917 / HA: EPI1599470 / NP: EPI1600587 / NA: EPI1599469 / MP: EPI1600150 / NS: EPI1600588 | A/Env/Guangdong/EN17288131/CZH/2017/4/19 | H9N2 |
| 393301 | PB2: EPI1599916 / PB1: EPI1599465 / PA: EPI1599915 / HA: EPI1599467 / NP: EPI1600585 / NA: EPI1599466 / MP: EPI1600149 / NS: EPI1600586 | A/Env/Guangdong/EN17288084/CZH/2017/3/2 | H9N2 |
| 393300 | PB2: EPI1599914 / PB1: EPI1599462 / PA: EPI1599913 / HA: EPI1599464 / NP: EPI1600583 / NA: EPI1599463 / MP: EPI1600148 / NS: EPI1600584 | A/Env/Guangdong/EN17288064/CHZH/2017/2/23 | H9N2 |
| 393299 | PB2: EPI1599912 / PB1: EPI1599459 / PA: EPI1599911 / HA: EPI1599461 / NP: EPI1600581 / NA: EPI1599460 / MP: EPI1600147 / NS: EPI1600582 | A/Env/Guangdong/EN17288057/CZH/2017/2/27 | H9N2 |
| 393298 | PB2: EPI1599910 / PB1: EPI1599456 / PA: EPI1599909 / HA: EPI1599458 / NP: EPI1600579 / NA: EPI1599457 / MP: EPI1600146 / NS: EPI1600580 | A/Env/Guangdong/EN17288039/CZH/2017/2/23 | H9N2 |
| 393297 | PB2: EPI1599908 / PB1: EPI1599453 / PA: EPI1599907 / HA: EPI1599455 / NP: EPI1600577 / NA: EPI1599454 / MP: EPI1600145 / NS: EPI1600578 | A/Env/Guangdong/EN17288035/CZH/2017/2/23 | H9N2 |
| 393296 | PB2: EPI1599906 / PB1: EPI1599450 / PA: EPI1599905 / HA: EPI1599452 / NP: EPI1600575 / NA: EPI1599451 / MP: EPI1600144 / NS: EPI1600576 | A/Env/Guangdong/EN17284314/YJ/2017/8/16 | H9N2 |
| 393295 | PB2: EPI1599904 / PB1: EPI1599447 / PA: EPI1599903 / HA: EPI1599449 / NP: EPI1600573 / NA: EPI1599448 / MP: EPI1600143 / NS: EPI1600574 | A/Env/Guangdong/EN17284245/YJ/2017/7/12 | H9N2 |
| 393294 | PB2: EPI1599902 / PB1: EPI1599444 / PA: EPI1599901 / HA: EPI1599446 / NP: EPI1600571 / NA: EPI1599445 / MP: EPI1600142 / NS: EPI1600572 | A/Env/Guangdong/EN17281370/MZH/2017/10/24 | H9N2 |
| 393293 | PB2: EPI1599900 / PB1: EPI1599441 / PA: EPI1599899 / HA: EPI1599443 / NP: EPI1600569 / NA: EPI1599442 / MP: EPI1600141 / NS: EPI1600570 | A/Env/Guangdong/EN17281360/MZH/2017/9/25 | H9N2 |
| 393292 | PB2: EPI1599898 / PB1: EPI1599438 / PA: EPI1599897 / HA: EPI1599440 / NP: EPI1600567 / NA: EPI1599439 / MP: EPI1600140 / NS: EPI1600568 | A/Env/Guangdong/EN17281358/MZH/2017/9/25 | H9N2 |
| 393291 | PB2: EPI1599896 / PB1: EPI1599435 / PA: EPI1599895 / HA: EPI1599437 / NP: EPI1600565 / NA: EPI1599436 / MP: EPI1600139 / NS: EPI1600566 | A/Env/Guangdong/EN17281356/MZH/2017/9/25 | H9N2 |
| 393290 | PB2: EPI1599894 / PB1: EPI1599432 / PA: EPI1599893 / HA: EPI1599434 / NP: EPI1600563 / NA: EPI1599433 / MP: EPI1600138 / NS: EPI1600564 | A/Env/Guangdong/EN17281354/MZH/2017/9/25 | H9N2 |
| 393289 | PB2: EPI1599892 / PB1: EPI1599429 / PA: EPI1599891 / HA: EPI1599431 / NP: EPI1600561 / NA: EPI1599430 / MP: EPI1600137 / NS: EPI1600562 | A/Env/Guangdong/EN17281352/MZH/2017/9/25 | H9N2 |
| 393288 | PB2: EPI1599890 / PB1: EPI1599426 / PA: EPI1599889 / HA: EPI1599428 / NP: EPI1600559 / NA: EPI1599427 / MP: EPI1600136 / NS: EPI1600560 | A/Env/Guangdong/EN17281350/MZH/2017/9/25 | H9N2 |
| 393287 | PB2: EPI1599888 / PB1: EPI1599423 / PA: EPI1599887 / HA: EPI1599425 / NP: EPI1600557 / NA: EPI1599424 / MP: EPI1600135 / NS: EPI1600558 | A/Env/Guangdong/EN17281348/MZH/2017/9/25 | H9N2 |
| 393286 | PB2: EPI1599886 / PB1: EPI1599420 / PA: EPI1599885 / HA: EPI1599422 / NP: EPI1600555 / NA: EPI1599421 / MP: EPI1600134 / NS: EPI1600556 | A/Env/Guangdong/EN17281346/MZH/2017/9/25 | H9N2 |
| 393285 | PB2: EPI1599884 / PB1: EPI1599417 / PA: EPI1599883 / HA: EPI1599419 / NP: EPI1600553 / NA: EPI1599418 / MP: EPI1600133 / NS: EPI1600554 | A/Env/Guangdong/EN17281344/MZH/2017/9/25 | H9N2 |
| 393284 | PB2: EPI1599882 / PB1: EPI1599414 / PA: EPI1599881 / HA: EPI1599416 / NP: EPI1600551 / NA: EPI1599415 / MP: EPI1600132 / NS: EPI1600552 | A/Env/Guangdong/EN17281338/MZH/2017/9/25 | H9N2 |
| 393283 | PB2: EPI1599880 / PB1: EPI1599411 / PA: EPI1599879 / HA: EPI1599413 / NP: EPI1600549 / NA: EPI1599412 / MP: EPI1600131 / NS: EPI1600550 | A/Env/Guangdong/EN17281336/MZH/2017/9/25 | H9N2 |
| 393282 | PB2: EPI1599878 / PB1: EPI1599408 / PA: EPI1599877 / HA: EPI1599410 / NP: EPI1600547 / NA: EPI1599409 / MP: EPI1600130 / NS: EPI1600548 | A/Env/Guangdong/EN17281330/MZH/2017/9/25 | H9N2 |
| 393281 | PB2: EPI1599876 / PB1: EPI1599405 / PA: EPI1599875 / HA: EPI1599407 / NP: EPI1600545 / NA: EPI1599406 / MP: EPI1600129 / NS: EPI1600546 | A/Env/Guangdong/EN17281247/MZH/2017/8/21 | H9N2 |
| 393280 | PB2: EPI1599874 / PB1: EPI1599402 / PA: EPI1599873 / HA: EPI1599404 / NP: EPI1600543 / NA: EPI1599403 / MP: EPI1600128 / NS: EPI1600544 | A/Env/Guangdong/EN17281243/MZH/2017/8/21 | H9N2 |
| 393279 | PB2: EPI1599872 / PB1: EPI1599399 / PA: EPI1599871 / HA: EPI1599401 / NP: EPI1600541 / NA: EPI1599400 / MP: EPI1600127 / NS: EPI1600542 | A/Env/Guangdong/EN17281240/MZH/2017/7/11 | H9N2 |
| 393278 | PB2: EPI1599870 / PB1: EPI1599396 / PA: EPI1599869 / HA: EPI1599398 / NP: EPI1600539 / NA: EPI1599397 / MP: EPI1600126 / NS: EPI1600540 | A/Env/Guangdong/EN17281224/MZH/2017/7/11 | H9N2 |
| 393277 | PB2: EPI1599868 / PB1: EPI1599393 / PA: EPI1599867 / HA: EPI1599395 / NP: EPI1600537 / NA: EPI1599394 / MP: EPI1600125 / NS: EPI1600538 | A/Env/Guangdong/EN17281222/MZH/2017/7/11 | H9N2 |
| 393276 | PB2: EPI1599866 / PB1: EPI1599390 / PA: EPI1599865 / HA: EPI1599392 / NP: EPI1600535 / NA: EPI1599391 / MP: EPI1600124 / NS: EPI1600536 | A/Env/Guangdong/EN17281220/MZH/2017/7/11 | H9N2 |
| 393275 | PB2: EPI1599864 / PB1: EPI1599387 / PA: EPI1599863 / HA: EPI1599389 / NP: EPI1600533 / NA: EPI1599388 / MP: EPI1600123 / NS: EPI1600534 | A/Env/Guangdong/EN17281214/MZH/2017/7/11 | H9N2 |
| 393274 | PB2: EPI1599862 / PB1: EPI1599384 / PA: EPI1599861 / HA: EPI1599386 / NP: EPI1600531 / NA: EPI1599385 / MP: EPI1600122 / NS: EPI1600532 | A/Env/Guangdong/EN17281204/MZH/2017/7/11 | H9N2 |
| 393273 | PB2: EPI1599860 / PB1: EPI1599381 / PA: EPI1599859 / HA: EPI1599383 / NP: EPI1600529 / NA: EPI1599382 / MP: EPI1600121 / NS: EPI1600530 | A/Env/Guangdong/EN17281194/MZH/2017/7/11 | H9N2 |
| 393272 | PB2: EPI1599858 / PB1: EPI1599378 / PA: EPI1599857 / HA: EPI1599380 / NP: EPI1600527 / NA: EPI1599379 / MP: EPI1600120 / NS: EPI1600528 | A/Env/Guangdong/EN17281180/MZH/2017/6/19 | H9N2 |
| 393271 | PB2: EPI1599856 / PB1: EPI1599375 / PA: EPI1599855 / HA: EPI1599377 / NP: EPI1600525 / NA: EPI1599376 / MP: EPI1600119 / NS: EPI1600526 | A/Env/Guangdong/EN17281164/MZH/2017/6/19 | H9N2 |
| 393270 | PB2: EPI1599854 / PB1: EPI1599372 / PA: EPI1599853 / HA: EPI1599374 / NP: EPI1600523 / NA: EPI1599373 / MP: EPI1600118 / NS: EPI1600524 | A/Env/Guangdong/EN17281148/MZH/2017/6/19 | H9N2 |
| 393269 | PB2: EPI1599852 / PB1: EPI1599369 / PA: EPI1599851 / HA: EPI1599371 / NP: EPI1600521 / NA: EPI1599370 / MP: EPI1600117 / NS: EPI1600522 | A/Env/Guangdong/EN17281146/MZH/2017/6/19 | H9N2 |
| 393268 | PB2: EPI1599850 / PB1: EPI1599366 / PA: EPI1599849 / HA: EPI1599368 / NP: EPI1600519 / NA: EPI1599367 / MP: EPI1600116 / NS: EPI1600520 | A/Env/Guangdong/EN17281144/MZH/2017/6/19 | H9N2 |
| 393267 | PB2: EPI1599848 / PB1: EPI1599363 / PA: EPI1599847 / HA: EPI1599365 / NP: EPI1600517 / NA: EPI1599364 / MP: EPI1600115 / NS: EPI1600518 | A/Env/Guangdong/EN17281106/MZH/2017/5/23 | H9N2 |
| 393266 | PB2: EPI1599846 / PB1: EPI1599360 / PA: EPI1599845 / HA: EPI1599362 / NP: EPI1600515 / NA: EPI1599361 / MP: EPI1600114 / NS: EPI1600516 | A/Env/Guangdong/EN17281060/MZH/2017/4/26 | H9N2 |
| 393265 | PB2: EPI1599844 / PB1: EPI1599357 / PA: EPI1599843 / HA: EPI1599359 / NP: EPI1600513 / NA: EPI1599358 / MP: EPI1600113 / NS: EPI1600514 | A/Env/Guangdong/EN17281058/MZH/2017/4/26 | H9N2 |
| 393264 | PB2: EPI1599842 / PB1: EPI1599354 / PA: EPI1599841 / HA: EPI1599356 / NP: EPI1600511 / NA: EPI1599355 / MP: EPI1600112 / NS: EPI1600512 | A/Env/Guangdong/EN17281016/MZH/2017/4/26 | H9N2 |
| 393263 | PB2: EPI1599840 / PB1: EPI1599351 / PA: EPI1599839 / HA: EPI1599353 / NP: EPI1600509 / NA: EPI1599352 / MP: EPI1600111 / NS: EPI1600510 | A/Env/Guangdong/EN17278529/MM/2017/11/15 | H9N2 |
| 393262 | PB2: EPI1600508 / PB1: EPI1599348 / HA: EPI1599350 / NP: EPI1600505 / NA: EPI1599349 / MP: EPI1600507 / NS: EPI1600506 | A/Env/Guangdong/EN17278471/MM/2017/10/20 | H9N2 |
| 393261 | PB2: EPI1600504 / PB1: EPI1599345 / HA: EPI1599347 / NP: EPI1600501 / NA: EPI1599346 / MP: EPI1600503 / NS: EPI1600502 | A/Env/Guangdong/EN17278463/MM/2017/10/20 | H9N2 |
| 393260 | PB2: EPI1599838 / PA: EPI1599837 / HA: EPI1599344 / NP: EPI1600499 / NA: EPI1599343 / MP: EPI1600110 / NS: EPI1600500 | A/Env/Guangdong/EN17278453/MM/2017/10/20 | H9N2 |
| 393259 | PB2: EPI1599836 / PA: EPI1599835 / HA: EPI1599342 / NP: EPI1600497 / NA: EPI1599341 / MP: EPI1600109 / NS: EPI1600498 | A/Env/Guangdong/EN17278430/MM/2017/10/20 | H9N2 |
| 393258 | PB2: EPI1599834 / PA: EPI1599833 / HA: EPI1599340 / NP: EPI1600495 / NA: EPI1599339 / MP: EPI1600108 / NS: EPI1600496 | A/Env/Guangdong/EN17278429/MM/2017/10/20 | H9N2 |
| 393257 | PB2: EPI1599832 / PB1: EPI1599336 / PA: EPI1599831 / HA: EPI1599338 / NP: EPI1600493 / NA: EPI1599337 / MP: EPI1600107 / NS: EPI1600494 | A/Env/Guangdong/EN17278427/MM/2017/10/20 | H9N2 |
| 393256 | PB2: EPI1599830 / PB1: EPI1599333 / PA: EPI1599829 / HA: EPI1599335 / NP: EPI1600491 / NA: EPI1599334 / MP: EPI1600106 / NS: EPI1600492 | A/Env/Guangdong/EN17278399/MM/2017/9/13 | H9N2 |
| 393255 | PB2: EPI1599828 / PB1: EPI1599330 / PA: EPI1599827 / HA: EPI1599332 / NP: EPI1600489 / NA: EPI1599331 / MP: EPI1600105 / NS: EPI1600490 | A/Env/Guangdong/EN17278355/MM/2017/8/9 | H9N2 |
| 393254 | PB2: EPI1599826 / PB1: EPI1599327 / PA: EPI1599825 / HA: EPI1599329 / NP: EPI1600487 / NA: EPI1599328 / MP: EPI1600104 / NS: EPI1600488 | A/Env/Guangdong/EN17278349/MM/2017/8/9 | H9N2 |
| 393253 | PB2: EPI1599824 / PB1: EPI1599324 / PA: EPI1599823 / HA: EPI1599326 / NP: EPI1600485 / NA: EPI1599325 / MP: EPI1600103 / NS: EPI1600486 | A/Env/Guangdong/EN17278328/MM/2017/8/9 | H9N2 |
| 393252 | PB2: EPI1599822 / PB1: EPI1599321 / PA: EPI1599821 / HA: EPI1599323 / NP: EPI1600483 / NA: EPI1599322 / MP: EPI1600102 / NS: EPI1600484 | A/Env/Guangdong/EN17278325/MM/2017/8/9 | H9N2 |
| 393251 | PB2: EPI1599820 / PB1: EPI1599318 / PA: EPI1599819 / HA: EPI1599320 / NP: EPI1600481 / NA: EPI1599319 / MP: EPI1600101 / NS: EPI1600482 | A/Env/Guangdong/EN17278307/MM/2017/8/9 | H9N2 |
| 393250 | PB2: EPI1599818 / PB1: EPI1599315 / PA: EPI1599817 / HA: EPI1599317 / NP: EPI1600479 / NA: EPI1599316 / MP: EPI1600100 / NS: EPI1600480 | A/Env/Guangdong/EN17278303/MM/2017/8/9 | H9N2 |
| 393249 | PB2: EPI1599816 / PB1: EPI1599312 / PA: EPI1599815 / HA: EPI1599314 / NP: EPI1600477 / NA: EPI1599313 / MP: EPI1600099 / NS: EPI1600478 | A/Env/Guangdong/EN17278280/MM/2017/7/19 | H9N2 |
| 393248 | PB2: EPI1599814 / PB1: EPI1599309 / PA: EPI1599813 / HA: EPI1599311 / NP: EPI1600475 / NA: EPI1599310 / MP: EPI1600098 / NS: EPI1600476 | A/Env/Guangdong/EN17278173/MM/2017/5/12 | H9N2 |
| 393247 | PB2: EPI1599812 / PB1: EPI1599306 / PA: EPI1599811 / HA: EPI1599308 / NP: EPI1600473 / NA: EPI1599307 / MP: EPI1600097 / NS: EPI1600474 | A/Env/Guangdong/EN17278172/MM/2017/5/12 | H9N2 |
| 393246 | PB2: EPI1599810 / PB1: EPI1599303 / PA: EPI1599809 / HA: EPI1599305 / NP: EPI1600471 / NA: EPI1599304 / MP: EPI1600096 / NS: EPI1600472 | A/Env/Guangdong/EN17275107/FSH/2017/5/18 | H9N2 |
| 393245 | PB2: EPI1599808 / PB1: EPI1599300 / PA: EPI1599807 / HA: EPI1599302 / NP: EPI1600469 / NA: EPI1599301 / MP: EPI1600095 / NS: EPI1600470 | A/Env/Guangdong/EN17275049/FSH/2017/4/26 | H9N2 |
| 393243 | PB2: EPI1599804 / PB1: EPI1599295 / PA: EPI1599803 / HA: EPI1599297 / NP: EPI1600465 / NA: EPI1599296 / MP: EPI1600093 / NS: EPI1600466 | A/Env/Guangdong/EN17260028/GZH/2017/3/28 | H9N2 |
| 393242 | PB2: EPI1599802 / PB1: EPI1599292 / PA: EPI1599801 / HA: EPI1599294 / NP: EPI1600463 / NA: EPI1599293 / MP: EPI1600092 / NS: EPI1600464 | A/Env/Guangdong/EN17260026/GZH/2017/3/28 | H9N2 |
| 393241 | PB2: EPI1599800 / PB1: EPI1599289 / PA: EPI1599799 / HA: EPI1599291 / NP: EPI1600461 / NA: EPI1599290 / MP: EPI1600091 / NS: EPI1600462 | A/Env/Guangdong/EN17260009/GZH/2017/3/28 | H9N2 |
| 393240 | PB2: EPI1599798 / PB1: EPI1599286 / PA: EPI1599797 / HA: EPI1599288 / NP: EPI1600459 / NA: EPI1599287 / MP: EPI1600090 / NS: EPI1600460 | A/Env/Guangdong/EN17260008/GZH/2017/3/28 | H9N2 |
| 393239 | PB2: EPI1599796 / PB1: EPI1599283 / PA: EPI1599795 / HA: EPI1599285 / NP: EPI1600457 / NA: EPI1599284 / MP: EPI1600089 / NS: EPI1600458 | A/Env/Guangdong/EN17260007/GZH/2017/3/28 | H9N2 |
| 393238 | PB2: EPI1599794 / PB1: EPI1599280 / PA: EPI1599793 / HA: EPI1599282 / NP: EPI1600455 / NA: EPI1599281 / MP: EPI1600088 / NS: EPI1600456 | A/Env/Guangdong/EN17260006/GZH/2017/3/28 | H9N2 |
| 393237 | PB2: EPI1599792 / PB1: EPI1599277 / PA: EPI1599791 / HA: EPI1599279 / NP: EPI1600453 / NA: EPI1599278 / MP: EPI1600087 / NS: EPI1600454 | A/Env/Guangdong/EN1706260045/GZH/2017/7/10 | H9N2 |
| 393236 | PB2: EPI1599790 / PB1: EPI1599274 / PA: EPI1599789 / HA: EPI1599276 / NP: EPI1600451 / NA: EPI1599275 / MP: EPI1600086 / NS: EPI1600452 | A/Env/Guangdong/EN1706260044/GZH/2017/7/10 | H9N2 |
| 393235 | PB2: EPI1599788 / PB1: EPI1599271 / PA: EPI1599787 / HA: EPI1599273 / NP: EPI1600449 / NA: EPI1599272 / MP: EPI1600085 / NS: EPI1600450 | A/Env/Guangdong/EN1706260015/GZH/2017/7/11 | H9N2 |
| 393234 | PB2: EPI1599786 / PB1: EPI1599268 / PA: EPI1599785 / HA: EPI1599270 / NP: EPI1600447 / NA: EPI1599269 / MP: EPI1600084 / NS: EPI1600448 | A/Env/Guangdong/EN1704260043/GZH/2017/4/24 | H9N2 |
| 393233 | PB2: EPI1599784 / PB1: EPI1599265 / PA: EPI1599783 / HA: EPI1599267 / NP: EPI1600445 / NA: EPI1599266 / MP: EPI1600083 / NS: EPI1600446 | A/Env/Guangdong/EN1704260021/GZH/2017/4/24 | H9N2 |
| 393232 | PB2: EPI1599782 / PB1: EPI1599262 / PA: EPI1599781 / HA: EPI1599264 / NP: EPI1600443 / NA: EPI1599263 / MP: EPI1600082 / NS: EPI1600444 | A/Env/Guangdong/C17289266/JY/2017/4/12 | H9N2 |
| 393231 | PB2: EPI1599780 / PB1: EPI1599259 / PA: EPI1599779 / HA: EPI1599261 / NP: EPI1600441 / NA: EPI1599260 / MP: EPI1600081 / NS: EPI1600442 | A/Env/Guangdong/C172861686/DG/2017/5/31 | H9N2 |
| 393230 | PB2: EPI1599778 / PB1: EPI1599256 / PA: EPI1599777 / HA: EPI1599258 / NP: EPI1600439 / NA: EPI1599257 / MP: EPI1600080 / NS: EPI1600440 | A/Env/Guangdong/C172861678/DG/2017/5/31 | H9N2 |
| 393229 | PB2: EPI1599776 / PB1: EPI1599253 / PA: EPI1599775 / HA: EPI1599255 / NP: EPI1600437 / NA: EPI1599254 / MP: EPI1600079 / NS: EPI1600438 | A/Env/Guangdong/C172861678/DG/2017/05/31 | H9N2 |
| 393228 | PB2: EPI1599774 / PB1: EPI1599250 / PA: EPI1599773 / HA: EPI1599252 / NP: EPI1600435 / NA: EPI1599251 / MP: EPI1600078 / NS: EPI1600436 | A/Env/Guangdong/C172811486/MZH/2017/10/16 | H9N2 |
| 393227 | PB2: EPI1599772 / PA: EPI1599771 / HA: EPI1599249 / NP: EPI1600433 / NA: EPI1599248 / MP: EPI1600077 / NS: EPI1600434 | A/Env/Guangdong/C172811395/MZH/2017/10/16 | H9N2 |
| 393226 | PB2: EPI1599770 / PB1: EPI1599245 / PA: EPI1599769 / HA: EPI1599247 / NP: EPI1600431 / NA: EPI1599246 / MP: EPI1600076 / NS: EPI1600432 | A/Env/Guangdong/C172811390/MZH/2017/10/16 | H9N2 |
| 393225 | PB2: EPI1599768 / PB1: EPI1599242 / PA: EPI1599767 / HA: EPI1599244 / NP: EPI1600429 / NA: EPI1599243 / MP: EPI1600075 / NS: EPI1600430 | A/Env/Guangdong/C17279821/ZHQ/2017/10/23 | H9N2 |
| 393224 | PB2: EPI1599766 / PB1: EPI1599239 / PA: EPI1599765 / HA: EPI1599241 / NP: EPI1600427 / NA: EPI1599240 / MP: EPI1600074 / NS: EPI1600428 | A/Env/Guangdong/C172790586/ZHQ/2017/5/8 | H9N2 |
| 393223 | PB2: EPI1599764 / PB1: EPI1599236 / PA: EPI1599763 / HA: EPI1599238 / NP: EPI1600425 / NA: EPI1599237 / MP: EPI1600073 / NS: EPI1600426 | A/Env/Guangdong/C172790585/ZHQ/2017/5/8 | H9N2 |
| 393222 | PB2: EPI1599762 / PB1: EPI1599233 / PA: EPI1599761 / HA: EPI1599235 / NP: EPI1600423 / NA: EPI1599234 / MP: EPI1600072 / NS: EPI1600424 | A/Env/Guangdong/C172790557/ZHQ/2017/4/24 | H9N2 |
| 393221 | PB2: EPI1599760 / PB1: EPI1599230 / PA: EPI1599759 / HA: EPI1599232 / NP: EPI1600421 / NA: EPI1599231 / MP: EPI1600071 / NS: EPI1600422 | A/Env/Guangdong/C172790406/ZHQ/2017/3/6 | H9N2 |
| 393220 | PB2: EPI1599758 / PB1: EPI1599227 / PA: EPI1599757 / HA: EPI1599229 / NP: EPI1600419 / NA: EPI1599228 / MP: EPI1600070 / NS: EPI1600420 | A/Env/Guangdong/C172790404/ZHQ/2017/3/6 | H9N2 |
| 393219 | PB2: EPI1599756 / PB1: EPI1599224 / PA: EPI1599755 / HA: EPI1599226 / NP: EPI1600417 / NA: EPI1599225 / MP: EPI1600069 / NS: EPI1600418 | A/Env/Guangdong/C172790398/ZHQ/2017/3/6 | H9N2 |
| 393218 | PB2: EPI1599754 / PB1: EPI1599221 / PA: EPI1599753 / HA: EPI1599223 / NP: EPI1600415 / NA: EPI1599222 / MP: EPI1600068 / NS: EPI1600416 | A/Env/Guangdong/C17277173/ZHJ/2017/3/13 | H9N2 |
| 393217 | PB2: EPI1599752 / PB1: EPI1599218 / PA: EPI1599751 / HA: EPI1599220 / NP: EPI1600413 / NA: EPI1599219 / MP: EPI1600067 / NS: EPI1600414 | A/Env/Guangdong/C17277083/ZHJ/2017/2/28 | H9N2 |
| 393216 | PB2: EPI1599750 / PB1: EPI1599215 / PA: EPI1599749 / HA: EPI1599217 / NP: EPI1600411 / NA: EPI1599216 / MP: EPI1600066 / NS: EPI1600412 | A/Env/Guangdong/C17277053/ZHJ/2017/2/6 | H9N2 |
| 393215 | PB2: EPI1599748 / PB1: EPI1599212 / PA: EPI1599747 / HA: EPI1599214 / NP: EPI1600409 / NA: EPI1599213 / MP: EPI1600065 / NS: EPI1600410 | A/Env/Guangdong/C172761219/JM/2017/3/21 | H9N2 |
| 393214 | PB2: EPI1599746 / PB1: EPI1599209 / PA: EPI1599745 / HA: EPI1599211 / NP: EPI1600407 / NA: EPI1599210 / MP: EPI1600064 / NS: EPI1600408 | A/Env/Guangdong/C17274138/SHT/2017/2/28 | H9N2 |
| 393213 | PB2: EPI1599744 / PB1: EPI1599206 / PA: EPI1599743 / HA: EPI1599208 / NP: EPI1600405 / NA: EPI1599207 / MP: EPI1600063 / NS: EPI1600406 | A/Env/Guangdong/C17273316/ZHH/2017/4/18 | H9N2 |
| 393212 | PB2: EPI1599742 / PA: EPI1599741 / HA: EPI1599205 / NP: EPI1600403 / NA: EPI1599204 / MP: EPI1600062 / NS: EPI1600404 | A/Env/Guangdong/C17272380/SHG/2017/11/14 | H9N2 |
| 393211 | PB2: EPI1599740 / PB1: EPI1599201 / PA: EPI1599739 / HA: EPI1599203 / NP: EPI1600401 / NA: EPI1599202 / MP: EPI1600061 / NS: EPI1600402 | A/Env/Guangdong/C17272195/SHG/2017/3/14 | H9N2 |
| 393309 | PB2: EPI1599932 / PB1: EPI1599489 / PA: EPI1599931 / HA: EPI1599491 / NP: EPI1600601 / NA: EPI1599490 / MP: EPI1600157 / NS: EPI1600602 | A/Francolin/Guangdong/PO17260072/GZH/2017/3/29 | H9N2 |
| 393308 | PB2: EPI1599930 / PB1: EPI1599486 / PA: EPI1599929 / HA: EPI1599488 / NP: EPI1600599 / NA: EPI1599487 / MP: EPI1600156 / NS: EPI1600600 | A/Francolin/Guangdong/PO17260070/GZH/2017/3/29 | H9N2 |
| 393307 | PB2: EPI1599928 / PB1: EPI1599483 / PA: EPI1599927 / HA: EPI1599485 / NP: EPI1600597 / NA: EPI1599484 / MP: EPI1600155 / NS: EPI1600598 | A/Francolin/Guangdong/PO17260016/GZH/2017/3/28 | H9N2 |
| 393306 | PB2: EPI1599926 / PB1: EPI1599480 / PA: EPI1599925 / HA: EPI1599482 / NP: EPI1600595 / NA: EPI1599481 / MP: EPI1600154 / NS: EPI1600596 | A/Francolin/Guangdong/PO17260015/GZH/2017/3/28 | H9N2 |
| 393305 | PB2: EPI1599924 / PB1: EPI1599477 / PA: EPI1599923 / HA: EPI1599479 / NP: EPI1600593 / NA: EPI1599478 / MP: EPI1600153 / NS: EPI1600594 | A/Francolin/Guangdong/PO17260014/GZH/2017/3/28 | H9N2 |
| 393210 | PB2: EPI1599738 / PB1: EPI1599198 / PA: EPI1599737 / HA: EPI1599200 / NP: EPI1600399 / NA: EPI1599199 / MP: EPI1600060 / NS: EPI1600400 | A/Chicken/Guangdong/PO17288091/CZH/2017/7/4 | H9N2 |
| 393209 | PB2: EPI1599736 / PB1: EPI1599195 / PA: EPI1599735 / HA: EPI1599197 / NP: EPI1600397 / NA: EPI1599196 / MP: EPI1600059 / NS: EPI1600398 | A/Chicken/Guangdong/PO17288077/CZH/2017/6/20 | H9N2 |
| 393208 | PB2: EPI1599734 / PB1: EPI1599192 / PA: EPI1599733 / HA: EPI1599194 / NP: EPI1600395 / NA: EPI1599193 / MP: EPI1600058 / NS: EPI1600396 | A/Chicken/Guangdong/PO17288056/CZH/2017/6/19 | H9N2 |
| 393207 | PB2: EPI1599732 / PB1: EPI1599189 / PA: EPI1599731 / HA: EPI1599191 / NP: EPI1600393 / NA: EPI1599190 / MP: EPI1600057 / NS: EPI1600394 | A/Chicken/Guangdong/PO17288054/CZH/2017/6/19 | H9N2 |
| 393206 | PB2: EPI1599730 / PB1: EPI1599186 / PA: EPI1599729 / HA: EPI1599188 / NP: EPI1600391 / NA: EPI1599187 / MP: EPI1600056 / NS: EPI1600392 | A/Chicken/Guangdong/PO17288052/CZH/2017/6/19 | H9N2 |
| 393205 | PB2: EPI1599728 / PB1: EPI1599183 / PA: EPI1599727 / HA: EPI1599185 / NP: EPI1600389 / NA: EPI1599184 / MP: EPI1600055 / NS: EPI1600390 | A/Chicken/Guangdong/PO17288050/CZH/2017/6/19 | H9N2 |
| 393204 | PB2: EPI1599726 / PB1: EPI1599180 / PA: EPI1599725 / HA: EPI1599182 / NP: EPI1600387 / NA: EPI1599181 / MP: EPI1600054 / NS: EPI1600388 | A/Chicken/Guangdong/PO17288048/CZH/2017/6/19 | H9N2 |
| 393203 | PB2: EPI1599724 / PB1: EPI1599177 / PA: EPI1599723 / HA: EPI1599179 / NP: EPI1600385 / NA: EPI1599178 / MP: EPI1600053 / NS: EPI1600386 | A/Chicken/Guangdong/PO17288046/CZH/2017/6/19 | H9N2 |
| 393202 | PB2: EPI1599722 / PB1: EPI1599174 / PA: EPI1599721 / HA: EPI1599176 / NP: EPI1600383 / NA: EPI1599175 / MP: EPI1600052 / NS: EPI1600384 | A/Chicken/Guangdong/PO17288036/CZH/2017/6/19 | H9N2 |
| 393201 | PB2: EPI1599720 / PB1: EPI1599171 / PA: EPI1599719 / HA: EPI1599173 / NP: EPI1600381 / NA: EPI1599172 / MP: EPI1600051 / NS: EPI1600382 | A/Chicken/Guangdong/PO17288034/CZH/2017/6/19 | H9N2 |
| 393200 | PB2: EPI1599718 / PB1: EPI1599168 / PA: EPI1599717 / HA: EPI1599170 / NP: EPI1600379 / NA: EPI1599169 / MP: EPI1600050 / NS: EPI1600380 | A/Chicken/Guangdong/PO17288032/CZH/2017/6/19 | H9N2 |
| 393199 | PB2: EPI1599716 / PB1: EPI1599165 / PA: EPI1599715 / HA: EPI1599167 / NP: EPI1600377 / NA: EPI1599166 / MP: EPI1600049 / NS: EPI1600378 | A/Chicken/Guangdong/PO17288017/CZH/2017/2/22 | H9N2 |
| 393198 | PB2: EPI1599714 / PB1: EPI1599162 / PA: EPI1599713 / HA: EPI1599164 / NP: EPI1600375 / NA: EPI1599163 / MP: EPI1600048 / NS: EPI1600376 | A/Chicken/Guangdong/PO17288011/CZH/2017/2/22 | H9N2 |
| 393197 | PB2: EPI1599712 / PB1: EPI1599159 / PA: EPI1599711 / HA: EPI1599161 / NP: EPI1600373 / NA: EPI1599160 / MP: EPI1600047 / NS: EPI1600374 | A/Chicken/Guangdong/PO17288001/CZH/2017/2/22 | H9N2 |
| 393196 | PB2: EPI1599710 / PB1: EPI1599156 / PA: EPI1599709 / HA: EPI1599158 / NP: EPI1600371 / NA: EPI1599157 / MP: EPI1600046 / NS: EPI1600372 | A/Chicken/Guangdong/PO17284416/YJ/2017/9/14 | H9N2 |
| 393195 | PB2: EPI1599708 / PB1: EPI1599153 / PA: EPI1599707 / HA: EPI1599155 / NP: EPI1600369 / NA: EPI1599154 / MP: EPI1600045 / NS: EPI1600370 | A/Chicken/Guangdong/PO17284411/YJ/2017/9/14 | H9N2 |
| 393194 | PB2: EPI1599706 / PB1: EPI1599150 / PA: EPI1599705 / HA: EPI1599152 / NP: EPI1600367 / NA: EPI1599151 / MP: EPI1600044 / NS: EPI1600368 | A/Chicken/Guangdong/PO17284335/YJ/2017/8/16 | H9N2 |
| 393193 | PB2: EPI1599704 / PB1: EPI1599147 / PA: EPI1599703 / HA: EPI1599149 / NP: EPI1600365 / NA: EPI1599148 / MP: EPI1600043 / NS: EPI1600366 | A/Chicken/Guangdong/PO17284329/YJ/2017/8/16 | H9N2 |
| 393192 | PB2: EPI1599702 / PB1: EPI1599144 / PA: EPI1599701 / HA: EPI1599146 / NP: EPI1600363 / NA: EPI1599145 / MP: EPI1600042 / NS: EPI1600364 | A/Chicken/Guangdong/PO17284294/YJ/2017/7/12 | H9N2 |
| 393191 | PB2: EPI1599700 / PB1: EPI1599141 / PA: EPI1599699 / HA: EPI1599143 / NP: EPI1600361 / NA: EPI1599142 / MP: EPI1600041 / NS: EPI1600362 | A/Chicken/Guangdong/PO17284114/YJ/2017/4/11 | H9N2 |
| 393190 | PB2: EPI1599698 / PB1: EPI1599138 / PA: EPI1599697 / HA: EPI1599140 / NP: EPI1600359 / NA: EPI1599139 / MP: EPI1600040 / NS: EPI1600360 | A/Chicken/Guangdong/PO17284109/YJ/2017/4/11 | H9N2 |
| 393189 | PB2: EPI1599696 / PB1: EPI1599135 / PA: EPI1599695 / HA: EPI1599137 / NP: EPI1600357 / NA: EPI1599136 / MP: EPI1600039 / NS: EPI1600358 | A/Chicken/Guangdong/PO17284014/YJ/2017/3/16 | H9N2 |
| 393188 | PB2: EPI1599694 / PB1: EPI1599132 / PA: EPI1599693 / HA: EPI1599134 / NP: EPI1600355 / NA: EPI1599133 / MP: EPI1600038 / NS: EPI1600356 | A/Chicken/Guangdong/PO17281360/MZH/2017/9/25 | H9N2 |
| 393187 | PB2: EPI1599692 / PB1: EPI1599129 / PA: EPI1599691 / HA: EPI1599131 / NP: EPI1600353 / NA: EPI1599130 / MP: EPI1600037 / NS: EPI1600354 | A/Chicken/Guangdong/PO17281358/MZH/2017/9/25 | H9N2 |
| 393186 | PB2: EPI1599690 / PB1: EPI1599126 / PA: EPI1599689 / HA: EPI1599128 / NP: EPI1600351 / NA: EPI1599127 / MP: EPI1600036 / NS: EPI1600352 | A/Chicken/Guangdong/PO17281356/MZH/2017/9/25 | H9N2 |
| 393185 | PB2: EPI1599688 / PB1: EPI1599123 / PA: EPI1599687 / HA: EPI1599125 / NP: EPI1600349 / NA: EPI1599124 / MP: EPI1600035 / NS: EPI1600350 | A/Chicken/Guangdong/PO17281354/MZH/2017/9/25 | H9N2 |
| 393184 | PB2: EPI1599686 / PB1: EPI1599120 / PA: EPI1599685 / HA: EPI1599122 / NP: EPI1600347 / NA: EPI1599121 / MP: EPI1600034 / NS: EPI1600348 | A/Chicken/Guangdong/PO17281352/MZH/2017/9/25 | H9N2 |
| 393183 | PB2: EPI1599684 / PB1: EPI1599117 / PA: EPI1599683 / HA: EPI1599119 / NP: EPI1600345 / NA: EPI1599118 / MP: EPI1600033 / NS: EPI1600346 | A/Chicken/Guangdong/PO17281350/MZH/2017/9/25 | H9N2 |
| 393182 | PB2: EPI1599682 / PB1: EPI1599114 / PA: EPI1599681 / HA: EPI1599116 / NP: EPI1600343 / NA: EPI1599115 / MP: EPI1600032 / NS: EPI1600344 | A/Chicken/Guangdong/PO17281340/MZH/2017/9/25 | H9N2 |
| 393181 | PB2: EPI1599680 / PB1: EPI1599111 / PA: EPI1599679 / HA: EPI1599113 / NP: EPI1600341 / NA: EPI1599112 / MP: EPI1600031 / NS: EPI1600342 | A/Chicken/Guangdong/PO17281338/MZH/2017/9/25 | H9N2 |
| 393180 | PB2: EPI1599678 / PB1: EPI1599108 / PA: EPI1599677 / HA: EPI1599110 / NP: EPI1600339 / NA: EPI1599109 / MP: EPI1600030 / NS: EPI1600340 | A/Chicken/Guangdong/PO17281336/MZH/2017/9/25 | H9N2 |
| 393179 | PB2: EPI1599676 / PB1: EPI1599105 / PA: EPI1599675 / HA: EPI1599107 / NP: EPI1600337 / NA: EPI1599106 / MP: EPI1600029 / NS: EPI1600338 | A/Chicken/Guangdong/PO17281330/MZH/2017/9/25 | H9N2 |
| 393178 | PB2: EPI1599674 / PB1: EPI1599102 / PA: EPI1599673 / HA: EPI1599104 / NP: EPI1600335 / NA: EPI1599103 / MP: EPI1600028 / NS: EPI1600336 | A/Chicken/Guangdong/PO17281322/MZH/2017/9/25 | H9N2 |
| 393177 | PB2: EPI1599672 / PB1: EPI1599099 / PA: EPI1599671 / HA: EPI1599101 / NP: EPI1600333 / NA: EPI1599100 / MP: EPI1600027 / NS: EPI1600334 | A/Chicken/Guangdong/PO17281314/MZH/2017/9/25 | H9N2 |
| 393176 | PB2: EPI1599670 / PB1: EPI1599096 / PA: EPI1599669 / HA: EPI1599098 / NP: EPI1600331 / NA: EPI1599097 / MP: EPI1600026 / NS: EPI1600332 | A/Chicken/Guangdong/PO17281305/MZH/2017/9/25 | H9N2 |
| 393175 | PB2: EPI1599668 / PB1: EPI1599093 / PA: EPI1599667 / HA: EPI1599095 / NP: EPI1600329 / NA: EPI1599094 / MP: EPI1600025 / NS: EPI1600330 | A/Chicken/Guangdong/PO17281284/MZH/2017/8/21 | H9N2 |
| 393174 | PB2: EPI1599666 / PB1: EPI1599090 / PA: EPI1599665 / HA: EPI1599092 / NP: EPI1600327 / NA: EPI1599091 / MP: EPI1600024 / NS: EPI1600328 | A/Chicken/Guangdong/PO17281280/MZH/2017/8/21 | H9N2 |
| 393173 | PB2: EPI1599664 / PB1: EPI1599087 / PA: EPI1599663 / HA: EPI1599089 / NP: EPI1600325 / NA: EPI1599088 / MP: EPI1600023 / NS: EPI1600326 | A/Chicken/Guangdong/PO17281276/MZH/2017/8/21 | H9N2 |
| 393172 | PB2: EPI1599662 / PB1: EPI1599084 / PA: EPI1599661 / HA: EPI1599086 / NP: EPI1600323 / NA: EPI1599085 / MP: EPI1600022 / NS: EPI1600324 | A/Chicken/Guangdong/PO17281266/MZH/2017/8/21 | H9N2 |
| 393171 | PB2: EPI1599660 / PB1: EPI1599081 / PA: EPI1599659 / HA: EPI1599083 / NP: EPI1600321 / NA: EPI1599082 / MP: EPI1600021 / NS: EPI1600322 | A/Chicken/Guangdong/PO17281248/MZH/2017/8/21 | H9N2 |
| 393170 | PB2: EPI1599658 / PB1: EPI1599078 / PA: EPI1599657 / HA: EPI1599080 / NP: EPI1600319 / NA: EPI1599079 / MP: EPI1600020 / NS: EPI1600320 | A/Chicken/Guangdong/PO17281246/MZH/2017/8/21 | H9N2 |
| 393169 | PB2: EPI1599656 / PB1: EPI1599075 / PA: EPI1599655 / HA: EPI1599077 / NP: EPI1600317 / NA: EPI1599076 / MP: EPI1600019 / NS: EPI1600318 | A/Chicken/Guangdong/PO17281242/MZH/2017/8/21 | H9N2 |
| 393168 | PB2: EPI1599654 / PB1: EPI1599072 / PA: EPI1599653 / HA: EPI1599074 / NP: EPI1600315 / NA: EPI1599073 / MP: EPI1600018 / NS: EPI1600316 | A/Chicken/Guangdong/PO17281230/MZH/2017/7/11 | H9N2 |
| 393167 | PB2: EPI1599652 / PB1: EPI1599069 / PA: EPI1599651 / HA: EPI1599071 / NP: EPI1600313 / NA: EPI1599070 / MP: EPI1600017 / NS: EPI1600314 | A/Chicken/Guangdong/PO17281228/MZH/2017/7/11 | H9N2 |
| 393166 | PB2: EPI1599650 / PB1: EPI1599066 / PA: EPI1599649 / HA: EPI1599068 / NP: EPI1600311 / NA: EPI1599067 / MP: EPI1600016 / NS: EPI1600312 | A/Chicken/Guangdong/PO17281224/MZH/2017/7/11 | H9N2 |
| 393165 | PB2: EPI1599648 / PB1: EPI1599063 / PA: EPI1599647 / HA: EPI1599065 / NP: EPI1600309 / NA: EPI1599064 / MP: EPI1600015 / NS: EPI1600310 | A/Chicken/Guangdong/PO17281218/MZH/2017/7/11 | H9N2 |
| 393164 | PB2: EPI1599646 / PB1: EPI1599060 / PA: EPI1599645 / HA: EPI1599062 / NP: EPI1600307 / NA: EPI1599061 / MP: EPI1600014 / NS: EPI1600308 | A/Chicken/Guangdong/PO17281212/MZH/2017/7/11 | H9N2 |
| 393163 | PB2: EPI1599644 / PB1: EPI1599057 / PA: EPI1599643 / HA: EPI1599059 / NP: EPI1600305 / NA: EPI1599058 / MP: EPI1600013 / NS: EPI1600306 | A/Chicken/Guangdong/PO17281210/MZH/2017/7/11 | H9N2 |
| 393162 | PB2: EPI1599642 / PB1: EPI1599054 / PA: EPI1599641 / HA: EPI1599056 / NP: EPI1600303 / NA: EPI1599055 / MP: EPI1600012 / NS: EPI1600304 | A/Chicken/Guangdong/PO17281204/MZH/2017/7/11 | H9N2 |
| 393161 | PB2: EPI1599640 / PB1: EPI1599051 / PA: EPI1599639 / HA: EPI1599053 / NP: EPI1600301 / NA: EPI1599052 / MP: EPI1600011 / NS: EPI1600302 | A/Chicken/Guangdong/PO17281188/MZH/2017/7/11 | H9N2 |
| 393160 | PB2: EPI1599638 / PB1: EPI1599048 / PA: EPI1599637 / HA: EPI1599050 / NP: EPI1600299 / NA: EPI1599049 / MP: EPI1600010 / NS: EPI1600300 | A/Chicken/Guangdong/PO17281186/MZH/2017/7/11 | H9N2 |
| 393159 | PB2: EPI1599636 / PB1: EPI1599045 / PA: EPI1599635 / HA: EPI1599047 / NP: EPI1600297 / NA: EPI1599046 / MP: EPI1600009 / NS: EPI1600298 | A/Chicken/Guangdong/PO17281168/MZH/2017/6/19 | H9N2 |
| 393158 | PB2: EPI1599634 / PB1: EPI1599042 / PA: EPI1599633 / HA: EPI1599044 / NP: EPI1600295 / NA: EPI1599043 / MP: EPI1600008 / NS: EPI1600296 | A/Chicken/Guangdong/PO17281162/MZH/2017/6/19 | H9N2 |
| 393157 | PB2: EPI1599632 / PB1: EPI1599039 / PA: EPI1599631 / HA: EPI1599041 / NP: EPI1600293 / NA: EPI1599040 / MP: EPI1600007 / NS: EPI1600294 | A/Chicken/Guangdong/PO17281159/MZH/2017/6/19 | H9N2 |
| 393156 | PB2: EPI1599630 / PB1: EPI1599036 / PA: EPI1599629 / HA: EPI1599038 / NP: EPI1600291 / NA: EPI1599037 / MP: EPI1600006 / NS: EPI1600292 | A/Chicken/Guangdong/PO17281146/MZH/2017/6/19 | H9N2 |
| 393155 | PB2: EPI1599628 / PB1: EPI1599033 / PA: EPI1599627 / HA: EPI1599035 / NP: EPI1600289 / NA: EPI1599034 / MP: EPI1600005 / NS: EPI1600290 | A/Chicken/Guangdong/PO17281144/MZH/2017/6/19 | H9N2 |
| 393154 | PB2: EPI1599626 / PB1: EPI1599030 / PA: EPI1599625 / HA: EPI1599032 / NP: EPI1600287 / NA: EPI1599031 / MP: EPI1600004 / NS: EPI1600288 | A/Chicken/Guangdong/PO17281142/MZH/2017/6/19 | H9N2 |
| 393153 | PB2: EPI1599624 / PB1: EPI1599027 / PA: EPI1599623 / HA: EPI1599029 / NP: EPI1600285 / NA: EPI1599028 / MP: EPI1600003 / NS: EPI1600286 | A/Chicken/Guangdong/PO17281140/MZH/2017/6/19 | H9N2 |
| 393152 | PB2: EPI1599622 / PB1: EPI1599024 / PA: EPI1599621 / HA: EPI1599026 / NP: EPI1600283 / NA: EPI1599025 / MP: EPI1600002 / NS: EPI1600284 | A/Chicken/Guangdong/PO17281138/MZH/2017/6/19 | H9N2 |
| 393151 | PB2: EPI1599620 / PB1: EPI1599021 / PA: EPI1599619 / HA: EPI1599023 / NP: EPI1600281 / NA: EPI1599022 / MP: EPI1600001 / NS: EPI1600282 | A/Chicken/Guangdong/PO17281136/MZH/2017/6/19 | H9N2 |
| 393150 | PB2: EPI1599618 / PB1: EPI1599018 / PA: EPI1599617 / HA: EPI1599020 / NP: EPI1600279 / NA: EPI1599019 / MP: EPI1600000 / NS: EPI1600280 | A/Chicken/Guangdong/PO17281106/MZH/2017/5/23 | H9N2 |
| 393149 | PB2: EPI1599616 / PB1: EPI1599015 / PA: EPI1599615 / HA: EPI1599017 / NP: EPI1600277 / NA: EPI1599016 / MP: EPI1599999 / NS: EPI1600278 | A/Chicken/Guangdong/PO17281104/MZH/2017/5/23 | H9N2 |
| 393148 | PB2: EPI1599614 / PB1: EPI1599012 / PA: EPI1599613 / HA: EPI1599014 / NP: EPI1600275 / NA: EPI1599013 / MP: EPI1599998 / NS: EPI1600276 | A/Chicken/Guangdong/PO17281102/MZH/2017/5/23 | H9N2 |
| 393147 | PB2: EPI1599612 / PB1: EPI1599009 / PA: EPI1599611 / HA: EPI1599011 / NP: EPI1600273 / NA: EPI1599010 / MP: EPI1599997 / NS: EPI1600274 | A/Chicken/Guangdong/PO17281074/MZH/2017/5/23 | H9N2 |
| 393146 | PB2: EPI1599610 / PB1: EPI1599006 / PA: EPI1599609 / HA: EPI1599008 / NP: EPI1600271 / NA: EPI1599007 / MP: EPI1599996 / NS: EPI1600272 | A/Chicken/Guangdong/PO17281066/MZH/2017/5/23 | H9N2 |
| 393145 | PB2: EPI1599608 / PB1: EPI1599003 / PA: EPI1599607 / HA: EPI1599005 / NP: EPI1600269 / NA: EPI1599004 / MP: EPI1599995 / NS: EPI1600270 | A/Chicken/Guangdong/PO17281036/MZH/2017/4/26 | H9N2 |
| 393144 | PB2: EPI1599606 / PB1: EPI1599000 / PA: EPI1599605 / HA: EPI1599002 / NP: EPI1600267 / NA: EPI1599001 / MP: EPI1599994 / NS: EPI1600268 | A/Chicken/Guangdong/PO17281024/MZH/2017/4/26 | H9N2 |
| 393143 | PB2: EPI1599604 / PB1: EPI1598997 / PA: EPI1599603 / HA: EPI1598999 / NP: EPI1600265 / NA: EPI1598998 / MP: EPI1599993 / NS: EPI1600266 | A/Chicken/Guangdong/PO17281018/MZH/2017/4/26 | H9N2 |
| 393142 | PB2: EPI1599602 / PB1: EPI1598994 / PA: EPI1599601 / HA: EPI1598996 / NP: EPI1600263 / NA: EPI1598995 / MP: EPI1599992 / NS: EPI1600264 | A/Chicken/Guangdong/PO17278539/MM/2017/11/15 | H9N2 |
| 393141 | PB2: EPI1599600 / PB1: EPI1598991 / PA: EPI1599599 / HA: EPI1598993 / NP: EPI1600261 / NA: EPI1598992 / MP: EPI1599991 / NS: EPI1600262 | A/Chicken/Guangdong/PO17278537/MM/2017/11/15 | H9N2 |
| 393140 | PB2: EPI1600260 / PB1: EPI1598988 / HA: EPI1598990 / NP: EPI1600257 / NA: EPI1598989 / MP: EPI1600259 / NS: EPI1600258 | A/Chicken/Guangdong/PO17278465/MM/2017/10/20 | H9N2 |
| 393139 | PB2: EPI1600256 / PB1: EPI1598985 / HA: EPI1598987 / NP: EPI1600253 / NA: EPI1598986 / MP: EPI1600255 / NS: EPI1600254 | A/Chicken/Guangdong/PO17278461/MM/2017/10/20 | H9N2 |
| 393138 | PB2: EPI1599598 / PA: EPI1599597 / HA: EPI1598984 / NP: EPI1600251 / NA: EPI1598983 / MP: EPI1599990 / NS: EPI1600252 | A/Chicken/Guangdong/PO17278431/MM/2017/10/20 | H9N2 |
| 393137 | PB2: EPI1599596 / PB1: EPI1598980 / PA: EPI1599595 / HA: EPI1598982 / NP: EPI1600249 / NA: EPI1598981 / MP: EPI1599989 / NS: EPI1600250 | A/Chicken/Guangdong/PO17278429/MM/2017/10/20 | H9N2 |
| 393136 | PB2: EPI1599594 / PB1: EPI1598977 / PA: EPI1599593 / HA: EPI1598979 / NP: EPI1600247 / NA: EPI1598978 / MP: EPI1599988 / NS: EPI1600248 | A/Chicken/Guangdong/PO17278399/MM/2017/9/14 | H9N2 |
| 393135 | PB2: EPI1599592 / PB1: EPI1598974 / PA: EPI1599591 / HA: EPI1598976 / NP: EPI1600245 / NA: EPI1598975 / MP: EPI1599987 / NS: EPI1600246 | A/Chicken/Guangdong/PO17278397/MM/2017/9/13 | H9N2 |
| 393134 | PB2: EPI1599590 / PB1: EPI1598971 / PA: EPI1599589 / HA: EPI1598973 / NP: EPI1600243 / NA: EPI1598972 / MP: EPI1599986 / NS: EPI1600244 | A/Chicken/Guangdong/PO17278365/MM/2017/9/13 | H9N2 |
| 393133 | PB2: EPI1599588 / PB1: EPI1598968 / PA: EPI1599587 / HA: EPI1598970 / NP: EPI1600241 / NA: EPI1598969 / MP: EPI1599985 / NS: EPI1600242 | A/Chicken/Guangdong/PO17278363/MM/2017/9/13 | H9N2 |
| 393132 | PB2: EPI1599586 / PB1: EPI1598965 / PA: EPI1599585 / HA: EPI1598967 / NP: EPI1600239 / NA: EPI1598966 / MP: EPI1599984 / NS: EPI1600240 | A/Chicken/Guangdong/PO17278357/MM/2017/8/9 | H9N2 |
| 393131 | PB2: EPI1599584 / PB1: EPI1598962 / PA: EPI1599583 / HA: EPI1598964 / NP: EPI1600237 / NA: EPI1598963 / MP: EPI1599983 / NS: EPI1600238 | A/Chicken/Guangdong/PO17278355/MM/2017/8/9 | H9N2 |
| 393130 | PB2: EPI1599582 / PB1: EPI1598959 / PA: EPI1599581 / HA: EPI1598961 / NP: EPI1600235 / NA: EPI1598960 / MP: EPI1599982 / NS: EPI1600236 | A/Chicken/Guangdong/PO17278353/MM/2017/8/9 | H9N2 |
| 393129 | PB2: EPI1599580 / PB1: EPI1598956 / PA: EPI1599579 / HA: EPI1598958 / NP: EPI1600233 / NA: EPI1598957 / MP: EPI1599981 / NS: EPI1600234 | A/Chicken/Guangdong/PO17278351/MM/2017/8/9 | H9N2 |
| 393128 | PB2: EPI1599578 / PB1: EPI1598953 / PA: EPI1599577 / HA: EPI1598955 / NP: EPI1600231 / NA: EPI1598954 / MP: EPI1599980 / NS: EPI1600232 | A/Chicken/Guangdong/PO17278341/MM/2017/8/9 | H9N2 |
| 393127 | PB2: EPI1599576 / PB1: EPI1598950 / PA: EPI1599575 / HA: EPI1598952 / NP: EPI1600229 / NA: EPI1598951 / MP: EPI1599979 / NS: EPI1600230 | A/Chicken/Guangdong/PO17278325/MM/2017/8/9 | H9N2 |
| 393126 | PB2: EPI1599574 / PB1: EPI1598947 / PA: EPI1599573 / HA: EPI1598949 / NP: EPI1600227 / NA: EPI1598948 / MP: EPI1599978 / NS: EPI1600228 | A/Chicken/Guangdong/PO17278323/MM/2017/8/9 | H9N2 |
| 393125 | PB2: EPI1599572 / PB1: EPI1598944 / PA: EPI1599571 / HA: EPI1598946 / NP: EPI1600225 / NA: EPI1598945 / MP: EPI1599977 / NS: EPI1600226 | A/Chicken/Guangdong/PO17278319/MM/2017/8/9 | H9N2 |
| 393124 | PB2: EPI1599570 / PB1: EPI1598941 / PA: EPI1599569 / HA: EPI1598943 / NP: EPI1600223 / NA: EPI1598942 / MP: EPI1599976 / NS: EPI1600224 | A/Chicken/Guangdong/PO17278317/MM/2017/8/9 | H9N2 |
| 393123 | PB2: EPI1599568 / PB1: EPI1598938 / PA: EPI1599567 / HA: EPI1598940 / NP: EPI1600221 / NA: EPI1598939 / MP: EPI1599975 / NS: EPI1600222 | A/Chicken/Guangdong/PO17278315/MM/2017/8/9 | H9N2 |
| 393122 | PB2: EPI1599566 / PB1: EPI1598935 / PA: EPI1599565 / HA: EPI1598937 / NP: EPI1600219 / NA: EPI1598936 / MP: EPI1599974 / NS: EPI1600220 | A/Chicken/Guangdong/PO17278313/MM/2017/8/9 | H9N2 |
| 393121 | PB2: EPI1599564 / PB1: EPI1598932 / PA: EPI1599563 / HA: EPI1598934 / NP: EPI1600217 / NA: EPI1598933 / MP: EPI1599973 / NS: EPI1600218 | A/Chicken/Guangdong/PO17278311/MM/2017/8/9 | H9N2 |
| 393120 | PB2: EPI1599562 / PB1: EPI1598929 / PA: EPI1599561 / HA: EPI1598931 / NP: EPI1600215 / NA: EPI1598930 / MP: EPI1599972 / NS: EPI1600216 | A/Chicken/Guangdong/PO17278309/MM/2017/8/9 | H9N2 |
| 393119 | PB2: EPI1599560 / PB1: EPI1598926 / PA: EPI1599559 / HA: EPI1598928 / NP: EPI1600213 / NA: EPI1598927 / MP: EPI1599971 / NS: EPI1600214 | A/Chicken/Guangdong/PO17278308/MM/2017/8/9 | H9N2 |
| 393118 | PB2: EPI1599558 / PB1: EPI1598923 / PA: EPI1599557 / HA: EPI1598925 / NP: EPI1600211 / NA: EPI1598924 / MP: EPI1599970 / NS: EPI1600212 | A/Chicken/Guangdong/PO17278307/MM/2017/8/9 | H9N2 |
| 393117 | PB2: EPI1599556 / PB1: EPI1598920 / PA: EPI1599555 / HA: EPI1598922 / NP: EPI1600209 / NA: EPI1598921 / MP: EPI1599969 / NS: EPI1600210 | A/Chicken/Guangdong/PO17278297/MM/2017/7/19 | H9N2 |
| 393116 | PB2: EPI1599554 / PB1: EPI1598917 / PA: EPI1599553 / HA: EPI1598919 / NP: EPI1600207 / NA: EPI1598918 / MP: EPI1599968 / NS: EPI1600208 | A/Chicken/Guangdong/PO17278190/MM/2017/6/14 | H9N2 |
| 393115 | PB2: EPI1599552 / PB1: EPI1598914 / PA: EPI1599551 / HA: EPI1598916 / NP: EPI1600205 / NA: EPI1598915 / MP: EPI1599967 / NS: EPI1600206 | A/Chicken/Guangdong/PO17278189/MM/2017/6/14 | H9N2 |
| 393114 | PB2: EPI1599550 / PB1: EPI1598911 / PA: EPI1599549 / HA: EPI1598913 / NP: EPI1600203 / NA: EPI1598912 / MP: EPI1599966 / NS: EPI1600204 | A/Chicken/Guangdong/PO17278187/MM/2017/6/14 | H9N2 |
| 393113 | PB2: EPI1599548 / PB1: EPI1598908 / PA: EPI1599547 / HA: EPI1598910 / NP: EPI1600201 / NA: EPI1598909 / MP: EPI1599965 / NS: EPI1600202 | A/Chicken/Guangdong/PO17278113/MM/2017/5/04 | H9N2 |
| 393112 | PB2: EPI1599546 / PB1: EPI1598905 / PA: EPI1599545 / HA: EPI1598907 / NP: EPI1600199 / NA: EPI1598906 / MP: EPI1599964 / NS: EPI1600200 | A/Chicken/Guangdong/PO17278097/MM/2017/5/04 | H9N2 |
| 393111 | PB2: EPI1599544 / PA: EPI1599543 / HA: EPI1598904 / NP: EPI1600197 / NA: EPI1598903 / MP: EPI1599963 / NS: EPI1600198 | A/Chicken/Guangdong/PO17278091/MM/2017/5/04 | H9N2 |
| 393110 | PB2: EPI1599542 / PB1: EPI1598900 / PA: EPI1599541 / HA: EPI1598902 / NP: EPI1600195 / NA: EPI1598901 / MP: EPI1599962 / NS: EPI1600196 | A/Chicken/Guangdong/PO17278055/MM/2017/3/28 | H9N2 |
| 393109 | PB2: EPI1599540 / PB1: EPI1598897 / PA: EPI1599539 / HA: EPI1598899 / NP: EPI1600193 / NA: EPI1598898 / MP: EPI1599961 / NS: EPI1600194 | A/Chicken/Guangdong/PO17278037/MM/2017/3/28 | H9N2 |
| 393108 | PB2: EPI1599538 / PB1: EPI1598894 / PA: EPI1599537 / HA: EPI1598896 / NP: EPI1600191 / NA: EPI1598895 / MP: EPI1599960 / NS: EPI1600192 | A/Chicken/Guangdong/PO17275048/FSH/2017/4/26 | H9N2 |
| 393107 | PB2: EPI1599536 / PB1: EPI1598891 / PA: EPI1599535 / HA: EPI1598893 / NP: EPI1600189 / NA: EPI1598892 / MP: EPI1599959 / NS: EPI1600190 | A/Chicken/Guangdong/PO17275046/FSH/2017/4/26 | H9N2 |
| 393106 | PB2: EPI1599534 / PB1: EPI1598888 / PA: EPI1599533 / HA: EPI1598890 / NP: EPI1600187 / NA: EPI1598889 / MP: EPI1599958 / NS: EPI1600188 | A/Chicken/Guangdong/PO17275042/FSH/2017/4/26 | H9N2 |
| 393105 | PB2: EPI1599532 / PB1: EPI1598885 / PA: EPI1599531 / HA: EPI1598887 / NP: EPI1600185 / NA: EPI1598886 / MP: EPI1599957 / NS: EPI1600186 | A/Chicken/Guangdong/PO17275005/FSH/2017/4/26 | H9N2 |
| 393104 | PB2: EPI1599530 / PB1: EPI1598882 / PA: EPI1599529 / HA: EPI1598884 / NP: EPI1600183 / NA: EPI1598883 / MP: EPI1599956 / NS: EPI1600184 | A/Chicken/Guangdong/PO17260058/GZH/2017/4/24 | H9N2 |
| 393103 | PB2: EPI1599528 / PB1: EPI1598879 / PA: EPI1599527 / HA: EPI1598881 / NP: EPI1600181 / NA: EPI1598880 / MP: EPI1599955 / NS: EPI1600182 | A/Chicken/Guangdong/PO17260057/GZH/2017/4/24 | H9N2 |
| 393102 | PB2: EPI1599526 / PB1: EPI1598876 / PA: EPI1599525 / HA: EPI1598878 / NP: EPI1600179 / NA: EPI1598877 / MP: EPI1599954 / NS: EPI1600180 | A/Chicken/Guangdong/PO17260056/GZH/2017/3/28 | H9N2 |
| 393101 | PB2: EPI1599524 / PB1: EPI1598873 / PA: EPI1599523 / HA: EPI1598875 / NP: EPI1600177 / NA: EPI1598874 / MP: EPI1599953 / NS: EPI1600178 | A/Chicken/Guangdong/PO17260052/GZH/2017/3/28 | H9N2 |
| 393100 | PB2: EPI1599522 / PB1: EPI1598870 / PA: EPI1599521 / HA: EPI1598872 / NP: EPI1600175 / NA: EPI1598871 / MP: EPI1599952 / NS: EPI1600176 | A/Chicken/Guangdong/PO17260050/GZH/2017/3/28 | H9N2 |
| 393099 | PB2: EPI1599520 / PB1: EPI1598867 / PA: EPI1599519 / HA: EPI1598869 / NP: EPI1600173 / NA: EPI1598868 / MP: EPI1599951 / NS: EPI1600174 | A/Chicken/Guangdong/PO17260043/GZH/2017/4/24 | H9N2 |
| 393098 | PB2: EPI1599518 / PB1: EPI1598864 / PA: EPI1599517 / HA: EPI1598866 / NP: EPI1600171 / NA: EPI1598865 / MP: EPI1599950 / NS: EPI1600172 | A/Chicken/Guangdong/PO17260023/GZH/2017/4/24 | H9N2 |
| 393097 | PB2: EPI1599516 / PB1: EPI1598861 / PA: EPI1599515 / HA: EPI1598863 / NP: EPI1600169 / NA: EPI1598862 / MP: EPI1599949 / NS: EPI1600170 | A/Chicken/Guangdong/PO17260015/GZH/2017/4/24 | H9N2 |
| 393096 | PB2: EPI1599514 / PB1: EPI1598858 / PA: EPI1599513 / HA: EPI1598860 / NP: EPI1600167 / NA: EPI1598859 / MP: EPI1599948 / NS: EPI1600168 | A/Chicken/Guangdong/PO1704260031/GZH/2017/4/24 | H9N2 |
| 393095 | PB2: EPI1599512 / PB1: EPI1598855 / PA: EPI1599511 / HA: EPI1598857 / NP: EPI1600165 / NA: EPI1598856 / MP: EPI1599947 / NS: EPI1600166 | A/Chicken/Guangdong/EN17278179/MM/2017/5/12 | H9N2 |
| 393316 | PB2: EPI1599946 / PB1: EPI1599508 / PA: EPI1599945 / HA: EPI1599510 / NP: EPI1600615 / NA: EPI1599509 / MP: EPI1600164 / NS: EPI1600616 | A/Quail/Guangdong/PO17260082/GZH/2017/3/29 | H9N2 |
| 393315 | PB2: EPI1599944 / PB1: EPI1599505 / PA: EPI1599943 / HA: EPI1599507 / NP: EPI1600613 / NA: EPI1599506 / MP: EPI1600163 / NS: EPI1600614 | A/Quail/Guangdong/PO17260035/GZH/2017/4/24 | H9N2 |
| 393314 | PB2: EPI1599942 / PB1: EPI1599502 / PA: EPI1599941 / HA: EPI1599504 / NP: EPI1600611 / NA: EPI1599503 / MP: EPI1600162 / NS: EPI1600612 | A/Quail/Guangdong/PO17260029/GZH/2017/4/24 | H9N2 |
| 393313 | PB2: EPI1599940 / PB1: EPI1599499 / PA: EPI1599939 / HA: EPI1599501 / NP: EPI1600609 / NA: EPI1599500 / MP: EPI1600161 / NS: EPI1600610 | A/Quail/Guangdong/PO17260012/GZH/2017/4/24 | H9N2 |
| 393312 | PB2: EPI1599938 / PB1: EPI1599496 / PA: EPI1599937 / HA: EPI1599498 / NP: EPI1600607 / NA: EPI1599497 / MP: EPI1600160 / NS: EPI1600608 | A/Pigeon/Guangdong/PO17278287/MM/2017/7/19 | H9N2 |
| 393311 | PB2: EPI1599936 / PB1: EPI1599493 / PA: EPI1599935 / HA: EPI1599495 / NP: EPI1600605 / NA: EPI1599494 / MP: EPI1600159 / NS: EPI1600606 | A/Pigeon/Guangdong/PO17278095/MM/2017/5/04 | H9N2 |
