# Supplementary material for: Exploring utility of genomic epidemiology to trace origins of highly pathogenic influenza A/H7N9 in Guangdong
Source: Virus Evol. 2020 Dec 18;6(2):veaa097. doi: 10.1093/ve/veaa097 (PMC7758296; doi:10.1093/ve/veaa097)
Supplement: veaa097_Supplementary_Data [file veaa097_supplementary_data.zip › S table 2.docx]

Supplementary table 2 Live poultry importing from different region from January 2016 to July 2018

|  | Live poultry importing from different regions (×100,000) | | | | | |
| --- | --- | --- | --- | --- | --- | --- |
| Time | Northwest | Southwest | North | Central | East | South |
| Jan 2016 | 2.4 | 23.4 | 2.3 | 6.0 | 8.7 | 1.3 |
| Feb 2016 | 2.4 | 34.4 | 2.7 | 6.2 | 9.7 | 0.0 |
| Mar 2016 | 3.5 | 51.4 | 5.4 | 5.1 | 14.2 | 0.0 |
| Apr 2016 | 6.6 | 54.8 | 25.0 | 8.1 | 14.2 | 0.9 |
| May 2016 | 8.5 | 60.2 | 32.9 | 8.7 | 12.4 | 0.8 |
| Jun 2016 | 7.3 | 55.3 | 21.9 | 8.0 | 12.5 | 1.5 |
| Jul 2016 | 6.0 | 68.8 | 6.5 | 9.9 | 14.3 | 1.1 |
| Aug 2016 | 98.0 | 1078.4 | 124.4 | 189.7 | 529.4 | 61.3 |
| Sep 2016 | 7.3 | 48.6 | 4.5 | 9.1 | 11.4 | 3.5 |
| Oct 2016 | 4.9 | 37.1 | 3.2 | 12.2 | 12.3 | 3.6 |
| Nov 2016 | 2.4 | 28.3 | 2.7 | 5.2 | 12.9 | 4.3 |
| Dec 2016 | 0.7 | 29.8 | 2.2 | 5.2 | 43.2 | 2.4 |
| Jan 2017 | 1.7 | 36.9 | 2.2 | 8.1 | 31.7 | 2.4 |
| Feb 2017 | 2.4 | 57.9 | 3.3 | 9.8 | 20.6 | 2.5 |
| Mar 2017 | 4.1 | 54.0 | 8.0 | 14.4 | 25.5 | 2.9 |
| Apr 2017 | 4.1 | 52.5 | 15.3 | 11.3 | 32.3 | 2.3 |
| May 2017 | 5.7 | 46.4 | 20.7 | 9.6 | 15.5 | 3.1 |
| Jun 2017 | 8.8 | 45.1 | 11.5 | 8.8 | 36.4 | 2.3 |
| Jul 2017 | 5.4 | 39.6 | 4.8 | 5.9 | 21.5 | 1.9 |
| Aug 2017 | 45.1 | 540.7 | 38.9 | 78.7 | 251.5 | 28.1 |
| Sep 2017 | 2.1 | 56.6 | 3.6 | 7.9 | 17.7 | 3.0 |
| Oct 2017 | 2.6 | 41.9 | 1.9 | 7.2 | 15.3 | 2.7 |
| Nov 2017 | 3.7 | 31.0 | 1.6 | 6.2 | 20.8 | 1.9 |
| Dec 2017 | 3.7 | 22.0 | 1.4 | 7.1 | 36.2 | 2.0 |
| Jan 2018 | 3.8 | 24.1 | 1.0 | 3.3 | 23.3 | 3.3 |
| Feb 2018 | 2.3 | 29.1 | 1.0 | 5.1 | 17.7 | 2.1 |
| Mar 2018 | 3.4 | 46.8 | 1.5 | 6.1 | 20.4 | 1.7 |
| Apr 2018 | 3.9 | 56.1 | 4.1 | 5.3 | 20.1 | 2.1 |
| May 2018 | 4.6 | 57.9 | 9.5 | 6.9 | 18.8 | 2.6 |
| Jun 2018 | 7.5 | 55.1 | 5.9 | 7.6 | 20.3 | 2.3 |
| Jul 2018 | 3.9 | 64.6 | 2.9 | 7.3 | 17.5 | 2.1 |
| Total | 268.9 | 2928.8 | 372.8 | 490 | 1358.4 | 152.3 |
