# Supplementary material for: Exploring utility of genomic epidemiology to trace origins of highly pathogenic influenza A/H7N9 in Guangdong
Source: Virus Evol. 2020 Dec 18;6(2):veaa097. doi: 10.1093/ve/veaa097 (PMC7758296; doi:10.1093/ve/veaa097)
Supplement: veaa097_Supplementary_Data [file veaa097_supplementary_data.zip › S table 3.docx]

Supplementary table 3. DNA accession No of reference sequences download from GISAID and NCBI

| **H7N9** | | | | | | | |  | **H9N2** | | | | | |
| --- | --- | --- | --- | --- | --- | --- | --- | --- | --- | --- | --- | --- | --- | --- |
| **HA** | **NA** | **PB2** | **PB1** | **PA** | **NP** | **M** | **NS** |  | **PB2** | **PB1** | **PA** | **NP** | **M** | **NS** |
| EPI1090701 | EPI953104 | EPI1090706 | EPI1090830 | EPI573560 | EPI573562 | EPI1090760 | EPI979543 |  | EPI691841 | EPI692113 | EPI502468 | EPI81889 | EPI466506 | EPI81946 |
| EPI1090693 | EPI967339 | EPI573558 | EPI573559 | EPI953101 | EPI1090710 | EPI573564 | EPI654155 |  | EPI654368 | EPI89190 | EPI502469 | EPI683066 | EPI466512 | EPI81949 |
| EPI1090709 | EPI953334 | EPI952594 | EPI973692 | EPI953333 | EPI1090694 | EPI967340 | EPI773769 |  | EPI1055516 | EPI406485 | EPI502467 | EPI557803 | EPI681156 | EPI277589 |
| EPI1090832 | EPI973696 | EPI967327 | EPI973700 | EPI967330 | EPI1090758 | EPI953331 | EPI1090697 |  | EPI406486 | EPI683152 | EPI502513 | EPI597804 | EPI682981 | EPI470748 |
| EPI573561 | EPI973704 | EPI953100 | EPI967335 | EPI967336 | EPI1090766 | EPI967333 | EPI1090705 |  | EPI837993 | EPI484481 | EPI502466 | EPI10045 | EPI548388 | EPI654388 |
| EPI1090765 | EPI1054076 | EPI967328 | EPI952635 | EPI973693 | EPI967338 | EPI953105 | EPI573565 |  | EPI502504 | EPI502522 | EPI502453 | EPI81905 | EPI692273 | EPI1092096 |
| EPI1090781 | EPI1054028 | EPI973691 | EPI953051 | EPI973701 | EPI967332 | EPI973697 | EPI654020 |  | EPI502490 | EPI502563 | EPI502514 | EPI81903 | EPI406487 | EPI1093068 |
| EPI1090789 | EPI1054036 | EPI973699 | EPI967329 | EPI1054082 | EPI973695 | EPI973705 | EPI654023 |  | EPI502451 | EPI502470 | EPI502521 | EPI10039 | EPI502509 | EPI1092144 |
| EPI1090757 | EPI1054085 | EPI1054079 | EPI1054081 | EPI1054089 | EPI973703 | EPI1054077 | EPI965375 |  | EPI502607 | EPI502459 | EPI502594 | EPI81911 | EPI502612 | EPI1092162 |
| EPI447598 | EPI1054092 | EPI1054080 | EPI1054072 | EPI1053951 | EPI953103 | EPI1054053 | EPI965265 |  | EPI502621 | EPI502498 | EPI502458 | EPI81909 | EPI502474 | EPI466484 |
| EPI439486 | EPI1053962 | EPI1054071 | EPI1054088 | EPI1053959 | EPI953330 | EPI1054061 | EPI965488 |  | EPI502562 | EPI502491 | EPI502519 | EPI81867 | EPI502560 | EPI692047 |
| EPI744956 | EPI1053970 | EPI1053957 | EPI1053958 | EPI1053967 | EPI1054075 | EPI1054045 | EPI1090836 |  | EPI502460 | EPI502543 | EPI502516 | EPI81915 | EPI502464 | EPI692064 |
| EPI941039 | EPI1053954 | EPI1053949 | EPI1053950 | EPI1054073 | EPI1054084 | EPI1054069 | EPI1090761 |  | EPI502452 | EPI502505 | EPI502517 | EPI621144 | EPI502502 | EPI680994 |
| EPI509102 | EPI1054052 | EPI1053965 | EPI1053966 | EPI1054025 | EPI1054091 | EPI1053971 | EPI1090769 |  | EPI502497 | EPI502484 | EPI502518 | EPI277586 | EPI502533 | EPI680995 |
| EPI509103 | EPI1054060 | EPI1054023 | EPI1090779 | EPI1054033 | EPI1053961 | EPI1053963 | EPI1090785 |  | EPI502483 | EPI502608 | EPI502515 | EPI81883 | EPI502495 | EPI692048 |
| EPI509118 | EPI1054044 | EPI1054031 | EPI1090787 | EPI1054041 | EPI1053953 | EPI1054029 | EPI1090793 |  | EPI502556 | EPI502476 | EPI502586 | EPI81885 | EPI502554 | EPI680996 |
| EPI628080 | EPI1054068 | EPI1054039 | EPI1090755 | EPI1053985 | EPI1053969 | EPI1054086 | EPI953106 |  | EPI502528 | EPI502550 | EPI82024 | EPI81877 | EPI502526 | EPI654170 |
| EPI457725 | EPI1053988 | EPI1054055 | EPI1090763 | EPI1054001 | EPI1054027 | EPI1054005 | EPI973698 |  | EPI502542 | EPI502536 | EPI82020 | EPI383186 | EPI502547 | EPI654180 |
| EPI545788 | EPI1054012 | EPI1054047 | EPI965357 | EPI1054049 | EPI1054035 | EPI1054021 | EPI973706 |  | EPI502535 | EPI502615 | EPI82018 | EPI10043 | EPI502481 | EPI692031 |
| EPI663435 | EPI1054004 | EPI1054063 | EPI965337 | EPI1054065 | EPI1053987 | EPI1054093 | EPI967341 |  | EPI502614 | EPI502529 | EPI277584 | EPI81865 | EPI502619 | EPI557663 |
| EPI451266 | EPI1053996 | EPI1053983 | EPI965525 | EPI1054017 | EPI1053995 | EPI1054013 | EPI967334 |  | EPI502549 | EPI502557 | EPI680760 | EPI81881 | EPI502488 | EPI683108 |
| EPI447606 | EPI1053980 | EPI1053991 | EPI1090691 | EPI1054057 | EPI1054051 | EPI1053981 | EPI953332 |  | EPI457561 | EPI122535 | EPI594008 | EPI81873 | EPI502564 | EPI654365 |
| EPI447616 | EPI1054020 | EPI1054015 | EPI773763 | EPI1054009 | EPI1054003 | EPI1053997 | EPI1054030 |  | EPI572739 | EPI122491 | EPI560307 | EPI81869 | EPI502540 | EPI899152 |
| EPI464519 | EPI573563 | EPI1054007 | EPI1090707 | EPI1053975 | EPI1054019 | EPI1054037 | EPI1054038 |  | EPI572746 | EPI122575 | EPI292304 | EPI10025 | EPI654342 | EPI406488 |
| EPI509120 | EPI1090711 | EPI1053973 | EPI1090699 | EPI1053993 | EPI1054011 | EPI1053955 | EPI1054087 |  | EPI122497 | EPI122573 | EPI103273 | EPI81901 | EPI654348 | EPI177861 |
| EPI729464 | EPI1090703 | EPI1053999 | EPI692084 | EPI1090708 | EPI1054043 | EPI1053989 | EPI1054094 |  | EPI89151 | EPI118754 | EPI103307 | EPI81871 | EPI81766 | EPI127455 |
| EPI1090818 | EPI1090695 | EPI1090754 | EPI457795 | EPI1090692 | EPI1054059 | EPI1090704 | EPI1054078 |  | EPI89193 | EPI654369 | EPI140911 | EPI81875 | EPI277588 | EPI177857 |
| EPI682921 | EPI1090834 | EPI1090762 | EPI593333 | EPI1090831 | EPI1053979 | EPI1090784 | EPI1053964 |  | EPI118756 | EPI340338 | EPI487775 | EPI135022 | EPI81757 | EPI778434 |
| EPI443028 | EPI773767 | EPI1090778 | EPI593340 | EPI965516 | EPI1054067 | EPI1090768 | EPI1053956 |  | EPI899158 | EPI122427 | EPI654337 | EPI135040 | EPI81760 | EPI590293 |
| EPI443034 | EPI965405 | EPI1090786 | EPI457731 | EPI965406 | EPI1090702 | EPI1090792 | EPI1053972 |  | EPI238759 | EPI122537 | EPI1092146 | EPI103277 | EPI81748 | EPI81937 |
| EPI440095 | EPI965256 | EPI773762 | EPI656384 | EPI965541 | EPI979542 | EPI773768 | EPI1053990 |  | EPI82156 | EPI340354 | EPI1092164 | EPI81895 | EPI383188 | EPI90361 |
| EPI447609 | EPI965455 | EPI1090807 | EPI628390 | EPI773764 | EPI1090782 | EPI1090696 | EPI1054062 |  | EPI82134 | EPI340346 | EPI1093070 | EPI383194 | EPI597806 | EPI163960 |
| EPI447615 | EPI1090759 | EPI1090690 | EPI628198 | EPI1090756 | EPI1090790 | EPI1090835 | EPI1054070 |  | EPI469113 | EPI122649 | EPI1092098 | EPI81899 | EPI292308 | EPI10241 |
| EPI464525 | EPI1090767 | EPI1090698 | EPI628150 | EPI1090764 | EPI773766 | EPI965467 | EPI1053998 |  | EPI469124 | EPI122615 | EPI691927 | EPI81893 | EPI10219 | EPI81997 |
| EPI457741 | EPI1090783 | EPI965241 | EPI628270 | EPI1090700 | EPI1090833 | EPI965214 | EPI1054046 |  | EPI469114 | EPI122651 | EPI466486 | EPI89158 | EPI81799 | EPI81922 |
| EPI1090797 | EPI1090791 | EPI965171 | EPI627639 | EPI1090780 | EPI965449 | EPI965382 | EPI1054054 |  | EPI569534 | EPI10087 | EPI691911 | EPI89172 | EPI81796 | EPI81982 |
| EPI531118 | EPI447903 | EPI965232 | EPI656154 | EPI1090788 | EPI965157 | EPI1090712 | EPI1054014 |  | EPI105770 | EPI82054 | EPI691910 | EPI287741 | EPI81790 | EPI81952 |
| EPI613807 | EPI439487 | EPI691649 | EPI627703 | EPI979545 | EPI965349 | EPI692266 | EPI1054006 |  | EPI177862 | EPI82070 | EPI680729 | EPI287725 | EPI10213 | EPI103108 |
| EPI613776 | EPI503954 | EPI1090210 | EPI583269 | EPI691864 | EPI1158817 | EPI1101578 | EPI1053982 |  | EPI590286 | EPI82078 | EPI680731 | EPI287733 | EPI81787 | EPI110438 |
| EPI613828 | EPI457764 | EPI1090218 | EPI581575 | EPI1090838 | EPI691945 | EPI516601 | EPI1054022 |  | EPI127440 | EPI82074 | EPI680730 | EPI287749 | EPI89181 | EPI337049 |
| EPI613835 | EPI941041 | EPI597371 | EPI581581 | EPI691865 | EPI1090840 | EPI582328 | EPI692009 |  | EPI177850 | EPI82072 | EPI838051 | EPI163968 | EPI10217 | EPI81991 |
| EPI613790 | EPI581568 | EPI627662 | EPI490880 | EPI577888 | EPI691941 | EPI580330 | EPI532298 |  | EPI569536 | EPI277583 | EPI594005 | EPI81917 | EPI81730 | EPI81919 |
| EPI613783 | EPI440684 | EPI627638 | EPI509511 | EPI487861 | EPI470596 | EPI654128 | EPI764621 |  | EPI569538 | EPI122577 | EPI122487 | EPI81897 | EPI81754 | EPI10261 |
| EPI516488 | EPI440692 | EPI627702 | EPI1102883 | EPI516594 | EPI1101656 | EPI628531 | EPI532299 |  | EPI639592 | EPI103305 | EPI441591 | EPI10023 | EPI621146 | EPI81943 |
| EPI613821 | EPI457836 | EPI628437 | EPI1102891 | EPI656398 | EPI1018218 | EPI627188 | EPI764625 |  | EPI469107 | EPI140910 | EPI487645 | EPI81907 | EPI81802 | EPI81961 |
| EPI620174 | EPI457788 | EPI627270 | EPI1102739 | EPI627053 | EPI1101664 | EPI656237 | EPI476482 |  | EPI469076 | EPI122452 | EPI639312 | EPI81887 | EPI81769 | EPI122277 |
| EPI447625 | EPI573431 | EPI656271 | EPI1102859 | EPI656438 | EPI1101672 | EPI581876 | EPI628050 |  | EPI469109 | EPI122613 | EPI487771 | EPI110436 | EPI235294 | EPI122447 |
| EPI450850 | EPI457860 | EPI1090410 | EPI1102851 | EPI627661 | EPI979598 | EPI581854 | EPI573432 |  | EPI469127 | EPI469306 | EPI487641 | EPI103101 | EPI81733 | EPI383189 |
| EPI509087 | EPI469526 | EPI866574 | EPI1018214 | EPI656082 | EPI628105 | EPI583238 | EPI1090657 |  | EPI469100 | EPI654333 | EPI487636 | EPI337046 | EPI81805 | EPI103317 |
| EPI447619 | EPI447916 | EPI656415 | EPI1101637 | EPI628532 | EPI583222 | EPI628003 | EPI764630 |  | EPI469105 | EPI654392 | EPI487638 | EPI406489 | EPI457567 | EPI140916 |
| EPI447608 | EPI468968 | EPI1101532 | EPI1101661 | EPI1090348 | EPI655911 | EPI577962 | EPI1036705 |  | EPI82122 | EPI1093072 | EPI487627 | EPI691965 | EPI238764 | EPI90351 |
| EPI464524 | EPI453606 | EPI1090330 | EPI1101597 | EPI576693 | EPI577905 | EPI583273 | EPI872952 |  | EPI122581 | EPI1092100 | EPI487632 | EPI691995 | EPI899153 | EPI90356 |
| EPI447614 | EPI469534 | EPI1090378 | EPI656248 | EPI576883 | EPI577919 | EPI581579 | EPI1100777 |  | EPI122541 | EPI1092148 | EPI487646 | EPI122275 | EPI177860 | EPI287736 |
| EPI620078 | EPI572730 | EPI1090306 | EPI578238 | EPI509522 | EPI1100696 | EPI577907 | EPI1102847 |  | EPI122619 | EPI1092166 | EPI593999 | EPI103311 | EPI127452 | EPI287728 |
| EPI613814 | EPI569719 | EPI578203 | EPI581685 | EPI490878 | EPI1103356 | EPI577921 | EPI917069 |  | EPI122579 | EPI466488 | EPI487633 | EPI140913 | EPI590292 | EPI287744 |
| EPI636680 | EPI460769 | EPI520861 | EPI581673 | EPI576692 | EPI1090826 | EPI655977 | EPI628698 |  | EPI122617 | EPI692132 | EPI683238 | EPI466483 | EPI778433 | EPI287752 |
| EPI442707 | EPI467338 | EPI654150 | EPI581709 | EPI1090260 | EPI1101016 | EPI627660 | EPI628714 |  | EPI340339 | EPI692133 | EPI406484 | EPI691978 | EPI177856 | EPI597810 |
| EPI620085 | EPI453477 | EPI627630 | EPI597344 | EPI1090268 | EPI887797 | EPI656166 | EPI627858 |  | EPI340355 | EPI692149 | EPI654390 | EPI691979 | EPI89139 | EPI292309 |
| EPI620064 | EPI442714 | EPI627845 | EPI596939 | EPI656502 | EPI1100776 | EPI1090288 | EPI756018 |  | EPI340347 | EPI680677 | EPI691891 | EPI680836 | EPI10201 | EPI81979 |
| EPI497902 | EPI639589 | EPI627853 | EPI596889 | EPI628092 | EPI628697 | EPI596969 | EPI593473 |  | EPI122539 | EPI680676 | EPI89188 | EPI680835 | EPI81736 | EPI10263 |
| EPI447627 | EPI447857 | EPI656247 | EPI597386 | EPI655874 | EPI628601 | EPI656493 | EPI593501 |  | EPI122429 | EPI680678 | EPI89146 | EPI680837 | EPI81784 | EPI81985 |
| EPI443675 | EPI457876 | EPI578222 | EPI581691 | EPI628492 | EPI773701 | EPI627708 | EPI627435 |  | EPI122653 | EPI122282 | EPI122280 | EPI470508 | EPI81751 | EPI81988 |
| EPI615180 | EPI497859 | EPI1090338 | EPI581755 | EPI655882 | EPI627018 | EPI509875 | EPI1090537 |  | EPI235297 | EPI122493 | EPI122450 | EPI654328 | EPI81745 | EPI10257 |
| EPI497865 | EPI447843 | EPI593310 | EPI581608 | EPI577381 | EPI656331 | EPI627636 | EPI580375 |  | EPI569532 | EPI621142 | EPI383184 | EPI654387 | EPI639597 | EPI81976 |
| EPI447630 | EPI497816 | EPI593235 | EPI581730 | EPI577846 | EPI627258 | EPI627700 | EPI628754 |  | EPI1155595 | EPI82056 | EPI122571 | EPI1092143 | EPI81739 | EPI90348 |
| EPI532300 | EPI447730 | EPI593200 | EPI581601 | EPI576721 | EPI656315 | EPI1057939 | EPI703998 |  | EPI466319 | EPI82104 | EPI82010 | EPI1092161 | EPI81793 | EPI135019 |
| EPI450842 | EPI447804 | EPI593221 | EPI581737 | EPI577511 | EPI773713 | EPI1057947 | EPI593545 |  | EPI270078 | EPI82066 | EPI82006 | EPI1093067 | EPI163977 | EPI135037 |
| EPI620111 | EPI509068 | EPI593214 | EPI581743 | EPI577528 | EPI627106 | EPI1102952 | EPI566102 |  | EPI270086 | EPI383183 | EPI383192 | EPI1092095 | EPI81763 | EPI81928 |
| EPI457757 | EPI457442 | EPI593193 | EPI628526 | EPI577384 | EPI656339 | EPI1102944 | EPI566062 |  | EPI270085 | EPI597802 | EPI82008 | EPI654169 | EPI103105 | EPI81931 |
| EPI620135 | EPI447797 | EPI593228 | EPI627982 | EPI577377 | EPI1102950 | EPI1102960 | EPI627315 |  | EPI270278 | EPI82098 | EPI82030 | EPI502500 | EPI337048 | EPI103283 |
| EPI620046 | EPI590928 | EPI578103 | EPI654039 | EPI577534 | EPI730488 | EPI1090256 | EPI627347 |  | EPI246035 | EPI82092 | EPI82034 | EPI502507 | EPI81808 | EPI81973 |
| EPI441794 | EPI1090639 | EPI593298 | EPI656060 | EPI576720 | EPI628713 | EPI628643 | EPI566110 |  | EPI270277 | EPI82094 | EPI82004 | EPI502617 | EPI10199 | EPI10243 |
| EPI620071 | EPI1090679 | EPI593292 | EPI1090315 | EPI576718 | EPI1101616 | EPI1090328 | EPI566054 |  | EPI243496 | EPI10089 | EPI10157 | EPI502531 | EPI81778 | EPI81925 |
| EPI620092 | EPI1090631 | EPI578110 | EPI627679 | EPI576723 | EPI1057921 | EPI656089 | EPI627339 |  | EPI243480 | EPI82100 | EPI82036 | EPI502605 | EPI110440 | EPI81934 |
| EPI620149 | EPI1090655 | EPI593207 | EPI627942 | EPI576725 | EPI930816 | EPI580366 | EPI627499 |  | EPI243520 | EPI10067 | EPI135028 | EPI502610 | EPI81772 | EPI81970 |
| EPI443042 | EPI1090663 | EPI593304 | EPI627998 | EPI576699 | EPI971301 | EPI627204 | EPI627323 |  | EPI243536 | EPI82086 | EPI135046 | EPI502479 | EPI89153 | EPI383197 |
| EPI663440 | EPI628023 | EPI578096 | EPI656012 | EPI576713 | EPI1103960 | EPI655913 | EPI1090609 |  | EPI243488 | EPI82106 | EPI82016 | EPI502472 | EPI89167 | EPI81964 |
| EPI457733 | EPI628407 | EPI578117 | EPI627775 | EPI576698 | EPI627058 | EPI440688 | EPI566046 |  | EPI243504 | EPI159724 | EPI10175 | EPI502524 | EPI135016 | EPI81967 |
| EPI499887 | EPI583132 | EPI597165 | EPI656004 | EPI576714 | EPI656403 | EPI457752 | EPI1090601 |  | EPI243528 | EPI82096 | EPI82000 | EPI502538 | EPI135034 | EPI81955 |
| EPI620178 | EPI692630 | EPI596917 | EPI509886 | EPI577783 | EPI1102878 | EPI457792 | EPI592581 |  | EPI243512 | EPI110444 | EPI621112 | EPI502552 | EPI122270 | EPI81958 |
| EPI464555 | EPI692621 | EPI597419 | EPI578021 | EPI577463 | EPI1102886 | EPI439493 | EPI592519 |  | EPI103269 | EPI82076 | EPI82002 | EPI502520 | EPI122440 | EPI122482 |
| EPI636673 | EPI692617 | EPI597425 | EPI578014 | EPI577379 | EPI1100720 | EPI744955 | EPI592278 |  | EPI82118 | EPI103095 | EPI82050 | EPI502486 | EPI81781 | EPI764736 |
| EPI477402 | EPI692624 | EPI597444 | EPI656076 | EPI577500 | EPI1100736 | EPI941042 | EPI592248 |  | EPI82142 | EPI337042 | EPI82012 | EPI502493 | EPI383196 | EPI352574 |
| EPI620128 | EPI692625 | EPI516603 | EPI578191 | EPI576727 | EPI1057937 | EPI531772 | EPI627914 |  | EPI82138 | EPI89176 | EPI597800 | EPI502545 | EPI81775 | EPI838177 |
| EPI439507 | EPI583104 | EPI509877 | EPI597137 | EPI576728 | EPI1057945 | EPI627516 | EPI592464 |  | EPI383190 | EPI89162 | EPI82046 | EPI502462 | EPI103315 | EPI470724 |
| EPI615174 | EPI583117 | EPI627989 | EPI578091 | EPI576770 | EPI1040444 | EPI1090192 | EPI593171 |  | EPI135024 | EPI82082 | EPI82040 | EPI89144 | EPI103281 | EPI470715 |
| EPI446746 | EPI583110 | EPI656035 | EPI597123 | EPI577530 | EPI887629 | EPI531788 | EPI592285 |  | EPI135042 | EPI287730 | EPI82044 | EPI122445 | EPI81742 | EPI470725 |
| EPI613769 | EPI628255 | EPI627965 | EPI593299 | EPI576697 | EPI1102187 | EPI531780 | EPI592512 |  | EPI82114 | EPI287722 | EPI82038 | EPI89186 | EPI140915 | EPI569478 |
| EPI447624 | EPI582342 | EPI656051 | EPI593293 | EPI576782 | EPI1102958 | EPI656198 | EPI592488 |  | EPI82116 | EPI287746 | EPI10177 | EPI81891 | EPI287735 | EPI639598 |
| EPI497894 | EPI477412 | EPI627686 | EPI593305 | EPI576781 | EPI971269 | EPI628155 | EPI593150 |  | EPI10113 | EPI287738 | EPI82028 | EPI899154 | EPI287727 | EPI470744 |
| EPI443022 | EPI477451 | EPI627758 | EPI627655 | EPI577342 | EPI1022610 | EPI628203 | EPI592596 |  | EPI82112 | EPI135044 | EPI89160 | EPI238762 | EPI287743 | EPI470720 |
| EPI446962 | EPI583168 | EPI656503 | EPI627798 | EPI576726 | EPI1103976 | EPI628219 | EPI627906 |  | EPI82144 | EPI135026 | EPI89174 | EPI457565 | EPI287751 | EPI470696 |
| EPI620185 | EPI583153 | EPI592184 | EPI656100 | EPI576730 | EPI1036686 | EPI628395 | EPI627826 |  | EPI82120 | EPI10069 | EPI287731 | EPI105764 | EPI122625 | EPI470763 |
| EPI515778 | EPI583139 | EPI592191 | EPI656122 | EPI576696 | EPI1102164 | EPI531764 | EPI566094 |  | EPI621141 | EPI82090 | EPI287723 | EPI590290 | EPI340341 | EPI470689 |
| EPI515788 | EPI583097 | EPI627718 | EPI583234 | EPI577521 | EPI1101608 | EPI656206 | EPI593178 |  | EPI82110 | EPI82058 | EPI287739 | EPI177858 | EPI122547 | EPI470694 |
| EPI447623 | EPI583090 | EPI656137 | EPI581872 | EPI576766 | EPI1101624 | EPI596963 | EPI627675 |  | EPI82158 | EPI89148 | EPI287747 | EPI127448 | EPI122592 | EPI81994 |
| EPI471834 | EPI583146 | EPI597075 | EPI655988 | EPI576764 | EPI979510 | EPI627676 | EPI656396 |  | EPI292302 | EPI82062 | EPI82052 | EPI177854 | EPI122589 | EPI621147 |
| EPI663437 | EPI583124 | EPI596757 | EPI581850 | EPI576769 | EPI979590 | EPI447882 | EPI627051 |  | EPI597801 | EPI82088 | EPI10155 | EPI81913 | EPI122433 | EPI238765 |
| EPI615177 | EPI583074 | EPI597179 | EPI628006 | EPI577452 | EPI1013266 | EPI1090320 | EPI656436 |  | EPI10127 | EPI103271 | EPI82032 | EPI569705 | EPI122550 | EPI457568 |
| EPI447621 | EPI582939 | EPI596905 | EPI476699 | EPI577457 | EPI1040464 | EPI1090264 | EPI577963 |  | EPI82154 | EPI82084 | EPI163946 | EPI639595 | EPI118742 | EPI81940 |
| EPI457436 | EPI583046 | EPI597397 | EPI503497 | EPI577365 | EPI1101576 | EPI1090272 | EPI628122 |  | EPI82152 | EPI82060 | EPI82042 | EPI352573 | EPI340349 | EPI764739 |
| EPI509070 | EPI628359 | EPI597031 | EPI628518 | EPI577495 | EPI628609 | EPI592204 | EPI506229 |  | EPI10133 | EPI383191 | EPI82022 | EPI838093 | EPI122511 | EPI235295 |
| EPI457789 | EPI535146 | EPI597582 | EPI581843 | EPI577513 | EPI872951 | EPI627652 | EPI515871 |  | EPI82146 | EPI82064 | EPI110442 | EPI292307 | EPI122460 | EPI122422 |
| EPI457861 | EPI628327 | EPI578137 | EPI577924 | EPI577876 | EPI1101640 | EPI656057 | EPI655920 |  | EPI82148 | EPI569524 | EPI103097 | EPI122474 | EPI122628 | EPI122606 |
| EPI460767 | EPI628335 | EPI597065 | EPI581594 | EPI577345 | EPI887973 | EPI457800 | EPI628114 |  | EPI89165 | EPI292303 | EPI337044 | EPI470481 | EPI340334 | EPI122476 |
| EPI469524 | EPI628319 | EPI596801 | EPI498798 | EPI577531 | EPI1022658 | EPI628115 | EPI655864 |  | EPI89179 | EPI441106 | EPI577893 | EPI569703 | EPI122415 | EPI122479 |
| EPI569717 | EPI535138 | EPI597158 | EPI581587 | EPI577529 | EPI1101592 | EPI656151 | EPI655928 |  | EPI287745 | EPI469370 | EPI654349 | EPI470469 | EPI122508 | EPI118749 |
| EPI453604 | EPI535162 | EPI516595 | EPI583241 | EPI577498 | EPI1101648 | EPI447659 | EPI627739 |  | EPI287721 | EPI639593 | EPI654361 | EPI470482 | EPI569684 | EPI122639 |
| EPI572728 | EPI566123 | EPI627646 | EPI578157 | EPI577510 | EPI566077 | EPI597477 | EPI627707 |  | EPI287737 | EPI569522 | EPI590288 | EPI470504 | EPI560124 | EPI122561 |
| EPI469532 | EPI582961 | EPI627622 | EPI577937 | EPI577348 | EPI627466 | EPI637886 | EPI1090425 |  | EPI287729 | EPI469379 | EPI105766 | EPI470477 | EPI569683 | EPI340348 |
| EPI447596 | EPI582356 | EPI627206 | EPI577993 | EPI577501 | EPI627330 | EPI637879 | EPI627635 |  | EPI161623 | EPI469367 | EPI177852 | EPI470472 | EPI469961 | EPI340340 |
| EPI468967 | EPI582968 | EPI656279 | EPI577931 | EPI577502 | EPI627306 | EPI637893 | EPI628506 |  | EPI82140 | EPI469380 | EPI127444 | EPI470474 | EPI469951 | EPI122603 |
| EPI442708 | EPI583009 | EPI1090250 | EPI583262 | EPI577344 | EPI627274 | EPI663490 | EPI597248 |  | EPI10111 | EPI838008 | EPI177864 | EPI470463 | EPI469960 | EPI340333 |
| EPI442706 | EPI582907 | EPI656327 | EPI577951 | EPI577499 | EPI627282 | EPI581706 | EPI597314 |  | EPI82108 | EPI469302 | EPI82026 | EPI470468 | EPI469948 | EPI122642 |
| EPI573429 | EPI582946 | EPI580360 | EPI583220 | EPI515865 | EPI627482 | EPI656135 | EPI627763 |  | EPI82150 | EPI469375 | EPI221854 | EPI235296 | EPI469947 | EPI122564 |
| EPI457877 | EPI582985 | EPI628645 | EPI577910 | EPI499913 | EPI566037 | EPI447631 | EPI627779 |  | EPI82130 | EPI469366 | EPI441373 | EPI81879 | EPI469953 | EPI122530 |
| EPI467345 | EPI583023 | EPI1090322 | EPI583297 | EPI515857 | EPI627474 | EPI457728 | EPI656032 |  | EPI110446 | EPI469361 | EPI441374 | EPI122559 | EPI469942 | EPI122527 |
| EPI639587 | EPI582349 | EPI1090202 | EPI577896 | EPI628116 | EPI627857 | EPI490974 | EPI656048 |  | EPI103093 | EPI469372 | EPI457571 | EPI118747 | EPI838134 | EPI270091 |
| EPI442713 | EPI583016 | EPI628221 | EPI581864 | EPI655922 | EPI756015 | EPI503512 | EPI627962 |  | EPI337043 | EPI82080 | EPI899155 | EPI122472 | EPI680942 | EPI270284 |
| EPI453480 | EPI627815 | EPI628197 | EPI655916 | EPI627956 | EPI1101632 | EPI490966 | EPI656150 |  | EPI140909 | EPI899159 | EPI238761 | EPI122520 | EPI680941 | EPI270092 |
| EPI453609 | EPI627807 | EPI628149 | EPI628110 | EPI627765 | EPI926826 | EPI503515 | EPI655888 |  | EPI383182 | EPI238760 | EPI82048 | EPI122637 | EPI680943 | EPI270075 |
| EPI497814 | EPI627871 | EPI628269 | EPI1090259 | EPI627788 | EPI926834 | EPI578136 | EPI627954 |  | EPI122454 | EPI457562 | EPI82014 | EPI122470 | EPI691688 | EPI246032 |
| EPI497857 | EPI627879 | EPI1090346 | EPI1090267 | EPI628508 | EPI1101568 | EPI457680 | EPI656142 |  | EPI122495 | EPI127442 | EPI235299 | EPI122601 | EPI691676 | EPI270283 |
| EPI447602 | EPI627895 | EPI553467 | EPI577958 | EPI506240 | EPI1100784 | EPI457688 | EPI627786 |  | EPI122655 | EPI177851 | EPI122489 | EPI122420 | EPI466485 | EPI243543 |
| EPI447603 | EPI627887 | EPI627654 | EPI577917 | EPI515873 | EPI1100792 | EPI457712 | EPI1090281 |  | EPI103303 | EPI177863 | EPI122485 | EPI122635 | EPI691677 | EPI243527 |
| EPI576565 | EPI627823 | EPI627797 | EPI577903 | EPI516602 | EPI919592 | EPI583217 | EPI627747 |  | EPI122284 | EPI105768 | EPI122609 | EPI122522 | EPI469987 | EPI243495 |
| EPI497918 | EPI627903 | EPI626990 | EPI581857 | EPI577468 | EPI918737 | EPI447645 | EPI628482 |  | EPI122583 | EPI590287 | EPI122569 | EPI122557 | EPI1092163 | EPI243487 |
| EPI443635 | EPI592225 | EPI577957 | EPI583227 | EPI577496 | EPI965017 | EPI583210 | EPI627986 |  | EPI82160 | EPI82102 | EPI118752 | EPI340343 | EPI1092097 | EPI243503 |
| EPI447629 | EPI592478 | EPI628533 | EPI655980 | EPI656215 | EPI656283 | EPI692298 | EPI627699 |  | EPI82124 | EPI235298 | EPI122611 | EPI340335 | EPI1092145 | EPI243519 |
| EPI576566 | EPI592260 | EPI656439 | EPI627934 | EPI577776 | EPI1102942 | EPI447715 | EPI655992 |  | EPI10131 | EPI82068 | EPI340337 | EPI122599 | EPI1093069 | EPI243511 |
| EPI497873 | EPI592239 | EPI627054 | EPI583255 | EPI576885 | EPI1018259 | EPI457434 | EPI577922 |  | EPI82136 | EPI569520 | EPI340353 | EPI340351 | EPI122463 | EPI243535 |
| EPI442716 | EPI592549 | EPI656399 | EPI583283 | EPI509876 | EPI1101680 | EPI692289 | EPI577908 |  | EPI82132 | EPI569518 | EPI340345 | EPI691976 | EPI569682 | EPI1055469 |
| EPI576564 | EPI592556 | EPI656495 | EPI1090331 | EPI656199 | EPI1102846 | EPI509061 | EPI516600 |  | EPI82128 | EPI314010 | EPI122567 | EPI1209286 | EPI569681 | EPI441372 |
| EPI1090261 | EPI592587 | EPI581600 | EPI1090379 | EPI627645 | EPI1090486 | EPI457704 | EPI627691 |  | EPI82126 | EPI313908 | EPI122425 | EPI654364 | EPI314015 | EPI1155607 |
| EPI1090269 | EPI592167 | EPI581607 | EPI656416 | EPI627757 | EPI628745 | EPI447722 | EPI656205 |  | EPI277582 | EPI313916 | EPI122645 | EPI1092185 | EPI313913 | EPI470758 |
| EPI476697 | EPI592218 | EPI581715 | EPI1101533 | EPI627717 | EPI1102966 | EPI692296 | EPI476701 |  | EPI691750 | EPI243497 | EPI122647 | EPI470519 | EPI313921 | EPI470759 |
| EPI503499 | EPI593155 | EPI581697 | EPI596971 | EPI656136 | EPI627370 | EPI692265 | EPI503503 |  | EPI691736 | EPI243481 | EPI122533 | EPI470513 | EPI568681 | EPI470776 |
| EPI515877 | EPI592601 | EPI581742 | EPI553198 | EPI553199 | EPI1103006 | EPI692291 | EPI628106 |  | EPI691737 | EPI243537 | EPI569511 | EPI470514 | EPI246033 | EPI470751 |
| EPI506225 | EPI592620 | EPI581736 | EPI628014 | EPI1090324 | EPI1101096 | EPI692303 | EPI655912 |  | EPI680662 | EPI243489 | EPI243482 | EPI470515 | EPI270076 | EPI470752 |
| EPI628512 | EPI592253 | EPI597058 | EPI656044 | EPI627205 | EPI1101104 | EPI692305 | EPI577991 |  | EPI680660 | EPI243521 | EPI243522 | EPI470516 | EPI270089 | EPI583883 |
| EPI627960 | EPI592232 | EPI581729 | EPI578000 | EPI656278 | EPI1100496 | EPI692299 | EPI602531 |  | EPI680661 | EPI243505 | EPI243538 | EPI569567 | EPI270282 | EPI838172 |
| EPI654043 | EPI592627 | EPI581920 | EPI578007 | EPI628644 | EPI1100504 | EPI692301 | EPI602533 |  | EPI469080 | EPI243513 | EPI243490 | EPI243492 | EPI270090 | EPI470786 |
| EPI655902 | EPI592153 | EPI581754 | EPI502371 | EPI656326 | EPI1090686 | EPI692297 | EPI581913 |  | EPI654332 | EPI243529 | EPI243498 | EPI243484 | EPI270281 | EPI470783 |
| EPI628448 | EPI592290 | EPI581995 | EPI656130 | EPI580362 | EPI1102198 | EPI692302 | EPI577998 |  | EPI654391 | EPI569526 | EPI243506 | EPI243516 | EPI221856 | EPI470780 |
| EPI628104 | EPI592124 | EPI581760 | EPI656209 | EPI628012 | EPI1102398 | EPI692295 | EPI581848 |  | EPI1092099 | EPI270077 | EPI243514 | EPI243532 | EPI243542 | EPI470781 |
| EPI628504 | EPI628375 | EPI597437 | EPI583276 | EPI656042 | EPI1102032 | EPI692292 | EPI655976 |  | EPI1092147 | EPI270087 | EPI243530 | EPI243508 | EPI243526 | EPI470782 |
| EPI628464 | EPI576625 | EPI581722 | EPI597152 | EPI577343 | EPI1102040 | EPI447701 | EPI581862 |  | EPI1093071 | EPI270279 | EPI569509 | EPI243500 | EPI243502 | EPI470784 |
| EPI628480 | EPI592160 | EPI581748 | EPI507085 | EPI577497 | EPI1103014 | EPI692290 | EPI583232 |  | EPI1092165 | EPI246034 | EPI313917 | EPI270079 | EPI243486 | EPI470778 |
| EPI655958 | EPI592131 | EPI582323 | EPI627695 | EPI577884 | EPI887845 | EPI447892 | EPI577901 |  | EPI466487 | EPI270088 | EPI313909 | EPI246036 | EPI243518 | EPI470779 |
| EPI655950 | EPI592659 | EPI581877 | EPI515867 | EPI577849 | EPI888077 | EPI440696 | EPI628514 |  | EPI654173 | EPI270280 | EPI314011 | EPI270084 | EPI243494 | EPI470749 |
| EPI628456 | EPI593162 | EPI581912 | EPI499906 | EPI656230 | EPI887733 | EPI692294 | EPI506222 |  | EPI654183 | EPI692124 | EPI557735 | EPI270083 | EPI243510 | EPI654175 |
| EPI655942 | EPI592647 | EPI581891 | EPI515859 | EPI1090308 | EPI887821 | EPI591860 | EPI515863 |  | EPI691727 | EPI837997 | EPI441592 | EPI270276 | EPI243534 | EPI692061 |
| EPI515861 | EPI593647 | EPI596924 | EPI581837 | EPI627629 | EPI887853 | EPI447687 | EPI581914 |  | EPI557659 | EPI1155854 | EPI441593 | EPI270275 | EPI469983 | EPI583773 |
| EPI581692 | EPI593654 | EPI596764 | EPI581767 | EPI577389 | EPI887869 | EPI457696 | EPI490876 |  | EPI942252 | EPI596711 | EPI317180 | EPI569707 | EPI469956 | EPI583667 |
| EPI581703 | EPI1102972 | EPI596822 | EPI581781 | EPI577805 | EPI979630 | EPI692304 | EPI581599 |  | EPI683195 | EPI572927 | EPI484505 | EPI1093151 | EPI441091 | EPI838164 |
| EPI581762 | EPI627152 | EPI597025 | EPI581774 | EPI577469 | EPI1090614 | EPI457816 | EPI583239 |  | EPI221853 | EPI1155596 | EPI466358 | EPI1093157 | EPI1155606 | EPI583681 |
| EPI581717 | EPI627144 | EPI596794 | EPI581831 | EPI1090340 | EPI887757 | EPI457808 | EPI583225 |  | EPI569530 | EPI569658 | EPI466326 | EPI466323 | EPI469996 | EPI583428 |
| EPI499628 | EPI593246 | EPI578124 | EPI581915 | EPI577793 | EPI1101584 | EPI477311 | EPI577915 |  | EPI313915 | EPI569657 | EPI594093 | EPI569636 | EPI470000 | EPI583597 |
| EPI581750 | EPI592455 | EPI597270 | EPI581723 | EPI577494 | EPI1101440 | EPI477312 | EPI581855 |  | EPI313907 | EPI221859 | EPI1155852 | EPI466307 | EPI470003 | EPI838200 |
| EPI506233 | EPI593280 | EPI597350 | EPI581996 | EPI577804 | EPI979622 | EPI443670 | EPI583288 |  | EPI314009 | EPI466320 | EPI1093058 | EPI569629 | EPI470004 | EPI583555 |
| EPI515885 | EPI593190 | EPI597364 | EPI581878 | EPI577777 | EPI1090374 | EPI457824 | EPI583260 |  | EPI484480 | EPI529266 | EPI1093064 | EPI466355 | EPI529235 | EPI583625 |
| EPI654025 | EPI592448 | EPI597200 | EPI582324 | EPI577878 | EPI457790 | EPI583187 | EPI581592 |  | EPI1093059 | EPI317179 | EPI594099 | EPI317182 | EPI470005 | EPI470771 |
| EPI654032 | EPI592654 | EPI596828 | EPI581892 | EPI577851 | EPI580328 | EPI471837 | EPI583274 |  | EPI1093065 | EPI351712 | EPI594102 | EPI1093055 | EPI469988 | EPI470785 |
| EPI583270 | EPI592441 | EPI597012 | EPI581921 | EPI577787 | EPI553463 | EPI461480 | EPI656110 |  | EPI691730 | EPI466312 | EPI351704 | EPI1093061 | EPI470002 | EPI470777 |
| EPI581582 | EPI592103 | EPI597193 | EPI597013 | EPI577460 | EPI627266 | EPI509085 | EPI628002 |  | EPI572953 | EPI466360 | EPI246037 | EPI484484 | EPI470001 | EPI470762 |
| EPI581576 | EPI592138 | EPI597186 | EPI597026 | EPI577458 | EPI656267 | EPI692293 | EPI655984 |  | EPI317178 | EPI466328 | EPI270274 | EPI484492 | EPI469989 | EPI470767 |
| EPI654027 | EPI576612 | EPI597249 | EPI581749 | EPI577459 | EPI656411 | EPI457840 | EPI577956 |  | EPI837955 | EPI569655 | EPI270081 | EPI838081 | EPI470011 | EPI470750 |
| EPI499644 | EPI597483 | EPI597172 | EPI581702 | EPI502369 | EPI1101528 | EPI583194 | EPI498794 |  | EPI466359 | EPI466304 | EPI270273 | EPI572838 | EPI470020 | EPI470760 |
| EPI654015 | EPI628071 | EPI597214 | EPI581761 | EPI577391 | EPI580268 | EPI581632 | EPI509509 |  | EPI466327 | EPI466509 | EPI270082 | EPI691973 | EPI470014 | EPI470746 |
| EPI577994 | EPI627032 | EPI596771 | EPI581716 | EPI656246 | EPI566085 | EPI440680 | EPI583246 |  | EPI569666 | EPI466515 | EPI270080 | EPI578037 | EPI470015 | EPI470747 |
| EPI581873 | EPI593148 | EPI597207 | EPI597358 | EPI627621 | EPI656251 | EPI457832 | EPI577929 |  | EPI351709 | EPI441107 | EPI466310 | EPI599670 | EPI470017 | EPI466372 |
| EPI655990 | EPI654145 | EPI597315 | EPI596752 | EPI577536 | EPI580348 | EPI471836 | EPI583302 |  | EPI466311 | EPI441108 | EPI691899 | EPI600034 | EPI470018 | EPI529252 |
| EPI583235 | EPI596949 | EPI596751 | EPI596925 | EPI627844 | EPI580284 | EPI581994 | EPI627930 |  | EPI572968 | EPI441375 | EPI838039 | EPI600027 | EPI470016 | EPI573386 |
| EPI581851 | EPI596915 | EPI597357 | EPI596795 | EPI627852 | EPI580276 | EPI582321 | EPI583267 |  | EPI837967 | EPI441378 | EPI1155598 | EPI600139 | EPI470012 | EPI466284 |
| EPI628008 | EPI597395 | EPI597549 | EPI597059 | EPI577780 | EPI559410 | EPI1007139 | EPI655968 |  | EPI441109 | EPI560120 | EPI529223 | EPI680829 | EPI470013 | EPI441371 |
| EPI578158 | EPI628231 | EPI581830 | EPI597438 | EPI627685 | EPI656355 | EPI447743 | EPI655872 |  | EPI441376 | EPI837999 | EPI466318 | EPI680830 | EPI469985 | EPI441101 |
| EPI583263 | EPI627128 | EPI581766 | EPI597201 | EPI506243 | EPI580300 | EPI443045 | EPI628090 |  | EPI569668 | EPI838002 | EPI466302 | EPI680845 | EPI469986 | EPI441102 |
| EPI577904 | EPI707966 | EPI581773 | EPI597316 | EPI515881 | EPI580356 | EPI441798 | EPI655880 |  | EPI569669 | EPI416225 | EPI466507 | EPI532340 | EPI470019 | EPI838167 |
| EPI577918 | EPI707969 | EPI581836 | EPI597250 | EPI577349 | EPI580315 | EPI597499 | EPI499910 |  | EPI441113 | EPI416227 | EPI466513 | EPI680847 | EPI838130 | EPI838170 |
| EPI577959 | EPI627424 | EPI581780 | EPI596829 | EPI628524 | EPI566665 | EPI596792 | EPI515855 |  | EPI560119 | EPI529268 | EPI838053 | EPI838087 | EPI1209288 | EPI529255 |
| EPI581858 | EPI627432 | EPI596952 | EPI597365 | EPI627980 | EPI580292 | EPI597515 | EPI506237 |  | EPI529272 | EPI592016 | EPI838044 | EPI838084 | EPI1209327 | EPI466505 |
| EPI583256 | EPI592356 | EPI581905 | EPI596823 | EPI654037 | EPI628705 | EPI597334 | EPI515879 |  | EPI441377 | EPI569649 | EPI838041 | EPI600125 | EPI470010 | EPI466511 |
| EPI656116 | EPI592342 | EPI596787 | EPI597351 | EPI591249 | EPI566029 | EPI597594 | EPI578026 |  | EPI441114 | EPI351711 | EPI838046 | EPI600041 | EPI529264 | EPI560125 |
| EPI627936 | EPI592563 | EPI578169 | EPI578125 | EPI656160 | EPI580338 | EPI597087 | EPI627794 |  | EPI466287 | EPI351710 | EPI487776 | EPI599383 | EPI469999 | EPI529254 |
| EPI655982 | EPI592040 | EPI597556 | EPI597271 | EPI520860 | EPI656291 | EPI578148 | EPI656096 |  | EPI466508 | EPI569593 | EPI487773 | EPI600146 | EPI469994 | EPI416220 |
| EPI577938 | EPI592047 | EPI581627 | EPI597187 | EPI507083 | EPI1090262 | EPI597529 | EPI628098 |  | EPI466514 | EPI692130 | EPI487774 | EPI591943 | EPI469995 | EPI416221 |
| EPI628520 | EPI592363 | EPI628469 | EPI597194 | EPI656128 | EPI1090270 | EPI443037 | EPI655896 |  | EPI466439 | EPI515828 | EPI487777 | EPI569568 | EPI469997 | EPI838179 |
| EPI577932 | EPI592054 | EPI583247 | EPI597208 | EPI577493 | EPI627650 | EPI451263 | EPI628442 |  | EPI466303 | EPI515836 | EPI487798 | EPI691977 | EPI469998 | EPI764748 |
| EPI654034 | EPI592117 | EPI655963 | EPI597215 | EPI656207 | EPI656117 | EPI447694 | EPI628498 |  | EPI466279 | EPI591941 | EPI487799 | EPI515831 | EPI469992 | EPI764741 |
| EPI506218 | EPI592068 | EPI581678 | EPI596772 | EPI627693 | EPI656095 | EPI467316 | EPI627731 |  | EPI466263 | EPI469186 | EPI487802 | EPI515839 | EPI469993 | EPI351717 |
| EPI515869 | EPI592370 | EPI581947 | EPI596765 | EPI591247 | EPI627793 | EPI596850 | EPI628450 |  | EPI466351 | EPI469279 | EPI487804 | EPI470587 | EPI470007 | EPI351718 |
| EPI583228 | EPI592398 | EPI581963 | EPI597173 | EPI577883 | EPI476702 | EPI581952 | EPI655936 |  | EPI466335 | EPI469183 | EPI487800 | EPI470583 | EPI470008 | EPI416219 |
| EPI583242 | EPI592082 | EPI583309 | EPI581614 | EPI656106 | EPI503500 | EPI581981 | EPI628474 |  | EPI466343 | EPI469282 | EPI487801 | EPI470586 | EPI470006 | EPI351716 |
| EPI577925 | EPI592075 | EPI578150 | EPI581635 | EPI577795 | EPI655903 | EPI597017 | EPI655952 |  | EPI466295 | EPI469185 | EPI487806 | EPI470590 | EPI470009 | EPI466436 |
| EPI581588 | EPI592110 | EPI597082 | EPI581621 | EPI577874 | EPI627953 | EPI597030 | EPI656088 |  | EPI466399 | EPI469188 | EPI487803 | EPI470584 | EPI600127 | EPI469502 |
| EPI581595 | EPI592434 | EPI596958 | EPI581642 | EPI577873 | EPI597360 | EPI628483 | EPI628458 |  | EPI484488 | EPI469184 | EPI529224 | EPI470585 | EPI838126 | EPI764643 |
| EPI577952 | EPI592061 | EPI597494 | EPI628470 | EPI591459 | EPI593335 | EPI581741 | EPI509147 |  | EPI416229 | EPI469281 | EPI598570 | EPI470581 | EPI600148 | EPI594371 |
| EPI581844 | EPI592174 | EPI597589 | EPI583248 | EPI591441 | EPI597061 | EPI581747 | EPI655944 |  | EPI416230 | EPI600144 | EPI598439 | EPI592305 | EPI578039 | EPI515834 |
| EPI654022 | EPI592377 | EPI597329 | EPI655964 | EPI577875 | EPI597440 | EPI627780 | EPI654132 |  | EPI351708 | EPI600039 | EPI599265 | EPI583629 | EPI600043 | EPI515842 |
| EPI628112 | EPI592349 | EPI592199 | EPI581885 | EPI577872 | EPI581718 | EPI477405 | EPI656064 |  | EPI569659 | EPI599381 | EPI598438 | EPI497542 | EPI600029 | EPI469511 |
| EPI583221 | EPI592420 | EPI578143 | EPI581679 | EPI591457 | EPI581763 | EPI627748 | EPI597128 |  | EPI569598 | EPI578035 | EPI838042 | EPI1092833 | EPI600050 | EPI469503 |
| EPI655918 | EPI592335 | EPI578131 | EPI581990 | EPI591456 | EPI509132 | EPI457768 | EPI597142 |  | EPI569602 | EPI600130 | EPI598567 | EPI497501 | EPI600134 | EPI469509 |
| EPI577897 | EPI592412 | EPI597524 | EPI596959 | EPI591458 | EPI509139 | EPI656269 | EPI509140 |  | EPI466247 | EPI538911 | EPI598568 | EPI1091969 | EPI600036 | EPI469505 |
| EPI583298 | EPI592328 | EPI583316 | EPI597495 | EPI591445 | EPI581966 | EPI1090424 | EPI509131 |  | EPI466271 | EPI654314 | EPI598437 | EPI623619 | EPI599672 | EPI469506 |
| EPI577911 | EPI592089 | EPI581933 | EPI597511 | EPI591444 | EPI596941 | EPI509150 | EPI656197 |  | EPI466415 | EPI838000 | EPI598435 | EPI1092545 | EPI600141 | EPI469508 |
| EPI583284 | EPI592405 | EPI581989 | EPI578132 | EPI591442 | EPI581687 | EPI627732 | EPI509882 |  | EPI600122 | EPI600025 | EPI598569 | EPI1092803 | EPI583721 | EPI469504 |
| EPI581865 | EPI592026 | EPI581898 | EPI578151 | EPI591440 | EPI597346 | EPI457632 | EPI1101529 |  | EPI837958 | EPI600032 | EPI487852 | EPI1092821 | EPI654301 | EPI469507 |
| EPI581782 | EPI592384 | EPI583289 | EPI596965 | EPI591439 | EPI581711 | EPI1090280 | EPI1090289 |  | EPI600136 | EPI600137 | EPI487851 | EPI1092791 | EPI583666 | EPI466252 |
| EPI581775 | EPI592391 | EPI581884 | EPI597337 | EPI591446 | EPI596891 | EPI596936 | EPI831438 |  | EPI600024 | EPI600123 | EPI469446 | EPI1092077 | EPI838122 | EPI461708 |
| EPI581832 | EPI592427 | EPI581940 | EPI597330 | EPI591461 | EPI581675 | EPI597320 | EPI1090385 |  | EPI600031 | EPI469309 | EPI487849 | EPI1092461 | EPI581661 | EPI600044 |
| EPI581768 | EPI592033 | EPI581926 | EPI597083 | EPI591463 | EPI597388 | EPI597205 | EPI1090441 |  | EPI599667 | EPI469310 | EPI487848 | EPI1092521 | EPI654354 | EPI600149 |
| EPI581838 | EPI592096 | EPI581976 | EPI578144 | EPI591465 | EPI627722 | EPI578129 | EPI627938 |  | EPI578034 | EPI469238 | EPI487855 | EPI596713 | EPI583335 | EPI484487 |
| EPI581710 | EPI628263 | EPI581647 | EPI578077 | EPI627397 | EPI597245 | EPI597275 | EPI656072 |  | EPI599380 | EPI838003 | EPI487850 | EPI1093085 | EPI583484 | EPI600135 |
| EPI581615 | EPI628279 | EPI581970 | EPI596809 | EPI627924 | EPI628417 | EPI596799 | EPI656024 |  | EPI600143 | EPI469307 | EPI487791 | EPI1093187 | EPI691686 | EPI599673 |
| EPI581674 | EPI535178 | EPI581954 | EPI596953 | EPI628028 | EPI628505 | EPI596929 | EPI1090433 |  | EPI600038 | EPI469316 | EPI576716 | EPI572845 | EPI583427 | EPI600051 |
| EPI581636 | EPI1090671 | EPI597472 | EPI592207 | EPI628172 | EPI597028 | EPI596776 | EPI627970 |  | EPI600045 | EPI469317 | EPI654440 | EPI583538 | EPI583547 | EPI600030 |
| EPI581643 | EPI627552 | EPI578176 | EPI578084 | EPI628164 | EPI581732 | EPI597212 | EPI627723 |  | EPI468991 | EPI469318 | EPI487797 | EPI583887 | EPI1092133 | EPI600142 |
| EPI581622 | EPI1090487 | EPI596852 | EPI596978 | EPI628212 | EPI581610 | EPI597198 | EPI1090337 |  | EPI515827 | EPI469311 | EPI487787 | EPI583489 | EPI843872 | EPI600037 |
| EPI581602 | EPI628311 | EPI596931 | EPI581934 | EPI1090652 | EPI581644 | EPI597219 | EPI1090321 |  | EPI515835 | EPI469312 | EPI487788 | EPI583524 | EPI654261 | EPI767545 |
| EPI581609 | EPI533261 | EPI596815 | EPI581941 | EPI1090660 | EPI1090318 | EPI597177 | EPI655904 |  | EPI591940 | EPI469313 | EPI577140 | EPI600048 | EPI654422 | EPI767559 |
| EPI581686 | EPI533245 | EPI597322 | EPI581964 | EPI528358 | EPI627666 | EPI596769 | EPI656118 |  | EPI468990 | EPI469314 | EPI490999 | EPI691958 | EPI583512 | EPI568625 |
| EPI578239 | EPI533252 | EPI597263 | EPI581977 | EPI528294 | EPI628473 | EPI597191 | EPI656000 |  | EPI468986 | EPI469304 | EPI491007 | EPI838109 | EPI583596 | EPI466316 |
| EPI582325 | EPI683030 | EPI597301 | EPI581971 | EPI528374 | EPI583250 | EPI597442 | EPI627651 |  | EPI468988 | EPI469305 | EPI491023 | EPI1091753 | EPI583624 | EPI466300 |
| EPI597060 | EPI627248 | EPI597115 | EPI627735 | EPI528350 | EPI1090422 | EPI597080 | EPI628418 |  | EPI468993 | EPI469308 | EPI577123 | EPI1092647 | EPI583554 | EPI466420 |
| EPI597439 | EPI535186 | EPI597336 | EPI656092 | EPI528366 | EPI627730 | EPI597063 | EPI656008 |  | EPI468989 | EPI469325 | EPI491015 | EPI942174 | EPI838158 | EPI466268 |
| EPI597345 | EPI627576 | EPI596964 | EPI627711 | EPI552398 | EPI581745 | EPI596833 | EPI627946 |  | EPI468984 | EPI469327 | EPI491031 | EPI1093019 | EPI583882 | EPI497539 |
| EPI597014 | EPI627416 | EPI597510 | EPI656146 | EPI627181 | EPI628481 | EPI597362 | EPI627771 |  | EPI468987 | EPI469326 | EPI576919 | EPI1055512 | EPI583708 | EPI466244 |
| EPI597027 | EPI627408 | EPI578243 | EPI627751 | EPI591468 | EPI581901 | EPI596827 | EPI656056 |  | EPI837969 | EPI469328 | EPI654182 | EPI1092701 | EPI583680 | EPI469499 |
| EPI581997 | EPI627448 | EPI592206 | EPI655868 | EPI627245 | EPI581739 | EPI597254 | EPI656016 |  | EPI837960 | EPI469235 | EPI838037 | EPI1092455 | EPI466285 | EPI469498 |
| EPI581744 | EPI535170 | EPI596977 | EPI1090275 | EPI1090532 | EPI581693 | EPI597369 | EPI578019 |  | EPI837957 | EPI469237 | EPI576923 | EPI1092815 | EPI590799 | EPI466452 |
| EPI581756 | EPI628063 | EPI578083 | EPI509145 | EPI576622 | EPI581992 | EPI596756 | EPI627667 |  | EPI469082 | EPI469331 | EPI577139 | EPI1092371 | EPI466317 | EPI466498 |
| EPI581738 | EPI627560 | EPI596808 | EPI627743 | EPI1090252 | EPI655935 | EPI597355 | EPI627994 |  | EPI529273 | EPI469332 | EPI577138 | EPI1092401 | EPI441093 | EPI557664 |
| EPI581724 | EPI509670 | EPI628421 | EPI628422 | EPI498085 | EPI628113 | EPI597376 | EPI628490 |  | EPI469040 | EPI469233 | EPI844505 | EPI1091837 | EPI441092 | EPI838171 |
| EPI581731 | EPI627600 | EPI1090314 | EPI656169 | EPI532293 | EPI582362 | EPI497832 | EPI1090353 |  | EPI469052 | EPI469239 | EPI487781 | EPI1092377 | EPI838136 | EPI532326 |
| EPI581922 | EPI628303 | EPI578190 | EPI509129 | EPI457729 | EPI581704 | EPI627955 | EPI1090313 |  | EPI583330 | EPI469234 | EPI487795 | EPI1091897 | EPI441165 | EPI450835 |
| EPI581893 | EPI627839 | EPI597122 | EPI509142 | EPI443041 | EPI655895 | EPI487906 | EPI654148 |  | EPI592302 | EPI469236 | EPI487783 | EPI1091891 | EPI441164 | EPI680999 |
| EPI581879 | EPI542095 | EPI578090 | EPI627727 | EPI447747 | EPI583312 | EPI442724 | EPI578209 |  | EPI583493 | EPI529267 | EPI487784 | EPI1092869 | EPI838125 | EPI681001 |
| EPI581916 | EPI627544 | EPI597136 | EPI1090435 | EPI453476 | EPI581603 | EPI532306 | EPI627627 |  | EPI654194 | EPI491025 | EPI487779 | EPI1091951 | EPI838128 | EPI680989 |
| EPI597387 | EPI594313 | EPI628461 | EPI655908 | EPI441795 | EPI509881 | EPI515856 | EPI1090417 |  | EPI491016 | EPI583578 | EPI487780 | EPI1091939 | EPI692269 | EPI681000 |
| EPI596890 | EPI628143 | EPI655955 | EPI1090427 | EPI457745 | EPI581923 | EPI581639 | EPI730489 |  | EPI491032 | EPI469329 | EPI577133 | EPI1091957 | EPI654487 | EPI681005 |
| EPI596940 | EPI628239 | EPI656059 | EPI627671 | EPI656494 | EPI656087 | EPI581625 | EPI979591 |  | EPI654324 | EPI469324 | EPI577142 | EPI1090076 | EPI466365 | EPI681010 |
| EPI596926 | EPI528345 | EPI628477 | EPI655972 | EPI577509 | EPI596754 | EPI581618 | EPI1013267 |  | EPI837979 | EPI491001 | EPI654312 | EPI1098437 | EPI466253 | EPI680988 |
| EPI597359 | EPI528337 | EPI655939 | EPI627958 | EPI577369 | EPI597259 | EPI499909 | EPI656173 |  | EPI490992 | EPI491009 | EPI654464 | EPI1091801 | EPI654225 | EPI681006 |
| EPI597317 | EPI528377 | EPI628445 | EPI627767 | EPI577519 | EPI628121 | EPI581646 | EPI627643 |  | EPI491000 | EPI490993 | EPI1061402 | EPI1091813 | EPI583582 | EPI532327 |
| EPI596753 | EPI528297 | EPI655899 | EPI627790 | EPI627637 | EPI581725 | EPI597348 | EPI627715 |  | EPI491008 | EPI583324 | EPI1062118 | EPI1092839 | EPI491022 | EPI681008 |
| EPI578126 | EPI528361 | EPI628453 | EPI628502 | EPI627701 | EPI597139 | EPI581677 | EPI627755 |  | EPI583709 | EPI583501 | EPI654210 | EPI1055468 | EPI583505 | EPI532325 |
| EPI597272 | EPI528369 | EPI655947 | EPI628446 | EPI656152 | EPI655887 | EPI581689 | EPI656080 |  | EPI583626 | EPI583683 | EPI654296 | EPI1055473 | EPI583659 | EPI681009 |
| EPI596830 | EPI528353 | EPI628101 | EPI628102 | EPI576772 | EPI597311 | EPI597390 | EPI627659 |  | EPI654447 | EPI583697 | EPI654199 | EPI1091885 | EPI583575 | EPI764639 |
| EPI597251 | EPI628295 | EPI628501 | EPI655900 | EPI577792 | EPI581630 | EPI596893 | EPI654123 |  | EPI654408 | EPI491017 | EPI654280 | EPI1091855 | EPI583491 | EPI692049 |
| EPI597366 | EPI683025 | EPI581690 | EPI628462 | EPI577796 | EPI578016 | EPI581713 | EPI656134 |  | EPI583479 | EPI491033 | EPI577141 | EPI1091861 | EPI583526 | EPI692050 |
| EPI596824 | EPI683026 | EPI581708 | EPI655956 | EPI577877 | EPI597015 | EPI597261 | EPI656228 |  | EPI654257 | EPI583710 | EPI577135 | EPI1091849 | EPI583540 | EPI529256 |
| EPI597352 | EPI535194 | EPI578237 | EPI655940 | EPI577373 | EPI655863 | EPI578242 | EPI597460 |  | EPI583472 | EPI583466 | EPI838038 | EPI942178 | EPI583561 | EPI681027 |
| EPI597202 | EPI627536 | EPI581672 | EPI628478 | EPI577387 | EPI628497 | EPI596943 | EPI656244 |  | EPI583528 | EPI1061401 | EPI654452 | EPI1092113 | EPI583673 | EPI1155627 |
| EPI596773 | EPI594138 | EPI596888 | EPI628454 | EPI577807 | EPI581637 | EPI583252 | EPI627099 |  | EPI583382 | EPI1062117 | EPI577137 | EPI680519 | EPI583568 | EPI764750 |
| EPI596796 | EPI594299 | EPI596938 | EPI655948 | EPI591250 | EPI655919 | EPI663487 | EPI627219 |  | EPI583375 | EPI654490 | EPI577143 | EPI838240 | EPI583589 | EPI1103568 |
| EPI597195 | EPI594334 | EPI597385 | EPI515875 | EPI577532 | EPI578193 | EPI545798 | EPI597074 |  | EPI654319 | EPI654201 | EPI577128 | EPI1098439 | EPI583533 | EPI1103569 |
| EPI597209 | EPI594341 | EPI581684 | EPI628510 | EPI577382 | EPI581757 | EPI663488 | EPI593323 |  | EPI583486 | EPI654216 | EPI838074 | EPI843858 | EPI583736 | EPI1103570 |
| EPI597188 | EPI594292 | EPI597343 | EPI1090419 | EPI577789 | EPI581623 | EPI545805 | EPI593352 |  | EPI583521 | EPI654414 | EPI577083 | EPI1092137 | EPI583645 | EPI1103571 |
| EPI597216 | EPI592181 | EPI628117 | EPI628118 | EPI577488 | EPI581751 | EPI581889 | EPI593359 |  | EPI583535 | EPI469330 | EPI577067 | EPI1092785 | EPI583889 | EPI466444 |
| EPI596766 | EPI594243 | EPI655923 | EPI506223 | EPI577386 | EPI578023 | EPI583098 | EPI593199 |  | EPI583542 | EPI469320 | EPI577125 | EPI1092881 | EPI592307 | EPI654191 |
| EPI597174 | EPI583941 | EPI656019 | EPI655924 | EPI577856 | EPI628097 | EPI593322 | EPI592190 |  | EPI1092189 | EPI469321 | EPI577089 | EPI1091993 | EPI490990 | EPI466364 |
| EPI583291 | EPI583920 | EPI628125 | EPI628126 | EPI577470 | EPI597125 | EPI593351 | EPI596763 |  | EPI844490 | EPI583515 | EPI577189 | EPI1091999 | EPI490998 | EPI466388 |
| EPI581928 | EPI583907 | EPI655931 | EPI655932 | EPI577857 | EPI656172 | EPI593358 | EPI597185 |  | EPI1092123 | EPI583508 | EPI487782 | EPI1093097 | EPI491006 | EPI568628 |
| EPI581991 | EPI509673 | EPI1090418 | EPI628486 | EPI577840 | EPI656196 | EPI578181 | EPI656500 |  | EPI654427 | EPI583361 | EPI487786 | EPI1093115 | EPI654451 | EPI764724 |
| EPI581649 | EPI535202 | EPI1090426 | EPI655892 | EPI577388 | EPI628457 | EPI583147 | EPI516592 |  | EPI654297 | EPI583599 | EPI487789 | EPI1093163 | EPI654401 | EPI654185 |
| EPI581978 | EPI594250 | EPI1090434 | EPI597243 | EPI577845 | EPI655951 | EPI583173 | EPI566078 |  | EPI837989 | EPI654309 | EPI487790 | EPI1093109 | EPI654493 | EPI466218 |
| EPI597551 | EPI594236 | EPI627949 | EPI597257 | EPI577390 | EPI655927 | EPI583140 | EPI627842 |  | EPI844486 | EPI583487 | EPI538218 | EPI1093121 | EPI1061406 | EPI1103572 |
| EPI596960 | EPI583901 | EPI578013 | EPI596846 | EPI577347 | EPI581783 | EPI583154 | EPI593213 |  | EPI469033 | EPI592303 | EPI487785 | EPI1092383 | EPI1062122 | EPI594420 |
| EPI597512 | EPI592321 | EPI509885 | EPI597309 | EPI591248 | EPI581769 | EPI583091 | EPI627850 |  | EPI469034 | EPI583885 | EPI487793 | EPI1091915 | EPI583714 | EPI572877 |
| EPI596789 | EPI594271 | EPI578020 | EPI506231 | EPI577794 | EPI581776 | EPI477413 | EPI593227 |  | EPI469042 | EPI583522 | EPI487794 | EPI1093091 | EPI1092487 | EPI594435 |
| EPI597526 | EPI583895 | EPI627957 | EPI515883 | EPI577383 | EPI581833 | EPI477452 | EPI597157 |  | EPI469078 | EPI583536 | EPI487792 | EPI1093133 | EPI1092457 | EPI594442 |
| EPI597474 | EPI594306 | EPI597256 | EPI628438 | EPI577803 | EPI581839 | EPI583259 | EPI593206 |  | EPI469079 | EPI583331 | EPI487796 | EPI1093079 | EPI1092559 | EPI568629 |
| EPI655966 | EPI627392 | EPI597242 | EPI655996 | EPI577802 | EPI656055 | EPI577914 | EPI593241 |  | EPI837962 | EPI583641 | EPI577130 | EPI1093139 | EPI1092445 | EPI764760 |
| EPI581900 | EPI594173 | EPI596845 | EPI656036 | EPI577350 | EPI596848 | EPI583301 | EPI597069 |  | EPI469056 | EPI583732 | EPI577124 | EPI1091747 | EPI1092409 | EPI764726 |
| EPI581965 | EPI594257 | EPI597308 | EPI627990 | EPI577351 | EPI597318 | EPI583231 | EPI597459 |  | EPI469057 | EPI583613 | EPI577136 | EPI1092767 | EPI1092439 | EPI681030 |
| EPI628472 | EPI594285 | EPI627750 | EPI656052 | EPI457793 | EPI627674 | EPI515864 | EPI627683 |  | EPI469003 | EPI583627 | EPI1209284 | EPI1091843 | EPI1092421 | EPI681029 |
| EPI581907 | EPI594152 | EPI1090274 | EPI627966 | EPI457769 | EPI627969 | EPI656214 | EPI597588 |  | EPI469059 | EPI583557 | EPI1209323 | EPI1092029 | EPI1092331 | EPI681014 |
| EPI581958 | EPI594145 | EPI627710 | EPI655884 | EPI447733 | EPI656063 | EPI506221 | EPI593234 |  | EPI469054 | EPI583669 | EPI515829 | EPI1092035 | EPI1091875 | EPI681020 |
| EPI581949 | EPI594159 | EPI656145 | EPI628494 | EPI447807 | EPI656015 | EPI628515 | EPI597171 |  | EPI469055 | EPI583543 | EPI515837 | EPI843854 | EPI1091905 | EPI681034 |
| EPI583318 | EPI594194 | EPI509144 | EPI628094 | EPI439490 | EPI627945 | EPI577955 | EPI1090345 |  | EPI469058 | EPI583606 | EPI691924 | EPI843856 | EPI461707 | EPI764634 |
| EPI583249 | EPI628343 | EPI627734 | EPI655876 | EPI941038 | EPI656023 | EPI628107 | EPI578123 |  | EPI469060 | EPI583564 | EPI691925 | EPI1092467 | EPI843879 | EPI845712 |
| EPI581942 | EPI583927 | EPI656091 | EPI597166 | EPI447649 | EPI627937 | EPI656413 | EPI509874 |  | EPI469039 | EPI583655 | EPI577077 | EPI1093049 | EPI654366 | EPI568632 |
| EPI581935 | EPI592314 | EPI627973 | EPI578223 | EPI450849 | EPI655999 | EPI583245 | EPI597081 |  | EPI469043 | EPI583585 | EPI487805 | EPI1092197 | EPI490982 | EPI568626 |
| EPI581886 | EPI594264 | EPI627678 | EPI578138 | EPI497886 | EPI656007 | EPI581847 | EPI656165 |  | EPI469045 | EPI469319 | EPI490983 | EPI1092203 | EPI583357 | EPI764721 |
| EPI581629 | EPI594355 | EPI656168 | EPI597398 | EPI477401 | EPI656071 | EPI581598 | EPI578228 |  | EPI469046 | EPI469315 | EPI654237 | EPI1092209 | EPI583687 | EPI764749 |
| EPI581680 | EPI627584 | EPI509128 | EPI516604 | EPI663512 | EPI627770 | EPI583224 | EPI656158 |  | EPI469037 | EPI469322 | EPI577088 | EPI1092995 | EPI583652 | EPI692045 |
| EPI583311 | EPI535154 | EPI509143 | EPI592185 | EPI490967 | EPI627993 | EPI581591 | EPI592197 |  | EPI469038 | EPI469323 | EPI691892 | EPI1093007 | EPI583701 | EPI568627 |
| EPI581972 | EPI628039 | EPI628509 | EPI593208 | EPI503521 | EPI628441 | EPI577900 | EPI593316 |  | EPI469041 | EPI581657 | EPI529260 | EPI1093001 | EPI654383 | EPI764747 |
| EPI578145 | EPI594222 | EPI628485 | EPI597426 | EPI450841 | EPI597203 | EPI627931 | EPI593338 |  | EPI469035 | EPI490985 | EPI654437 | EPI1093013 | EPI654406 | EPI569574 |
| EPI596966 | EPI594229 | EPI655891 | EPI628534 | EPI457753 | EPI1090438 | EPI577990 | EPI656213 |  | EPI469036 | EPI529259 | EPI654232 | EPI1092407 | EPI491030 | EPI466380 |
| EPI597496 | EPI594180 | EPI655867 | EPI596758 | EPI447783 | EPI1090430 | EPI577997 | EPI597450 |  | EPI469047 | EPI654432 | EPI838073 | EPI1092611 | EPI491014 | EPI466412 |
| EPI597558 | EPI583913 | EPI627742 | EPI597180 | EPI457865 | EPI655991 | EPI577941 | EPI597164 |  | EPI691748 | EPI692114 | EPI577127 | EPI1092629 | EPI654242 | EPI764754 |
| EPI597591 | EPI594201 | EPI656027 | EPI578097 | EPI457841 | EPI627985 | EPI577935 | EPI578102 |  | EPI691845 | EPI583494 | EPI577134 | EPI1093031 | EPI838147 | EPI568771 |
| EPI578078 | EPI583934 | EPI627726 | EPI578118 | EPI515777 | EPI656047 | EPI583266 | EPI597461 |  | EPI538910 | EPI583571 | EPI577129 | EPI1091729 | EPI654171 | EPI764742 |
| EPI596810 | EPI594215 | EPI655907 | EPI593194 | EPI497880 | EPI627961 | EPI581861 | EPI596923 |  | EPI1209282 | EPI590918 | EPI577090 | EPI1093253 | EPI583420 | EPI568772 |
| EPI578133 | EPI627568 | EPI627670 | EPI597159 | EPI509081 | EPI656031 | EPI656174 | EPI578116 |  | EPI1209321 | EPI1086305 | EPI577091 | EPI942891 | EPI583365 | EPI635477 |
| EPI597084 | EPI594278 | EPI655971 | EPI1090339 | EPI447684 | EPI628089 | EPI577928 | EPI520858 |  | EPI583884 | EPI1093162 | EPI577087 | EPI1092239 | EPI583603 | EPI572858 |
| EPI597331 | EPI627616 | EPI627766 | EPI597076 | EPI628052 | EPI655879 | EPI581868 | EPI597457 |  | EPI583640 | EPI1209283 | EPI591267 | EPI683065 | EPI583694 | EPI568770 |
| EPI597338 | EPI594320 | EPI627789 | EPI597445 | EPI457697 | EPI628521 | EPI656429 | EPI578109 |  | EPI583731 | EPI1209322 | EPI577192 | EPI683067 | EPI583449 | EPI466404 |
| EPI597303 | EPI628047 | EPI627941 | EPI597066 | EPI515787 | EPI654035 | EPI498795 | EPI596807 |  | EPI469049 | EPI1093084 | EPI577080 | EPI1092437 | EPI583380 | EPI838165 |
| EPI597265 | EPI594166 | EPI656011 | EPI578104 | EPI497940 | EPI655871 | EPI583287 | EPI593220 |  | EPI469050 | EPI1093096 | EPI577085 | EPI1092569 | EPI583387 | EPI466237 |
| EPI597324 | EPI594208 | EPI627997 | EPI597583 | EPI487862 | EPI627977 | EPI655985 | EPI627619 |  | EPI469048 | EPI590917 | EPI577131 | EPI858159 | EPI654181 | EPI515817 |
| EPI508780 | EPI594187 | EPI627774 | EPI578111 | EPI487860 | EPI628489 | EPI628443 | EPI628522 |  | EPI469051 | EPI1092886 | EPI577145 | EPI1092749 | EPI583463 | EPI764725 |
| EPI508779 | EPI594348 | EPI656003 | EPI597032 | EPI447656 | EPI581650 | EPI628499 | EPI627978 |  | EPI583514 | EPI838017 | EPI576920 | EPI1092773 | EPI654330 | EPI515809 |
| EPI508802 | EPI594327 | EPI581641 | EPI597452 | EPI457817 | EPI581950 | EPI628475 | EPI654036 |  | EPI654233 | EPI838015 | EPI487815 | EPI1092779 | EPI654176 | EPI764758 |
| EPI1090317 | EPI535122 | EPI581620 | EPI1090307 | EPI447775 | EPI578172 | EPI628459 | EPI626987 |  | EPI583507 | EPI654207 | EPI490991 | EPI1091945 | EPI838161 | EPI764757 |
| EPI627681 | EPI627528 | EPI581613 | EPI596906 | EPI628364 | EPI583292 | EPI655969 | EPI581911 |  | EPI583360 | EPI690485 | EPI654268 | EPI1092563 | EPI843876 | EPI466468 |
| EPI596986 | EPI457636 | EPI581634 | EPI627687 | EPI1090668 | EPI597266 | EPI655873 | EPI597555 |  | EPI583598 | EPI838006 | EPI1092554 | EPI1092623 | EPI583477 | EPI506282 |
| EPI597519 | EPI457772 | EPI499905 | EPI627846 | EPI628252 | EPI581979 | EPI655865 | EPI553202 |  | EPI352576 | EPI1091848 | EPI654318 | EPI1092011 | EPI838157 | EPI594477 |
| EPI627745 | EPI447751 | EPI515858 | EPI627854 | EPI590925 | EPI596811 | EPI655953 | EPI581786 |  | EPI654308 | EPI590915 | EPI654355 | EPI1092005 | EPI1092205 | EPI596663 |
| EPI627737 | EPI940793 | EPI515866 | EPI520862 | EPI510000 | EPI578134 | EPI628099 | EPI581939 |  | EPI490984 | EPI590916 | EPI577082 | EPI1092047 | EPI1092211 | EPI465417 |
| EPI656094 | EPI940794 | EPI506230 | EPI627623 | EPI576561 | EPI578146 | EPI628451 | EPI583281 |  | EPI529258 | EPI838007 | EPI654396 | EPI1092311 | EPI583498 | EPI469512 |
| EPI656062 | EPI620180 | EPI515882 | EPI509878 | EPI447834 | EPI597332 | EPI656317 | EPI596793 |  | EPI654431 | EPI1092988 | EPI466254 | EPI1092551 | EPI1092553 | EPI467580 |
| EPI596954 | EPI636782 | EPI627981 | EPI930813 | EPI440697 | EPI578153 | EPI593337 | EPI628434 |  | EPI691828 | EPI1093030 | EPI466374 | EPI1092605 | EPI843873 | EPI553358 |
| EPI596854 | EPI620201 | EPI654038 | EPI1100789 | EPI457809 | EPI578079 | EPI593344 | EPI1090265 |  | EPI837982 | EPI1092016 | EPI466286 | EPI1091819 | EPI1092157 | EPI681012 |
| EPI597373 | EPI620066 | EPI628525 | EPI1101677 | EPI457825 | EPI596967 | EPI593303 | EPI1090273 |  | EPI583422 | EPI1092028 | EPI351706 | EPI1092323 | EPI654285 | EPI681011 |
| EPI578171 | EPI636732 | EPI628493 | EPI869671 | EPI457801 | EPI597339 | EPI509883 | EPI596858 |  | EPI583451 | EPI1092046 | EPI352577 | EPI1092515 | EPI838154 | EPI681013 |
| EPI597117 | EPI620130 | EPI655883 | EPI1101669 | EPI471856 | EPI597475 | EPI578018 | EPI597377 |  | EPI491024 | EPI1092052 | EPI484508 | EPI1092527 | EPI1092187 | EPI681015 |
| EPI596933 | EPI636828 | EPI628093 | EPI593201 | EPI447642 | EPI581943 | EPI578025 | EPI581779 |  | EPI583577 | EPI1091986 | EPI529226 | EPI1092287 | EPI654186 | EPI681016 |
| EPI578152 | EPI620169 | EPI655875 | EPI593236 | EPI509097 | EPI597304 | EPI553465 | EPI581870 |  | EPI583323 | EPI1091992 | EPI466366 | EPI1092449 | EPI654336 | EPI466460 |
| EPI596979 | EPI636724 | EPI502370 | EPI597420 | EPI509108 | EPI597592 | EPI627268 | EPI581869 |  | EPI844487 | EPI1092094 | EPI594065 | EPI736586 | EPI451173 | EPI469496 |
| EPI596817 | EPI620094 | EPI627694 | EPI578204 | EPI635611 | EPI597559 | EPI656143 | EPI581772 |  | EPI581654 | EPI1092058 | EPI461730 | EPI1092419 | EPI583617 | EPI764640 |
| EPI578178 | EPI620155 | EPI577999 | EPI627631 | EPI612867 | EPI597513 | EPI578195 | EPI581829 |  | EPI583689 | EPI1076640 | EPI466262 | EPI1092431 | EPI583631 | EPI838169 |
| EPI578085 | EPI613830 | EPI507084 | EPI654151 | EPI612866 | EPI583319 | EPI593297 | EPI597562 |  | EPI583696 | EPI1092262 | EPI466350 | EPI1091783 | EPI654377 | EPI974026 |
| EPI592208 | EPI636842 | EPI578006 | EPI596918 | EPI612861 | EPI596855 | EPI593309 | EPI581696 |  | EPI583682 | EPI838029 | EPI466278 | EPI1092335 | EPI583610 | EPI596687 |
| EPI655870 | EPI636696 | EPI583275 | EPI592192 | EPI619995 | EPI596927 | EPI597141 | EPI581784 |  | EPI583647 | EPI1091836 | EPI466390 | EPI1092341 | EPI692274 | EPI1055442 |
| EPI509151 | EPI620187 | EPI628013 | EPI593229 | EPI619992 | EPI596818 | EPI597127 | EPI581721 |  | EPI583415 | EPI1092532 | EPI594029 | EPI1092395 | EPI492274 | EPI469510 |
| EPI578192 | EPI613823 | EPI656043 | EPI593215 | EPI619997 | EPI596790 | EPI578095 | EPI581788 |  | EPI583458 | EPI1092292 | EPI594039 | EPI1092281 | EPI569551 | EPI469513 |
| EPI597124 | EPI636675 | EPI597037 | EPI593222 | EPI619996 | EPI597552 | EPI656182 | EPI597121 |  | EPI654397 | EPI1092454 | EPI487662 | EPI1092539 | EPI557777 | EPI466428 |
| EPI578092 | EPI439509 | EPI553197 | EPI593311 | EPI612863 | EPI596955 | EPI597522 | EPI578012 |  | EPI654313 | EPI1092520 | EPI487663 | EPI1093229 | EPI492281 | EPI469514 |
| EPI597138 | EPI636821 | EPI597500 | EPI516596 | EPI620013 | EPI592202 | EPI597049 | EPI578005 |  | EPI583352 | EPI1092328 | EPI577075 | EPI1092269 | EPI492288 | EPI764761 |
| EPI1090421 | EPI620073 | EPI596984 | EPI593318 | EPI612868 | EPI581936 | EPI596989 | EPI656104 |  | EPI654221 | EPI692147 | EPI1103216 | EPI1092425 | EPI568664 | EPI568764 |
| EPI600767 | EPI620144 | EPI597517 | EPI593347 | EPI612869 | EPI581681 | EPI597042 | EPI597050 |  | EPI583584 | EPI692146 | EPI1103223 | EPI1092413 | EPI1092769 | EPI568762 |
| EPI600768 | EPI620087 | EPI578183 | EPI593354 | EPI612862 | EPI581908 | EPI596975 | EPI592212 |  | EPI583563 | EPI838020 | EPI654291 | EPI1092443 | EPI468359 | EPI529253 |
| EPI627713 | EPI620062 | EPI597044 | EPI596802 | EPI620021 | EPI578179 | EPI597505 | EPI578089 |  | EPI583612 | EPI654252 | EPI594043 | EPI1092359 | EPI473796 | EPI497518 |
| EPI656148 | EPI636872 | EPI596970 | EPI656232 | EPI612865 | EPI581959 | EPI578011 | EPI596983 |  | EPI583556 | EPI654234 | EPI691908 | EPI1093127 | EPI466373 | EPI497523 |
| EPI627753 | EPI620162 | EPI597151 | EPI656217 | EPI612864 | EPI597325 | EPI578188 | EPI628010 |  | EPI583668 | EPI654448 | EPI691882 | EPI1092167 | EPI529234 | EPI470757 |
| EPI627641 | EPI613771 | EPI597563 | EPI627663 | EPI439503 | EPI597085 | EPI553201 | EPI655960 |  | EPI583605 | EPI654398 | EPI577300 | EPI1092317 | EPI568671 | EPI470769 |
| EPI627705 | EPI613785 | EPI1090258 | EPI627647 | EPI635681 | EPI597374 | EPI627692 | EPI596821 |  | EPI583654 | EPI942237 | EPI1086304 | EPI1092479 | EPI591932 | EPI470761 |
| EPI656156 | EPI636858 | EPI1090266 | EPI627759 | EPI581566 | EPI597527 | EPI655889 | EPI581904 |  | EPI654489 | EPI1093054 | EPI572899 | EPI1092473 | EPI838132 | EPI470770 |
| EPI627784 | EPI636775 | EPI476698 | EPI627719 | EPI457881 | EPI581973 | EPI655897 | EPI583253 |  | EPI654200 | EPI1092196 | EPI1103547 | EPI1092503 | EPI654360 | EPI451174 |
| EPI600769 | EPI636851 | EPI503496 | EPI656138 | EPI446960 | EPI596961 | EPI628523 | EPI581700 |  | EPI654227 | EPI1092130 | EPI1103546 | EPI1092509 | EPI594476 | EPI654230 |
| EPI1090277 | EPI636807 | EPI628517 | EPI457859 | EPI576835 | EPI581929 | EPI583280 | EPI581890 |  | EPI654413 | EPI1092268 | EPI1103544 | EPI1092245 | EPI596662 | EPI692062 |
| EPI627769 | EPI636747 | EPI578156 | EPI509126 | EPI576867 | EPI597497 | EPI628011 | EPI582316 |  | EPI1062116 | EPI1092112 | EPI1103545 | EPI1092293 | EPI416211 | EPI491013 |
| EPI627792 | EPI636946 | EPI583261 | EPI457787 | EPI576866 | EPI895534 | EPI597568 | EPI582319 |  | EPI1061400 | EPI1092106 | EPI577151 | EPI1091909 | EPI470051 | EPI491029 |
| EPI597244 | EPI636739 | EPI583282 | EPI460765 | EPI576868 | EPI895530 | EPI597587 | EPI582317 |  | EPI469081 | EPI654435 | EPI1155613 | EPI1076637 | EPI470056 | EPI1209289 |
| EPI597310 | EPI613809 | EPI628109 | EPI450840 | EPI576870 | EPI895535 | EPI656073 | EPI581653 |  | EPI469061 | EPI654442 | EPI577338 | EPI1092485 | EPI596708 | EPI1209328 |
| EPI596847 | EPI636916 | EPI583219 | EPI532297 | EPI576836 | EPI592209 | EPI578004 | EPI581953 |  | EPI469053 | EPI654419 | EPI1155630 | EPI1092557 | EPI680979 | EPI470754 |
| EPI597258 | EPI620120 | EPI655915 | EPI628366 | EPI477409 | EPI578086 | EPI628491 | EPI596976 |  | EPI469044 | EPI654212 | EPI680768 | EPI1067643 | EPI466301 | EPI470755 |
| EPI578015 | EPI636796 | EPI490879 | EPI447671 | EPI477448 | EPI596980 | EPI656105 | EPI597506 |  | EPI469062 | EPI654239 | EPI681084 | EPI578199 | EPI529237 | EPI470753 |
| EPI578022 | EPI636761 | EPI509510 | EPI497834 | EPI447740 | EPI895538 | EPI628091 | EPI581932 |  | EPI583570 | EPI838011 | EPI681083 | EPI838113 | EPI466437 | EPI470756 |
| EPI578185 | EPI620176 | EPI583268 | EPI477400 | EPI460774 | EPI887829 | EPI627979 | EPI583295 |  | EPI583465 | EPI466288 | EPI529222 | EPI1091873 | EPI351714 | EPI470773 |
| EPI597565 | EPI636835 | EPI581574 | EPI447784 | EPI447754 | EPI930808 | EPI656127 | EPI628466 |  | EPI654178 | EPI572934 | EPI528390 | EPI1091903 | EPI692281 | EPI470774 |
| EPI597039 | EPI620034 | EPI581580 | EPI457699 | EPI447800 | EPI1101552 | EPI628435 | EPI581969 |  | EPI583500 | EPI466376 | EPI528382 | EPI1091921 | EPI352572 | EPI470772 |
| EPI597502 | EPI636879 | EPI581849 | EPI515794 | EPI446452 | EPI1022642 | EPI502368 | EPI578182 |  | EPI583661 | EPI484489 | EPI548408 | EPI843860 | EPI764801 | EPI470775 |
| EPI596972 | EPI636932 | EPI628005 | EPI457667 | EPI457649 | EPI1040480 | EPI597156 | EPI581962 |  | EPI837954 | EPI466392 | EPI528398 | EPI1092689 | EPI416212 | EPI683110 |
| EPI597046 | EPI620027 | EPI583233 | EPI692081 | EPI440689 | EPI884215 | EPI507082 | EPI656040 |  | EPI583444 | EPI466248 | EPI681082 | EPI1092257 | EPI529236 | EPI529265 |
| EPI628016 | EPI620106 | EPI655987 | EPI692085 | EPI457777 | EPI1018113 | EPI655905 | EPI578175 |  | EPI583703 | EPI466272 | EPI681074 | EPI1092155 | EPI351713 | EPI692032 |
| EPI656046 | EPI486493 | EPI577992 | EPI583190 | EPI461484 | EPI866570 | EPI627963 | EPI597043 |  | EPI583877 | EPI466256 | EPI681081 | EPI843859 | EPI466333 | EPI654429 |
| EPI583277 | EPI636924 | EPI583254 | EPI477320 | EPI447705 | EPI887837 | EPI656025 | EPI583315 |  | EPI583549 | EPI416226 | EPI681085 | EPI843861 | EPI466341 | EPI583611 |
| EPI578008 | EPI620080 | EPI627933 | EPI447692 | EPI576872 | EPI596934 | EPI656033 | EPI578189 |  | EPI583619 | EPI466424 | EPI681090 | EPI1092221 | EPI466293 | EPI1093044 |
| EPI578001 | EPI620055 | EPI655979 | EPI583213 | EPI487859 | EPI627785 | EPI627987 | EPI581633 |  | EPI583591 | EPI466416 | EPI681092 | EPI1092215 | EPI466397 | EPI1093056 |
| EPI597153 | EPI620113 | EPI577930 | EPI692083 | EPI443666 | EPI628449 | EPI656041 | EPI581946 |  | EPI837990 | EPI466456 | EPI681094 | EPI1092617 | EPI466245 | EPI1093062 |
| EPI627697 | EPI636754 | EPI577936 | EPI447720 | EPI477325 | EPI627762 | EPI1090336 | EPI596937 |  | EPI583675 | EPI465411 | EPI654412 | EPI1092593 | EPI466413 | EPI652955 |
| EPI656132 | EPI613837 | EPI581856 | EPI457435 | EPI447896 | EPI655943 | EPI1090384 | EPI583322 |  | EPI654275 | EPI466472 | EPI654226 | EPI1092575 | EPI466499 | EPI497500 |
| EPI656211 | EPI636689 | EPI577902 | EPI692070 | EPI447698 | EPI578093 | EPI656081 | EPI596990 |  | EPI654292 | EPI572905 | EPI654250 | EPI1092251 | EPI466325 | EPI1093098 |
| EPI656179 | EPI620041 | EPI577916 | EPI692082 | EPI471853 | EPI582326 | EPI655921 | EPI597478 |  | EPI654251 | EPI572909 | EPI654215 | EPI1093217 | EPI764798 | EPI1086299 |
| EPI509135 | EPI636865 | EPI577923 | EPI692088 | EPI457833 | EPI581880 | EPI1101530 | EPI597523 |  | EPI654303 | EPI469189 | EPI654446 | EPI594460 | EPI466277 | EPI590950 |
| EPI656078 | EPI620137 | EPI583240 | EPI692090 | EPI576876 | EPI581894 | EPI627684 | EPI507081 |  | EPI837953 | EPI592002 | EPI654262 | EPI594467 | EPI466261 | EPI838175 |
| EPI656124 | EPI620099 | EPI498797 | EPI692080 | EPI447691 | EPI581917 | EPI627644 | EPI656126 |  | EPI654459 | EPI553352 | EPI654494 | EPI557814 | EPI466349 | EPI590947 |
| EPI627800 | EPI636909 | EPI581842 | EPI692078 | EPI477329 | EPI627738 | EPI627716 | EPI502367 |  | EPI837986 | EPI469190 | EPI1092134 | EPI594446 | EPI654219 | EPI590948 |
| EPI627657 | EPI636682 | EPI577909 | EPI692086 | EPI447726 | EPI509148 | EPI656245 | EPI578082 |  | EPI1093125 | EPI469187 | EPI1209315 | EPI838103 | EPI590944 | EPI838176 |
| EPI656102 | EPI620048 | EPI581586 | EPI692077 | EPI509057 | EPI626986 | EPI592189 | EPI596814 |  | EPI1093155 | EPI469191 | EPI1209331 | EPI1055744 | EPI838155 | EPI590949 |
| EPI1090429 | EPI636768 | EPI577950 | EPI457707 | EPI457705 | EPI499908 | EPI656229 | EPI597509 |  | EPI1093167 | EPI466384 | EPI1098450 | EPI838092 | EPI1092277 | EPI1093230 |
| EPI656038 | EPI613816 | EPI581863 | EPI583206 | EPI627605 | EPI515854 | EPI1090352 | EPI597595 |  | EPI1093113 | EPI466408 | EPI654402 | EPI590937 | EPI1093057 | EPI1092180 |
| EPI655894 | EPI613792 | EPI583296 | EPI447706 | EPI576558 | EPI655967 | EPI593198 | EPI597307 |  | EPI654453 | EPI466240 | EPI654482 | EPI1092713 | EPI1093063 | EPI838195 |
| EPI627729 | EPI516487 | EPI581871 | EPI467775 | EPI576559 | EPI506234 | EPI593205 | EPI764633 |  | EPI466367 | EPI572929 | EPI654417 | EPI1092125 | EPI838153 | EPI1092090 |
| EPI655998 | EPI531120 | EPI577895 | EPI457843 | EPI576560 | EPI515878 | EPI593240 | EPI597335 |  | EPI466375 | EPI572921 | EPI654426 | EPI654461 | EPI1092181 | EPI1091982 |
| EPI628488 | EPI613778 | EPI581593 | EPI471847 | EPI576722 | EPI506226 | EPI593226 | EPI597328 |  | EPI529274 | EPI635475 | EPI654220 | EPI654271 | EPI1093231 | EPI1092042 |
| EPI627992 | EPI636939 | EPI497822 | EPI447650 | EPI576729 | EPI515870 | EPI593219 | EPI597530 |  | EPI844489 | EPI572920 | EPI466438 | EPI838091 | EPI568789 | EPI1091988 |
| EPI509136 | EPI636789 | EPI1055429 | EPI457683 | EPI576724 | EPI628465 | EPI593233 | EPI597516 |  | EPI680651 | EPI572923 | EPI577081 | EPI1055598 | EPI572789 | EPI1092060 |
| EPI655974 | EPI620194 | EPI532309 | EPI457691 | EPI576731 | EPI655959 | EPI578101 | EPI597269 |  | EPI680643 | EPI572917 | EPI351705 | EPI1055814 | EPI568794 | EPI1092012 |
| EPI627673 | EPI636814 | EPI447911 | EPI692079 | EPI576733 | EPI627706 | EPI578227 | EPI597342 |  | EPI466447 | EPI466336 | EPI416222 | EPI1055798 | EPI569549 | EPI1092024 |
| EPI628128 | EPI636710 | EPI730492 | EPI692089 | EPI576758 | EPI656141 | EPI520859 | EPI597569 |  | EPI466501 | EPI466296 | EPI572874 | EPI1055790 | EPI760574 | EPI1093026 |
| EPI655934 | EPI636703 | EPI457850 | EPI477319 | EPI576757 | EPI627746 | EPI597449 | EPI592205 |  | EPI466471 | EPI466344 | EPI680769 | EPI1055806 | EPI568737 | EPI654278 |
| EPI628440 | EPI636717 | EPI509127 | EPI447897 | EPI576732 | EPI656149 | EPI593212 | EPI596944 |  | EPI466431 | EPI466400 | EPI680739 | EPI1092017 | EPI764791 | EPI858160 |
| EPI628120 | EPI637558 | EPI457858 | EPI457715 | EPI576756 | EPI627634 | EPI656341 | EPI581699 |  | EPI466423 | EPI469274 | EPI532332 | EPI1092731 | EPI469990 | EPI1092252 |
| EPI655926 | EPI638034 | EPI457786 | EPI443665 | EPI576719 | EPI627778 | EPI597163 | EPI581701 |  | EPI451168 | EPI680682 | EPI680741 | EPI583848 | EPI469991 | EPI961392 |
| EPI627968 | EPI638041 | EPI460764 | EPI509080 | EPI576780 | EPI627698 | EPI578142 | EPI581626 |  | EPI583859 | EPI466464 | EPI577311 | EPI1055449 | EPI466269 | EPI1092750 |
| EPI656054 | EPI636895 | EPI568750 | EPI591856 | EPI576891 | EPI1090278 | EPI597430 | EPI581787 |  | EPI583789 | EPI974020 | EPI654187 | EPI583820 | EPI466421 | EPI961372 |
| EPI655910 | EPI637789 | EPI627869 | EPI692087 | EPI576776 | EPI581616 | EPI596806 | EPI581707 |  | EPI583796 | EPI1055446 | EPI466213 | EPI583827 | EPI466381 | EPI736589 |
| EPI1090437 | EPI637782 | EPI583100 | EPI583183 | EPI576700 | EPI596774 | EPI578108 | EPI581655 |  | EPI466235 | EPI838001 | EPI577313 | EPI583813 | EPI466238 | EPI838182 |
| EPI627777 | EPI638055 | EPI583113 | EPI692069 | EPI576765 | EPI578127 | EPI1090312 | EPI581640 |  | EPI466383 | EPI596682 | EPI577314 | EPI583834 | EPI466405 | EPI838185 |
| EPI627944 | EPI637051 | EPI583120 | EPI692076 | EPI628468 | EPI597273 | EPI656065 | EPI597349 |  | EPI466407 | EPI591962 | EPI572737 | EPI590938 | EPI654279 | EPI578202 |
| EPI656006 | EPI637775 | EPI1090178 | EPI692075 | EPI576881 | EPI597353 | EPI597402 | EPI581714 |  | EPI572954 | EPI764806 | EPI572744 | EPI654277 | EPI470053 | EPI654203 |
| EPI628000 | EPI637726 | EPI1090634 | EPI509056 | EPI655962 | EPI596797 | EPI597184 | EPI597405 |  | EPI572961 | EPI466432 | EPI487838 | EPI1093241 | EPI1092679 | EPI838197 |
| EPI656014 | EPI637915 | EPI628773 | EPI447727 | EPI576767 | EPI597217 | EPI597073 | EPI581728 |  | EPI572973 | EPI515802 | EPI487837 | EPI838111 | EPI1093075 | EPI690482 |
| EPI627952 | EPI637516 | EPI628821 | EPI1055430 | EPI577526 | EPI596831 | EPI597036 | EPI581656 |  | EPI572958 | EPI467582 | EPI487841 | EPI1092179 | EPI568612 | EPI961414 |
| EPI656022 | EPI637037 | EPI593643 | EPI566122 | EPI577527 | EPI597196 | EPI627628 | EPI581785 |  | EPI635478 | EPI594366 | EPI838048 | EPI1092233 | EPI1092037 | EPI1091922 |
| EPI628424 | EPI636953 | EPI593650 | EPI1090203 | EPI577880 | EPI597252 | EPI656293 | EPI581606 |  | EPI572959 | EPI572939 | EPI577079 | EPI838101 | EPI1092031 | EPI1091916 |
| EPI656030 | EPI637964 | EPI1090650 | EPI971306 | EPI577517 | EPI597210 | EPI578115 | EPI581735 |  | EPI572946 | EPI594444 | EPI577084 | EPI838099 | EPI1092085 | EPI1091802 |
| EPI627976 | EPI637922 | EPI1090658 | EPI627335 | EPI577525 | EPI597189 | EPI578208 | EPI581619 |  | EPI572942 | EPI594465 | EPI654302 | EPI654202 | EPI942102 | EPI1091814 |
| EPI656070 | EPI637971 | EPI628357 | EPI1101021 | EPI577512 | EPI597175 | EPI597456 | EPI581957 |  | EPI467583 | EPI515811 | EPI654274 | EPI690483 | EPI1076643 | EPI1091850 |
| EPI656418 | EPI638069 | EPI1090626 | EPI1102571 | EPI577879 | EPI597367 | EPI596910 | EPI581897 |  | EPI837961 | EPI572935 | EPI654243 | EPI497515 | EPI1091767 | EPI1091856 |
| EPI1101535 | EPI637072 | EPI1090674 | EPI1102587 | EPI576773 | EPI596767 | EPI578122 | EPI581956 |  | EPI468980 | EPI680712 | EPI838063 | EPI497526 | EPI1091911 | EPI1091790 |
| EPI1090333 | EPI637200 | EPI729448 | EPI1100725 | EPI576715 | EPI596825 | EPI627756 | EPI597262 |  | EPI468979 | EPI506283 | EPI654323 | EPI599440 | EPI1092793 | EPI1091862 |
| EPI654138 | EPI637509 | EPI531800 | EPI1100741 | EPI576771 | EPI597118 | EPI627620 | EPI597068 |  | EPI466455 | EPI469280 | EPI577078 | EPI530487 | EPI1092835 | EPI1091820 |
| EPI1090381 | EPI637649 | EPI729456 | EPI1100557 | EPI576887 | EPI581887 | EPI627851 | EPI597064 |  | EPI468978 | EPI680685 | EPI576924 | EPI599390 | EPI1092841 | EPI843887 |
| EPI627984 | EPI637285 | EPI628653 | EPI1100637 | EPI576768 | EPI581852 | EPI656453 | EPI597443 |  | EPI528391 | EPI680684 | EPI577126 | EPI599412 | EPI1092805 | EPI843886 |
| EPI628528 | EPI637399 | EPI593332 | EPI1100629 | EPI576774 | EPI581874 | EPI597424 | EPI578130 |  | EPI572974 | EPI680713 | EPI576927 | EPI599433 | EPI1091935 | EPI843888 |
| EPI655878 | EPI638104 | EPI593339 | EPI1100677 | EPI576778 | EPI628001 | EPI593315 | EPI597206 |  | EPI837972 | EPI592009 | EPI577086 | EPI599503 | EPI1092823 | EPI1092258 |
| EPI628496 | EPI637887 | EPI580376 | EPI628614 | EPI576777 | EPI583236 | EPI627843 | EPI597321 |  | EPI654263 | EPI466448 | EPI576926 | EPI599461 | EPI1092625 | EPI1091874 |
| EPI628096 | EPI638090 | EPI580384 | EPI593404 | EPI576775 | EPI655983 | EPI1090344 | EPI596770 |  | EPI1092135 | EPI466502 | EPI654407 | EPI599517 | EPI1092013 | EPI1091904 |
| EPI655886 | EPI637100 | EPI627022 | EPI1102194 | EPI576918 | EPI583285 | EPI596762 | EPI597178 |  | EPI1092141 | EPI469271 | EPI577074 | EPI599524 | EPI1092463 | EPI1093050 |
| EPI596759 | EPI637385 | EPI656335 | EPI1102147 | EPI656058 | EPI628513 | EPI656111 | EPI597213 |  | EPI654483 | EPI680691 | EPI654256 | EPI599531 | EPI1092649 | EPI767098 |
| EPI597181 | EPI637537 | EPI628069 | EPI1103981 | EPI1090316 | EPI506219 | EPI597170 | EPI597220 |  | EPI1098460 | EPI532331 | EPI838070 | EPI599447 | EPI1092523 | EPI692044 |
| EPI656219 | EPI637719 | EPI592228 | EPI1036683 | EPI591462 | EPI515862 | EPI656159 | EPI597199 |  | EPI1209329 | EPI680692 | EPI844507 | EPI599419 | EPI1092169 | EPI623622 |
| EPI627721 | EPI637371 | EPI627813 | EPI1102170 | EPI577491 | EPI577939 | EPI596922 | EPI596777 |  | EPI1209313 | EPI468356 | EPI654458 | EPI600097 | EPI1092613 | EPI961416 |
| EPI656140 | EPI637357 | EPI627877 | EPI1090806 | EPI577858 | EPI655975 | EPI516593 | EPI597192 |  | EPI515810 | EPI352575 | EPI1093112 | EPI599404 | EPI1092319 | EPI838192 |
| EPI627665 | EPI638006 | EPI628373 | EPI1100413 | EPI577489 | EPI627929 | EPI592196 | EPI582329 |  | EPI466479 | EPI466440 | EPI1093124 | EPI600076 | EPI1067638 | EPI838188 |
| EPI1179232 | EPI637392 | EPI627550 | EPI971330 | EPI577847 | EPI577933 | EPI451019 | EPI596851 |  | EPI572978 | EPI466368 | EPI492303 | EPI600104 | EPI1092631 | EPI1091730 |
| EPI597167 | EPI637307 | EPI628277 | EPI1100421 | EPI656144 | EPI583257 | EPI576603 | EPI582322 |  | EPI515801 | EPI529269 | EPI572902 | EPI600083 | EPI1092061 | EPI838187 |
| EPI596919 | EPI637233 | EPI628285 | EPI1100485 | EPI627709 | EPI577995 | EPI506236 | EPI597255 |  | EPI468994 | EPI692105 | EPI572884 | EPI599817 | EPI1092091 | EPI599485 |
| EPI578139 | EPI637901 | EPI1090466 | EPI971514 | EPI627733 | EPI583264 | EPI515880 | EPI596930 |  | EPI680634 | EPI600074 | EPI641378 | EPI599510 | EPI682980 | EPI599534 |
| EPI596803 | EPI637740 | EPI596911 | EPI1100541 | EPI656090 | EPI578159 | EPI457880 | EPI597363 |  | EPI680649 | EPI572916 | EPI845707 | EPI600055 | EPI680977 | EPI530488 |
| EPI627649 | EPI637023 | EPI597451 | EPI971522 | EPI627485 | EPI581859 | EPI655961 | EPI581883 |  | EPI680641 | EPI767543 | EPI560311 | EPI599489 | EPI942118 | EPI600107 |
| EPI593230 | EPI637698 | EPI597479 | EPI627375 | EPI627477 | EPI656109 | EPI581896 | EPI597356 |  | EPI680642 | EPI767557 | EPI577303 | EPI599496 | EPI682979 | EPI599415 |
| EPI593195 | EPI637866 | EPI628229 | EPI1103965 | EPI627333 | EPI581845 | EPI446961 | EPI597370 |  | EPI680650 | EPI451169 | EPI569452 | EPI599426 | EPI691670 | EPI599681 |
| EPI593237 | EPI637321 | EPI627422 | EPI887634 | EPI566040 | EPI581596 | EPI656277 | EPI596800 |  | EPI680657 | EPI654339 | EPI569455 | EPI600069 | EPI583470 | EPI600058 |
| EPI593216 | EPI637079 | EPI627430 | EPI919597 | EPI509146 | EPI581589 | EPI627995 | EPI597276 |  | EPI680658 | EPI654345 | EPI572887 | EPI599552 | EPI583519 | EPI599464 |
| EPI593223 | EPI637349 | EPI596945 | EPI918732 | EPI628452 | EPI498793 | EPI627939 | EPI457671 |  | EPI680656 | EPI583797 | EPI572889 | EPI600062 | EPI583328 | EPI600093 |
| EPI593202 | EPI637476 | EPI597391 | EPI965014 | EPI655946 | EPI577953 | EPI656325 | EPI447906 |  | EPI680659 | EPI583790 | EPI572900 | EPI599538 | EPI654372 | EPI600065 |
| EPI592193 | EPI637164 | EPI592437 | EPI1101573 | EPI628476 | EPI577926 | EPI627772 | EPI447732 |  | EPI591985 | EPI583860 | EPI680773 | EPI599475 | EPI654236 | EPI599555 |
| EPI707946 | EPI636960 | EPI592099 | EPI594303 | EPI655938 | EPI583243 | EPI581961 | EPI447806 |  | EPI592008 | EPI583480 | EPI492306 | EPI599482 | EPI838140 | EPI600100 |
| EPI708131 | EPI637950 | EPI592651 | EPI833702 | EPI628444 | EPI577898 | EPI627740 | EPI941043 |  | EPI468992 | EPI654179 | EPI594036 | EPI599736 | EPI654457 | EPI600114 |
| EPI708036 | EPI637434 | EPI592643 | EPI1101453 | EPI628500 | EPI577912 | EPI637494 | EPI615176 |  | EPI468996 | EPI583452 | EPI691912 | EPI599454 | EPI843875 | EPI599492 |
| EPI708042 | EPI637219 | EPI593186 | EPI593651 | EPI627781 | EPI581866 | EPI637085 | EPI764623 |  | EPI468995 | EPI583662 | EPI691913 | EPI599743 | EPI1093051 | EPI599499 |
| EPI708132 | EPI638048 | EPI592156 | EPI1090243 | EPI627749 | EPI583299 | EPI460778 | EPI764535 |  | EPI680653 | EPI583704 | EPI573369 | EPI599545 | EPI1092691 | EPI599746 |
| EPI708037 | EPI637314 | EPI592127 | EPI566042 | EPI655954 | EPI578140 | EPI636987 | EPI764624 |  | EPI680626 | EPI837996 | EPI838078 | EPI530451 | EPI1098430 | EPI599739 |
| EPI1179233 | EPI636995 | EPI592451 | EPI627479 | EPI655898 | EPI592194 | EPI637655 | EPI583125 |  | EPI680624 | EPI583676 | EPI846030 | EPI599580 | EPI1209280 | EPI599400 |
| EPI628536 | EPI637908 | EPI592637 | EPI627487 | EPI628100 | EPI656243 | EPI637585 | EPI443663 |  | EPI680628 | EPI838032 | EPI1092020 | EPI599397 | EPI1209319 | EPI599820 |
| EPI1090341 | EPI637544 | EPI592444 | EPI627279 | EPI628460 | EPI656212 | EPI637900 | EPI457759 |  | EPI680625 | EPI583423 | EPI1055460 | EPI600111 | EPI654249 | EPI599520 |
| EPI597033 | EPI637171 | EPI592134 | EPI627287 | EPI1090420 | EPI593301 | EPI627764 | EPI628386 |  | EPI680627 | EPI583878 | EPI577122 | EPI1155640 | EPI654231 | EPI599457 |
| EPI597077 | EPI637957 | EPI593158 | EPI1090571 | EPI1090276 | EPI1090342 | EPI620065 | EPI656380 |  | EPI680629 | EPI583592 | EPI594032 | EPI599468 | EPI1092121 | EPI599527 |
| EPI578224 | EPI637586 | EPI627566 | EPI1090723 | EPI476700 | EPI516591 | EPI637178 | EPI627515 |  | EPI680635 | EPI583620 | EPI468357 | EPI600090 | EPI1093225 | EPI599393 |
| EPI597067 | EPI638062 | EPI583093 | EPI1101013 | EPI503518 | EPI578225 | EPI637036 | EPI457655 |  | EPI680633 | EPI583550 | EPI487856 | EPI1092683 | EPI1092307 | EPI600079 |
| EPI627633 | EPI637342 | EPI583135 | EPI1090715 | EPI576760 | EPI627841 | EPI447673 | EPI457663 |  | EPI680631 | EPI596704 | EPI492297 | EPI1092107 | EPI1092301 | EPI599422 |
| EPI654153 | EPI637469 | EPI583149 | EPI971274 | EPI576763 | EPI627849 | EPI637155 | EPI507151 |  | EPI680632 | EPI469273 | EPI492294 | EPI1092263 | EPI1092223 | EPI599471 |
| EPI1090349 | EPI637299 | EPI583086 | EPI1090483 | EPI628004 | EPI597071 | EPI637127 | EPI447760 |  | EPI680648 | EPI497498 | EPI473792 | EPI1092149 | EPI1093219 | EPI599450 |
| EPI578205 | EPI637455 | EPI583142 | EPI1090283 | EPI576879 | EPI578113 | EPI447796 | EPI508930 |  | EPI680638 | EPI1091932 | EPI680733 | EPI1092299 | EPI497502 | EPI599513 |
| EPI656234 | EPI637483 | EPI627142 | EPI627263 | EPI655986 | EPI1090310 | EPI447708 | EPI895543 |  | EPI680639 | EPI1091920 | EPI680732 | EPI1092305 | EPI1091851 | EPI530494 |
| EPI566044 | EPI637712 | EPI583128 | EPI656320 | EPI576762 | EPI592187 | EPI503502 | EPI439494 |  | EPI680637 | EPI1091794 | EPI680746 | EPI652944 | EPI1091881 | EPI599548 |
| EPI627473 | EPI637156 | EPI628021 | EPI627111 | EPI576685 | EPI656164 | EPI636893 | EPI639591 |  | EPI680636 | EPI1091884 | EPI680748 | EPI599757 | EPI1091803 | EPI599562 |
| EPI627489 | EPI637016 | EPI628405 | EPI656344 | EPI576880 | EPI596908 | EPI638054 | EPI446460 |  | EPI681041 | EPI1091926 | EPI680750 | EPI1098436 | EPI1091917 | EPI599436 |
| EPI627481 | EPI637335 | EPI654143 | EPI1100701 | EPI498796 | EPI627618 | EPI578081 | EPI453479 |  | EPI681048 | EPI1091806 | EPI680751 | EPI1092983 | EPI590943 | EPI599583 |
| EPI627337 | EPI637803 | EPI592559 | EPI1102675 | EPI656112 | EPI597400 | EPI636795 | EPI447860 |  | EPI553351 | EPI1091818 | EPI838068 | EPI1055634 | EPI1092049 | EPI599478 |
| EPI627281 | EPI637247 | EPI592338 | EPI628566 | EPI576681 | EPI597078 | EPI637592 | EPI467340 |  | EPI573391 | EPI1091890 | EPI838065 | EPI1091735 | EPI838123 | EPI600086 |
| EPI627289 | EPI637420 | EPI592380 | EPI656288 | EPI576759 | EPI597168 | EPI620200 | EPI497861 |  | EPI1091709 | EPI1091866 | EPI577306 | EPI1091741 | EPI538915 | EPI599443 |
| EPI627761 | EPI638020 | EPI592029 | EPI627063 | EPI576695 | EPI578206 | EPI613784 | EPI497818 |  | EPI653010 | EPI1091854 | EPI577185 | EPI652948 | EPI1093159 | EPI599429 |
| EPI592201 | EPI637030 | EPI592106 | EPI656408 | EPI576888 | EPI656157 | EPI620136 | EPI509124 |  | EPI653001 | EPI1091860 | EPI1086309 | EPI1092893 | EPI1092007 | EPI599506 |
| EPI1090181 | EPI637254 | EPI592387 | EPI1101621 | EPI628516 | EPI578120 | EPI576604 | EPI447847 |  | EPI653005 | EPI838035 | EPI838064 | EPI641385 | EPI1092019 | EPI599407 |
| EPI656086 | EPI637593 | EPI592078 | EPI1057942 | EPI576689 | EPI627642 | EPI581778 | EPI457863 |  | EPI653003 | EPI838019 | EPI838072 | EPI1092365 | EPI1092067 | EPI599541 |
| EPI597453 | EPI637684 | EPI592401 | EPI1057950 | EPI576683 | EPI578099 | EPI636901 | EPI477406 |  | EPI652995 | EPI1092736 | EPI577146 | EPI1092671 | EPI1091887 | EPI600072 |
| EPI597446 | EPI637058 | EPI592373 | EPI1057926 | EPI576694 | EPI597034 | EPI620186 | EPI457615 |  | EPI652997 | EPI1092010 | EPI577147 | EPI1092737 | EPI1091791 | EPI683109 |
| EPI654125 | EPI637226 | EPI592071 | EPI979635 | EPI576686 | EPI516599 | EPI620061 | EPI532274 |  | EPI652999 | EPI1092022 | EPI838047 | EPI1092887 | EPI1091929 | EPI599760 |
| EPI597421 | EPI637009 | EPI592359 | EPI1103361 | EPI576687 | EPI509873 | EPI636952 | EPI532275 |  | EPI653006 | EPI1055454 | EPI598342 | EPI1092857 | EPI1091923 | EPI1155643 |
| EPI597584 | EPI637093 | EPI592050 | EPI1103973 | EPI577882 | EPI597154 | EPI596982 | EPI1057946 |  | EPI653000 | EPI583811 | EPI577299 | EPI1092863 | EPI1091785 | EPI961362 |
| EPI597427 | EPI637114 | EPI592036 | EPI971090 | EPI576884 | EPI520857 | EPI581945 | EPI446749 |  | EPI653002 | EPI583839 | EPI577301 | EPI1092845 | EPI1091815 | EPI961390 |
| EPI592186 | EPI636967 | EPI592043 | EPI887610 | EPI577881 | EPI593203 | EPI620105 | EPI895539 |  | EPI653009 | EPI583846 | EPI577302 | EPI1092827 | EPI416210 | EPI583837 |
| EPI597160 | EPI638083 | EPI592423 | EPI1102275 | EPI576877 | EPI593217 | EPI637732 | EPI447873 |  | EPI653011 | EPI583818 | EPI1091708 | EPI1092851 | EPI568618 | EPI557787 |
| EPI596907 | EPI637427 | EPI592352 | EPI1101149 | EPI628108 | EPI593231 | EPI637767 | EPI467309 |  | EPI652998 | EPI583825 | EPI680775 | EPI654443 | EPI594419 | EPI1055479 |
| EPI627201 | EPI637600 | EPI592113 | EPI1103337 | EPI655914 | EPI593224 | EPI628419 | EPI615182 |  | EPI680630 | EPI1091974 | EPI1093088 | EPI599573 | EPI557776 | EPI1055487 |
| EPI656306 | EPI637852 | EPI592415 | EPI887866 | EPI576690 | EPI593196 | EPI637192 | EPI515782 |  | EPI583716 | EPI623616 | EPI1093166 | EPI530493 | EPI532317 | EPI1092774 |
| EPI627273 | EPI637551 | EPI592057 | EPI1103311 | EPI576761 | EPI593238 | EPI637113 | EPI439499 |  | EPI583633 | EPI1091758 | EPI1093100 | EPI530469 | EPI680952 | EPI1055450 |
| EPI656274 | EPI637086 | EPI592394 | EPI887882 | EPI576682 | EPI1090350 | EPI576602 | EPI447634 |  | EPI592001 | EPI1092448 | EPI1093148 | EPI530475 | EPI680954 | EPI652953 |
| EPI1090309 | EPI637328 | EPI592085 | EPI887890 | EPI576684 | EPI596920 | EPI457864 | EPI464585 |  | EPI530515 | EPI1091950 | EPI680758 | EPI530481 | EPI594391 | EPI652956 |
| EPI656506 | EPI637121 | EPI592331 | EPI1101157 | EPI576878 | EPI597422 | EPI638075 | EPI457823 |  | EPI583337 | EPI1092568 | EPI680757 | EPI599677 | EPI594462 | EPI652952 |
| EPI597399 | EPI637943 | EPI592408 | EPI1090611 | EPI576886 | EPI597428 | EPI457720 | EPI692013 |  | EPI654338 | EPI1091914 | EPI680754 | EPI602283 | EPI594455 | EPI652967 |
| EPI627848 | EPI637523 | EPI592324 | EPI1013247 | EPI576882 | EPI597161 | EPI637956 | EPI568651 |  | EPI654344 | EPI1092376 | EPI680770 | EPI602364 | EPI594469 | EPI654305 |
| EPI627856 | EPI637565 | EPI592345 | EPI1018166 | EPI627932 | EPI597454 | EPI638040 | EPI620189 |  | EPI530503 | EPI1092406 | EPI680759 | EPI1092083 | EPI1103564 | EPI654310 |
| EPI627625 | EPI637999 | EPI592170 | EPI1102135 | EPI655978 | EPI597585 | EPI637669 | EPI457623 |  | EPI530509 | EPI599755 | EPI680761 | EPI1055697 | EPI1103566 | EPI654316 |
| EPI627689 | EPI637670 | EPI592092 | EPI1103353 | EPI447663 | EPI596804 | EPI439497 | EPI451258 |  | EPI654385 | EPI692129 | EPI680755 | EPI1092797 | EPI1103565 | EPI583358 |
| EPI656203 | EPI637462 | EPI592366 | EPI971266 | EPI457849 | EPI597447 | EPI620086 | EPI692000 |  | EPI653004 | EPI557812 | EPI680753 | EPI1091867 | EPI1103567 | EPI1061407 |
| EPI656171 | EPI637292 | EPI592064 | EPI971226 | EPI691860 | EPI578106 | EPI581925 | EPI447704 |  | EPI596703 | EPI1155637 | EPI680756 | EPI1092917 | EPI594434 | EPI1092186 |
| EPI578105 | EPI637635 | EPI592022 | EPI1101189 | EPI447677 | EPI654147 | EPI636753 | EPI440679 |  | EPI569601 | EPI654228 | EPI680752 | EPI1098438 | EPI466210 | EPI654254 |
| EPI578112 | EPI637733 | EPI592430 | EPI1054124 | EPI497930 | EPI596760 | EPI636702 | EPI691661 |  | EPI596688 | EPI654264 | EPI591179 | EPI1092053 | EPI594441 | EPI583457 |
| EPI578098 | EPI637179 | EPI583019 | EPI1103917 | EPI453837 | EPI597182 | EPI581612 | EPI447711 |  | EPI683193 | EPI1092754 | EPI577149 | EPI1092089 | EPI680944 | EPI583844 |
| EPI578119 | EPI638076 | EPI583012 | EPI1103909 | EPI509092 | EPI627682 | EPI583314 | EPI447918 |  | EPI591954 | EPI1091824 | EPI577148 | EPI530511 | EPI680961 | EPI583851 |
| EPI593319 | EPI637263 | EPI628253 | EPI1102507 | EPI691863 | EPI656227 | EPI597247 | EPI692005 |  | EPI681075 | EPI1091800 | EPI577186 | EPI530457 | EPI572735 | EPI583492 |
| EPI593348 | EPI638027 | EPI582981 | EPI1102523 | EPI691856 | EPI627714 | EPI637781 | EPI628410 |  | EPI681076 | EPI1092820 | EPI652782 | EPI599566 | EPI572742 | EPI583541 |
| EPI593355 | EPI637894 | EPI582942 | EPI971506 | EPI692636 | EPI627754 | EPI637774 | EPI457719 |  | EPI591934 | EPI1092382 | EPI652796 | EPI530463 | EPI845711 | EPI583527 |
| EPI593312 | EPI636988 | EPI582957 | EPI1103372 | EPI692634 | EPI656133 | EPI498090 | EPI692001 |  | EPI596724 | EPI1092484 | EPI652783 | EPI530517 | EPI568793 | EPI838203 |
| EPI593294 | EPI637754 | EPI582345 | EPI971234 | EPI691866 | EPI627626 | EPI620193 | EPI620182 |  | EPI594365 | EPI1092286 | EPI652781 | EPI578044 | EPI568787 | EPI654394 |
| EPI593306 | EPI637614 | EPI583005 | EPI971242 | EPI692638 | EPI593295 | EPI637844 | EPI535173 |  | EPI767540 | EPI844502 | EPI652785 | EPI599764 | EPI599385 | EPI583646 |
| EPI593209 | EPI637859 | EPI582352 | EPI443657 | EPI691868 | EPI593307 | EPI613808 | EPI583195 |  | EPI767554 | EPI1092400 | EPI1091834 | EPI602290 | EPI1155642 | EPI583485 |
| EPI593300 | EPI637628 | EPI582359 | EPI446456 | EPI691862 | EPI593349 | EPI628507 | EPI447718 |  | EPI594443 | EPI1092274 | EPI1091846 | EPI599376 | EPI497514 | EPI583737 |
| EPI656250 | EPI637985 | EPI582964 | EPI443649 | EPI586010 | EPI593356 | EPI636834 | EPI457767 |  | EPI594464 | EPI1092544 | EPI680764 | EPI1092989 | EPI497527 | EPI654425 |
| EPI708040 | EPI637186 | EPI582935 | EPI532296 | EPI447768 | EPI593210 | EPI440097 | EPI457751 |  | EPI594457 | EPI578197 | EPI680765 | EPI591950 | EPI692270 | EPI583890 |
| EPI708038 | EPI637240 | EPI583042 | EPI443673 | EPI447820 | EPI593313 | EPI597120 | EPI576585 |  | EPI594450 | EPI736583 | EPI680771 | EPI1093043 | EPI1091749 | EPI592309 |
| EPI708041 | EPI636902 | EPI628261 | EPI457763 | EPI443033 | EPI593320 | EPI656221 | EPI442725 |  | EPI591927 | EPI683151 | EPI680749 | EPI599635 | EPI568665 | EPI583715 |
| EPI708039 | EPI637607 | EPI628309 | EPI1090809 | EPI441599 | EPI597040 | EPI637760 | EPI443574 |  | EPI680647 | EPI683153 | EPI680772 | EPI1091831 | EPI692276 | EPI654450 |
| EPI580327 | EPI637810 | EPI592221 | EPI627519 | EPI447940 | EPI502366 | EPI581938 | EPI447799 |  | EPI515818 | EPI1076649 | EPI680747 | EPI1091795 | EPI838145 | EPI654486 |
| EPI627065 | EPI637880 | EPI627805 | EPI1090187 | EPI447874 | EPI596973 | EPI636908 | EPI457879 |  | EPI506284 | EPI942222 | EPI838054 | EPI1092725 | EPI470065 | EPI654224 |
| EPI656410 | EPI637378 | EPI627901 | EPI656504 | EPI467320 | EPI628009 | EPI597313 | EPI692004 |  | EPI469001 | EPI1092118 | EPI681088 | EPI1092041 | EPI470061 | EPI654214 |
| EPI580267 | EPI637065 | EPI627885 | EPI1090347 | EPI504734 | EPI656039 | EPI637218 | EPI691660 |  | EPI468985 | EPI858164 | EPI654172 | EPI1093025 | EPI470067 | EPI654197 |
| EPI580347 | EPI637530 | EPI627821 | EPI626991 | EPI504737 | EPI597566 | EPI457744 | EPI457783 |  | EPI680640 | EPI1092622 | EPI844508 | EPI1091981 | EPI470062 | EPI654246 |
| EPI580355 | EPI637579 | EPI627893 | EPI1090411 | EPI509076 | EPI627690 | EPI638103 | EPI545806 |  | EPI764805 | EPI1092574 | EPI1092884 | EPI1092023 | EPI470066 | EPI654213 |
| EPI580313 | EPI638013 | EPI592286 | EPI887834 | EPI515451 | EPI656204 | EPI637134 | EPI464588 |  | EPI468977 | EPI1092598 | EPI838060 | EPI1091987 | EPI515841 | EPI654410 |
| EPI566092 | EPI637768 | EPI592249 | EPI930805 | EPI515474 | EPI596987 | EPI581605 | EPI447789 |  | EPI468981 | EPI1092364 | EPI1093196 | EPI1092059 | EPI470063 | EPI1062123 |
| EPI656258 | EPI637978 | EPI593242 | EPI1101557 | EPI445911 | EPI597520 | EPI636788 | EPI509098 |  | EPI466463 | EPI1093132 | EPI1034479 | EPI578068 | EPI470069 | EPI583569 |
| EPI580275 | EPI637413 | EPI593276 | EPI1022647 | EPI515466 | EPI1090334 | EPI656049 | EPI692014 |  | EPI591961 | EPI1092628 | EPI1034481 | EPI602357 | EPI515833 | EPI583590 |
| EPI580283 | EPI637831 | EPI592163 | EPI884220 | EPI516361 | EPI654131 | EPI578088 | EPI447739 |  | EPI596681 | EPI1091788 | EPI1034482 | EPI1093235 | EPI470060 | EPI583660 |
| EPI628712 | EPI636974 | EPI592552 | EPI1040485 | EPI515458 | EPI1090382 | EPI613770 | EPI545799 |  | EPI837959 | EPI1092634 | EPI1034483 | EPI1092275 | EPI470064 | EPI581662 |
| EPI580291 | EPI637448 | EPI592597 | EPI1101653 | EPI452274 | EPI553200 | EPI636945 | EPI461481 |  | EPI1155611 | EPI1092616 | EPI1034478 | EPI838100 | EPI497543 | EPI583548 |
| EPI580299 | EPI637277 | EPI592214 | EPI997215 | EPI452275 | EPI578186 | EPI620047 | EPI498091 |  | EPI596717 | EPI1092316 | EPI1034480 | EPI1091927 | EPI1092139 | EPI490989 |
| EPI580337 | EPI637270 | EPI592616 | EPI926815 | EPI576563 | EPI578002 | EPI596820 | EPI497898 |  | EPI680646 | EPI1092778 | EPI681093 | EPI1091789 | EPI1092199 | EPI490997 |
| EPI656362 | EPI637002 | EPI592120 | EPI1013223 | EPI457641 | EPI507080 | EPI497868 | EPI613818 |  | EPI1155628 | EPI1092082 | EPI416223 | EPI1091879 | EPI1098428 | EPI491005 |
| EPI566036 | EPI637929 | EPI592474 | EPI960357 | EPI576816 | EPI583278 | EPI637468 | EPI504723 |  | EPI680652 | EPI1092784 | EPI416224 | EPI1092329 | EPI1098429 | EPI654326 |
| EPI656298 | EPI637838 | EPI592545 | EPI919605 | EPI576829 | EPI578009 | EPI578155 | EPI447958 |  | EPI691735 | EPI942238 | EPI529225 | EPI1103202 | EPI591952 | EPI583576 |
| EPI1179234 | EPI637642 | EPI592256 | EPI960356 | EPI443650 | EPI656103 | EPI637753 | EPI663500 |  | EPI591947 | EPI1091710 | EPI466294 | EPI1091963 | EPI591945 | EPI583722 |
| EPI656402 | EPI637761 | EPI592623 | EPI656496 | EPI508923 | EPI597503 | EPI636695 | EPI460779 |  | EPI680668 | EPI1098452 | EPI466342 | EPI1092755 | EPI680983 | EPI583653 |
| EPI627057 | EPI636981 | EPI593151 | EPI627782 | EPI443642 | EPI656125 | EPI581734 | EPI516365 |  | EPI680669 | EPI1090079 | EPI466398 | EPI1093169 | EPI680981 | EPI583618 |
| EPI656442 | EPI637845 | EPI592149 | EPI656068 | EPI447761 | EPI597047 | EPI509101 | EPI457831 |  | EPI528383 | EPI1092076 | EPI466334 | EPI1091975 | EPI680984 | EPI583632 |
| EPI1090253 | EPI637193 | EPI592235 | EPI627950 | EPI515795 | EPI691937 | EPI636688 | EPI457807 |  | EPI548394 | EPI1092172 | EPI466422 | EPI1093103 | EPI680967 | EPI583695 |
| EPI866577 | EPI637824 | EPI1090666 | EPI656020 | EPI457625 | EPI628433 | EPI637050 | EPI448940 |  | EPI528399 | EPI1092322 | EPI466239 | EPI1093145 | EPI680976 | EPI583688 |
| EPI887980 | EPI637621 | EPI656367 | EPI627974 | EPI663509 | EPI656499 | EPI627604 | EPI451276 |  | EPI837963 | EPI1092478 | EPI466246 | EPI683063 | EPI590945 | EPI583506 |
| EPI972246 | EPI637691 | EPI656463 | EPI656028 | EPI467323 | EPI628529 | EPI490877 | EPI452264 |  | EPI599779 | EPI1092508 | EPI466414 | EPI1055705 | EPI838133 | EPI583702 |
| EPI972257 | EPI637441 | EPI627070 | EPI656328 | EPI460750 | EPI578240 | EPI637384 | EPI530821 |  | EPI594372 | EPI1092514 | EPI466270 | EPI1055657 | EPI590946 | EPI583583 |
| EPI1101647 | EPI637364 | EPI656391 | EPI628646 | EPI497937 | EPI577960 | EPI620079 | EPI531122 |  | EPI594379 | EPI1092340 | EPI767541 | EPI942929 | EPI838139 | EPI490981 |
| EPI972239 | EPI637663 | EPI528335 | EPI627207 | EPI453649 | EPI581583 | EPI637078 | EPI613780 |  | EPI583738 | EPI1092502 | EPI767555 | EPI729788 | EPI1098426 | EPI491021 |
| EPI1101615 | EPI637817 | EPI528295 | EPI1090251 | EPI498105 | EPI581577 | EPI636774 | EPI620203 |  | EPI583782 | EPI1092424 | EPI572880 | EPI1055713 | EPI1092661 | EPI583562 |
| EPI972270 | EPI637873 | EPI528343 | EPI1090323 | EPI457737 | EPI583271 | EPI636864 | EPI457775 |  | EPI568784 | EPI1092346 | EPI466382 | EPI1092353 | EPI1092055 | EPI583471 |
| EPI972266 | EPI637107 | EPI528359 | EPI580361 | EPI1090796 | EPI509508 | EPI620112 | EPI471842 |  | EPI1103534 | EPI1093138 | EPI591177 | EPI1092899 | EPI1091737 | EPI583674 |
| EPI972268 | EPI637149 | EPI533256 | EPI656280 | EPI627517 | EPI490875 | EPI470074 | EPI515800 |  | EPI1103537 | EPI1093144 | EPI466406 | EPI1091807 | EPI1091743 | EPI583336 |
| EPI1040451 | EPI637502 | EPI628317 | EPI580345 | EPI1090188 | EPI656395 | EPI636681 | EPI620171 |  | EPI1103536 | EPI1092430 | EPI572881 | EPI1092923 | EPI1092895 | EPI838183 |
| EPI972301 | EPI637044 | EPI535136 | EPI580353 | EPI628220 | EPI627050 | EPI581910 | EPI446966 |  | EPI1103532 | EPI1092580 | EPI568778 | EPI1093175 | EPI1092775 | EPI654260 |
| EPI930807 | EPI637495 | EPI535160 | EPI866567 | EPI628196 | EPI656435 | EPI576601 | EPI443655 |  | EPI1103533 | EPI530486 | EPI635480 | EPI1093181 | EPI1092919 | EPI1092132 |
| EPI1101559 | EPI637936 | EPI628325 | EPI553468 | EPI628148 | EPI869666 | EPI581882 | EPI464581 |  | EPI846036 | EPI530468 | EPI568777 | EPI1092875 | EPI1092847 | EPI654480 |
| EPI972263 | EPI637705 | EPI535144 | EPI627271 | EPI628268 | EPI926842 | EPI627668 | EPI457695 |  | EPI654362 | EPI530522 | EPI572893 | EPI1092929 | EPI1092853 | EPI1209320 |
| EPI887836 | EPI637796 | EPI628333 | EPI656272 | EPI628156 | EPI874331 | EPI637447 | EPI1090822 |  | EPI654350 | EPI599741 | EPI572885 | EPI1092953 | EPI1092859 | EPI1098445 |
| EPI974523 | EPI637128 | EPI628141 | EPI831454 | EPI628204 | EPI977388 | EPI637071 | EPI447662 |  | EPI654356 | EPI599762 | EPI572898 | EPI1092959 | EPI1092829 | EPI1209281 |
| EPI628648 | EPI637747 | EPI542090 | EPI580273 | EPI531806 | EPI1090414 | EPI450853 | EPI445916 |  | EPI681049 | EPI599585 | EPI560314 | EPI1055494 | EPI1092865 | EPI654218 |
| EPI1090325 | EPI637572 | EPI627542 | EPI566090 | EPI656183 | EPI979642 | EPI637363 | EPI692010 |  | EPI1092393 | EPI599564 | EPI466430 | EPI1055486 | EPI1086301 | EPI654444 |
| EPI580363 | EPI637135 | EPI627558 | EPI580281 | EPI531815 | EPI457646 | EPI636923 | EPI613832 |  | EPI1092201 | EPI578042 | EPI465412 | EPI1055689 | EPI1092733 | EPI583421 |
| EPI656330 | EPI638097 | EPI628237 | EPI580265 | EPI531809 | EPI439491 | EPI637816 | EPI451018 |  | EPI529276 | EPI1092280 | EPI467589 | EPI1092533 | EPI568615 | EPI583464 |
| EPI626993 | EPI637406 | EPI533247 | EPI580297 | EPI531812 | EPI457670 | EPI637199 | EPI457743 |  | EPI583803 | EPI578066 | EPI590907 | EPI1098512 | EPI596715 | EPI654436 |
| EPI627209 | EPI637142 | EPI533240 | EPI580311 | EPI656222 | EPI457654 | EPI636931 | EPI457847 |  | EPI599563 | EPI530462 | EPI590908 | EPI1098511 | EPI838142 | EPI843884 |
| EPI656282 | EPI637677 | EPI509998 | EPI656296 | EPI692633 | EPI583167 | EPI450845 | EPI509091 |  | EPI599584 | EPI530498 | EPI591191 | EPI1098513 | EPI1093165 | EPI654456 |
| EPI656290 | EPI637656 | EPI535120 | EPI580325 | EPI532268 | EPI1090206 | EPI636723 | EPI487904 |  | EPI599674 | EPI602281 | EPI568780 | EPI1092659 | EPI1093123 | EPI838196 |
| EPI656498 | EPI636886 | EPI628301 | EPI566034 | EPI447949 | EPI628217 | EPI627971 | EPI457711 |  | EPI602287 | EPI602362 | EPI492300 | EPI1092599 | EPI1093111 | EPI1093110 |
| EPI1101583 | EPI637992 | EPI628341 | EPI580289 | EPI447888 | EPI628385 | EPI637872 | EPI457432 |  | EPI530521 | EPI599675 | EPI568779 | EPI1093199 | EPI1093153 | EPI1093122 |
| EPI972276 | EPI457780 | EPI627837 | EPI566670 | EPI663515 | EPI627658 | EPI446748 | EPI447895 |  | EPI602280 | EPI602288 | EPI515803 | EPI1092065 | EPI583815 | EPI1093164 |
| EPI972259 | EPI497875 | EPI627406 | EPI656360 | EPI691858 | EPI656079 | EPI447813 | EPI627603 |  | EPI602361 | EPI602355 | EPI515812 | EPI1092695 | EPI530495 | EPI1092306 |
| EPI1101631 | EPI464513 | EPI627446 | EPI580335 | EPI532269 | EPI532290 | EPI637550 | EPI583188 |  | EPI1155619 | EPI530474 | EPI466478 | EPI1092071 | EPI599378 | EPI654248 |
| EPI1090413 | EPI447744 | EPI627414 | EPI628710 | EPI443674 | EPI532291 | EPI636980 | EPI443639 |  | EPI683191 | EPI530480 | EPI680744 | EPI1091711 | EPI599738 | EPI1055474 |
| EPI972273 | EPI508928 | EPI628061 | EPI559415 | EPI442720 | EPI457782 | EPI620154 | EPI591861 |  | EPI1055461 | EPI599374 | EPI532335 | EPI1091717 | EPI1092433 | EPI1055502 |
| EPI972272 | EPI585980 | EPI566121 | EPI628078 | EPI457617 | EPI460768 | EPI637830 | EPI447697 |  | EPI837994 | EPI1092442 | EPI680745 | EPI1092665 | EPI1090074 | EPI654289 |
| EPI972275 | EPI545790 | EPI628293 | EPI627814 | EPI457713 | EPI573430 | EPI581771 | EPI576584 |  | EPI846028 | EPI1092472 | EPI680774 | EPI1092941 | EPI1092703 | EPI583823 |
| EPI887844 | EPI457676 | EPI627598 | EPI627878 | EPI576873 | EPI509123 | EPI581652 | EPI692008 |  | EPI729798 | EPI1092238 | EPI529227 | EPI942175 | EPI599463 | EPI583816 |
| EPI972248 | EPI497912 | EPI627574 | EPI628774 | EPI439498 | EPI443660 | EPI637914 | EPI620196 |  | EPI844485 | EPI838016 | EPI680763 | EPI1055665 | EPI599745 | EPI583830 |
| EPI972250 | EPI442717 | EPI627582 | EPI628822 | EPI443634 | EPI457758 | EPI656017 | EPI663522 |  | EPI654287 | EPI1093240 | EPI691909 | EPI1055673 | EPI599512 | EPI594392 |
| EPI1036702 | EPI450527 | EPI535176 | EPI627031 | EPI542255 | EPI443652 | EPI597561 | EPI439510 |  | EPI654374 | EPI1093234 | EPI680762 | EPI1055681 | EPI599491 | EPI466534 |
| EPI1040487 | EPI542313 | EPI535168 | EPI971282 | EPI596649 | EPI628361 | EPI636820 | EPI636677 |  | EPI654269 | EPI1092298 | EPI1155621 | EPI1055478 | EPI599519 | EPI515826 |
| EPI972284 | EPI487903 | EPI535184 | EPI627159 | EPI497890 | EPI453605 | EPI637426 | EPI497877 |  | EPI654379 | EPI1091734 | EPI466470 | EPI1055719 | EPI600099 | EPI764637 |
| EPI972282 | EPI457652 | EPI627390 | EPI552397 | EPI457633 | EPI469535 | EPI581931 | EPI497840 |  | EPI654418 | EPI1006842 | EPI572873 | EPI1055727 | EPI600113 | EPI681032 |
| EPI1101655 | EPI615187 | EPI628037 | EPI627183 | EPI447959 | EPI451267 | EPI637725 | EPI457703 |  | EPI654434 | EPI1092892 | EPI572869 | EPI1055614 | EPI599406 | EPI681018 |
| EPI972234 | EPI457668 | EPI627526 | EPI627175 | EPI440681 | EPI447731 | EPI619986 | EPI515456 |  | EPI654244 | EPI1092370 | EPI680734 | EPI1055622 | EPI599484 | EPI680991 |
| EPI1101599 | EPI497851 | EPI627614 | EPI628830 | EPI545207 | EPI447805 | EPI542314 | EPI440695 |  | EPI654238 | EPI1092748 | EPI680736 | EPI1092347 | EPI599414 | EPI680993 |
| EPI1101663 | EPI442711 | EPI627534 | EPI628798 | EPI457721 | EPI627514 | EPI447666 | EPI509107 |  | EPI654211 | EPI1093042 | EPI680742 | EPI1092947 | EPI599428 | EPI680990 |
| EPI1018220 | EPI447716 | EPI535200 | EPI1090563 | EPI576562 | EPI1090812 | EPI637246 | EPI692007 |  | EPI654441 | EPI1092676 | EPI680740 | EPI1191039 | EPI599421 | EPI680992 |
| EPI1101639 | EPI443653 | EPI535192 | EPI1090547 | EPI509114 | EPI457710 | EPI497852 | EPI613811 |  | EPI837970 | EPI1092742 | EPI532333 | EPI1191036 | EPI599399 | EPI764735 |
| EPI972296 | EPI532270 | EPI535152 | EPI1102971 | EPI576875 | EPI509059 | EPI453651 | EPI447641 |  | EPI1090080 | EPI652988 | EPI680735 | EPI1191037 | EPI599456 | EPI466324 |
| EPI972292 | EPI460753 | EPI628045 | EPI628814 | EPI448945 | EPI457702 | EPI637163 | EPI576587 |  | EPI1093035 | EPI1098451 | EPI466462 | EPI1076646 | EPI1092991 | EPI466308 |
| EPI972287 | EPI503501 | EPI594169 | EPI1090619 | EPI497934 | EPI691932 | EPI451275 | EPI452272 |  | EPI1092231 | EPI1092088 | EPI487847 | EPI942162 | EPI1093003 | EPI764759 |
| EPI972285 | EPI577961 | EPI594295 | EPI1100517 | EPI457873 | EPI691934 | EPI637802 | EPI446455 |  | EPI1092909 | EPI1092064 | EPI506277 | EPI942177 | EPI1092997 | EPI468354 |
| EPI972290 | EPI447772 | EPI583909 | EPI833712 | EPI457689 | EPI691933 | EPI515872 | EPI691659 |  | EPI1092915 | EPI1092730 | EPI680766 | EPI1092707 | EPI1093009 | EPI466356 |
| EPI972294 | EPI457620 | EPI594239 | EPI1090499 | EPI545208 | EPI691652 | EPI581585 | EPI576582 |  | EPI837992 | EPI1092724 | EPI680737 | EPI1092809 | EPI1093015 | EPI681004 |
| EPI972289 | EPI447674 | EPI594246 | EPI532273 | EPI691861 | EPI691940 | EPI637284 | EPI450854 |  | EPI681043 | EPI1091872 | EPI680738 | EPI1091723 | EPI1092517 | EPI532324 |
| EPI972295 | EPI443637 | EPI594337 | EPI450848 | EPI447712 | EPI691938 | EPI638033 | EPI460771 |  | EPI681042 | EPI1092802 | EPI466446 | EPI1091777 | EPI1092325 | EPI681007 |
| EPI503509 | EPI457660 | EPI594351 | EPI457651 | EPI447670 | EPI691944 | EPI638012 | EPI443679 |  | EPI681051 | EPI1092898 | EPI466454 | EPI1098514 | EPI652908 | EPI599386 |
| EPI581567 | EPI576571 | EPI583930 | EPI627926 | EPI447635 | EPI451020 | EPI637536 | EPI692003 |  | EPI681050 | EPI1092034 | EPI466500 | EPI1092173 | EPI1092079 | EPI838168 |
| EPI443025 | EPI515798 | EPI594323 | EPI627399 | EPI490975 | EPI583192 | EPI637106 | EPI764619 |  | EPI681054 | EPI1092040 | EPI487842 | EPI1092641 | EPI1092781 | EPI578040 |
| EPI542311 | EPI443026 | EPI594344 | EPI628030 | EPI692635 | EPI591858 | EPI637405 | EPI497922 |  | EPI681056 | EPI1092922 | EPI577316 | EPI1092653 | EPI1006756 | EPI600128 |
| EPI457749 | EPI443044 | EPI583937 | EPI628214 | EPI691857 | EPI477314 | EPI637148 | EPI469515 |  | EPI683194 | EPI1091740 | EPI577317 | EPI1092635 | EPI1092367 | EPI538916 |
| EPI451278 | EPI497896 | EPI594162 | EPI628166 | EPI457681 | EPI447689 | EPI637851 | EPI504718 |  | EPI1093197 | EPI1091746 | EPI487839 | EPI441097 | EPI1092889 | EPI569474 |
| EPI447611 | EPI577906 | EPI583897 | EPI628174 | EPI663518 | EPI509083 | EPI470073 | EPI692002 |  | EPI837976 | EPI1091878 | EPI487840 | EPI569699 | EPI641387 | EPI221857 |
| EPI447605 | EPI443677 | EPI594330 | EPI528376 | EPI692637 | EPI691946 | EPI637269 | EPI447676 |  | EPI1034490 | EPI1091908 | EPI557657 | EPI221852 | EPI1092673 | EPI569476 |
| EPI585979 | EPI447758 | EPI594211 | EPI528368 | EPI443571 | EPI583215 | EPI620161 | EPI457631 |  | EPI1034494 | EPI1098453 | EPI680723 | EPI313911 | EPI1092739 | EPI441100 |
| EPI1179236 | EPI450852 | EPI583903 | EPI528352 | EPI691867 | EPI471841 | EPI578174 | EPI447753 |  | EPI1034492 | EPI1092832 | EPI680724 | EPI313919 | EPI1092799 | EPI314016 |
| EPI504714 | EPI471838 | EPI594183 | EPI895605 | EPI509065 | EPI457814 | EPI509088 | EPI453613 |  | EPI1034495 | EPI1092850 | EPI532334 | EPI314013 | EPI1092337 | EPI313922 |
| EPI451254 | EPI627608 | EPI583891 | EPI895612 | EPI447791 | EPI691935 | EPI636915 | EPI509115 |  | EPI1034491 | EPI1092856 | EPI450830 | EPI243524 | EPI1092343 | EPI313914 |
| EPI504725 | EPI450844 | EPI594232 | EPI895593 | EPI691859 | EPI447724 | EPI447779 | EPI692011 |  | EPI1034493 | EPI1092862 | EPI680743 | EPI243540 | EPI1076635 | EPI317185 |
| EPI515796 | EPI447639 | EPI594148 | EPI895610 | EPI457443 | EPI691936 | EPI637482 | EPI447774 |  | EPI681044 | EPI1092868 | EPI577336 | EPI466315 | EPI1092571 | EPI469497 |
| EPI457629 | EPI457644 | EPI592177 | EPI895591 | EPI497844 | EPI691943 | EPI637253 | EPI576583 |  | EPI681047 | EPI530492 | EPI838043 | EPI351702 | EPI1092595 | EPI692039 |
| EPI942077 | EPI443645 | EPI592317 | EPI895598 | EPI569716 | EPI691947 | EPI637291 | EPI464587 |  | EPI681055 | EPI599571 | EPI974028 | EPI1155600 | EPI1091947 | EPI681038 |
| EPI942085 | EPI498100 | EPI594316 | EPI1018253 | EPI447919 | EPI691939 | EPI636827 | EPI615185 |  | EPI654471 | EPI530516 | EPI1055444 | EPI466435 | EPI1092415 | EPI1093086 |
| EPI457661 | EPI464512 | EPI594197 | EPI1101685 | EPI468975 | EPI691942 | EPI597306 | EPI440687 |  | EPI837991 | EPI1092436 | EPI681087 | EPI466299 | EPI736588 | EPI596716 |
| EPI509071 | EPI476703 | EPI583916 | EPI1102955 | EPI469562 | EPI457822 | EPI628467 | EPI515472 |  | EPI1092687 | EPI1092772 | EPI577333 | EPI441181 | EPI1092361 | EPI764743 |
| EPI443643 | EPI443023 | EPI594267 | EPI1102963 | EPI453620 | EPI477313 | EPI637795 | EPI471843 |  | EPI729799 | EPI838025 | EPI591181 | EPI441099 | EPI1092529 | EPI652954 |
| EPI457669 | EPI532294 | EPI594218 | EPI1102947 | EPI572696 | EPI583185 | EPI460754 | EPI457799 |  | EPI1092177 | EPI1093012 | EPI468351 | EPI466363 | EPI1092427 | EPI764636 |
| EPI457653 | EPI457748 | EPI594204 | EPI683160 | EPI469565 | EPI457686 | EPI637557 | EPI583211 |  | EPI466391 | EPI1093000 | EPI1098449 | EPI569638 | EPI1092373 | EPI764632 |
| EPI457677 | EPI447765 | EPI594141 | EPI626983 | EPI467337 | EPI457806 | EPI637998 | EPI447655 |  | EPI461701 | EPI1093018 | EPI1092002 | EPI466504 | EPI1092577 | EPI764638 |
| EPI508926 | EPI663447 | EPI594190 | EPI627071 | EPI447862 | EPI447703 | EPI443024 | EPI613773 |  | EPI572947 | EPI1092994 | EPI1092140 | EPI466510 | EPI1093141 | EPI466332 |
| EPI507147 | EPI509073 | EPI592310 | EPI656392 | EPI497883 | EPI691651 | EPI509069 | EPI692015 |  | EPI691842 | EPI1093006 | EPI451170 | EPI572843 | EPI1092403 | EPI466276 |
| EPI447612 | EPI497867 | EPI594134 | EPI628662 | EPI639311 | EPI457694 | EPI545791 | EPI477315 |  | EPI572960 | EPI654466 | EPI654343 | EPI568757 | EPI1093135 | EPI466260 |
| EPI447610 | EPI577920 | EPI594309 | EPI1090555 | EPI453616 | EPI440694 | EPI637935 | EPI515792 |  | EPI572972 | EPI1092808 | EPI654331 | EPI568761 | EPI599392 | EPI466348 |
| EPI576569 | EPI497831 | EPI594253 | EPI628606 | EPI446457 | EPI691653 | EPI620072 | EPI457639 |  | EPI466255 | EPI1092838 | EPI1092188 | EPI568799 | EPI583836 | EPI466396 |
| EPI457645 | EPI583230 | EPI594155 | EPI1090539 | EPI447849 | EPI443668 | EPI636973 | EPI613839 |  | EPI569466 | EPI1092844 | EPI1092122 | EPI569564 | EPI583850 | EPI466340 |
| EPI532301 | EPI447829 | EPI594176 | EPI628734 | EPI497826 | EPI457838 | EPI638096 | EPI457855 |  | EPI529275 | EPI1092796 | EPI1092302 | EPI569566 | EPI583822 | EPI466292 |
| EPI1090581 | EPI581584 | EPI594281 | EPI628726 | EPI576832 | EPI457678 | EPI637984 | EPI457727 |  | EPI416228 | EPI1092826 | EPI1092224 | EPI569701 | EPI583829 | EPI692026 |
| EPI510157 | EPI490881 | EPI594260 | EPI627471 | EPI628244 | EPI583208 | EPI592211 | EPI443647 |  | EPI680644 | EPI1092766 | EPI568440 | EPI470526 | EPI1091995 | EPI469522 |
| EPI628056 | EPI654031 | EPI594225 | EPI627231 | EPI628132 | EPI457798 | EPI476704 | EPI457871 |  | EPI592015 | EPI1093252 | EPI515821 | EPI529262 | EPI1092001 | EPI470812 |
| EPI708080 | EPI509506 | EPI583923 | EPI656488 | EPI1090476 | EPI447647 | EPI636738 | EPI457815 |  | EPI680645 | EPI1092460 | EPI577337 | EPI490980 | EPI1091827 | EPI583329 |
| EPI708081 | EPI583272 | EPI594274 | EPI656472 | EPI1090468 | EPI461482 | EPI637341 | EPI692006 |  | EPI837964 | EPI1092814 | EPI572890 | EPI838104 | EPI858161 | EPI583520 |
| EPI627129 | EPI654024 | EPI594288 | EPI627023 | EPI628084 | EPI447894 | EPI637170 | EPI581570 |  | EPI468349 | EPI1092208 | EPI497499 | EPI583482 | EPI578201 | EPI484495 |
| EPI628032 | EPI581578 | EPI594302 | EPI656336 | EPI628380 | EPI1055425 | EPI443030 | EPI457839 |  | EPI557658 | EPI1092214 | EPI1091792 | EPI583378 | EPI1092751 | EPI569572 |
| EPI627401 | EPI654026 | EPI593317 | EPI656352 | EPI628180 | EPI532267 | EPI597327 | EPI515464 |  | EPI680654 | EPI1092244 | EPI1091804 | EPI583418 | EPI1092283 | EPI568765 |
| EPI628368 | EPI628415 | EPI576607 | EPI627119 | EPI628348 | EPI1090819 | EPI638019 | EPI515449 |  | EPI450828 | EPI1098525 | EPI1091816 | EPI583461 | EPI1091821 | EPI681037 |
| EPI627928 | EPI457732 | EPI515793 | EPI656432 | EPI628188 | EPI503513 | EPI637648 | EPI656332 |  | EPI680621 | EPI1098523 | EPI1091858 | EPI583615 | EPI843871 | EPI764647 |
| EPI628176 | EPI498089 | EPI447785 | EPI628686 | EPI531492 | EPI457630 | EPI515781 | EPI627019 |  | EPI680655 | EPI1098524 | EPI1091864 | EPI691966 | EPI1092259 | EPI492261 |
| EPI628168 | EPI1090583 | EPI583070 | EPI1090355 | EPI628428 | EPI447682 | EPI636857 | EPI773704 |  | EPI532314 | EPI1055603 | EPI1091852 | EPI583712 | EPI843878 | EPI492268 |
| EPI628216 | EPI510155 | EPI1090578 | EPI627455 | EPI577887 | EPI497837 | EPI597341 | EPI627363 |  | EPI680666 | EPI1055491 | EPI838077 | EPI491012 | EPI843877 | EPI681003 |
| EPI620167 | EPI628055 | EPI576615 | EPI941045 | EPI656191 | EPI583103 | EPI497913 | EPI769587 |  | EPI680665 | EPI1055662 | EPI654286 | EPI491028 | EPI623621 | EPI764649 |
| EPI620160 | EPI627400 | EPI1102546 | EPI627239 | EPI627868 | EPI663482 | EPI637599 | EPI1090225 |  | EPI680667 | EPI1055678 | EPI838056 | EPI583685 | EPI1092607 | EPI568669 |
| EPI620199 | EPI628367 | EPI1101372 | EPI628638 | EPI627804 | EPI447773 | EPI637419 | EPI769643 |  | EPI572957 | EPI1055694 | EPI497541 | EPI583385 | EPI1093099 | EPI681002 |
| EPI620025 | EPI627927 | EPI1101380 | EPI656464 | EPI627876 | EPI613772 | EPI656097 | EPI560402 |  | EPI845705 | EPI1055686 | EPI497516 | EPI583503 | EPI1093105 | EPI492275 |
| EPI620053 | EPI628031 | EPI1101404 | EPI628630 | EPI627812 | EPI515797 | EPI637564 | EPI1090217 |  | EPI572976 | EPI1055670 | EPI1093154 | EPI583699 | EPI1093117 | EPI681031 |
| EPI620039 | EPI628175 | EPI1101412 | EPI627383 | EPI627820 | EPI457431 | EPI457736 | EPI895544 |  | EPI572977 | EPI1055724 | EPI1093160 | EPI583326 | EPI1093147 | EPI1092294 |
| EPI620153 | EPI628167 | EPI1101396 | EPI628622 | EPI627884 | EPI447717 | EPI637718 | EPI453608 |  | EPI532313 | EPI594387 | EPI838049 | EPI843855 | EPI1093087 | EPI583499 |
| EPI620032 | EPI628215 | EPI1101364 | EPI628694 | EPI627892 | EPI620138 | EPI1090800 | EPI469536 |  | EPI680619 | EPI599668 | EPI838050 | EPI583355 | EPI1093189 | EPI838202 |
| EPI620060 | EPI615184 | EPI1101388 | EPI656480 | EPI627900 | EPI447633 | EPI637475 | EPI451264 |  | EPI680618 | EPI600046 | EPI838071 | EPI583650 | EPI838143 | EPI843882 |
| EPI545802 | EPI663446 | EPI628077 | EPI627191 | EPI541773 | EPI637993 | EPI515791 | EPI447819 |  | EPI680620 | EPI680687 | EPI1091828 | EPI583692 | EPI838141 | EPI654474 |
| EPI545795 | EPI457740 | EPI628053 | EPI656312 | EPI1055420 | EPI637888 | EPI498101 | EPI585986 |  | EPI532312 | EPI532329 | EPI590910 | EPI491020 | EPI654411 | EPI654492 |
| EPI663466 | EPI447787 | EPI627398 | EPI1057966 | EPI626981 | EPI637930 | EPI457441 | EPI447767 |  | EPI680617 | EPI680688 | EPI1092182 | EPI583657 | EPI690480 | EPI838180 |
| EPI615183 | EPI1090799 | EPI628365 | EPI627215 | EPI1090732 | EPI638007 | EPI637837 | EPI451327 |  | EPI594386 | EPI680689 | EPI838069 | EPI583566 | EPI1092115 | EPI1091952 |
| EPI663434 | EPI457852 | EPI528375 | EPI1057934 | EPI566072 | EPI582355 | EPI497897 | EPI615188 |  | EPI569469 | EPI532328 | EPI1091876 | EPI583587 | EPI1092253 | EPI1091940 |
| EPI490979 | EPI447870 | EPI528367 | EPI626999 | EPI1090236 | EPI620131 | EPI637571 | EPI497869 |  | EPI572962 | EPI680686 | EPI1092332 | EPI581659 | EPI1092247 | EPI1091958 |
| EPI490971 | EPI467307 | EPI528351 | EPI627047 | EPI833704 | EPI447640 | EPI446454 | EPI447782 |  | EPI572964 | EPI654454 | EPI590909 | EPI838112 | EPI1092295 | EPI838204 |
| EPI503506 | EPI576574 | EPI1090354 | EPI656456 | EPI1101451 | EPI531121 | EPI636959 | EPI447938 |  | EPI572975 | EPI654195 | EPI654488 | EPI1092101 | EPI838129 | EPI846035 |
| EPI460775 | EPI457884 | EPI531794 | EPI1090363 | EPI627165 | EPI445913 | EPI613836 | EPI443038 |  | EPI594414 | EPI461702 | EPI654476 | EPI654455 | EPI600078 | EPI1055458 |
| EPI620104 | EPI576573 | EPI1090808 | EPI1040469 | EPI628652 | EPI613838 | EPI655937 | EPI441001 |  | EPI594471 | EPI466280 | EPI654205 | EPI843857 | EPI599498 | EPI764631 |
| EPI498087 | EPI464514 | EPI1090450 | EPI1090291 | EPI628612 | EPI497866 | EPI637865 | EPI447725 |  | EPI596657 | EPI466264 | EPI690484 | EPI1092119 | EPI600057 | EPI764635 |
| EPI620142 | EPI628079 | EPI593379 | EPI1090395 | EPI628580 | EPI637343 | EPI638068 | EPI533246 |  | EPI594429 | EPI466352 | EPI838059 | EPI1093223 | EPI599540 | EPI961394 |
| EPI620118 | EPI656385 | EPI592630 | EPI1013255 | EPI628572 | EPI637832 | EPI637412 | EPI627139 |  | EPI594436 | EPI572922 | EPI1092152 | EPI654217 | EPI600106 | EPI838194 |
| EPI620192 | EPI628391 | EPI592506 | EPI1013239 | EPI628588 | EPI620188 | EPI596857 | EPI627147 |  | EPI466211 | EPI572933 | EPI838057 | EPI654449 | EPI599449 | EPI838191 |
| EPI457765 | EPI509119 | EPI592242 | EPI1090387 | EPI627077 | EPI497830 | EPI637099 | EPI628770 |  | EPI680664 | EPI497538 | EPI1092290 | EPI470540 | EPI600092 | EPI594385 |
| EPI619985 | EPI628151 | EPI592574 | EPI1013215 | EPI627085 | EPI637706 | EPI596957 | EPI628818 |  | EPI652994 | EPI497519 | EPI1092530 | EPI470517 | EPI600071 | EPI599785 |
| EPI457709 | EPI628271 | EPI592458 | EPI1022615 | EPI628540 | EPI637909 | EPI613822 | EPI683112 |  | EPI652993 | EPI497522 | EPI1092422 | EPI470521 | EPI599477 | EPI594378 |
| EPI692160 | EPI628207 | EPI592279 | EPI1022631 | EPI627005 | EPI638084 | EPI637348 | EPI683113 |  | EPI652992 | EPI599466 | EPI577063 | EPI470524 | EPI600085 | EPI838173 |
| EPI457781 | EPI628159 | EPI593144 | EPI1013263 | EPI627093 | EPI637358 | EPI637327 | EPI1090569 |  | EPI652996 | EPI600067 | EPI577076 | EPI470525 | EPI599819 | EPI764646 |
| EPI442721 | EPI1090207 | EPI592482 | EPI926831 | EPI628740 | EPI530825 | EPI637015 | EPI764615 |  | EPI653007 | EPI600060 | EPI1076639 | EPI583496 | EPI557786 | EPI583478 |
| EPI487902 | EPI627376 | EPI592513 | EPI926823 | EPI628660 | EPI613786 | EPI637001 | EPI590930 |  | EPI654188 | EPI599815 | EPI1092464 | EPI470520 | EPI599526 | EPI594456 |
| EPI457621 | EPI628223 | EPI592590 | EPI1074592 | EPI683244 | EPI637804 | EPI509104 | EPI628018 |  | EPI681046 | EPI599473 | EPI691883 | EPI470511 | EPI599505 | EPI594449 |
| EPI508781 | EPI628199 | EPI593172 | EPI1090403 | EPI683245 | EPI443644 | EPI637711 | EPI628402 |  | EPI681045 | EPI600095 | EPI497525 | EPI470512 | EPI599435 | EPI594463 |
| EPI627609 | EPI457724 | EPI592272 | EPI919613 | EPI628772 | EPI637986 | EPI637092 | EPI764527 |  | EPI680663 | EPI600109 | EPI654367 | EPI470518 | EPI600064 | EPI594470 |
| EPI487905 | EPI1090191 | EPI593165 | EPI1090299 | EPI628820 | EPI447830 | EPI636760 | EPI764588 |  | EPI681052 | EPI599494 | EPI729792 | EPI470509 | EPI692275 | EPI681039 |
| EPI532302 | EPI627520 | EPI1090474 | EPI1074600 | EPI627157 | EPI515448 | EPI581903 | EPI764531 |  | EPI681053 | EPI599543 | EPI1091840 | EPI470537 | EPI599470 | EPI594413 |
| EPI443651 | EPI1090813 | EPI628349 | EPI1013167 | EPI627133 | EPI638091 | EPI637809 | EPI764538 |  | EPI654477 | EPI599480 | EPI1091894 | EPI470539 | EPI599554 | EPI764642 |
| EPI532303 | EPI509100 | EPI535128 | EPI1013183 | EPI621113 | EPI637336 | EPI447868 | EPI764539 |  | EPI654495 | EPI600102 | EPI1091900 | EPI470533 | EPI599533 | EPI1092192 |
| EPI447626 | EPI509105 | EPI628133 | EPI1013207 | EPI621114 | EPI576577 | EPI506228 | EPI764574 |  | EPI492284 | EPI599501 | EPI1092482 | EPI470534 | EPI599442 | EPI1092990 |
| EPI457685 | EPI628399 | EPI627590 | EPI1013199 | EPI628620 | EPI637421 | EPI637398 | EPI764604 |  | EPI492277 | EPI599459 | EPI1092380 | EPI470535 | EPI599561 | EPI1093008 |
| EPI457693 | EPI656186 | EPI628245 | EPI1013175 | EPI627381 | EPI457614 | EPI663489 | EPI764533 |  | EPI465410 | EPI599522 | EPI1092374 | EPI470536 | EPI599547 | EPI1093002 |
| EPI447628 | EPI531768 | EPI576617 | EPI1013191 | EPI656262 | EPI442722 | EPI509137 | EPI628250 |  | EPI573390 | EPI599536 | EPI1092404 | EPI470541 | EPI578046 | EPI1092996 |
| EPI498098 | EPI531776 | EPI628189 | EPI628702 | EPI627117 | EPI490972 | EPI637634 | EPI764610 |  | EPI691718 | EPI599402 | EPI838067 | EPI470538 | EPI599766 | EPI1093014 |
| EPI467313 | EPI531792 | EPI541771 | EPI628718 | EPI627229 | EPI637685 | EPI620033 | EPI592558 |  | EPI492291 | EPI600081 | EPI1092770 | EPI470527 | EPI594448 | EPI528396 |
| EPI460751 | EPI531784 | EPI628381 | EPI942228 | EPI656486 | EPI460776 | EPI637501 | EPI535149 |  | EPI691738 | EPI599452 | EPI1091762 | EPI470528 | EPI654255 | EPI528388 |
| EPI450526 | EPI656225 | EPI628085 | EPI627199 | EPI656478 | EPI620067 | EPI457776 | EPI535181 |  | EPI492263 | EPI599395 | EPI1092584 | EPI470529 | EPI1098506 | EPI548391 |
| EPI497910 | EPI509089 | EPI628181 | EPI656304 | EPI628692 | EPI638077 | EPI620119 | EPI627571 |  | EPI492270 | EPI599424 | EPI1092590 | EPI470530 | EPI1055600 | EPI528380 |
| EPI457637 | EPI470344 | EPI578027 | EPI628766 | EPI656470 | EPI638014 | EPI620179 | EPI535117 |  | EPI529277 | EPI599445 | EPI1092686 | EPI470510 | EPI843874 | EPI466476 |
| EPI457773 | EPI470347 | EPI656192 | EPI628550 | EPI626997 | EPI457638 | EPI655945 | EPI627555 |  | EPI468355 | EPI600053 | EPI1092104 | EPI838088 | EPI838151 | EPI492282 |
| EPI447601 | EPI470345 | EPI531475 | EPI1101429 | EPI627213 | EPI583008 | EPI637543 | EPI627802 |  | EPI569599 | EPI599431 | EPI1092308 | EPI654485 | EPI578070 | EPI492289 |
| EPI467305 | EPI470350 | EPI628429 | EPI1101197 | EPI656454 | EPI637470 | EPI637064 | EPI627882 |  | EPI473790 | EPI530456 | EPI1092716 | EPI1061404 | EPI599575 | EPI1086313 |
| EPI576623 | EPI470348 | EPI627438 | EPI1101205 | EPI1057932 | EPI637902 | EPI581569 | EPI627898 |  | EPI680622 | EPI599550 | EPI1093226 | EPI1062120 | EPI530489 | EPI1091706 |
| EPI576547 | EPI470346 | EPI566073 | EPI628678 | EPI627045 | EPI620095 | EPI1090432 | EPI627818 |  | EPI569600 | EPI599409 | EPI572886 | EPI470531 | EPI599637 | EPI1055698 |
| EPI535131 | EPI468781 | EPI627134 | EPI628790 | EPI656430 | EPI637965 | EPI663492 | EPI627890 |  | EPI680623 | EPI599438 | EPI572897 | EPI470577 | EPI654416 | EPI1091868 |
| EPI627593 | EPI470349 | EPI621126 | EPI1101437 | EPI627469 | EPI613779 | EPI637508 | EPI628306 |  | EPI691843 | EPI600088 | EPI577334 | EPI470572 | EPI530459 | EPI1076638 |
| EPI627864 | EPI545804 | EPI621133 | EPI628670 | EPI628636 | EPI1090798 | EPI581765 | EPI592292 |  | EPI691844 | EPI599417 | EPI577319 | EPI470576 | EPI530477 | EPI1092360 |
| EPI756011 | EPI663467 | EPI1090282 | EPI628782 | EPI628628 | EPI457846 | EPI470076 | EPI592220 |  | EPI532315 | EPI599734 | EPI577321 | EPI470573 | EPI530465 | EPI1092816 |
| EPI1055432 | EPI615178 | EPI580368 | EPI683158 | EPI656374 | EPI620074 | EPI637578 | EPI592255 |  | EPI599556 | EPI599529 | EPI487858 | EPI1209325 | EPI602359 | EPI1092834 |
| EPI628184 | EPI615181 | EPI628757 | EPI683154 | EPI628684 | EPI509106 | EPI470075 | EPI592155 |  | EPI351707 | EPI599388 | EPI487853 | EPI691992 | EPI530483 | EPI1092840 |
| EPI576542 | EPI663451 | EPI593467 | EPI1102435 | EPI656350 | EPI637172 | EPI637276 | EPI592481 |  | EPI600129 | EPI599515 | EPI487857 | EPI691993 | EPI530513 | EPI1092894 |
| EPI656195 | EPI545797 | EPI593495 | EPI1102475 | EPI656422 | EPI619987 | EPI637683 | EPI592169 |  | EPI691719 | EPI599487 | EPI487854 | EPI470506 | EPI602285 | EPI1092030 |
| EPI578029 | EPI663450 | EPI593403 | EPI942236 | EPI656446 | EPI638028 | EPI447827 | EPI592603 |  | EPI497517 | EPI599508 | EPI1103543 | EPI470507 | EPI530471 | EPI1092036 |
| EPI628432 | EPI464590 | EPI593546 | EPI1101029 | EPI1090556 | EPI637271 | EPI627795 | EPI593157 |  | EPI497524 | EPI569588 | EPI553353 | EPI470532 | EPI602366 | EPI1098441 |
| EPI628248 | EPI615175 | EPI593539 | EPI866559 | EPI628732 | EPI637463 | EPI637334 | EPI592551 |  | EPI596710 | EPI557661 | EPI577339 | EPI470522 | EPI530453 | EPI1091736 |
| EPI531468 | EPI576575 | EPI566105 | EPI1101549 | EPI628724 | EPI457622 | EPI637313 | EPI592262 |  | EPI497497 | EPI641383 | EPI591178 | EPI470523 | EPI599568 | EPI1091742 |
| EPI1090477 | EPI656115 | EPI627909 | EPI866551 | EPI1090540 | EPI637379 | EPI636841 | EPI592622 |  | EPI572965 | EPI845706 | EPI591180 | EPI654229 | EPI602292 | EPI681180 |
| EPI628192 | EPI583258 | EPI627829 | EPI971250 | EPI627493 | EPI637692 | EPI637620 | EPI592589 |  | EPI497540 | EPI568781 | EPI680767 | EPI538913 | EPI530519 | EPI681171 |
| EPI628136 | EPI627935 | EPI627917 | EPI1101477 | EPI627237 | EPI515455 | EPI636767 | EPI592227 |  | EPI837965 | EPI764809 | EPI591184 | EPI654190 | EPI654481 | EPI681168 |
| EPI628088 | EPI655981 | EPI1090594 | EPI1104021 | EPI627301 | EPI507148 | EPI655881 | EPI592241 |  | EPI590911 | EPI572924 | EPI591190 | EPI654393 | EPI484486 | EPI681172 |
| EPI628352 | EPI1090820 | EPI1090602 | EPI917063 | EPI627277 | EPI637874 | EPI637120 | EPI592126 |  | EPI590912 | EPI572926 | EPI838076 | EPI654315 | EPI484494 | EPI654400 |
| EPI628384 | EPI729465 | EPI566065 | EPI1090507 | EPI627285 | EPI637867 | EPI637907 | EPI592629 |  | EPI837966 | EPI572937 | EPI680728 | EPI490988 | EPI317184 | EPI654284 |
| EPI1090629 | EPI447660 | EPI627318 | EPI1090515 | EPI628804 | EPI497815 | EPI581759 | EPI566118 |  | EPI1092333 | EPI468350 | EPI680726 | EPI490996 | EPI572788 | EPI654266 |
| EPI583137 | EPI509122 | EPI627502 | EPI866543 | EPI627453 | EPI620063 | EPI637991 | EPI628058 |  | EPI590913 | EPI680670 | EPI680725 | EPI491004 | EPI764795 | EPI654371 |
| EPI583144 | EPI627863 | EPI566049 | EPI1101541 | EPI941046 | EPI620121 | EPI447786 | EPI627834 |  | EPI654281 | EPI492269 | EPI680727 | EPI583573 | EPI764799 | EPI654241 |
| EPI583095 | EPI1055423 | EPI627326 | EPI1100837 | EPI627253 | EPI582906 | EPI448939 | EPI627443 |  | EPI1092237 | EPI492262 | EPI654193 | EPI583643 | EPI568788 | EPI654421 |
| EPI583151 | EPI756016 | EPI566113 | EPI1100821 | EPI627221 | EPI637958 | EPI637185 | EPI627411 |  | EPI1092183 | EPI680681 | EPI577132 | EPI583734 | EPI568613 | EPI654439 |
| EPI627569 | EPI1055431 | EPI627342 | EPI1100501 | EPI627101 | EPI638000 | EPI637377 | EPI627403 |  | EPI837974 | EPI680683 | EPI530502 | EPI583545 | EPI635476 | EPI654415 |
| EPI583122 | EPI464515 | EPI627350 | EPI1100509 | EPI627293 | EPI443572 | EPI581720 | EPI528292 |  | EPI837985 | EPI450829 | EPI576921 | EPI583333 | EPI568614 | EPI654208 |
| EPI583088 | EPI447681 | EPI566057 | EPI1102555 | EPI769566 | EPI638035 | EPI447750 | EPI594287 |  | EPI654206 | EPI557660 | EPI530508 | EPI583880 | EPI466309 | EPI654235 |
| EPI583166 | EPI467315 | EPI627078 | EPI1102931 | EPI769574 | EPI637414 | EPI637461 | EPI594217 |  | EPI690486 | EPI532330 | EPI654177 | EPI583559 | EPI351715 | EPI573387 |
| EPI477410 | EPI596849 | EPI627086 | EPI1090459 | EPI627309 | EPI582984 | EPI613815 | EPI594245 |  | EPI837973 | EPI680690 | EPI591192 | EPI583608 | EPI466357 | EPI683107 |
| EPI477450 | EPI597246 | EPI628573 | EPI1102539 | EPI566127 | EPI441000 | EPI451326 | EPI628138 |  | EPI837975 | EPI838009 | EPI838062 | EPI583671 | EPI466389 | EPI681173 |
| EPI582340 | EPI597312 | EPI628581 | EPI1090443 | EPI566080 | EPI620156 | EPI636885 | EPI628234 |  | EPI1092057 | EPI838005 | EPI838066 | EPI654240 | EPI557779 | EPI681177 |
| EPI583108 | EPI627959 | EPI628589 | EPI1101421 | EPI627461 | EPI613817 | EPI620129 | EPI535189 |  | EPI1091991 | EPI844498 | EPI838061 | EPI583468 | EPI654395 | EPI681174 |
| EPI628256 | EPI654042 | EPI628741 | EPI1101381 | EPI576616 | EPI637972 | EPI637739 | EPI594350 |  | EPI1092045 | EPI654409 | EPI1091954 | EPI583425 | EPI681152 | EPI681182 |
| EPI583102 | EPI655933 | EPI627166 | EPI887698 | EPI535127 | EPI620202 | EPI581919 | EPI594266 |  | EPI1091985 | EPI1092190 | EPI1091942 | EPI583447 | EPI596693 | EPI681184 |
| EPI583115 | EPI628127 | EPI627006 | EPI1101365 | EPI627589 | EPI637895 | EPI637225 | EPI594147 |  | EPI1092015 | EPI654304 | EPI1091960 | EPI583706 | EPI568792 | EPI654353 |
| EPI582937 | EPI627768 | EPI627094 | EPI1055414 | EPI1090636 | EPI637286 | EPI447764 | EPI583936 |  | EPI1092027 | EPI837995 | EPI1103521 | EPI583664 | EPI764514 | EPI654347 |
| EPI583044 | EPI627791 | EPI628813 | EPI740736 | EPI1090180 | EPI638021 | EPI620093 | EPI628034 |  | EPI942887 | EPI583445 | EPI1103529 | EPI838080 | EPI838127 | EPI654359 |
| EPI583130 | EPI515876 | EPI683200 | EPI1090739 | EPI628356 | EPI638063 | EPI447638 | EPI583922 |  | EPI1093257 | EPI838031 | EPI1092116 | EPI838079 | EPI591966 | EPI652959 |
| EPI692629 | EPI655893 | EPI683201 | EPI1103392 | EPI535135 | EPI637699 | EPI637370 | EPI594343 |  | EPI1092243 | EPI1093060 | EPI680522 | EPI583678 | EPI596686 | EPI652971 |
| EPI628024 | EPI628447 | EPI552396 | EPI1101405 | EPI535143 | EPI447788 | EPI613777 | EPI594203 |  | EPI1093053 | EPI1093066 | EPI838238 | EPI838116 | EPI1155617 | EPI652957 |
| EPI628408 | EPI628511 | EPI627182 | EPI1101389 | EPI535159 | EPI439496 | EPI581835 | EPI594210 |  | EPI590914 | EPI1092184 | EPI858162 | EPI583594 | EPI654204 | EPI652963 |
| EPI692627 | EPI628103 | EPI1090610 | EPI1101397 | EPI628324 | EPI447668 | EPI1090440 | EPI594189 |  | EPI1086306 | EPI838027 | EPI623617 | EPI583552 | EPI596722 | EPI652961 |
| EPI692623 | EPI655957 | EPI627158 | EPI1103901 | EPI628316 | EPI637818 | EPI636938 | EPI594329 |  | EPI1006752 | EPI654245 | EPI844510 | EPI583622 | EPI1155634 | EPI652965 |
| EPI692615 | EPI628463 | EPI1090498 | EPI1101373 | EPI628332 | EPI637741 | EPI637522 | EPI594259 |  | EPI653008 | EPI654282 | EPI1092158 | EPI654299 | EPI680980 | EPI652960 |
| EPI692620 | EPI655901 | EPI1090730 | EPI1101413 | EPI576825 | EPI440678 | EPI636966 | EPI592183 |  | EPI1092087 | EPI654438 | EPI1093052 | EPI654247 | EPI1092547 | EPI652970 |
| EPI692622 | EPI628503 | EPI627510 | EPI1101493 | EPI576787 | EPI637727 | EPI628123 | EPI594273 |  | EPI1055482 | EPI654222 | EPI1092260 | EPI654196 | EPI532318 | EPI652962 |
| EPI583072 | EPI506227 | EPI1090538 | EPI961185 | EPI576817 | EPI638049 | EPI509134 | EPI594308 |  | EPI1055701 | EPI838028 | EPI1092194 | EPI654424 | EPI680936 | EPI652969 |
| EPI582983 | EPI628487 | EPI628621 | EPI1090491 | EPI576826 | EPI637322 | EPI636716 | EPI594224 |  | EPI1092987 | EPI1092154 | EPI1092128 | EPI838095 | EPI680946 | EPI652958 |
| EPI582354 | EPI596928 | EPI1057965 | EPI1101485 | EPI576820 | EPI637734 | EPI627787 | EPI583950 |  | EPI1091739 | EPI654460 | EPI1092110 | EPI560122 | EPI450834 | EPI652966 |
| EPI582944 | EPI581834 | EPI656479 | EPI979571 | EPI576821 | EPI447872 | EPI581695 | EPI594154 |  | EPI1091745 | EPI844499 | EPI1092266 | EPI441370 | EPI680955 | EPI1092798 |
| EPI582959 | EPI582320 | EPI627110 | EPI1102029 | EPI576785 | EPI457870 | EPI636674 | EPI594280 |  | EPI1092897 | EPI1092304 | EPI557733 | EPI441098 | EPI680935 | EPI1091724 |
| EPI583014 | EPI597016 | EPI656343 | EPI1101469 | EPI576786 | EPI497919 | EPI443573 | EPI535165 |  | EPI1091871 | EPI1092310 | EPI1092566 | EPI529247 | EPI680956 | EPI1091778 |
| EPI582966 | EPI597029 | EPI627262 | EPI1100437 | EPI576819 | EPI620100 | EPI637697 | EPI592323 |  | EPI1098456 | EPI583376 | EPI1092608 | EPI451171 | EPI680957 | EPI1092378 |
| EPI582361 | EPI597204 | EPI656319 | EPI1100469 | EPI576824 | EPI637944 | EPI637746 | EPI628338 |  | EPI1092369 | EPI583529 | EPI1092476 | EPI583510 | EPI468353 | EPI1092480 |
| EPI583007 | EPI597319 | EPI628629 | EPI1100477 | EPI576840 | EPI637797 | EPI581968 | EPI592316 |  | EPI1092675 | EPI583383 | EPI1092458 | EPI583363 | EPI470054 | EPI1092720 |
| EPI583021 | EPI628471 | EPI628637 | EPI888010 | EPI576865 | EPI637916 | EPI637921 | EPI627579 |  | EPI1092741 | EPI583473 | EPI1092560 | EPI583601 | EPI680986 | EPI1092702 |
| EPI582347 | EPI583251 | EPI1057925 | EPI888018 | EPI628284 | EPI637407 | EPI637970 | EPI528332 |  | EPI1092747 | EPI654325 | EPI1092488 | EPI583580 | EPI532319 | EPI1055513 |
| EPI628360 | EPI655965 | EPI628693 | EPI887986 | EPI628276 | EPI637449 | EPI613829 | EPI594168 |  | EPI1093041 | EPI838021 | EPI1092614 | EPI654253 | EPI1092943 | EPI1090077 |
| EPI628312 | EPI581746 | EPI627046 | EPI888042 | EPI628260 | EPI613831 | EPI637260 | EPI594301 |  | EPI641382 | EPI654320 | EPI1098522 | EPI838115 | EPI942115 | EPI1092120 |
| EPI592230 | EPI581705 | EPI656455 | EPI888050 | EPI628308 | EPI439505 | EPI637949 | EPI594182 |  | EPI1092891 | EPI583690 | EPI736584 | EPI838086 | EPI492267 | EPI1092246 |
| EPI593160 | EPI581740 | EPI627014 | EPI888058 | EPI627549 | EPI637393 | EPI596813 | EPI594231 |  | EPI1098458 | EPI583353 | EPI1091822 | EPI838083 | EPI680948 | EPI843885 |
| EPI592446 | EPI581967 | EPI627038 | EPI1102579 | EPI535175 | EPI582945 | EPI637676 | EPI627539 |  | EPI1092801 | EPI583416 | EPI1092752 | EPI529248 | EPI680947 | EPI846043 |
| EPI841962 | EPI581993 | EPI627278 | EPI1101949 | EPI628372 | EPI490964 | EPI509117 | EPI594322 |  | EPI1092855 | EPI583459 | EPI577848 | EPI416216 | EPI573381 | EPI843880 |
| EPI593645 | EPI581682 | EPI627286 | EPI1101957 | EPI591534 | EPI638105 | EPI637529 | EPI594196 |  | EPI1092831 | EPI654258 | EPI844503 | EPI515823 | EPI492260 | EPI1092630 |
| EPI592439 | EPI583313 | EPI627254 | EPI888002 | EPI591447 | EPI637713 | EPI439506 | EPI583943 |  | EPI1092867 | EPI469270 | EPI1093016 | EPI583799 | EPI529238 | EPI1092204 |
| EPI592101 | EPI596909 | EPI627454 | EPI888074 | EPI591321 | EPI585981 | EPI655929 | EPI528340 |  | EPI1092861 | EPI469272 | EPI1093010 | EPI583792 | EPI583808 | EPI1092210 |
| EPI592661 | EPI581733 | EPI941044 | EPI1102347 | EPI577535 | EPI447817 | EPI568650 | EPI583949 |  | EPI1092849 | EPI469275 | EPI1092998 | EPI583862 | EPI470052 | EPI1092222 |
| EPI593188 | EPI581758 | EPI627494 | EPI1102411 | EPI577380 | EPI627602 | EPI443027 | EPI594294 |  | EPI837977 | EPI572910 | EPI1092992 | EPI592011 | EPI466453 | EPI1092690 |
| EPI627145 | EPI581604 | EPI656431 | EPI888026 | EPI577385 | EPI448937 | EPI581975 | EPI628298 |  | EPI1091733 | EPI680716 | EPI1093004 | EPI592004 | EPI1093045 | EPI1093128 |
| EPI627153 | EPI581777 | EPI627214 | EPI971410 | EPI576609 | EPI582967 | EPI509505 | EPI627387 |  | EPI1091841 | EPI466212 | EPI683237 | EPI680843 | EPI974025 | EPI1093248 |
| EPI592639 | EPI581827 | EPI627382 | EPI971386 | EPI628228 | EPI446980 | EPI637704 | EPI594357 |  | EPI844488 | EPI680696 | EPI683239 | EPI680844 | EPI1055443 | EPI1103520 |
| EPI592136 | EPI581770 | EPI627470 | EPI971394 | EPI591552 | EPI637776 | EPI581727 | EPI628290 |  | EPI837978 | EPI594430 | EPI1092794 | EPI466371 | EPI1155626 | EPI1093176 |
| EPI592158 | EPI581840 | EPI1057933 | EPI1102899 | EPI591553 | EPI638056 | EPI447652 | EPI594315 |  | EPI594358 | EPI594437 | EPI1092818 | EPI466283 | EPI680974 | EPI1093182 |
| EPI592129 | EPI581924 | EPI626998 | EPI1100781 | EPI628068 | EPI620081 | EPI637515 | EPI583915 |  | EPI583852 | EPI572738 | EPI1092806 | EPI529246 | EPI506279 | EPI681028 |
| EPI592645 | EPI578147 | EPI656471 | EPI1104061 | EPI577790 | EPI613793 | EPI637788 | EPI627563 |  | EPI594407 | EPI572745 | EPI1092836 | EPI569639 | EPI680949 | EPI683105 |
| EPI592453 | EPI597560 | EPI628725 | EPI888034 | EPI591391 | EPI583022 | EPI636994 | EPI628258 |  | EPI837981 | EPI572936 | EPI1092842 | EPI838119 | EPI680950 | EPI594364 |
| EPI593652 | EPI597326 | EPI627230 | EPI971402 | EPI591320 | EPI477403 | EPI620150 | EPI627531 |  | EPI837980 | EPI1103541 | EPI1092680 | EPI838098 | EPI583864 | EPI681181 |
| EPI592476 | EPI597305 | EPI656487 | EPI971322 | EPI591327 | EPI637456 | EPI620168 | EPI535197 |  | EPI1086308 | EPI1103542 | EPI1093076 | EPI654223 | EPI591959 | EPI680997 |
| EPI628232 | EPI597267 | EPI628685 | EPI971418 | EPI591392 | EPI620107 | EPI637440 | EPI594175 |  | EPI837984 | EPI1103540 | EPI1093046 | EPI654259 | EPI583794 | EPI838193 |
| EPI628376 | EPI597333 | EPI656423 | EPI971258 | EPI591243 | EPI638042 | EPI457616 | EPI594336 |  | EPI837988 | EPI1103538 | EPI530490 | EPI654479 | EPI583801 | EPI1091760 |
| EPI576610 | EPI578135 | EPI627118 | EPI1100957 | EPI591239 | EPI509099 | EPI620040 | EPI583929 |  | EPI599570 | EPI1103539 | EPI568433 | EPI1098440 | EPI1093255 | EPI1092582 |
| EPI628072 | EPI597340 | EPI656351 | EPI1101973 | EPI591443 | EPI620195 | EPI656119 | EPI594140 |  | EPI1092681 | EPI594415 | EPI1092326 | EPI1209317 | EPI652901 | EPI1092588 |
| EPI627425 | EPI597361 | EPI583164 | EPI971298 | EPI591402 | EPI457862 | EPI447736 | EPI627595 |  | EPI1093077 | EPI594451 | EPI1092518 | EPI1209333 | EPI652910 | EPI1092456 |
| EPI627433 | EPI596755 | EPI628413 | EPI971474 | EPI591235 | EPI637755 | EPI638026 | EPI594238 |  | EPI530467 | EPI594458 | EPI577889 | EPI583531 | EPI652905 | EPI1103528 |
| EPI596913 | EPI597368 | EPI1102306 | EPI971482 | EPI591319 | EPI637315 | EPI636805 | EPI583948 |  | EPI530485 | EPI568783 | EPI598340 | EPI583475 | EPI652903 | EPI681017 |
| EPI597481 | EPI597354 | EPI627030 | EPI1101805 | EPI703993 | EPI637769 | EPI583321 | EPI594252 |  | EPI602354 | EPI692131 | EPI530484 | EPI838105 | EPI652898 | EPI681021 |
| EPI597393 | EPI596832 | EPI576620 | EPI1102251 | EPI591240 | EPI457750 | EPI637690 | EPI627523 |  | EPI1093161 | EPI692136 | EPI1092338 | EPI838090 | EPI652906 | EPI838198 |
| EPI596947 | EPI596826 | EPI895627 | EPI1103997 | EPI591451 | EPI637951 | EPI638089 | EPI628042 |  | EPI1093245 | EPI529271 | EPI1092344 | EPI654404 | EPI652895 | EPI838190 |
| EPI628280 | EPI597253 | EPI895634 | EPI1103957 | EPI531802 | EPI637762 | EPI637008 | EPI627611 |  | EPI1093233 | EPI844495 | EPI838058 | EPI351701 | EPI652897 | EPI681040 |
| EPI1090469 | EPI596768 | EPI895625 | EPI1104005 | EPI729450 | EPI620177 | EPI637232 | EPI542097 |  | EPI1091877 | EPI844497 | EPI1092236 | EPI572736 | EPI652909 | EPI1092552 |
| EPI628288 | EPI597274 | EPI895619 | EPI1090667 | EPI729458 | EPI620088 | EPI637928 | EPI594161 |  | EPI583810 | EPI591986 | EPI1093244 | EPI572743 | EPI652911 | EPI681023 |
| EPI627553 | EPI597211 | EPI895641 | EPI582936 | EPI1090196 | EPI467306 | EPI637029 | EPI1018252 |  | EPI583824 | EPI680714 | EPI1093232 | EPI466215 | EPI652899 | EPI681024 |
| EPI533259 | EPI597218 | EPI895648 | EPI583043 | EPI627037 | EPI447759 | EPI636731 | EPI1101681 |  | EPI1092735 | EPI680672 | EPI1093238 | EPI557804 | EPI652900 | EPI681025 |
| EPI627816 | EPI596798 | EPI895630 | EPI628262 | EPI628596 | EPI637386 | EPI637606 | EPI1102951 |  | EPI583845 | EPI680674 | EPI942208 | EPI594417 | EPI652902 | EPI681026 |
| EPI627880 | EPI596775 | EPI627126 | EPI628022 | EPI1090228 | EPI457742 | EPI531119 | EPI1102943 |  | EPI583838 | EPI680673 | EPI1076693 | EPI572853 | EPI942117 | EPI681019 |
| EPI1090485 | EPI597176 | EPI1101204 | EPI628406 | EPI1090244 | EPI613824 | EPI638005 | EPI1102959 |  | EPI583817 | EPI680675 | EPI1076685 | EPI594432 | EPI1093207 | EPI681035 |
| EPI533243 | EPI597197 | EPI1101196 | EPI628310 | EPI591252 | EPI583015 | EPI637391 | EPI613787 |  | EPI837983 | EPI594472 | EPI1076669 | EPI594439 | EPI942893 | EPI681022 |
| EPI533250 | EPI578128 | EPI1101428 | EPI593152 | EPI591401 | EPI638098 | EPI576600 | EPI613794 |  | EPI1091847 | EPI596658 | EPI1076677 | EPI583340 | EPI1092241 | EPI681036 |
| EPI682920 | EPI597190 | EPI628781 | EPI592624 | EPI591241 | EPI457766 | EPI637306 | EPI580295 |  | EPI1092051 | EPI654189 | EPI1092908 | EPI654184 | EPI1103563 | EPI764650 |
| EPI654146 | EPI597441 | EPI628677 | EPI592287 | EPI591259 | EPI457726 | EPI581841 | EPI580287 |  | EPI1092771 | EPI680680 | EPI1092914 | EPI654334 | EPI767544 | EPI654468 |
| EPI1102973 | EPI581944 | EPI628789 | EPI592222 | EPI591257 | EPI470594 | EPI497921 | EPI580305 |  | EPI942264 | EPI1092202 | EPI1067641 | EPI654340 | EPI767558 | EPI681178 |
| EPI627033 | EPI578173 | EPI628669 | EPI592164 | EPI591376 | EPI440686 | EPI636850 | EPI580321 |  | EPI1092573 | EPI1092394 | EPI1091906 | EPI529249 | EPI466461 | EPI838201 |
| EPI592561 | EPI581909 | EPI1101436 | EPI627806 | EPI591251 | EPI637350 | EPI636813 | EPI656252 |  | EPI1093023 | EPI1103226 | EPI1091912 | EPI351703 | EPI596729 | EPI942298 |
| EPI592052 | EPI581902 | EPI628733 | EPI627822 | EPI591238 | EPI637860 | EPI620054 | EPI566666 |  | EPI1092921 | EPI1093210 | EPI652793 | EPI600132 | EPI466429 | EPI681175 |
| EPI592326 | EPI581951 | EPI628565 | EPI592250 | EPI591383 | EPI637278 | EPI636871 | EPI580271 |  | EPI1067639 | EPI1091938 | EPI1091966 | EPI466419 | EPI557778 | EPI1093194 |
| EPI592080 | EPI578180 | EPI628661 | EPI592475 | EPI591377 | EPI452261 | EPI613791 | EPI580279 |  | EPI583831 | EPI492276 | EPI652797 | EPI416217 | EPI466445 | EPI680520 |
| EPI592396 | EPI583293 | EPI628765 | EPI592121 | EPI591381 | EPI637477 | EPI637641 | EPI580359 |  | EPI1055445 | EPI680671 | EPI1092470 | EPI416218 | EPI515808 | EPI838243 |
| EPI592024 | EPI581937 | EPI628549 | EPI592553 | EPI591389 | EPI637329 | EPI637613 | EPI628706 |  | EPI974019 | EPI548393 | EPI1092434 | EPI466387 | EPI532316 | EPI1093206 |
| EPI592403 | EPI581881 | EPI626982 | EPI592215 | EPI591265 | EPI620114 | EPI638082 | EPI566086 |  | EPI1055453 | EPI528384 | EPI1092368 | EPI461705 | EPI680953 | EPI681183 |
| EPI592038 | EPI583320 | EPI1090642 | EPI592598 | EPI591244 | EPI582348 | EPI627724 | EPI580351 |  | EPI654465 | EPI528400 | EPI1092674 | EPI591930 | EPI680951 | EPI1092546 |
| EPI592333 | EPI578087 | EPI895623 | EPI592617 | EPI591388 | EPI637365 | EPI637433 | EPI509058 |  | EPI1092813 | EPI528392 | EPI1092740 | EPI466275 | EPI568795 | EPI1092228 |
| EPI592347 | EPI581930 | EPI592582 | EPI627870 | EPI535151 | EPI447844 | EPI581683 | EPI637679 |  | EPI1091955 | EPI838004 | EPI1092746 | EPI466259 | EPI466477 | EPI681179 |
| EPI592094 | EPI597375 | EPI895638 | EPI592584 | EPI533258 | EPI515463 | EPI637662 | EPI447690 |  | EPI1091943 | EPI583783 | EPI1093040 | EPI466347 | EPI515816 | EPI1092936 |
| EPI592361 | EPI581980 | EPI895636 | EPI592546 | EPI533242 | EPI637881 | EPI638047 | EPI477316 |  | EPI1091961 | EPI583739 | EPI1092032 | EPI466331 | EPI594370 | EPI1092966 |
| EPI592066 | EPI581960 | EPI1090530 | EPI592257 | EPI533249 | EPI637811 | EPI637963 | EPI509082 |  | EPI1091895 | EPI599780 | EPI1092038 | EPI466339 | EPI680965 | EPI529257 |
| EPI592389 | EPI596981 | EPI683197 | EPI592150 | EPI627597 | EPI628409 | EPI636878 | EPI457647 |  | EPI1091901 | EPI594373 | EPI1092062 | EPI466291 | EPI467577 | EPI692046 |
| EPI592108 | EPI581611 | EPI683203 | EPI592236 | EPI535199 | EPI498088 | EPI637057 | EPI441799 |  | EPI1091913 | EPI594380 | EPI1092722 | EPI466395 | EPI1055465 | EPI692051 |
| EPI592432 | EPI581651 | EPI627198 | EPI1090467 | EPI535191 | EPI637400 | EPI637141 | EPI490965 |  | EPI1092885 | EPI1092610 | EPI1092410 | EPI466251 | EPI681149 | EPI569573 |
| EPI592410 | EPI581631 | EPI656303 | EPI627551 | EPI627533 | EPI447766 | EPI583294 | EPI490973 |  | EPI1092033 | EPI1092556 | EPI1091948 | EPI470582 | EPI681161 | EPI596709 |
| EPI592354 | EPI581888 | EPI627246 | EPI628374 | EPI627836 | EPI447935 | EPI637298 | EPI503522 |  | EPI1092039 | EPI1093048 | EPI1092524 | EPI470588 | EPI764796 | EPI473797 |
| EPI592059 | EPI596819 | EPI683199 | EPI576608 | EPI627413 | EPI637923 | EPI620175 | EPI503523 |  | EPI1091751 | EPI1092682 | EPI1091996 | EPI568760 | EPI568617 | EPI1092684 |
| EPI592087 | EPI582327 | EPI683198 | EPI627423 | EPI627405 | EPI447738 | EPI637858 | EPI1090585 |  | EPI1098457 | EPI1093078 | EPI1091798 | EPI572851 | EPI515825 | EPI1092264 |
| EPI592382 | EPI592210 | EPI1090618 | EPI627431 | EPI627445 | EPI637372 | EPI447680 | EPI593248 |  | EPI1093239 | EPI1092334 | EPI1093172 | EPI594389 | EPI466469 | EPI1092126 |
| EPI592425 | EPI581974 | EPI683202 | EPI592171 | EPI628060 | EPI637484 | EPI581828 | EPI593282 |  | EPI557811 | EPI683148 | EPI1093082 | EPI466236 | EPI569550 | EPI1092102 |
| EPI592073 | EPI596968 | EPI917062 | EPI1090643 | EPI628140 | EPI515471 | EPI637627 | EPI628354 |  | EPI600066 | EPI681066 | EPI1093094 | EPI466403 | EPI764517 | EPI1092108 |
| EPI592368 | EPI596856 | EPI866550 | EPI593145 | EPI628236 | EPI637825 | EPI597554 | EPI535133 |  | EPI599472 | EPI681069 | EPI1092920 | EPI466379 | EPI1086314 | EPI1092216 |
| EPI592115 | EPI581895 | EPI961184 | EPI592560 | EPI528334 | EPI637180 | EPI636781 | EPI628314 |  | EPI600101 | EPI729795 | EPI1091738 | EPI466411 | EPI1093033 | EPI1092300 |
| EPI592172 | EPI581726 | EPI1103380 | EPI654144 | EPI528342 | EPI508927 | EPI597268 | EPI535157 |  | EPI599521 | EPI838036 | EPI1091744 | EPI465414 | EPI592020 | EPI1093224 |
| EPI592340 | EPI597498 | EPI1103900 | EPI628230 | EPI535167 | EPI620056 | EPI637043 | EPI628322 |  | EPI599528 | EPI846029 | EPI1092734 | EPI553355 | EPI692278 | EPI652964 |
| EPI592045 | EPI596956 | EPI1090442 | EPI628070 | EPI627557 | EPI637293 | EPI637977 | EPI535141 |  | EPI578041 | EPI683150 | EPI1092014 | EPI680873 | EPI530507 | EPI654462 |
| EPI592418 | EPI597514 | EPI1101420 | EPI593159 | EPI627565 | EPI447752 | EPI637454 | EPI628330 |  | EPI599761 | EPI846037 | EPI1098446 | EPI470589 | EPI1091971 | EPI681167 |
| EPI592031 | EPI596962 | EPI1102538 | EPI593243 | EPI628300 | EPI457734 | EPI637942 | EPI628026 |  | EPI530497 | EPI654478 | EPI1092776 | EPI470593 | EPI680945 | EPI681176 |
| EPI592375 | EPI597476 | EPI1102554 | EPI593187 | EPI542092 | EPI637720 | EPI637356 | EPI528348 |  | EPI578065 | EPI654496 | EPI1092080 | EPI470592 | EPI1092475 | EPI961376 |
| EPI1090669 | EPI597593 | EPI1090458 | EPI593644 | EPI627541 | EPI620049 | EPI627947 | EPI528372 |  | EPI599577 | EPI1091728 | EPI1092782 | EPI466451 | EPI1092565 | EPI961409 |
| EPI628264 | EPI597086 | EPI1102930 | EPI592452 | EPI628036 | EPI620181 | EPI637320 | EPI528356 |  | EPI530473 | EPI1091782 | EPI1092896 | EPI466443 | EPI591990 | EPI1092330 |
| EPI535179 | EPI596791 | EPI740735 | EPI592229 | EPI627389 | EPI636917 | EPI638061 | EPI528364 |  | EPI530479 | EPI838018 | EPI1091870 | EPI572842 | EPI680940 | EPI961396 |
| EPI627808 | EPI597119 | EPI887801 | EPI592652 | EPI628044 | EPI637979 | EPI585985 | EPI447648 |  | EPI599373 | EPI1034485 | EPI1092800 | EPI572848 | EPI592013 | EPI961421 |
| EPI627872 | EPI628439 | EPI1103391 | EPI597480 | EPI627581 | EPI637937 | EPI467308 | EPI457679 |  | EPI599437 | EPI1034489 | EPI1055755 | EPI568753 | EPI583843 | EPI1092420 |
| EPI627888 | EPI581719 | EPI1018254 | EPI597392 | EPI628292 | EPI613810 | EPI636746 | EPI457687 |  | EPI599814 | EPI1034487 | EPI1098448 | EPI568756 | EPI838150 | EPI1092114 |
| EPI627904 | EPI581694 | EPI1101684 | EPI596912 | EPI627573 | EPI638070 | EPI655993 | EPI531771 |  | EPI600080 | EPI1034486 | EPI1092824 | EPI635474 | EPI1092499 | EPI1092522 |
| EPI627824 | EPI581764 | EPI1102962 | EPI596946 | EPI627525 | EPI516363 | EPI637022 | EPI1090193 |  | EPI599507 | EPI1034484 | EPI1092728 | EPI568759 | EPI838146 | EPI1092078 |
| EPI627896 | EPI515860 | EPI1102946 | EPI566082 | EPI627613 | EPI620145 | EPI497905 | EPI531763 |  | EPI599486 | EPI1034488 | EPI1092056 | EPI568755 | EPI680938 | EPI1092342 |
| EPI592223 | EPI503535 | EPI1102954 | EPI627495 | EPI535183 | EPI1090670 | EPI637239 | EPI628194 |  | EPI599514 | EPI838012 | EPI1092092 | EPI568754 | EPI680937 | EPI1092780 |
| EPI592216 | EPI581752 | EPI833710 | EPI1090235 | EPI535119 | EPI568649 | EPI636709 | EPI628146 |  | EPI599542 | EPI1093198 | EPI1092764 | EPI467579 | EPI680939 | EPI1091910 |
| EPI592237 | EPI515884 | EPI1101468 | EPI1090195 | EPI628340 | EPI637748 | EPI620026 | EPI628266 |  | EPI599479 | EPI1055757 | EPI1076653 | EPI680849 | EPI553357 | EPI1092792 |
| EPI592258 | EPI506235 | EPI1100436 | EPI1090227 | EPI1090810 | EPI447781 | EPI637823 | EPI628394 |  | EPI599408 | EPI1092952 | EPI1093250 | EPI450832 | EPI652893 | EPI1098442 |
| EPI592547 | EPI581676 | EPI1100428 | EPI628806 | EPI628764 | EPI447675 | EPI620143 | EPI1090815 |  | EPI599416 | EPI652984 | EPI1092944 | EPI532342 | EPI652907 | EPI1034474 |
| EPI592554 | EPI597347 | EPI888057 | EPI628598 | EPI628548 | EPI497895 | EPI443638 | EPI628218 |  | EPI599493 | EPI681057 | EPI1092452 | EPI468352 | EPI652892 | EPI1034475 |
| EPI592288 | EPI597260 | EPI888049 | EPI627303 | EPI628564 | EPI443035 | EPI476481 | EPI628154 |  | EPI599387 | EPI681067 | EPI1092446 | EPI592018 | EPI652896 | EPI1034477 |
| EPI592618 | EPI596942 | EPI887985 | EPI627039 | EPI628604 | EPI545796 | EPI1090680 | EPI628202 |  | EPI599465 | EPI681068 | EPI1092428 | EPI492266 | EPI652894 | EPI1034472 |
| EPI592151 | EPI596892 | EPI971377 | EPI566129 | EPI628780 | EPI471840 | EPI457648 | EPI531779 |  | EPI599394 | EPI652974 | EPI1092440 | EPI492259 | EPI681162 | EPI838186 |
| EPI592599 | EPI597389 | EPI1100468 | EPI627311 | EPI1101195 | EPI535124 | EPI1018256 | EPI656220 |  | EPI599451 | EPI681062 | EPI1092626 | EPI466459 | EPI654306 | EPI1034476 |
| EPI592585 | EPI581617 | EPI1100476 | EPI627463 | EPI1101203 | EPI627586 | EPI1101682 | EPI531787 |  | EPI600059 | EPI680698 | EPI1091786 | EPI596706 | EPI654267 | EPI1034473 |
| EPI593153 | EPI581688 | EPI888041 | EPI627295 | EPI1101427 | EPI460752 | EPI682988 | EPI450846 |  | EPI599401 | EPI680693 | EPI1093130 | EPI583841 | EPI654311 | EPI843881 |
| EPI592625 | EPI581624 | EPI1100956 | EPI627255 | EPI628676 | EPI497911 | EPI692300 | EPI457437 |  | EPI600094 | EPI680695 | EPI1092362 | EPI838085 | EPI1092883 | EPI1092282 |
| EPI592165 | EPI581645 | EPI1101948 | EPI769564 | EPI628788 | EPI498099 | EPI627244 | EPI509066 |  | EPI600108 | EPI680697 | EPI1092170 | EPI591964 | EPI838148 | EPI1092324 |
| EPI592122 | EPI581638 | EPI1101956 | EPI769573 | EPI628668 | EPI453650 | EPI1101562 | EPI895545 |  | EPI599458 | EPI680679 | EPI1092320 | EPI596684 | EPI838121 | EPI1092528 |
| EPI592251 | EPI578241 | EPI888025 | EPI627103 | EPI1101435 | EPI467314 | EPI971303 | EPI628186 |  | EPI599430 | EPI583717 | EPI1092578 | EPI470574 | EPI681166 | EPI1092288 |
| EPI592319 | EPI581712 | EPI971409 | EPI627223 | EPI740734 | EPI457830 | EPI1102568 | EPI531799 |  | EPI599535 | EPI591955 | EPI844509 | EPI470575 | EPI465416 | EPI1092516 |
| EPI592179 | EPI581918 | EPI1102410 | EPI627862 | EPI627197 | EPI895537 | EPI971271 | EPI628130 |  | EPI599549 | EPI681078 | EPI844511 | EPI1086303 | EPI838149 | EPI1092450 |
| EPI583925 | EPI597062 | EPI1102346 | EPI756013 | EPI656302 | EPI895536 | EPI1103962 | EPI1090481 |  | EPI600073 | EPI681063 | EPI1092632 | EPI594368 | EPI838152 | EPI843883 |
| EPI594346 | EPI578154 | EPI971321 | EPI1055422 | EPI1103883 | EPI895532 | EPI1101146 | EPI895541 |  | EPI1155636 | EPI681077 | EPI1092314 | EPI767542 | EPI599759 | EPI1092444 |
| EPI583939 | EPI597528 | EPI1102362 | EPI628414 | EPI683243 | EPI895531 | EPI1090208 | EPI576546 |  | EPI599733 | EPI654298 | EPI1091750 | EPI767556 | EPI1091731 | EPI1092570 |
| EPI594157 | EPI597553 | EPI1102442 | EPI509999 | EPI1100515 | EPI895533 | EPI1100794 | EPI1090457 |  | EPI600052 | EPI1091998 | EPI1092890 | EPI691657 | EPI1092817 | EPI1092606 |
| EPI594164 | EPI578080 | EPI971401 | EPI1090211 | EPI866565 | EPI1090182 | EPI1090416 | EPI895547 |  | EPI599500 | EPI1092004 | EPI1092848 | EPI466475 | EPI1091839 | EPI838199 |
| EPI583899 | EPI596812 | EPI888001 | EPI1090219 | EPI627173 | EPI576579 | EPI1100786 | EPI531472 |  | EPI599423 | EPI844501 | EPI1092830 | EPI515814 | EPI1055728 | EPI654329 |
| EPI594213 | EPI502372 | EPI888073 | EPI769637 | EPI628828 | EPI583116 | EPI866572 | EPI628426 |  | EPI600087 | EPI1092142 | EPI1092854 | EPI515805 | EPI1093237 | EPI654272 |
| EPI594206 | EPI656257 | EPI888009 | EPI769581 | EPI1090564 | EPI628249 | EPI1101610 | EPI1090843 |  | EPI599444 | EPI1092124 | EPI1092860 | EPI572855 | EPI1092541 | EPI1092150 |
| EPI594220 | EPI707953 | EPI888017 | EPI627367 | EPI628796 | EPI582341 | EPI1101642 | EPI627507 |  | EPI599740 | EPI1092412 | EPI1092866 | EPI466243 | EPI1092271 | EPI1092156 |
| EPI594241 | EPI707952 | EPI971425 | EPI560396 | EPI683246 | EPI583109 | EPI930809 | EPI627866 |  | EPI530491 | EPI1092490 | EPI654307 | EPI466267 | EPI1092451 | EPI654300 |
| EPI594339 | EPI707954 | EPI971433 | EPI627359 | EPI1090620 | EPI447798 | EPI887831 | EPI576551 |  | EPI530455 | EPI1092562 | EPI1091768 | EPI569442 | EPI1092289 | EPI838163 |
| EPI594325 | EPI566671 | EPI971473 | EPI866575 | EPI1090548 | EPI593342 | EPI1101554 | EPI541778 |  | EPI530461 | EPI1093174 | EPI1092248 | EPI568741 | EPI1092397 | EPI583450 |
| EPI594269 | EPI580317 | EPI971297 | EPI1102061 | EPI1090500 | EPI590927 | EPI1100810 | EPI535125 |  | EPI683196 | EPI1093024 | EPI1092500 | EPI466427 | EPI1092379 | EPI838174 |
| EPI594297 | EPI580293 | EPI971257 | EPI1100749 | EPI628812 | EPI447885 | EPI730490 | EPI627587 |  | EPI1092093 | EPI1093114 | EPI1093136 | EPI764816 | EPI1092481 | EPI583534 |
| EPI594136 | EPI559416 | EPI971481 | EPI656368 | EPI1100827 | EPI583123 | EPI1040482 | EPI628378 |  | EPI1093251 | EPI1093168 | EPI1093142 | EPI568798 | EPI682976 | EPI583381 |
| EPI594283 | EPI628711 | EPI1100548 | EPI535121 | EPI942206 | EPI628353 | EPI884217 | EPI656189 |  | EPI1092459 | EPI1093156 | EPI1093034 | EPI568796 | EPI942107 | EPI583388 |
| EPI594199 | EPI566035 | EPI1102602 | EPI535153 | EPI1101587 | EPI535132 | EPI1101650 | EPI895546 |  | EPI1092489 | EPI1093126 | EPI1091732 | EPI466497 | EPI681160 | EPI654321 |
| EPI594332 | EPI656297 | EPI1101804 | EPI628342 | EPI1100819 | EPI628313 | EPI1101570 | EPI628082 |  | EPI1092561 | EPI1093108 | EPI1092284 | EPI466467 | EPI942114 | EPI838189 |
| EPI594178 | EPI580269 | EPI1102250 | EPI627615 | EPI979569 | EPI535140 | EPI1022684 | EPI628242 |  | EPI1092327 | EPI1093246 | EPI1092398 | EPI596691 | EPI1103191 | EPI583513 |
| EPI594353 | EPI580349 | EPI1103996 | EPI627567 | EPI1090492 | EPI628321 | EPI1022676 | EPI628346 |  | EPI1092519 | EPI1093090 | EPI1092278 | EPI506281 | EPI1091833 | EPI583366 |
| EPI594248 | EPI580357 | EPI1103956 | EPI627527 | EPI961186 | EPI535156 | EPI1022692 | EPI592636 |  | EPI1092423 | EPI1093150 | EPI1091924 | EPI594474 | EPI1091845 | EPI583604 |
| EPI594276 | EPI580339 | EPI1104004 | EPI535201 | EPI1101539 | EPI628329 | EPI1040890 | EPI628178 |  | EPI1098459 | EPI1093102 | EPI1092542 | EPI596660 | EPI654290 | EPI654405 |
| EPI594304 | EPI580301 | EPI971385 | EPI627535 | EPI1101483 | EPI583152 | EPI1018193 | EPI656236 |  | EPI1092255 | EPI1093120 | EPI1092272 | EPI974023 | EPI654295 | EPI530518 |
| EPI594318 | EPI580329 | EPI971393 | EPI627559 | EPI833713 | EPI583096 | EPI1018147 | EPI578033 |  | EPI1091997 | EPI654386 | EPI1092206 | EPI1055441 | EPI1034458 | EPI530500 |
| EPI583918 | EPI566091 | EPI971441 | EPI628302 | EPI1102027 | EPI1090630 | EPI1018171 | EPI587895 |  | EPI1092003 | EPI583338 | EPI1092212 | EPI680841 | EPI1034457 | EPI530506 |
| EPI594143 | EPI580277 | EPI971361 | EPI535177 | EPI1102545 | EPI1090678 | EPI1018155 | EPI627179 |  | EPI1092555 | EPI530504 | EPI1092200 | EPI680840 | EPI1034459 | EPI654382 |
| EPI594192 | EPI656409 | EPI971369 | EPI628238 | EPI1103379 | EPI1090662 | EPI1054113 | EPI627187 |  | EPI1092609 | EPI530510 | EPI1092074 | EPI680842 | EPI838144 | EPI654341 |
| EPI594255 | EPI627064 | EPI888033 | EPI628046 | EPI866557 | EPI477411 | EPI1054129 | EPI656308 |  | EPI1091823 | EPI591935 | EPI1092296 | EPI468358 | EPI654420 | EPI680998 |
| EPI594171 | EPI580285 | EPI971353 | EPI535193 | EPI1101547 | EPI477449 | EPI1067568 | EPI628562 |  | EPI1091883 | EPI583634 | EPI1098447 | EPI492273 | EPI1034455 | EPI681170 |
| EPI583932 | EPI656361 | EPI971417 | EPI528336 | EPI917064 | EPI583089 | EPI973366 | EPI628546 |  | EPI1091889 | EPI680694 | EPI1092506 | EPI591988 | EPI1034454 | EPI1155618 |
| EPI583905 | EPI578207 | EPI1100780 | EPI528296 | EPI1090516 | EPI583045 | EPI1018091 | EPI628810 |  | EPI1093029 | EPI1055702 | EPI1092512 | EPI1155623 | EPI1034456 | EPI1155635 |
| EPI594311 | EPI654152 | EPI1104060 | EPI627543 | EPI1090508 | EPI582938 | EPI1022668 | EPI1101201 |  | EPI1092009 | EPI680719 | EPI1091936 | EPI680868 | EPI680964 | EPI596723 |
| EPI594227 | EPI578003 | EPI1100756 | EPI542091 | EPI887888 | EPI532266 | EPI1102592 | EPI1101193 |  | EPI1092063 | EPI680718 | EPI1092620 | EPI680850 | EPI599582 | EPI681033 |
| EPI594234 | EPI656045 | EPI1102882 | EPI533257 | EPI1103335 | EPI582960 | EPI1102010 | EPI1101425 |  | EPI736582 | EPI680720 | EPI1092986 | EPI492280 | EPI594412 | EPI568670 |
| EPI592312 | EPI654124 | EPI1102890 | EPI533248 | EPI1103359 | EPI503510 | EPI1018131 | EPI628674 |  | EPI1092525 | EPI680717 | EPI1093022 | EPI532343 | EPI680968 | EPI596694 |
| EPI594290 | EPI627696 | EPI1100724 | EPI533241 | EPI887864 | EPI576576 | EPI1100842 | EPI628778 |  | EPI1092795 | EPI681065 | EPI1092086 | EPI473794 | EPI583638 | EPI583343 |
| EPI594262 | EPI628015 | EPI1100740 | EPI1057958 | EPI1103308 | EPI576578 | EPI1022703 | EPI1101433 |  | EPI1092819 | EPI681064 | EPI942205 | EPI532341 | EPI654389 | EPI583639 |
| EPI594185 | EPI1090311 | EPI1100748 | EPI628142 | EPI1101147 | EPI1090654 | EPI1018083 | EPI628666 |  | EPI1092807 | EPI680721 | EPI1092704 | EPI680851 | EPI654475 | EPI654335 |
| EPI594150 | EPI627632 | EPI1102898 | EPI535185 | EPI887880 | EPI510002 | EPI1022652 | EPI628786 |  | EPI1092825 | EPI680722 | EPI1090078 | EPI492287 | EPI681154 | EPI764641 |
| EPI583893 | EPI627847 | EPI1100820 | EPI627575 | EPI1101491 | EPI457662 | EPI1018261 | EPI621119 |  | EPI1092837 | EPI680704 | EPI691904 | EPI680848 | EPI654317 | EPI681169 |
| EPI583911 | EPI597155 | EPI1055413 | EPI627583 | EPI866549 | EPI447661 | EPI1022636 | EPI628594 |  | EPI1092843 | EPI680701 | EPI577315 | EPI470591 | EPI530501 | EPI596730 |
| EPI627601 | EPI1090351 | EPI979570 | EPI535169 | EPI1100435 | EPI509116 | EPI1018244 | EPI627131 |  | EPI942268 | EPI680700 | EPI577318 | EPI569565 | EPI692279 | EPI468360 |
| EPI628240 | EPI505123 | EPI1101028 | EPI628294 | EPI888040 | EPI628025 | EPI1022620 | EPI621131 |  | EPI1076650 | EPI680706 | EPI1055452 | EPI680853 | EPI592006 | EPI530476 |
| EPI593334 | EPI627855 | EPI1100836 | EPI628062 | EPI887984 | EPI628209 | EPI1040458 | EPI566070 |  | EPI1092117 | EPI680708 | EPI591183 | EPI1103205 | EPI583857 | EPI530470 |
| EPI593341 | EPI656210 | EPI942266 | EPI627599 | EPI1100427 | EPI628161 | EPI1010102 | EPI621124 |  | EPI680523 | EPI680705 | EPI577184 | EPI680872 | EPI680959 | EPI599379 |
| EPI627561 | EPI509111 | EPI1101484 | EPI627838 | EPI888016 | EPI627921 | EPI1040474 | EPI1090545 |  | EPI838236 | EPI680707 | EPI577150 | EPI681119 | EPI681153 | EPI654294 |
| EPI509669 | EPI656131 | EPI1101540 | EPI627415 | EPI888008 | EPI628169 | EPI1018115 | EPI627291 |  | EPI623615 | EPI681071 | EPI577152 | EPI680875 | EPI1055459 | EPI1093152 |
| EPI1179235 | EPI507086 | EPI1102028 | EPI627407 | EPI971424 | EPI663479 | EPI1018123 | EPI769571 |  | EPI1093005 | EPI681061 | EPI577181 | EPI680879 | EPI594363 | EPI1093158 |
| EPI528354 | EPI1090335 | EPI1101492 | EPI627447 | EPI971432 | EPI457438 | EPI1018107 | EPI769579 |  | EPI1093011 | EPI652980 | EPI577183 | EPI680878 | EPI838137 | EPI599576 |
| EPI528346 | EPI1090383 | EPI1090738 | EPI583129 | EPI1102409 | EPI509067 | EPI1018236 | EPI773729 |  | EPI1093017 | EPI652982 | EPI1092008 | EPI680861 | EPI1098427 | EPI1091766 |
| EPI627840 | EPI654137 | EPI866542 | EPI628182 | EPI971408 | EPI627865 | EPI1100850 | EPI628658 |  | EPI1092993 | EPI652979 | EPI577182 | EPI680870 | EPI1092619 | EPI1092498 |
| EPI627417 | EPI656417 | EPI866558 | EPI531474 | EPI888024 | EPI1090638 | EPI1018099 | EPI628730 |  | EPI1092999 | EPI652991 | EPI683234 | EPI680871 | EPI1092985 | EPI1092906 |
| EPI627449 | EPI1101534 | EPI1101548 | EPI628430 | EPI888072 | EPI1090646 | EPI1022604 | EPI1100697 |  | EPI1091793 | EPI652990 | EPI1092572 | EPI573384 | EPI1093021 | EPI1092912 |
| EPI627409 | EPI627072 | EPI1100524 | EPI628350 | EPI1102361 | EPI627138 | EPI1100858 | EPI1022667 |  | EPI1091865 | EPI652989 | EPI1092596 | EPI591957 | EPI1092103 | EPI1092942 |
| EPI528370 | EPI656393 | EPI919612 | EPI628382 | EPI888000 | EPI627146 | EPI1018139 | EPI1102591 |  | EPI1091853 | EPI652981 | EPI1092416 | EPI590935 | EPI1091707 | EPI602293 |
| EPI535203 | EPI628527 | EPI1090394 | EPI628134 | EPI1102345 | EPI447904 | EPI1013228 | EPI683114 |  | EPI1091859 | EPI652985 | EPI598339 | EPI590936 | EPI652904 | EPI578047 |
| EPI627393 | EPI627983 | EPI1074591 | EPI1090475 | EPI1102441 | EPI941040 | EPI1100866 | EPI627003 |  | EPI1091919 | EPI652983 | EPI683236 | EPI680846 | EPI680985 | EPI602360 |
| EPI628048 | EPI654040 | EPI926822 | EPI628278 | EPI1102577 | EPI569718 | EPI628067 | EPI627091 |  | EPI1091931 | EPI652986 | EPI683235 | EPI583636 | EPI599680 | EPI530512 |
| EPI628040 | EPI628495 | EPI1013238 | EPI628286 | EPI1101955 | EPI447917 | EPI1090814 | EPI866571 |  | EPI1091805 | EPI652978 | EPI599219 | EPI596727 | EPI681157 | EPI599569 |
| EPI528378 | EPI655877 | EPI1013254 | EPI628190 | EPI1101947 | EPI639588 | EPI1090576 | EPI628762 |  | EPI1091925 | EPI652975 | EPI598466 | EPI681114 | EPI680958 | EPI691665 |
| EPI528298 | EPI628095 | EPI1090298 | EPI628086 | EPI971368 | EPI453612 | EPI1102544 | EPI566133 |  | EPI1091817 | EPI652977 | EPI599278 | EPI681115 | EPI680960 | EPI599767 |
| EPI566124 | EPI655885 | EPI1074599 | EPI578028 | EPI971360 | EPI446458 | EPI627859 | EPI627011 |  | EPI1091769 | EPI652976 | EPI599268 | EPI1055457 | EPI470071 | EPI599638 |
| EPI628328 | EPI627991 | EPI1013174 | EPI628246 | EPI971352 | EPI469527 | EPI756019 | EPI627451 |  | EPI578196 | EPI652973 | EPI599274 | EPI838120 | EPI470068 | EPI530482 |
| EPI628320 | EPI656053 | EPI1013182 | EPI656193 | EPI971440 | EPI497858 | EPI583908 | EPI941051 |  | EPI1092579 | EPI652987 | EPI599225 | EPI843853 | EPI470072 | EPI1091748 |
| EPI535147 | EPI656037 | EPI1013206 | EPI627127 | EPI888048 | EPI497874 | EPI594223 | EPI628738 |  | EPI1092447 | EPI652972 | EPI598458 | EPI680881 | EPI594384 | EPI1091826 |
| EPI628336 | EPI627967 | EPI1013190 | EPI1090635 | EPI888056 | EPI457854 | EPI594202 | EPI627491 |  | EPI1093131 | EPI681060 | EPI599220 | EPI583719 | EPI583787 | EPI1092726 |
| EPI535139 | EPI655997 | EPI1013166 | EPI1090179 | EPI1100755 | EPI468971 | EPI594307 | EPI656476 |  | EPI1092399 | EPI681058 | EPI599266 | EPI1103557 | EPI594377 | EPI1092048 |
| EPI535163 | EPI578017 | EPI1013198 | EPI703992 | EPI1102849 | EPI572729 | EPI594356 | EPI628690 |  | EPI1092279 | EPI681059 | EPI598347 | EPI680857 | EPI838131 | EPI1091796 |
| EPI535187 | EPI578024 | EPI1090290 | EPI627143 | EPI1100747 | EPI453475 | EPI594328 | EPI627163 |  | EPI1091799 | EPI1092232 | EPI598452 | EPI680852 | EPI583780 | EPI1092084 |
| EPI627249 | EPI1090439 | EPI1090386 | EPI627151 | EPI1102897 | EPI467344 | EPI583896 | EPI626979 |  | EPI1092339 | EPI1191057 | EPI598492 | EPI680854 | EPI599784 | EPI1098444 |
| EPI627577 | EPI656061 | EPI1013214 | EPI627247 | EPI971384 | EPI447858 | EPI583914 | EPI627155 |  | EPI1092345 | EPI1092526 | EPI568449 | EPI583454 | EPI1092715 | EPI1092882 |
| EPI535155 | EPI628423 | EPI1022614 | EPI1102307 | EPI971392 | EPI1090190 | EPI594153 | EPI628610 |  | EPI1093191 | EPI1091716 | EPI599207 | EPI680865 | EPI1092127 | EPI1093032 |
| EPI627545 | EPI627672 | EPI1013262 | EPI580369 | EPI971416 | EPI656379 | EPI594209 | EPI628570 |  | EPI1092273 | EPI1091722 | EPI599208 | EPI680866 | EPI1092109 | EPI1092198 |
| EPI542093 | EPI655973 | EPI1018093 | EPI628758 | EPI971320 | EPI628193 | EPI594251 | EPI628578 |  | EPI1092543 | EPI1093036 | EPI568450 | EPI680864 | EPI1092193 | EPI1092000 |
| EPI528338 | EPI656170 | EPI973370 | EPI627439 | EPI1104059 | EPI628145 | EPI583921 | EPI628650 |  | EPI1092285 | EPI838034 | EPI598646 | EPI680867 | EPI1092151 | EPI1092138 |
| EPI628296 | EPI509133 | EPI1018141 | EPI566098 | EPI888032 | EPI628265 | EPI594188 | EPI628586 |  | EPI1092531 | EPI654357 | EPI598647 | EPI680860 | EPI1092265 | EPI1091994 |
| EPI509672 | EPI509138 | EPI1018149 | EPI627910 | EPI1100467 | EPI1090494 | EPI594265 | EPI1090829 |  | EPI1092291 | EPI654351 | EPI530454 | EPI680876 | EPI680975 | EPI1092054 |
| EPI627617 | EPI656178 | EPI1018125 | EPI627918 | EPI1100475 | EPI457878 | EPI594195 | EPI627075 |  | EPI1092453 | EPI654363 | EPI568432 | EPI680863 | EPI680971 | EPI1092438 |
| EPI628144 | EPI628479 | EPI1022622 | EPI566106 | EPI971256 | EPI576580 | EPI594146 | EPI627083 |  | EPI1091907 | EPI1091968 | EPI568441 | EPI681126 | EPI680969 | EPI1091784 |
| EPI535195 | EPI655941 | EPI1018173 | EPI627830 | EPI1100955 | EPI457718 | EPI594321 | EPI1090737 |  | EPI1092729 | EPI1092760 | EPI599264 | EPI596720 | EPI680966 | EPI1092432 |
| EPI628344 | EPI655909 | EPI1018085 | EPI627351 | EPI971296 | EPI447946 | EPI594216 | EPI531805 |  | EPI1092207 | EPI1103522 | EPI568435 | EPI680874 | EPI680973 | EPI1092576 |
| EPI628064 | EPI628455 | EPI1018199 | EPI566050 | EPI971472 | EPI542312 | EPI594335 | EPI628802 |  | EPI1092213 | EPI1103530 | EPI530460 | EPI1155615 | EPI680970 | EPI1092312 |
| EPI628304 | EPI627951 | EPI1018133 | EPI627327 | EPI971480 | EPI979550 | EPI594237 | EPI627275 |  | EPI1092411 | EPI680524 | EPI530514 | EPI1155632 | EPI680982 | EPI1092594 |
| EPI535171 | EPI1090431 | EPI1018263 | EPI566114 | EPI1101803 | EPI509074 | EPI594293 | EPI729463 |  | EPI1091949 | EPI838237 | EPI598648 | EPI572854 | EPI583456 | EPI1092462 |
| EPI627585 | EPI655949 | EPI1100852 | EPI566058 | EPI1101971 | EPI576581 | EPI583928 | EPI627035 |  | EPI1092375 | EPI1091980 | EPI530520 | EPI845709 | EPI680972 | EPI1092504 |
| EPI535123 | EPI1090319 | EPI1067575 | EPI1090595 | EPI1103955 | EPI583073 | EPI583942 | EPI566125 |  | EPI1092405 | EPI1091770 | EPI530478 | EPI572852 | EPI680987 | EPI1092474 |
| EPI627537 | EPI509887 | EPI1010096 | EPI627343 | EPI1104003 | EPI627394 | EPI594314 | EPI729455 |  | EPI1093137 | EPI683149 | EPI530466 | EPI572841 | EPI470070 | EPI1092510 |
| EPI528362 | EPI656021 | EPI1054131 | EPI627319 | EPI1102249 | EPI451256 | EPI594181 | EPI628722 |  | EPI1093143 | EPI729797 | EPI530472 | EPI583806 | EPI583342 | EPI1098443 |
| EPI627529 | EPI627728 | EPI1018109 | EPI1090603 | EPI1103995 | EPI504716 | EPI594279 | EPI627283 |  | EPI1092483 | EPI1092976 | EPI598645 | EPI942167 | EPI528389 | EPI1092804 |
| EPI447600 | EPI656077 | EPI1018246 | EPI566066 | EPI866541 | EPI504728 | EPI594272 | EPI1055418 |  | EPI1092477 | EPI1092982 | EPI598441 | EPI1092491 | EPI528381 | EPI1092822 |
| EPI457869 | EPI656069 | EPI1040460 | EPI627503 | EPI1100835 | EPI443676 | EPI592322 | EPI627235 |  | EPI1092507 | EPI1092712 | EPI599272 | EPI1055626 | EPI528397 | EPI1092558 |
| EPI497836 | EPI627975 | EPI1100860 | EPI729449 | EPI1101467 | EPI450843 | EPI594258 | EPI464586 |  | EPI1092513 | EPI1055619 | EPI599221 | EPI1055752 | EPI681165 | EPI1092486 |
| EPI497849 | EPI656029 | EPI1100868 | EPI531801 | EPI1090300 | EPI450851 | EPI583935 | EPI627251 |  | EPI1092501 | EPI942227 | EPI599209 | EPI594453 | EPI681158 | EPI1092612 |
| EPI638095 | EPI627799 | EPI1100844 | EPI729457 | EPI1074601 | EPI446453 | EPI594167 | EPI627307 |  | EPI1091757 | EPI1055811 | EPI599269 | EPI1103553 | EPI681150 | EPI1092408 |
| EPI638102 | EPI656101 | EPI1022606 | EPI566136 | EPI1013173 | EPI452269 | EPI592182 | EPI942288 |  | EPI1103523 | EPI1055710 | EPI599270 | EPI1103554 | EPI681151 | EPI1092564 |
| EPI440685 | EPI627656 | EPI1040476 | EPI1101301 | EPI1013197 | EPI656180 | EPI594286 | EPI628634 |  | EPI1103531 | EPI1055749 | EPI838045 | EPI1103555 | EPI838160 | EPI1091946 |
| EPI440693 | EPI656123 | EPI1018101 | EPI1101309 | EPI1013165 | EPI628393 | EPI594342 | EPI627379 |  | EPI1191060 | EPI1055795 | EPI599271 | EPI1103556 | EPI1091869 | EPI1093146 |
| EPI457837 | EPI553469 | EPI1018242 | EPI1090747 | EPI1013189 | EPI628153 | EPI594160 | EPI656260 |  | EPI1191062 | EPI1055483 | EPI599277 | EPI1091825 | EPI1092721 | EPI1093104 |
| EPI464556 | EPI627272 | EPI1018157 | EPI1103402 | EPI1013181 | EPI628201 | EPI594244 | EPI628626 |  | EPI858163 | EPI1055499 | EPI599224 | EPI1091765 | EPI591939 | EPI1093116 |
| EPI469657 | EPI656273 | EPI1022638 | EPI580377 | EPI1013205 | EPI531762 | EPI592315 | EPI773735 |  | EPI1092021 | EPI1092874 | EPI599275 | EPI1091759 | EPI1055451 | EPI1092984 |
| EPI469659 | EPI627200 | EPI1054115 | EPI580385 | EPI1074593 | EPI531770 | EPI594230 | EPI656372 |  | EPI1092753 | EPI1092700 | EPI599223 | EPI1092581 | EPI838156 | EPI1093020 |
| EPI469671 | EPI656305 | EPI1022686 | EPI1057918 | EPI1090292 | EPI531778 | EPI594139 | EPI656468 |  | EPI1092081 | EPI1091812 | EPI599267 | EPI1092587 | EPI654445 | EPI1093080 |
| EPI469670 | EPI578094 | EPI1022678 | EPI971338 | EPI1090396 | EPI531786 | EPI594300 | EPI656420 |  | EPI1092783 | EPI1092070 | EPI599222 | EPI1093247 | EPI654463 | EPI1093134 |
| EPI469669 | EPI578194 | EPI1022694 | EPI971346 | EPI926821 | EPI1055417 | EPI594174 | EPI627227 |  | EPI1092777 | EPI1093204 | EPI530496 | EPI1092761 | EPI1091857 | EPI1093092 |
| EPI469658 | EPI597126 | EPI1022705 | EPI1055406 | EPI919611 | EPI1090734 | EPI583902 | EPI627115 |  | EPI1092435 | EPI1093192 | EPI598566 | EPI1076655 | EPI1091863 | EPI1093140 |
| EPI469660 | EPI597140 | EPI1040901 | EPI1101293 | EPI1013213 | EPI583145 | EPI594349 | EPI773723 |  | EPI1092363 | EPI1092178 | EPI599210 | EPI1076679 | EPI573380 | EPI1091772 |
| EPI590926 | EPI509149 | EPI1022654 | EPI1101285 | EPI1090388 | EPI583138 | EPI1100442 | EPI628682 |  | EPI1092597 | EPI1191054 | EPI598471 | EPI1076663 | EPI680963 | EPI530452 |
| EPI1090661 | EPI627783 | EPI1022670 | EPI1102323 | EPI1013237 | EPI451273 | EPI1100450 | EPI656348 |  | EPI1092621 | EPI1092538 | EPI568434 | EPI1076671 | EPI680978 | EPI530458 |
| EPI1090653 | EPI627752 | EPI1102594 | EPI887674 | EPI1013253 | EPI447956 | EPI979644 | EPI1102303 |  | EPI1092471 | EPI1092904 | EPI598442 | EPI838108 | EPI680962 | EPI602286 |
| EPI1090637 | EPI1090279 | EPI1013230 | EPI887810 | EPI1054130 | EPI497850 | EPI627060 | EPI626995 |  | EPI1091829 | EPI1092358 | EPI599226 | EPI680859 | EPI529239 | EPI602367 |
| EPI1090677 | EPI627680 | EPI1018117 | EPI1101933 | EPI1010098 | EPI628073 | EPI656501 | EPI831439 |  | EPI1091835 | EPI1191058 | EPI599273 | EPI599559 | EPI692277 | EPI1103213 |
| EPI457885 | EPI593336 | EPI447837 | EPI1100717 | EPI1018108 | EPI447654 | EPI1101474 | EPI1057954 |  | EPI837987 | EPI1191059 | EPI598440 | EPI583785 | EPI692280 | EPI1092888 |
| EPI576567 | EPI1090423 | EPI457666 | EPI1101925 | EPI1018247 | EPI497903 | EPI1104018 | EPI1057962 |  | EPI1092381 | EPI1093216 | EPI599276 | EPI583778 | EPI1092937 | EPI641388 |
| EPI439502 | EPI627712 | EPI457650 | EPI1090587 | EPI1040459 | EPI663481 | EPI1102143 | EPI585264 |  | EPI844492 | EPI1092646 | EPI577890 | EPI594382 | EPI1103525 | EPI942297 |
| EPI448936 | EPI627640 | EPI582338 | EPI1100445 | EPI1067572 | EPI545803 | EPI1090720 | EPI773716 |  | EPI1092159 | EPI1092352 | EPI1155638 | EPI594375 | EPI1092967 | EPI961388 |
| EPI576568 | EPI656147 | EPI583106 | EPI1100453 | EPI1022621 | EPI545789 | EPI1101010 | EPI773740 |  | EPI1092261 | EPI1092658 | EPI691914 | EPI838089 | EPI1191026 | EPI1092336 |
| EPI568648 | EPI656155 | EPI1090242 | EPI1100525 | EPI1022605 | EPI663480 | EPI1090828 | EPI627211 |  | EPI1091787 | EPI1092652 | EPI844504 | EPI599782 | EPI1091773 | EPI1091832 |
| EPI447613 | EPI627999 | EPI1090226 | EPI1100949 | EPI1018100 | EPI515789 | EPI593544 | EPI1057930 |  | EPI1092465 | EPI1092640 | EPI844506 | EPI680831 | EPI1055610 | EPI1091886 |
| EPI457853 | EPI627736 | EPI627358 | EPI1100925 | EPI1018240 | EPI515779 | EPI593384 | EPI773747 |  | EPI1092633 | EPI1092496 | EPI654378 | EPI680832 | EPI654273 | EPI1091928 |
| EPI464557 | EPI656013 | EPI628597 | EPI1100941 | EPI1040475 | EPI628049 | EPI1101018 | EPI627043 |  | EPI1092615 | EPI680699 | EPI654373 | EPI680834 | EPI681164 | EPI578071 |
| EPI654154 | EPI655925 | EPI627366 | EPI1100933 | EPI1100859 | EPI447696 | EPI1102058 | EPI656452 |  | EPI1092315 | EPI680703 | EPI654384 | EPI680833 | EPI846042 | EPI1092786 |
| EPI464554 | EPI655869 | EPI560395 | EPI1101917 | EPI1100867 | EPI895529 | EPI979600 | EPI656428 |  | EPI1092627 | EPI569591 | EPI681086 | EPI529250 | EPI1091893 | EPI652968 |
| EPI497829 | EPI593343 | EPI769636 | EPI1101885 | EPI1018124 | EPI576548 | EPI930818 | EPI654127 |  | EPI1092129 | EPI654293 | EPI573371 | EPI680869 | EPI1091899 | EPI1006757 |
| EPI628160 | EPI628119 | EPI769580 | EPI1102875 | EPI1018148 | EPI541775 | EPI926828 | EPI627259 |  | EPI1092195 | EPI654484 | EPI652789 | EPI680877 | EPI1092949 | EPI1091880 |
| EPI628208 | EPI627744 | EPI443656 | EPI1100645 | EPI1018172 | EPI1090454 | EPI887975 | EPI656340 |  | EPI1092111 | EPI838014 | EPI652795 | EPI691981 | EPI1092583 | EPI838184 |
| EPI656386 | EPI627943 | EPI532308 | EPI1100653 | EPI1018140 | EPI592633 | EPI979512 | EPI773710 |  | EPI1092267 | EPI654428 | EPI652800 | EPI691980 | EPI1092589 | EPI1092234 |
| EPI628392 | EPI596935 | EPI457658 | EPI1100605 | EPI973368 | EPI531797 | EPI887631 | EPI627107 |  | EPI1092105 | EPI654276 | EPI652792 | EPI530499 | EPI1091761 | EPI1093242 |
| EPI1090205 | EPI627776 | EPI703991 | EPI1101813 | EPI1018092 | EPI656235 | EPI1090464 | EPI656316 |  | EPI1092303 | EPI596689 | EPI652787 | EPI530505 | EPI654469 | EPI1093236 |
| EPI627377 | EPI656005 | EPI442709 | EPI1101789 | EPI1100851 | EPI443636 | EPI1101770 | EPI1090505 |  | EPI1092309 | EPI591928 | EPI652798 | EPI691697 | EPI838162 | EPI530464 |
| EPI628200 | EPI627704 | EPI477407 | EPI1101821 | EPI1100843 | EPI443043 | EPI1018219 | EPI1090201 |  | EPI1076641 | EPI473791 | EPI652788 | EPI838118 | EPI1092871 | EPI1092006 |
| EPI628272 | EPI656093 | EPI477446 | EPI1100621 | EPI1022677 | EPI441796 | EPI872953 | EPI593345 |  | EPI1092567 | EPI466480 | EPI652790 | EPI691698 | EPI729780 | EPI1092018 |
| EPI628152 | EPI578010 | EPI482857 | EPI1101845 | EPI1054114 | EPI447745 | EPI1101658 | EPI447669 |  | EPI1092441 | EPI569589 | EPI652791 | EPI1093205 | EPI846034 | EPI1092066 |
| EPI628400 | EPI597567 | EPI498081 | EPI628542 | EPI1022693 | EPI457774 | EPI1101594 | EPI1101473 |  | EPI1092417 | EPI1092418 | EPI652784 | EPI528395 | EPI1092685 | EPI729790 |
| EPI628224 | EPI597521 | EPI627150 | EPI628558 | EPI1040894 | EPI470595 | EPI1101634 | EPI1104017 |  | EPI844491 | EPI838024 | EPI652786 | EPI548390 | EPI729781 | EPI1093212 |
| EPI1090811 | EPI596988 | EPI628701 | EPI1055398 | EPI1022637 | EPI470597 | EPI593648 | EPI1057922 |  | EPI844493 | EPI594359 | EPI652794 | EPI528379 | EPI838159 | EPI1055791 |
| EPI627521 | EPI596974 | EPI628717 | EPI1090523 | EPI1022685 | EPI683069 | EPI593655 | EPI1057938 |  | EPI1093227 | EPI838022 | EPI652799 | EPI528387 | EPI1093195 | EPI1055799 |
| EPI1090189 | EPI597504 | EPI1090546 | EPI1101093 | EPI1022653 | EPI683068 | EPI627332 | EPI628074 |  | EPI599754 | EPI1086307 | EPI1093028 | EPI681122 | EPI1092745 | EPI1091964 |
| EPI531769 | EPI597048 | EPI628797 | EPI887850 | EPI1022704 | EPI683070 | EPI1090216 | EPI1100457 |  | EPI1092927 | EPI838026 | EPI1091930 | EPI652934 | EPI1093039 | EPI1091970 |
| EPI656187 | EPI578187 | EPI1090562 | EPI888082 | EPI1018156 | EPI528371 | EPI1090224 | EPI971446 |  | EPI1092933 | EPI838030 | EPI1091918 | EPI652945 | EPI1092907 | EPI1092384 |
| EPI531793 | EPI597041 | EPI627174 | EPI887762 | EPI1022669 | EPI528347 | EPI627372 | EPI1102399 |  | EPI1092957 | EPI594408 | EPI1091882 | EPI652936 | EPI1092913 | EPI1091754 |
| EPI531785 | EPI583279 | EPI628829 | EPI1103019 | EPI1102593 | EPI528291 | EPI566095 | EPI627459 |  | EPI1092963 | EPI583853 | EPI1091888 | EPI652933 | EPI1092727 | EPI1092468 |
| EPI531777 | EPI593211 | EPI457674 | EPI1102547 | EPI1018084 | EPI528363 | EPI627020 | EPI1090185 |  | EPI683192 | EPI1091752 | EPI1092026 | EPI652932 | EPI1092385 | EPI1092756 |
| EPI656226 | EPI593314 | EPI941036 | EPI712924 | EPI1018200 | EPI654139 | EPI566031 | EPI627467 |  | EPI1093101 | EPI1091842 | EPI1092050 | EPI652947 | EPI1191029 | EPI1091976 |
| EPI509062 | EPI593302 | EPI457794 | EPI1103382 | EPI1018132 | EPI592362 | EPI656469 | EPI1090233 |  | EPI1093119 | EPI1091896 | EPI1091984 | EPI1092191 | EPI1191028 | EPI1093170 |
| EPI457717 | EPI593296 | EPI439488 | EPI1103893 | EPI1018260 | EPI592404 | EPI580340 | EPI576627 |  | EPI1103235 | EPI1091902 | EPI1091990 | EPI680856 | EPI1092229 | EPI1103209 |
| EPI692158 | EPI593308 | EPI447735 | EPI1102021 | EPI576619 | EPI592025 | EPI628707 | EPI764626 |  | EPI1093209 | EPI1091830 | EPI1092044 | EPI1086310 | EPI1191024 | EPI1092870 |
| EPI692162 | EPI578121 | EPI447810 | EPI979587 | EPI457665 | EPI592081 | EPI580318 | EPI1090641 |  | EPI1093173 | EPI599557 | EPI654433 | EPI583855 | EPI1092073 | EPI1191043 |
| EPI692172 | EPI627624 | EPI457746 | EPI1102259 | EPI1090284 | EPI592095 | EPI566087 | EPI683116 |  | EPI1092153 | EPI599578 | EPI846038 | EPI594361 | EPI1093201 | EPI942295 |
| EPI443667 | EPI656505 | EPI497821 | EPI1102427 | EPI497847 | EPI592088 | EPI656301 | EPI627123 |  | EPI1093089 | EPI692134 | EPI681091 | EPI838106 | EPI1091809 | EPI1055736 |
| EPI692163 | EPI520863 | EPI440682 | EPI1102315 | EPI457657 | EPI592327 | EPI566667 | EPI942296 |  | EPI1093083 | EPI692135 | EPI1006754 | EPI594410 | EPI1103517 | EPI1055740 |
| EPI692156 | EPI516605 | EPI457834 | EPI1101133 | EPI457673 | EPI592348 | EPI559412 | EPI1102431 |  | EPI1093095 | EPI583804 | EPI654470 | EPI838114 | EPI1093177 | EPI1093218 |
| EPI692165 | EPI627688 | EPI453622 | EPI1102299 | EPI1090817 | EPI592369 | EPI580294 | EPI1102967 |  | EPI1091979 | EPI591948 | EPI729793 | EPI838110 | EPI1093183 | EPI1091838 |
| EPI692155 | EPI628535 | EPI469531 | EPI1090771 | EPI447719 | EPI592046 | EPI580286 | EPI979511 |  | EPI1103233 | EPI1155620 | EPI838075 | EPI838107 | EPI1092877 | EPI1091892 |
| EPI692169 | EPI597448 | EPI468964 | EPI1102124 | EPI457428 | EPI592397 | EPI580278 | EPI926819 |  | EPI1092789 | EPI680709 | EPI1092230 | EPI591937 | EPI1092931 | EPI1091898 |
| EPI692161 | EPI597183 | EPI569714 | EPI887738 | EPI628412 | EPI592376 | EPI580304 | EPI740732 |  | EPI1093047 | EPI1092670 | EPI1092548 | EPI681113 | EPI1092925 | EPI1092396 |
| EPI692154 | EPI516597 | EPI453601 | EPI887706 | EPI1090580 | EPI592067 | EPI580358 | EPI833718 |  | EPI1092429 | EPI942234 | EPI1091774 | EPI681120 | EPI1092955 | EPI1092270 |
| EPI692166 | EPI627760 | EPI447922 | EPI1102339 | EPI447908 | EPI592419 | EPI580270 | EPI1090625 |  | EPI1092705 | EPI942888 | EPI1092938 | EPI1076687 | EPI1092961 | EPI1092540 |
| EPI692170 | EPI707949 | EPI469539 | EPI1103257 | EPI532292 | EPI592109 | EPI580350 | EPI919609 |  | EPI1091967 | EPI1093258 | EPI1092968 | EPI1006755 | EPI682978 | EPI1092276 |
| EPI692164 | EPI627664 | EPI639585 | EPI1101509 | EPI457761 | EPI592116 | EPI443678 | EPI971374 |  | EPI1091973 | EPI573388 | EPI681079 | EPI680880 | EPI680521 | EPI1092372 |
| EPI692168 | EPI656233 | EPI467333 | EPI1103949 | EPI443658 | EPI592252 | EPI457848 | EPI1018162 |  | EPI1092663 | EPI596725 | EPI681080 | EPI1092389 | EPI838242 | EPI1092402 |
| EPI447599 | EPI1179237 | EPI442712 | EPI1101125 | EPI628076 | EPI593279 | EPI703997 | EPI1102343 |  | EPI1092249 | EPI654423 | EPI681089 | EPI1191038 | EPI1093027 | EPI729789 |
| EPI457701 | EPI593218 | EPI453482 | EPI1103813 | EPI628396 | EPI592355 | EPI580374 | EPI971406 |  | EPI1092693 | EPI844496 | EPI1191048 | EPI654381 | EPI681155 | EPI1092618 |
| EPI457845 | EPI593204 | EPI447852 | EPI1101101 | EPI1090452 | EPI592334 | EPI628755 | EPI888070 |  | EPI1092219 | EPI1093222 | EPI1191051 | EPI683062 | EPI1092025 | EPI1055753 |
| EPI447617 | EPI593239 | EPI497811 | EPI1101109 | EPI591390 | EPI593245 | EPI1102304 | EPI887998 |  | EPI1092717 | EPI692104 | EPI1191050 | EPI683064 | EPI1091983 | EPI1092918 |
| EPI447622 | EPI597162 | EPI559489 | EPI887962 | EPI531796 | EPI592426 | EPI627907 | EPI971318 |  | EPI1092225 | EPI680715 | EPI1191049 | EPI654283 | EPI1091989 | EPI1191045 |
| EPI477308 | EPI627720 | EPI559488 | EPI1090371 | EPI656238 | EPI592032 | EPI1090560 | EPI888022 |  | EPI1092297 | EPI1155629 | EPI1191052 | EPI654399 | EPI1092043 | EPI1092624 |
| EPI692159 | EPI656139 | EPI497854 | EPI979627 | EPI591393 | EPI592562 | EPI628683 | EPI1102407 |  | EPI1092759 | EPI596718 | EPI1191053 | EPI654370 | EPI1091755 | EPI1092732 |
| EPI457797 | EPI627648 | EPI447865 | EPI1102691 | EPI591380 | EPI592411 | EPI656477 | EPI1102359 |  | EPI1093107 | EPI1155612 | EPI942197 | EPI654265 | EPI1093081 | EPI1092678 |
| EPI457821 | EPI656202 | EPI559490 | EPI1102699 | EPI591382 | EPI592074 | EPI566103 | EPI1022659 |  | EPI1093149 | EPI1055462 | EPI1092872 | EPI654376 | EPI1091965 | EPI1093074 |
| EPI457813 | EPI597169 | EPI443031 | EPI1102803 | EPI591255 | EPI592390 | EPI1090656 | EPI1013251 |  | EPI1055802 | EPI654375 | EPI1092698 | EPI1092131 | EPI1093093 | EPI1091844 |
| EPI457805 | EPI578100 | EPI441601 | EPI1102715 | EPI591400 | EPI592173 | EPI1090632 | EPI1090393 |  | EPI1191061 | EPI654270 | EPI1091714 | EPI681118 | EPI1093171 | EPI1092768 |
| EPI457829 | EPI596921 | EPI447944 | EPI1103853 | EPI591333 | EPI592053 | EPI1090664 | EPI971294 |  | EPI1055756 | EPI654380 | EPI1091720 | EPI681121 | EPI1103186 | EPI683106 |
| EPI440701 | EPI597072 | EPI613834 | EPI922308 | EPI591264 | EPI592039 | EPI626980 | EPI971470 |  | EPI1093221 | EPI654288 | EPI1093190 | EPI681116 | EPI1092787 | EPI942294 |
| EPI471835 | EPI593225 | EPI447778 | EPI1103236 | EPI591328 | EPI592341 | EPI1090728 | EPI971478 |  | EPI942257 | EPI1092694 | EPI942915 | EPI681117 | EPI1091977 | EPI942287 |
| EPI460535 | EPI707955 | EPI443640 | EPI1104117 | EPI591322 | EPI592433 | EPI627076 | EPI1013211 |  | EPI1092549 | EPI1092136 | EPI681073 | EPI681128 | EPI1092757 | EPI1092714 |
| EPI583207 | EPI596805 | EPI452265 | EPI1104133 | EPI591437 | EPI592060 | EPI627084 | EPI1101969 |  | EPI1092951 | EPI1098455 | EPI942204 | EPI681130 | EPI682977 | EPI1092072 |
| EPI509086 | EPI592188 | EPI452257 | EPI1104157 | EPI591263 | EPI592383 | EPI628771 | EPI1013259 |  | EPI1092699 | EPI1209314 | EPI1055481 | EPI681123 | EPI654198 | EPI1093200 |
| EPI477307 | EPI656163 | EPI515459 | EPI971146 | EPI591403 | EPI535188 | EPI628819 | EPI1100425 |  | EPI1191063 | EPI1209330 | EPI1055489 | EPI1176516 | EPI1055606 | EPI1091808 |
| EPI692157 | EPI656085 | EPI515452 | EPI971154 | EPI591335 | EPI628257 | EPI628611 | EPI627171 |  | EPI1093203 | EPI492290 | EPI1055722 | EPI529251 | EPI1055636 | EPI1092930 |
| EPI583214 | EPI596761 | EPI515475 | EPI1102291 | EPI628756 | EPI592224 | EPI628587 | EPI628826 |  | EPI1092069 | EPI492283 | EPI1055676 | EPI652935 | EPI1055642 | EPI1092924 |
| EPI476480 | EPI509879 | EPI515467 | EPI1103321 | EPI1102305 | EPI535172 | EPI628571 | EPI1090553 |  | EPI1092075 | EPI838023 | EPI1055668 | EPI573385 | EPI681159 | EPI1092954 |
| EPI447618 | EPI593197 | EPI516359 | EPI1104125 | EPI627828 | EPI628305 | EPI628579 | EPI628794 |  | EPI1091811 | EPI529270 | EPI1055684 | EPI846032 | EPI1092469 | EPI1092960 |
| EPI457433 | EPI597079 | EPI445909 | EPI1101709 | EPI627437 | EPI627801 | EPI627092 | EPI971398 |  | EPI1092879 | EPI573389 | EPI1055809 | EPI846040 | EPI1092313 | EPI1092348 |
| EPI583191 | EPI597423 | EPI530822 | EPI1101765 | EPI591531 | EPI627809 | EPI1101450 | EPI888030 |  | EPI1093179 | EPI1091956 | EPI1055715 | EPI654346 | EPI1093129 | EPI1092948 |
| EPI583184 | EPI578107 | EPI637069 | EPI1101749 | EPI591518 | EPI627873 | EPI627164 | EPI1104057 |  | EPI1093185 | EPI1091944 | EPI1055747 | EPI654352 | EPI1091953 | EPI1092174 |
| EPI692167 | EPI578226 | EPI447672 | EPI1101741 | EPI566096 | EPI627881 | EPI1090240 | EPI1103377 |  | EPI1091775 | EPI1091962 | EPI1055497 | EPI652949 | EPI1091941 | EPI1055635 |
| EPI591857 | EPI597429 | EPI620166 | EPI1101757 | EPI627908 | EPI627897 | EPI628651 | EPI1100465 |  | EPI1092939 | EPI569592 | EPI1055637 | EPI654358 | EPI1091959 | EPI1055495 |
| EPI447620 | EPI707950 | EPI509055 | EPI1104093 | EPI627916 | EPI627817 | EPI628739 | EPI1100473 |  | EPI1092969 | EPI680702 | EPI1055708 | EPI652938 | EPI1092667 | EPI1055658 |
| EPI443659 | EPI578114 | EPI447728 | EPI1104069 | EPI591471 | EPI627889 | EPI627004 | EPI1101361 |  | EPI1055602 | EPI680710 | EPI1055515 | EPI652941 | EPI1092709 | EPI1055674 |
| EPI692171 | EPI597586 | EPI461483 | EPI1102563 | EPI591539 | EPI592152 | EPI833708 | EPI1101401 |  | EPI1092537 | EPI680711 | EPI1055700 | EPI652937 | EPI681163 | EPI1055666 |
| EPI447607 | EPI597455 | EPI447707 | EPI1104077 | EPI566112 | EPI592238 | EPI628075 | EPI1101377 |  | EPI1055490 | EPI844494 | EPI1055629 | EPI652942 | EPI1091797 | EPI1055682 |
| EPI615186 | EPI597401 | EPI509079 | EPI1104149 | EPI1090596 | EPI592586 | EPI627068 | EPI1101385 |  | EPI1092351 | EPI681070 | EPI1055793 | EPI652939 | EPI1191025 | EPI1092900 |
| EPI442710 | EPI593232 | EPI613775 | EPI971290 | EPI566056 | EPI592259 | EPI656389 | EPI1101417 |  | EPI1092657 | EPI681072 | EPI1055601 | EPI652940 | EPI1055509 | EPI1092354 |
| EPI447604 | EPI1090183 | EPI620198 | EPI1104141 | EPI627341 | EPI592626 | EPI1100498 | EPI1101393 |  | EPI1098527 | EPI654472 | EPI1055617 | EPI652946 | EPI1055503 | EPI1055690 |
| EPI1168873 | EPI592203 | EPI613820 | EPI1104109 | EPI627349 | EPI592600 | EPI1100506 | EPI1101369 |  | EPI1098528 | EPI838033 | EPI1055692 | EPI652950 | EPI1055470 | EPI1055807 |
| EPI628416 | EPI578141 | EPI619994 | EPI971138 | EPI566048 | EPI592548 | EPI1057923 | EPI1090449 |  | EPI1092651 | EPI729796 | EPI1055660 | EPI652943 | EPI1055475 | EPI1055706 |
| EPI708071 | EPI1090343 | EPI613768 | EPI1104053 | EPI627325 | EPI592217 | EPI627452 | EPI1101409 |  | EPI1092639 | EPI1092688 | EPI1055801 | EPI1091933 | EPI1055746 | EPI1055815 |
| EPI1055424 | EPI597035 | EPI467302 | EPI1104101 | EPI627317 | EPI592555 | EPI627284 | EPI1022627 |  | EPI1092645 | EPI1191055 | EPI1092932 | EPI1093211 | EPI1055691 | EPI1055627 |
| EPI708085 | EPI707947 | EPI531124 | EPI1101517 | EPI1090604 | EPI592166 | EPI941050 | EPI1090513 |  | EPI942267 | EPI1191056 | EPI1092956 | EPI681124 | EPI1055776 | EPI1091712 |
| EPI628808 | EPI592195 | EPI620184 | EPI1101797 | EPI566064 | EPI592289 | EPI895525 | EPI1055410 |  | EPI1092711 | EPI1092940 | EPI1092962 | EPI1093193 | EPI1055779 | EPI1091718 |
| EPI1090245 | EPI707948 | EPI613813 | EPI1104029 | EPI627501 | EPI593154 | EPI628723 | EPI1090521 |  | EPI1091727 | EPI1092970 | EPI1092926 | EPI1092677 | EPI1055506 | EPI1093188 |
| EPI1090229 | EPI593350 | EPI439504 | EPI1104037 | EPI654134 | EPI593161 | EPI628347 | EPI1103387 |  | EPI1091781 | EPI1103230 | EPI1092878 | EPI1093073 | EPI1055731 | EPI1055720 |
| EPI1090213 | EPI593321 | EPI497862 | EPI1104085 | EPI1090204 | EPI592123 | EPI535126 | EPI1013235 |  | EPI942265 | EPI1055517 | EPI1093178 | EPI1091705 | EPI1092235 | EPI1055615 |
| EPI1090221 | EPI593357 | EPI447877 | EPI1102227 | EPI628436 | EPI592619 | EPI593247 | EPI1090401 |  | EPI1092945 | EPI1055639 | EPI1093184 | EPI1091771 | EPI1093243 | EPI1092366 |
| EPI627369 | EPI656218 | EPI613782 | EPI1102219 | EPI1090332 | EPI628065 | EPI592635 | EPI971254 |  | EPI1091715 | EPI1055631 | EPI1093214 | EPI1092935 | EPI1092355 | EPI1092738 |
| EPI769583 | EPI656249 | EPI447742 | EPI1100965 | EPI1090380 | EPI627522 | EPI593281 | EPI1101025 |  | EPI1091721 | EPI1055803 | EPI1091810 | EPI1092965 | EPI1092217 | EPI1093038 |
| EPI627361 | EPI1090255 | EPI446963 | EPI1104045 | EPI627964 | EPI627418 | EPI576550 | EPI888006 |  | EPI1055498 | EPI1092790 | EPI1092494 | EPI680838 | EPI1092901 | EPI1092672 |
| EPI769639 | EPI866576 | EPI447961 | EPI1104013 | EPI656034 | EPI627426 | EPI1090456 | EPI866555 |  | EPI1055810 | EPI1092550 | EPI1092656 | EPI680839 | EPI729782 | EPI1092744 |
| EPI593244 | EPI872957 | EPI620084 | EPI1101693 | EPI656050 | EPI627546 | EPI532304 | EPI866547 |  | EPI1055638 | EPI1092706 | EPI1098520 | EPI680855 | EPI944105 | EPI1092426 |
| EPI692631 | EPI972256 | EPI504721 | EPI1101701 | EPI655994 | EPI596948 | EPI628083 | EPI1101489 |  | EPI1055685 | EPI1092604 | EPI1098519 | EPI680858 | EPI1055648 | EPI729791 |
| EPI1090453 | EPI1101646 | EPI504731 | EPI1101989 | EPI627988 | EPI597394 | EPI541777 | EPI1104001 |  | EPI1055669 | EPI1098526 | EPI1098521 | EPI680862 | EPI1055645 | EPI1092636 |
| EPI1090839 | EPI1040450 | EPI448933 | EPI1101997 | EPI628124 | EPI592660 | EPI895524 | EPI887982 |  | EPI1055709 | EPI1092388 | EPI1092536 | EPI681125 | EPI1055651 | EPI1092660 |
| EPI708129 | EPI1036703 | EPI439495 | EPI1101733 | EPI655930 | EPI592137 | EPI578032 | EPI888054 |  | EPI1055661 | EPI1092910 | EPI1092176 | EPI681129 | EPI1092601 | EPI942282 |
| EPI592632 | EPI979516 | EPI568646 | EPI1100733 | EPI628484 | EPI592640 | EPI628283 | EPI1103993 |  | EPI1055794 | EPI1092916 | EPI1092068 | EPI654467 | EPI1055754 | EPI1067637 |
| EPI593278 | EPI972300 | EPI613796 | EPI1101781 | EPI655890 | EPI593189 | EPI656333 | EPI1100825 |  | EPI1055611 | EPI1098454 | EPI1093202 | EPI681127 | EPI1093249 | EPI1076647 |
| EPI656242 | EPI972262 | EPI613789 | EPI1102005 | EPI655906 | EPI592130 | EPI1090842 | EPI1100433 |  | EPI1055723 | EPI1092928 | EPI1092254 | EPI1092719 | EPI1092763 | EPI1092846 |
| EPI1090533 | EPI887835 | EPI613827 | EPI1101981 | EPI1090428 | EPI592447 | EPI628131 | EPI1090745 |  | EPI1055630 | EPI1092934 | EPI1092602 | EPI654491 | EPI1055714 | EPI1092864 |
| EPI593405 | EPI930811 | EPI620117 | EPI1101965 | EPI1090436 | EPI592231 | EPI1090480 | EPI888038 |  | EPI1055677 | EPI1092958 | EPI1092812 | EPI1092971 | EPI1055792 | EPI1092852 |
| EPI593548 | EPI972253 | EPI620134 | EPI1100773 | EPI655970 | EPI596914 | EPI628243 | EPI1090297 |  | EPI1055693 | EPI1092964 | EPI1092902 | EPI1092977 | EPI1055514 | EPI1092828 |
| EPI593469 | EPI1101558 | EPI457730 | EPI1101501 | EPI655866 | EPI597482 | EPI1090472 | EPI888014 |  | EPI729800 | EPI1092880 | EPI1092356 | EPI1034466 | EPI1055721 | EPI1092858 |
| EPI593497 | EPI730494 | EPI440690 | EPI887746 | EPI627725 | EPI593646 | EPI443662 | EPI1101465 |  | EPI1092975 | EPI1093180 | EPI1092392 | EPI1034467 | EPI1055628 | EPI1092762 |
| EPI1090645 | EPI972238 | EPI620127 | EPI887714 | EPI627653 | EPI592477 | EPI628379 | EPI1101801 |  | EPI1092981 | EPI1093186 | EPI1092668 | EPI838102 | EPI1055488 | EPI1092876 |
| EPI708004 | EPI1101614 | EPI620141 | EPI887722 | EPI627741 | EPI593653 | EPI628179 | EPI888046 |  | EPI1055618 | EPI1092664 | EPI1093256 | EPI1034468 | EPI1092811 | EPI1076690 |
| EPI708013 | EPI979604 | EPI457770 | EPI887826 | EPI627669 | EPI592159 | EPI628275 | EPI1102247 |  | EPI942901 | EPI1091776 | EPI942889 | EPI1034469 | EPI1091713 | EPI1076666 |
| EPI708006 | EPI1013272 | EPI477399 | EPI887858 | EPI656120 | EPI592454 | EPI531469 | EPI1101537 |  | EPI1092387 | EPI942908 | EPI1092242 | EPI1034471 | EPI1091719 | EPI1076698 |
| EPI708002 | EPI979596 | EPI620103 | EPI887874 | EPI656167 | EPI592102 | EPI531798 | EPI1103953 |  | EPI1092873 | EPI1067640 | EPI1092788 | EPI1034470 | EPI1092655 | EPI1076658 |
| EPI708000 | EPI972265 | EPI620191 | EPI1102355 | EPI509141 | EPI592440 | EPI628187 | EPI1013243 |  | EPI1092585 | EPI1092466 | EPI942192 | EPI838096 | EPI1055737 | EPI1076682 |
| EPI708009 | EPI972269 | EPI457634 | EPI1102371 | EPI509130 | EPI1057913 | EPI895520 | EPI1101545 |  | EPI1092591 | EPI942235 | EPI729794 | EPI729786 | EPI1055741 | EPI1076674 |
| EPI708003 | EPI972267 | EPI471848 | EPI1103246 | EPI656175 | EPI627362 | EPI627588 | EPI979567 |  | EPI1091763 | EPI1076652 | EPI1092974 | EPI729787 | EPI1092973 | EPI1092390 |
| EPI708005 | EPI1101574 | EPI590923 | EPI1101173 | EPI627948 | EPI1090214 | EPI656285 | EPI866539 |  | EPI1055748 | EPI1092946 | EPI1092980 | EPI838117 | EPI1092979 | EPI1092708 |
| EPI708001 | EPI1100790 | EPI447890 | EPI1101181 | EPI656018 | EPI1090222 | EPI628427 | EPI1102025 |  | EPI1055716 | EPI1091764 | EPI1091972 | EPI1092497 | EPI1092493 | EPI1055599 |
| EPI1090733 | EPI1100798 | EPI447665 | EPI1100549 | EPI627972 | EPI769584 | EPI1101250 | EPI1022611 |  | EPI1093215 | EPI1092586 | EPI1092710 | EPI1092743 | EPI1092643 | EPI1055609 |
| EPI592592 | EPI1101638 | EPI441801 | EPI1102603 | EPI656026 | EPI769640 | EPI979584 | EPI971422 |  | EPI1092495 | EPI1092592 | EPI1093208 | EPI1093037 | EPI1055816 | EPI1092648 |
| EPI708070 | EPI972254 | EPI443039 | EPI1100981 | EPI656066 | EPI560399 | EPI1101274 | EPI971430 |  | EPI1191064 | EPI1092718 | EPI1092758 | EPI1092905 | EPI1055808 | EPI1092696 |
| EPI592460 | EPI997160 | EPI447686 | EPI1102093 | EPI627940 | EPI627354 | EPI1101266 | EPI1100953 |  | EPI1191065 | EPI1092226 | EPI1091726 | EPI1092911 | EPI1092535 | EPI1092642 |
| EPI708105 | EPI656401 | EPI447749 | EPI1100533 | EPI656010 | EPI628225 | EPI1102624 | EPI1090497 |  | EPI1092603 | EPI1092256 | EPI1091780 | EPI1092227 | EPI1092697 | EPI1092654 |
| EPI592274 | EPI627056 | EPI545786 | EPI1101141 | EPI627677 | EPI535196 | EPI656001 | EPI1102439 |  | EPI1092171 | EPI844500 | EPI1092662 | EPI1191040 | EPI1093213 | EPI1092666 |
| EPI627441 | EPI656441 | EPI663461 | EPI887786 | EPI627996 | EPI576611 | EPI593149 | EPI1102727 |  | EPI1092321 | EPI1092160 | EPI1092638 | EPI1191041 | EPI1092175 | EPI1191042 |
| EPI592281 | EPI974539 | EPI447951 | EPI1103214 | EPI656002 | EPI628369 | EPI628211 | EPI1101945 |  | EPI1092765 | EPI1092220 | EPI1091756 | EPI1103527 | EPI1055616 | EPI1191044 |
| EPI593541 | EPI1101630 | EPI542309 | EPI1102101 | EPI656074 | EPI533260 | EPI627436 | EPI1102583 |  | EPI1076651 | EPI1093228 | EPI1092386 | EPI1103519 | EPI1055800 | EPI1092810 |
| EPI628704 | EPI972258 | EPI576599 | EPI1102211 | EPI627773 | EPI1057953 | EPI628619 | EPI1102511 |  | EPI1076667 | EPI1092250 | EPI1076648 | KP055062 | EPI1055480 | EPI1092534 |
| EPI628720 | EPI1101582 | EPI457626 | EPI1100997 | EPI628420 | EPI628273 | EPI656357 | EPI971310 |  | EPI1076659 | KP055059 | EPI942207 | KM609709 | EPI1055496 | EPI1092600 |
| EPI592508 | EPI1040470 | EPI457642 | EPI1100989 | EPI627796 | EPI628281 | EPI580382 | EPI1102575 |  | EPI1076675 | KC821067 | EPI1091978 | KM609703 | EPI1091725 | EPI1092414 |
| EPI592577 | EPI1040454 | EPI443648 | EPI1102483 | EPI656098 | EPI1090470 | EPI628171 | EPI971454 |  | EPI1076691 | KC821069 | EPI1093106 | KM609680 | EPI1091779 | EPI1092168 |
| EPI593146 | EPI626992 | EPI507144 | EPI1103075 | EPI577886 | EPI627554 | EPI1090736 | EPI1101953 |  | EPI1092723 | KC821068 | EPI1093118 | KM609707 | EPI1098504 | EPI1092318 |
| EPI592484 | EPI656329 | EPI447763 | EPI1102331 | EPI509884 | EPI533244 | EPI627228 | EPI1102711 |  | EPI1091937 | KC821072 | EPI1093220 | KM609681 | EPI1098503 | EPI1098518 |
| EPI592515 | EPI627208 | EPI508924 | EPI887954 | EPI577885 | EPI628137 | EPI592580 | EPI1074598 |  | EPI1092357 | KC821074 | EPI1092692 | KC821143 | EPI1098505 | EPI1191047 |
| EPI592244 | EPI656281 | EPI457754 | EPI888066 | EPI627269 | EPI628033 | EPI443654 | EPI1103897 |  | EPI1092903 | KC821073 | EPI1092218 | KC821158 | EPI1092505 | EPI1191046 |
| EPI703994 | EPI656289 | EPI691643 | EPI1103192 | EPI656270 | EPI594291 | EPI628363 | EPI926827 |  | EPI1092669 | KM609825 | EPI1092650 | KC821144 | EPI1092511 | EPI944112 |
| EPI708079 | EPI580365 | EPI691702 | EPI1100589 | EPI626989 | EPI627530 | EPI592463 | EPI869667 |  | EPI1098530 | KC821071 | EPI1092644 | KC821145 | EPI1092391 | EPI1092492 |
| EPI708076 | EPI656497 | EPI691645 | EPI1101829 | EPI1040447 | EPI535116 | EPI628163 | EPI1101665 |  | KC821030 | KC821085 | EPI1092950 | KC821168 | EPI1055683 | EPI1055745 |
| EPI682918 | EPI628647 | EPI691704 | EPI1103925 | EPI1101643 | EPI628289 | EPI1090360 | EPI1101673 |  | KC821035 | KC821078 | EPI1092350 | KC821162 | EPI1055654 | EPI942894 |
| EPI682919 | EPI1090327 | EPI691700 | EPI1100909 | EPI866573 | EPI533251 | EPI592284 | EPI874332 |  | KC821032 | KC821066 | KP055060 | KM609693 | EPI1055699 | EPI1091934 |
| EPI580371 | EPI887979 | EPI691647 | EPI1102907 | EPI926829 | EPI627594 | EPI592595 | EPI977389 |  | KC821031 | KC821080 | KC821107 | KM609705 | EPI1055787 | EPI1092240 |
| EPI692628 | EPI972245 | EPI447898 | EPI1102915 | EPI730491 | EPI627570 | EPI592247 | EPI926843 |  | KP055058 | KC821086 | KC821110 | KC821147 | EPI1055675 | EPI1092972 |
| EPI1102309 | EPI972283 | EPI477321 | EPI1103224 | EPI1101611 | EPI535180 | EPI592511 | EPI1101481 |  | KC821037 | KC821079 | KC821109 | KC821148 | EPI1055707 | EPI1092978 |
| EPI628760 | EPI887843 | EPI691644 | EPI1103805 | EPI628388 | EPI628057 | EPI656349 | EPI971382 |  | KC821034 | KC821096 | KC821106 | KC821146 | EPI1055667 | EPI1098517 |
| EPI1090285 | EPI972247 | EPI457714 | EPI887778 | EPI656382 | EPI535164 | EPI583075 | EPI971390 |  | KC821063 | KC821098 | KC821111 | KC821153 | EPI1055659 | EPI1098515 |
| EPI627912 | EPI972249 | EPI691639 | EPI971218 | EPI1100779 | EPI594228 | EPI592487 | EPI1100817 |  | KC821045 | KC821095 | KC821132 | KC821152 | EPI1092349 | EPI1098516 |
| EPI627920 | EPI884221 | EPI691640 | EPI887730 | EPI1101771 | EPI542094 | EPI593170 | EPI1100833 |  | KM609853 | KC821099 | KC821108 | KC821154 | EPI1092637 | EPI1093254 |
| EPI566100 | EPI1101654 | EPI691703 | EPI1103267 | EPI580370 | EPI627538 | EPI627915 | EPI961187 |  | KM609863 | KC821097 | KC821137 | KF059305 | EPI1191027 | KP055065 |
| EPI708128 | EPI1040486 | EPI691641 | EPI971314 | EPI580326 | EPI594354 | EPI592277 | EPI1022683 |  | KM609841 | KC821093 | KC821105 | KC821156 | KP055064 | KF059315 |
| EPI708103 | EPI972281 | EPI691638 | EPI1102515 | EPI577366 | EPI528331 | EPI628731 | EPI1100857 |  | KM609867 | KC821094 | KC821123 | KC821169 | KC821181 | KM609749 |
| EPI708043 | EPI1090415 | EPI691646 | EPI971202 | EPI656414 | EPI528339 | EPI656421 | EPI1100865 |  | KM609840 | KC821092 | KC821103 | KC821157 | KC821180 | KM609743 |
| EPI708087 | EPI972274 | EPI443664 | EPI971210 | EPI1101531 | EPI528355 | EPI627380 | EPI1022603 |  | KM609869 | KM609808 | KC821119 | KC821150 | KC821185 | KM609720 |
| EPI593174 | EPI972271 | EPI583182 | EPI971178 | EPI627021 | EPI627386 | EPI593177 | EPI1054128 |  | KM609871 | KM609815 | KC821120 | KC821160 | KC821184 | KM609721 |
| EPI593381 | EPI974540 | EPI691637 | EPI971170 | EPI656334 | EPI627562 | EPI580390 | EPI1067569 |  | KM609849 | KM609811 | KC821124 | KM609702 | KC821182 | KM609747 |
| EPI708052 | EPI972235 | EPI691650 | EPI971194 | EPI656286 | EPI594305 | EPI627116 | EPI1018098 |  | KM609868 | KC821091 | KC821125 | KM609704 | KC821183 | KM609728 |
| EPI708100 | EPI972293 | EPI591855 | EPI971162 | EPI1103979 | EPI628337 | EPI1090544 | EPI1018238 |  | KC821048 | KM609830 | KC821130 | KM609716 | KC821198 | KM609731 |
| EPI708124 | EPI1101662 | EPI583189 | EPI1100613 | EPI1036684 | EPI583912 | EPI627827 | EPI1040473 |  | KC821039 | KC821075 | KC821136 | KF059304 | KM609630 | KM609735 |
| EPI708104 | EPI1101598 | EPI457706 | EPI1102499 | EPI1102168 | EPI627610 | EPI628691 | EPI1018138 |  | KC821038 | KM609833 | KC821134 | KF059307 | KC821194 | KM609723 |
| EPI708093 | EPI972286 | EPI583205 | EPI1102659 | EPI580346 | EPI594186 | EPI592518 | EPI1022619 |  | KC821044 | KF059335 | KC821135 | KF059306 | KC821186 | KM609733 |
| EPI708097 | EPI974541 | EPI457818 | EPI1101525 | EPI656358 | EPI583900 | EPI628659 | EPI1013227 |  | KC821042 | KM609816 | KC821122 | KM609687 | KC821195 | KM609736 |
| EPI708092 | EPI972288 | EPI471849 | EPI1102651 | EPI580266 | EPI592313 | EPI656485 | EPI1018106 |  | KC821049 | KM609805 | KC821104 | KC821161 | KC821189 | KM609755 |
| EPI708106 | EPI972291 | EPI457802 | EPI1102667 | EPI566088 | EPI594333 | EPI1101290 | EPI1018122 |  | KC821040 | KM609804 | KC821133 | KM609710 | KC821196 | KM609725 |
| EPI708047 | EPI972233 | EPI440698 | EPI1100893 | EPI656254 | EPI583926 | EPI1101618 | EPI1040457 |  | KC821033 | KM609802 | KC821128 | KC821159 | KC821188 | KM609724 |
| EPI708051 | EPI974542 | EPI457826 | EPI1101357 | EPI566668 | EPI594200 | EPI656373 | EPI1022643 |  | KC821043 | KM609806 | KM609790 | KC821149 | KC821187 | KM609742 |
| EPI708044 | EPI447632 | EPI691642 | EPI1101333 | EPI580354 | EPI594158 | EPI576545 | EPI1018245 |  | KC821046 | KM609831 | KC821126 | KC821140 | KC821202 | KF059321 |
| EPI708101 | EPI451274 | EPI691701 | EPI1101349 | EPI628708 | EPI594298 | EPI627340 | EPI1010103 |  | KC821029 | KM609828 | KC821116 | KC821163 | KC821200 | KM609756 |
| EPI708120 | EPI1090263 | EPI691648 | EPI1101325 | EPI580290 | EPI594221 | EPI566047 | EPI979607 |  | KF059345 | KM609809 | KF059325 | KC821141 | KC821192 | KM609744 |
| EPI708086 | EPI1090271 | EPI457698 | EPI1101317 | EPI656294 | EPI628041 | EPI627324 | EPI1054112 |  | KC821047 | KM609819 | KC821121 | KC821142 | KC821201 | KM609746 |
| EPI708088 | EPI532295 | EPI583212 | EPI1101341 | EPI559413 | EPI594144 | EPI627316 | EPI1018146 |  | KM609848 | KM609810 | KC821127 | KC821167 | KC821178 | KM609754 |
| EPI593167 | EPI497838 | EPI447693 | EPI887682 | EPI580298 | EPI594312 | EPI627500 | EPI973367 |  | KM609855 | KM609818 | KF059327 | KC821166 | KC821210 | KM609737 |
| EPI708126 | EPI576572 | EPI477322 | EPI1101117 | EPI566032 | EPI594235 | EPI566055 | EPI1018262 |  | KM609851 | KM609821 | KF059324 | KC821155 | KC821199 | KF059318 |
| EPI708116 | EPI457429 | EPI457810 | EPI887642 | EPI580312 | EPI594256 | EPI1090600 | EPI1018154 |  | KM609852 | KM609814 | KC821118 | KC821174 | KM609629 | KM609757 |
| EPI708090 | EPI507149 | EPI447679 | EPI1102085 | EPI580336 | EPI594263 | EPI566063 | EPI1018170 |  | KF059347 | KM609839 | KC821117 | KC821165 | KM609603 | KM609740 |
| EPI708114 | EPI457868 | EPI497891 | EPI887818 | EPI580274 | EPI594347 | EPI566111 | EPI1100841 |  | KF059344 | KM609834 | KM609795 | KC821164 | KF059285 | KM609752 |
| EPI708123 | EPI499886 | EPI457866 | EPI887906 | EPI580282 | EPI594137 | EPI1090608 | EPI1018130 |  | KM609856 | KC821081 | KM609789 | KC821151 | KM609615 | KM609739 |
| EPI708089 | EPI447709 | EPI457874 | EPI1103027 | EPI654149 | EPI594207 | EPI627348 | EPI1018090 |  | KM609845 | KC821077 | KM609763 | KM609688 | KM609611 | KM609753 |
| EPI708049 | EPI446747 | EPI576597 | EPI1103329 | EPI656462 | EPI627402 | EPI628627 | EPI1018082 |  | KM609844 | KC821076 | KM609768 | KM609695 | KC821197 | KM609729 |
| EPI708112 | EPI451333 | EPI457882 | EPI1103933 | EPI627189 | EPI583906 | EPI628635 | EPI1018196 |  | KC821062 | KM609829 | KM609772 | KM609692 | KC821191 | KM609730 |
| EPI708113 | EPI497920 | EPI576598 | EPI979523 | EPI656310 | EPI627442 | EPI1090584 | EPI1022691 |  | KC821057 | KM609823 | KM609775 | KM609691 | KC821190 | KM609748 |
| EPI708118 | EPI460777 | EPI447756 | EPI887970 | EPI656366 | EPI583919 | EPI628411 | EPI1022635 |  | KC821041 | KM609801 | KM609771 | KM609717 | KC821208 | KM609741 |
| EPI708099 | EPI446965 | EPI515785 | EPI887922 | EPI627069 | EPI592320 | EPI627236 | EPI1022702 |  | KC821052 | KM609827 | KM609783 | KM609683 | KC821204 | KM609734 |
| EPI708048 | EPI439500 | EPI515775 | EPI887914 | EPI656390 | EPI594151 | EPI447803 | EPI1022651 |  | KC821053 | KM609800 | KM609760 | KM609715 | KM609608 | KM609738 |
| EPI708050 | EPI448938 | EPI450847 | EPI922027 | EPI1090356 | EPI594340 | EPI447729 | EPI1022675 |  | KC821051 | KM609813 | KM609787 | KM609712 | KC821203 | KM609759 |
| EPI708046 | EPI568652 | EPI1090794 | EPI1103837 | EPI580378 | EPI594179 | EPI451268 | EPI1040893 |  | KM609870 | KC821083 | KM609761 | KM609685 | KM609604 | KM609758 |
| EPI708107 | EPI581860 | EPI532311 | EPI887794 | EPI580386 | EPI594326 | EPI457760 | EPI692012 |  | KC821059 | KC821082 | KM609767 | KM609684 | KM609605 | KM609722 |
| EPI566108 | EPI576570 | EPI457738 | EPI1103203 | EPI627013 | EPI583933 | EPI582343 | EPI592234 |  | KC821060 | KC821100 | KM609786 | KM609700 | KM609612 | KM609726 |
| EPI627832 | EPI457628 | EPI457762 | EPI979531 | EPI1101019 | EPI594172 | EPI583111 | EPI1090209 |  | KC821061 | KC821084 | KM609798 | KM609689 | KF059287 | KM609750 |
| EPI1090597 | EPI515780 | EPI442718 | EPI979539 | EPI628700 | EPI594249 | EPI628355 | EPI663496 |  | KC821050 | KC821070 | KM609777 | KM609708 | KF059284 | KM609745 |
| EPI1090605 | EPI515790 | EPI442715 | EPI1103035 | EPI628716 | EPI628233 | EPI627923 | EPI615179 |  | KM609854 | KM609803 | KF059326 | KM609694 | KM609622 | KF059319 |
| EPI627505 | EPI447695 | EPI457618 | EPI887618 | EPI628748 | EPI594165 | EPI627508 | EPI545792 |  | KM609861 | KM609820 | KF059328 | KM609711 | KM609624 | KM609727 |
| EPI627329 | EPI447653 | EPI576596 | EPI1103345 | EPI869669 | EPI594242 | EPI628763 | EPI663497 |  | KM609850 | KM609832 | KF059331 | KM609701 | KM609636 | KM609732 |
| EPI566052 | EPI497904 | EPI576594 | EPI1102611 | EPI1101667 | EPI627578 | EPI566134 | EPI576586 |  | KM609873 | KM609812 | KM609782 | KM609690 | KM609607 | KF059316 |
| EPI566068 | EPI447884 | EPI576595 | EPI1102619 | EPI1101675 | EPI594277 | EPI628539 | EPI497906 |  | KM609858 | KF059337 | KM609784 | KM609698 | KF059286 | KF059317 |
| EPI627321 | EPI490978 | EPI627606 | EPI887754 | EPI1018116 | EPI594270 | EPI1102968 | EPI509075 |  | KM609859 | KF059334 | KM609796 | KM609713 | KF059291 | KF059314 |
| EPI627345 | EPI503514 | EPI1090805 | EPI887946 | EPI1100787 | EPI594319 | EPI1100514 | EPI447948 |  | KM609846 | KM609826 | KC821129 | KM609699 | KF059288 |  |
| EPI627353 | EPI490970 | EPI443672 | EPI1101165 | EPI1101571 | EPI594284 | EPI1040446 | EPI542315 |  | KM609842 | KM609817 | KC821131 | KM609719 | KM609616 |  |
| EPI566060 | EPI503511 | EPI457778 | EPI1102979 | EPI1101595 | EPI627410 | EPI1101602 | EPI1102543 |  | KM609874 | KF059336 | KM609765 | KM609696 | KM609613 |  |
| EPI566116 | EPI453611 | EPI663460 | EPI1103290 | EPI1018216 | EPI628297 | EPI592226 | EPI457735 |  | KM609879 | KF059341 | KM609764 | KF059311 | KM609626 |  |
| EPI621120 | EPI447780 | EPI532310 | EPI1103278 | EPI1101635 | EPI535148 | EPI532307 | EPI1090801 |  | KM609875 | KM609822 | KM609773 | KF059308 | KM609638 |  |
| EPI621127 | EPI443036 | EPI447651 | EPI1103300 | EPI1101659 | EPI594214 | EPI682986 | EPI656484 |  | KM609843 | KM609824 | KM609774 | KM609686 | KM609634 |  |
| EPI627137 | EPI440096 | EPI457682 | EPI1103003 | EPI1040467 | EPI592180 | EPI457664 | EPI628618 |  | KM609847 | KM609836 | KM609781 | KM609682 | KM609617 |  |
| EPI708077 | EPI443029 | EPI457690 | EPI1101213 | EPI1090404 | EPI583940 | EPI895526 | EPI447683 |  | KM609860 | KM609807 | KM609770 | KM609718 | KC821206 |  |
| EPI621115 | EPI451325 | EPI509078 | EPI1102635 | EPI1022629 | EPI594193 | EPI895522 | EPI467317 |  | KM609872 | KM609835 | KM609778 | KM609706 | KC821193 |  |
| EPI566076 | EPI447815 | EPI663458 | EPI1101229 | EPI887840 | EPI627833 | EPI576613 | EPI926835 |  | KF059348 | KM609837 | KM609791 | KM609714 | KC821177 |  |
| EPI627041 | EPI447667 | EPI545800 | EPI887994 | EPI1090412 | EPI583894 | EPI1090821 | EPI1101553 |  | KM609866 |  | KM609788 | KM609697 | KM609625 |  |
| EPI628600 | EPI451255 | EPI663459 | EPI1101221 | EPI979609 | EPI1090582 | EPI509121 | EPI887830 |  | KM609857 |  | KM609769 |  | KM609627 |  |
| EPI626985 | EPI504715 | EPI545793 | EPI1101261 | EPI979593 | EPI1090558 | EPI587894 | EPI930810 |  | KM609878 |  | KM609779 |  | KM609600 |  |
| EPI656482 | EPI504722 | EPI663468 | EPI1101245 | EPI1013269 | EPI1090286 | EPI627180 | EPI559411 |  | KM609877 |  | KM609793 |  | KM609623 |  |
| EPI627169 | EPI442723 | EPI627925 | EPI887770 | EPI1101579 | EPI576543 | EPI447901 | EPI656404 |  | KF059351 |  | KM609799 |  | KM609601 |  |
| EPI628656 | EPI477404 | EPI628029 | EPI1101237 | EPI997216 | EPI566117 | EPI497839 | EPI627059 |  | KM609862 |  | KM609794 |  | KM609619 |  |
| EPI1090825 | EPI515461 | EPI628173 | EPI446450 | EPI960359 | EPI1090478 | EPI528349 | EPI628362 |  | KM609864 |  | KM609762 |  | KM609633 |  |
| EPI1101023 | EPI445914 | EPI628165 | EPI447836 | EPI1013221 | EPI628129 | EPI592291 | EPI930819 |  | KM609876 |  | KM609766 |  | KM609609 |  |
| EPI1102063 | EPI503953 | EPI628213 | EPI497863 | EPI960358 | EPI628345 | EPI592557 | EPI997221 |  | KM609865 |  | KM609785 |  | KM609628 |  |
| EPI887636 | EPI515469 | EPI490968 | EPI447734 | EPI926813 | EPI628177 | EPI532305 | EPI926811 |  |  |  | KM609780 |  | KM609610 |  |
| EPI1103967 | EPI515477 | EPI490976 | EPI447809 | EPI919603 | EPI628185 | EPI627396 | EPI960368 |  |  |  | KM609792 |  | KM609631 |  |
| EPI971308 | EPI452270 | EPI503505 | EPI439489 | EPI1101627 | EPI628377 | EPI627300 | EPI960369 |  |  |  | KM609776 |  | KM609621 |  |
| EPI628576 | EPI452262 | EPI468997 | EPI497855 | EPI1022645 | EPI531471 | EPI627420 | EPI919601 |  |  |  |  |  | KM609614 |  |
| EPI627089 | EPI503956 | EPI469000 | EPI447801 | EPI1101651 | EPI628425 | EPI627428 | EPI1013219 |  |  |  |  |  | KM609618 |  |
| EPI628592 | EPI447737 | EPI468998 | EPI497812 | EPI926845 | EPI576624 | EPI453481 | EPI887974 |  |  |  |  |  | KM609632 |  |
| EPI628616 | EPI441797 | EPI468999 | EPI447863 | EPI874334 | EPI628241 | EPI583133 | EPI1100785 |  |  |  |  |  | KM609620 |  |
| EPI627081 | EPI581853 | EPI509112 | EPI447850 | EPI977391 | EPI628081 | EPI469525 | EPI1100793 |  |  |  |  |  | KM609637 |  |
| EPI628584 | EPI583237 | EPI457722 | EPI467334 | EPI1040483 | EPI656188 | EPI654141 | EPI1101569 |  |  |  |  |  | KC821179 |  |
| EPI628752 | EPI655989 | EPI509095 | EPI453621 | EPI884218 | EPI656475 | EPI895527 | EPI887838 |  |  |  |  |  | KM609639 |  |
| EPI628776 | EPI581875 | EPI509110 | EPI639586 | EPI887832 | EPI1090534 | EPI583118 | EPI1040481 |  |  |  |  |  | KM609635 |  |
| EPI628824 | EPI628007 | EPI628389 | EPI572727 | EPI930806 | EPI627506 | EPI583105 | EPI884216 |  |  |  |  |  | KM609602 |  |
| EPI628744 | EPI457756 | EPI1090804 | EPI469530 | EPI1101555 | EPI566132 | EPI895528 | EPI1018114 |  |  |  |  |  | KM609606 |  |
| EPI1090717 | EPI447893 | EPI627518 | EPI468965 | EPI1013261 | EPI566069 | EPI895519 | EPI1101609 |  |  |  |  |  |  |  |
| EPI1101015 | EPI477309 | EPI1090186 | EPI569715 | EPI1022661 | EPI1102230 | EPI895521 | EPI979599 |  |  |  |  |  |  |  |
| EPI1090725 | EPI457796 | EPI656383 | EPI453602 | EPI457857 | EPI1102238 | EPI895523 | EPI1101649 |  |  |  |  |  |  |  |
| EPI682923 | EPI691761 | EPI628397 | EPI447920 | EPI457785 | EPI578030 | EPI628403 | EPI597485 |  |  |  |  |  |  |  |
| EPI1090237 | EPI691757 | EPI628157 | EPI469538 | EPI460766 | EPI656363 | EPI628019 | EPI627547 |  |  |  |  |  |  |  |
| EPI833705 | EPI447688 | EPI628205 | EPI926847 | EPI509125 | EPI656491 | EPI592480 | EPI628274 |  |  |  |  |  |  |  |
| EPI1101455 | EPI691767 | EPI531807 | EPI497827 | EPI568736 | EPI628641 | EPI592456 | EPI596951 |  |  |  |  |  |  |  |
| EPI692632 | EPI583209 | EPI656184 | EPI583165 | EPI1090628 | EPI627202 | EPI592132 | EPI656284 |  |  |  |  |  |  |  |
| EPI627009 | EPI457820 | EPI531816 | EPI628358 | EPI1090676 | EPI656275 | EPI593191 | EPI1090473 |  |  |  |  |  |  |  |
| EPI692614 | EPI691773 | EPI531813 | EPI535137 | EPI576839 | EPI1090326 | EPI592449 | EPI628226 |  |  |  |  |  |  |  |
| EPI627097 | EPI583186 | EPI531810 | EPI628318 | EPI576837 | EPI656323 | EPI592641 | EPI576614 |  |  |  |  |  |  |  |
| EPI656474 | EPI447723 | EPI453827 | EPI535161 | EPI576784 | EPI580364 | EPI592139 | EPI597418 |  |  |  |  |  |  |  |
| EPI627049 | EPI583193 | EPI497819 | EPI628326 | EPI576838 | EPI1090254 | EPI593163 | EPI654140 |  |  |  |  |  |  |  |
| EPI656458 | EPI471839 | EPI457430 | EPI535145 | EPI591303 | EPI627154 | EPI592442 | EPI628066 |  |  |  |  |  |  |  |
| EPI576012 | EPI477310 | EPI460772 | EPI628334 | EPI591304 | EPI1103880 | EPI592161 | EPI1102775 |  |  |  |  |  |  |  |
| EPI576046 | EPI691770 | EPI457439 | EPI1090675 | EPI591316 | EPI552400 | EPI592104 | EPI1102823 |  |  |  |  |  |  |  |
| EPI627265 | EPI457684 | EPI509063 | EPI1090659 | EPI591201 | EPI627178 | EPI592648 | EPI1102831 |  |  |  |  |  |  |  |
| EPI1171791 | EPI447702 | EPI447700 | EPI1090627 | EPI591311 | EPI628545 | EPI596916 | EPI1090721 |  |  |  |  |  |  |  |
| EPI576004 | EPI457844 | EPI467310 | EPI1090651 | EPI591194 | EPI628561 | EPI597396 | EPI1101017 |  |  |  |  |  |  |  |
| EPI627113 | EPI457716 | EPI447802 | EPI583087 | EPI591313 | EPI1101200 | EPI628227 | EPI1090729 |  |  |  |  |  |  |  |
| EPI656346 | EPI591859 | EPI447658 | EPI477408 | EPI591378 | EPI1101192 | EPI597484 | EPI1101009 |  |  |  |  |  |  |  |
| EPI628640 | EPI457804 | EPI497899 | EPI583071 | EPI591236 | EPI1101424 | EPI596950 | EPI1100745 |  |  |  |  |  |  |  |
| EPI627497 | EPI467774 | EPI447637 | EPI583121 | EPI591318 | EPI628673 | EPI592336 | EPI1101577 |  |  |  |  |  |  |  |
| EPI627297 | EPI691764 | EPI447824 | EPI583101 | EPI591199 | EPI628785 | EPI592034 | EPI1101633 |  |  |  |  |  |  |  |
| EPI627217 | EPI509060 | EPI447770 | EPI583114 | EPI591237 | EPI1101432 | EPI593500 | EPI1074606 |  |  |  |  |  |  |  |
| EPI1090197 | EPI457812 | EPI447794 | EPI447755 | EPI591317 | EPI628665 | EPI592055 | EPI1090305 |  |  |  |  |  |  |  |
| EPI566131 | EPI583216 | EPI585982 | EPI457723 | EPI591309 | EPI628777 | EPI592111 | EPI1013187 |  |  |  |  |  |  |  |
| EPI627313 | EPI457828 | EPI497907 | EPI443032 | EPI591200 | EPI583131 | EPI592413 | EPI1013195 |  |  |  |  |  |  |  |
| EPI627105 | EPI532271 | EPI447644 | EPI441600 | EPI591315 | EPI628017 | EPI592041 | EPI1013203 |  |  |  |  |  |  |  |
| EPI627225 | EPI691768 | EPI498095 | EPI447942 | EPI591308 | EPI628401 | EPI592076 | EPI1013171 |  |  |  |  |  |  |  |
| EPI627257 | EPI440700 | EPI447714 | EPI545794 | EPI591195 | EPI703995 | EPI592421 | EPI1013179 |  |  |  |  |  |  |  |
| EPI627241 | EPI691763 | EPI509094 | EPI628054 | EPI591198 | EPI626978 | EPI592118 | EPI1013163 |  |  |  |  |  |  |  |
| EPI627457 | EPI457692 | EPI443632 | EPI585983 | EPI591310 | EPI627122 | EPI592343 | EPI627355 |  |  |  |  |  |  |  |
| EPI941047 | EPI691758 | EPI450839 | EPI447793 | EPI591196 | EPI627026 | EPI592428 | EPI627299 |  |  |  |  |  |  |  |
| EPI566084 | EPI691766 | EPI447721 | EPI447769 | EPI591307 | EPI729452 | EPI592062 | EPI627475 |  |  |  |  |  |  |  |
| EPI628728 | EPI691772 | EPI497870 | EPI447822 | EPI591305 | EPI531803 | EPI592371 | EPI627331 |  |  |  |  |  |  |  |
| EPI627001 | EPI457708 | EPI497915 | EPI457643 | EPI591245 | EPI729460 | EPI592175 | EPI683111 |  |  |  |  |  |  |  |
| EPI656450 | EPI447646 | EPI460748 | EPI457659 | EPI591306 | EPI627458 | EPI592329 | EPI1090249 |  |  |  |  |  |  |  |
| EPI576064 | EPI691755 | EPI453647 | EPI447777 | EPI591234 | EPI592509 | EPI656405 | EPI566038 |  |  |  |  |  |  |  |
| EPI573630 | EPI691760 | EPI457842 | EPI576591 | EPI591256 | EPI627242 | EPI592435 | EPI627483 |  |  |  |  |  |  |  |
| EPI568233 | EPI691765 | EPI656287 | EPI469192 | EPI591253 | EPI1090198 | EPI592399 | EPI628370 |  |  |  |  |  |  |  |
| EPI656434 | EPI691762 | EPI566081 | EPI457675 | EPI591314 | EPI1090718 | EPI592378 | EPI627810 |  |  |  |  |  |  |  |
| EPI627233 | EPI691769 | EPI627238 | EPI447762 | EPI591197 | EPI1090726 | EPI592083 | EPI627874 |  |  |  |  |  |  |  |
| EPI576054 | EPI691771 | EPI769563 | EPI568751 | EPI591193 | EPI1101008 | EPI592385 | EPI1018217 |  |  |  |  |  |  |  |
| EPI656490 | EPI443669 | EPI769562 | EPI467303 | EPI591312 | EPI1090774 | EPI592364 | EPI1101657 |  |  |  |  |  |  |  |
| EPI627025 | EPI443661 | EPI769572 | EPI663471 | EPI553466 | EPI593406 | EPI592069 | EPI1100849 |  |  |  |  |  |  |  |
| EPI575995 | EPI509084 | EPI627462 | EPI509077 | EPI979640 | EPI593498 | EPI592564 | EPI1040465 |  |  |  |  |  |  |  |
| EPI656338 | EPI691759 | EPI627486 | EPI576592 | EPI1101443 | EPI593470 | EPI592392 | EPI1090409 |  |  |  |  |  |  |  |
| EPI576028 | EPI583286 | EPI628805 | EPI447636 | EPI627373 | EPI593549 | EPI592350 | EPI628282 |  |  |  |  |  |  |  |
| EPI576037 | EPI498799 | EPI627478 | EPI447876 | EPI1090484 | EPI592275 | EPI592406 | EPI592650 |  |  |  |  |  |  |  |
| EPI627121 | EPI583244 | EPI566041 | EPI460773 | EPI566104 | EPI592578 | EPI592097 | EPI592133 |  |  |  |  |  |  |  |
| EPI656378 | EPI583223 | EPI627334 | EPI508925 | EPI769582 | EPI683073 | EPI592357 | EPI1090649 |  |  |  |  |  |  |  |
| EPI628688 | EPI628111 | EPI627302 | EPI497916 | EPI769638 | EPI593382 | EPI592048 | EPI593385 |  |  |  |  |  |  |  |
| EPI656354 | EPI655917 | EPI627102 | EPI447950 | EPI1090212 | EPI593147 | EPI592027 | EPI593164 |  |  |  |  |  |  |  |
| EPI656426 | EPI577913 | EPI627222 | EPI457747 | EPI1090220 | EPI1100512 | EPI592090 | EPI593649 |  |  |  |  |  |  |  |
| EPI576021 | EPI581867 | EPI1090194 | EPI576593 | EPI627357 | EPI592485 | EPI628195 | EPI593656 |  |  |  |  |  |  |  |
| EPI656370 | EPI577927 | EPI566128 | EPI497908 | EPI627365 | EPI683072 | EPI628267 | EPI592457 |  |  |  |  |  |  |  |
| EPI627073 | EPI583300 | EPI627310 | EPI498096 | EPI560397 | EPI592461 | EPI628387 | EPI592450 |  |  |  |  |  |  |  |
| EPI656394 | EPI577899 | EPI1101772 | EPI457875 | EPI1018255 | EPI1090550 | EPI628147 | EPI592162 |  |  |  |  |  |  |  |
| EPI628632 | EPI654021 | EPI979638 | EPI457627 | EPI1101683 | EPI592653 | EPI656253 | EPI592656 |  |  |  |  |  |  |  |
| EPI627385 | EPI577989 | EPI1090570 | EPI542310 | EPI1102961 | EPI593175 | EPI626988 | EPI592642 |  |  |  |  |  |  |  |
| EPI627465 | EPI577996 | EPI627294 | EPI545787 | EPI1102945 | EPI592593 | EPI656190 | EPI593192 |  |  |  |  |  |  |  |
| EPI627305 | EPI583265 | EPI566097 | EPI447960 | EPI1102953 | EPI592282 | EPI627052 | EPI592443 |  |  |  |  |  |  |  |
| EPI1090357 | EPI577940 | EPI1103964 | EPI457779 | EPI1100723 | EPI592516 | EPI656461 | EPI592105 |  |  |  |  |  |  |  |
| EPI656322 | EPI506220 | EPI1101476 | EPI497871 | EPI1100739 | EPI592245 | EPI1100698 | EPI592140 |  |  |  |  |  |  |  |
| EPI1090541 | EPI515868 | EPI1104020 | EPI440691 | EPI1057964 | EPI593168 | EPI1102392 | EPI1102165 |  |  |  |  |  |  |  |
| EPI656266 | EPI654033 | EPI1100628 | EPI457883 | EPI1057916 | EPI887669 | EPI1102464 | EPI1036689 |  |  |  |  |  |  |  |
| EPI580379 | EPI581590 | EPI1100676 | EPI457851 | EPI1057956 | EPI887805 | EPI1101922 | EPI1103977 |  |  |  |  |  |  |  |
| EPI580387 | EPI628519 | EPI1100636 | EPI507145 | EPI1102059 | EPI1102470 | EPI1100714 | EPI592091 |  |  |  |  |  |  |  |
| EPI1090557 | EPI581597 | EPI627861 | EPI453828 | EPI1102673 | EPI985838 | EPI1101930 | EPI592070 |  |  |  |  |  |  |  |
| EPI627193 | EPI577954 | EPI756012 | EPI490969 | EPI1102737 | EPI926858 | EPI627196 | EPI593552 |  |  |  |  |  |  |  |
| EPI656314 | EPI577934 | EPI1055421 | EPI509093 | EPI1102785 | EPI971181 | EPI682984 | EPI593409 |  |  |  |  |  |  |  |
| EPI656466 | EPI581846 | EPI1101500 | EPI447713 | EPI1102817 | EPI1102934 | EPI468969 | EPI592035 |  |  |  |  |  |  |  |
| EPI628624 | EPI576544 | EPI1090714 | EPI490977 | EPI971272 | EPI971485 | EPI447855 | EPI592386 |  |  |  |  |  |  |  |
| EPI654130 | EPI535130 | EPI627062 | EPI516360 | EPI1090444 | EPI926850 | EPI447915 | EPI592565 |  |  |  |  |  |  |  |
| EPI628696 | EPI457700 | EPI656407 | EPI515468 | EPI1090716 | EPI1154774 | EPI453607 | EPI592098 |  |  |  |  |  |  |  |
| EPI1057920 | EPI1090841 | EPI628605 | EPI530823 | EPI1101843 | EPI627010 | EPI460770 | EPI592429 |  |  |  |  |  |  |  |
| EPI1057936 | EPI1090535 | EPI1090722 | EPI445910 | EPI1100619 | EPI627825 | EPI457656 | EPI592414 |  |  |  |  |  |  |  |
| EPI866569 | EPI692618 | EPI1101012 | EPI515476 | EPI1101819 | EPI627434 | EPI1090488 | EPI592176 |  |  |  |  |  |  |  |
| EPI1057928 | EPI592634 | EPI1101900 | EPI515453 | EPI1100627 | EPI593542 | EPI457784 | EPI592436 |  |  |  |  |  |  |  |
| EPI1057960 | EPI521916 | EPI1103876 | EPI452258 | EPI1100675 | EPI1102302 | EPI576626 | EPI592077 |  |  |  |  |  |  |  |
| EPI1057968 | EPI656241 | EPI1160111 | EPI515460 | EPI1100635 | EPI580372 | EPI497876 | EPI592379 |  |  |  |  |  |  |  |
| EPI1057952 | EPI1090455 | EPI1101284 | EPI452266 | EPI1100603 | EPI628753 | EPI457856 | EPI592351 |  |  |  |  |  |  |  |
| EPI1057944 | EPI692613 | EPI1100700 | EPI471846 | EPI1101787 | EPI566093 | EPI467339 | EPI592365 |  |  |  |  |  |  |  |
| EPI1090293 | EPI593407 | EPI1102866 | EPI460749 | EPI1100555 | EPI627905 | EPI457872 | EPI592042 |  |  |  |  |  |  |  |
| EPI1090397 | EPI593550 | EPI627190 | EPI941037 | EPI1101811 | EPI627913 | EPI497817 | EPI592084 |  |  |  |  |  |  |  |
| EPI1090301 | EPI593471 | EPI656311 | EPI457771 | EPI1101251 | EPI566101 | EPI447841 | EPI592400 |  |  |  |  |  |  |  |
| EPI1074602 | EPI593499 | EPI580280 | EPI457635 | EPI979585 | EPI627498 | EPI469533 | EPI592119 |  |  |  |  |  |  |  |
| EPI1090373 | EPI578031 | EPI566089 | EPI895560 | EPI1102625 | EPI566053 | EPI627156 | EPI592393 |  |  |  |  |  |  |  |
| EPI1013169 | EPI1090647 | EPI580272 | EPI663469 | EPI1101275 | EPI1090598 | EPI453610 | EPI592358 |  |  |  |  |  |  |  |
| EPI1013193 | EPI707896 | EPI580344 | EPI447678 | EPI1101267 | EPI627346 | EPI446459 | EPI592056 |  |  |  |  |  |  |  |
| EPI1013177 | EPI628431 | EPI580264 | EPI497892 | EPI1101219 | EPI627338 | EPI497860 | EPI592337 |  |  |  |  |  |  |  |
| EPI1013201 | EPI628191 | EPI580324 | EPI440683 | EPI1101227 | EPI627322 | EPI639590 | EPI592422 |  |  |  |  |  |  |  |
| EPI1013209 | EPI628383 | EPI580288 | EPI457619 | EPI1101243 | EPI566061 | EPI627124 | EPI592344 |  |  |  |  |  |  |  |
| EPI1013185 | EPI531470 | EPI580296 | EPI545801 | EPI1101235 | EPI627314 | EPI628051 | EPI592407 |  |  |  |  |  |  |  |
| EPI972264 | EPI628351 | EPI566669 | EPI663470 | EPI1101211 | EPI566109 | EPI628715 | EPI592112 |  |  |  |  |  |  |  |
| EPI1040443 | EPI627592 | EPI580310 | EPI895617 | EPI1101259 | EPI566045 | EPI628699 | EPI592049 |  |  |  |  |  |  |  |
| EPI917291 | EPI628087 | EPI580334 | EPI457755 | EPI1100795 | EPI1090606 | EPI656261 | EPI592330 |  |  |  |  |  |  |  |
| EPI1074594 | EPI628247 | EPI580352 | EPI443641 | EPI1101027 | EPI1101088 | EPI1101482 | EPI592063 |  |  |  |  |  |  |  |
| EPI1090405 | EPI1090471 | EPI628709 | EPI443633 | EPI887632 | EPI1055393 | EPI1057963 | EPI592028 |  |  |  |  |  |  |  |
| EPI1040441 | EPI628287 | EPI656359 | EPI509096 | EPI1102179 | EPI1090526 | EPI627172 | EPI592372 |  |  |  |  |  |  |  |
| EPI1040435 | EPI541776 | EPI559414 | EPI509109 | EPI1102190 | EPI627130 | EPI971519 | EPI572725 |  |  |  |  |  |  |  |
| EPI1040437 | EPI656194 | EPI566033 | EPI453648 | EPI919595 | EPI621118 | EPI1101938 | EPI469528 |  |  |  |  |  |  |  |
| EPI1040439 | EPI628135 | EPI656295 | EPI457835 | EPI918734 | EPI621123 | EPI1102018 | EPI468970 |  |  |  |  |  |  |  |
| EPI1040453 | EPI628183 | EPI1102570 | EPI447910 | EPI965015 | EPI621130 | EPI971447 | EPI469517 |  |  |  |  |  |  |  |
| EPI1040455 | EPI1090479 | EPI887633 | EPI576618 | EPI627061 | EPI627034 | EPI971455 | EPI469516 |  |  |  |  |  |  |  |
| EPI1040471 | EPI576549 | EPI971305 | EPI497820 | EPI656406 | EPI628593 | EPI971463 | EPI469518 |  |  |  |  |  |  |  |
| EPI979613 | EPI707902 | EPI1102060 | EPI447643 | EPI1057924 | EPI1090246 | EPI1100538 | EPI553464 |  |  |  |  |  |  |  |
| EPI1013217 | EPI707903 | EPI926830 | EPI457867 | EPI1057940 | EPI1090230 | EPI971511 | EPI627267 |  |  |  |  |  |  |  |
| EPI1100831 | EPI1034275 | EPI869670 | EPI447889 | EPI1057948 | EPI628801 | EPI1102584 | EPI656268 |  |  |  |  |  |  |  |
| EPI1090389 | EPI1090239 | EPI1101676 | EPI447664 | EPI627421 | EPI627162 | EPI1102576 | EPI656492 |  |  |  |  |  |  |  |
| EPI926825 | EPI833707 | EPI1101668 | EPI447685 | EPI627429 | EPI628737 | EPI1100642 | EPI1100977 |  |  |  |  |  |  |  |
| EPI972298 | EPI1101454 | EPI628613 | EPI467311 | EPI1090724 | EPI627002 | EPI1100650 | EPI1100985 |  |  |  |  |  |  |  |
| EPI919615 | EPI627288 | EPI1102810 | EPI628206 | EPI1101011 | EPI627090 | EPI1100482 | EPI656324 |  |  |  |  |  |  |  |
| EPI972278 | EPI628655 | EPI1102850 | EPI628398 | EPI926837 | EPI1102056 | EPI1100410 | EPI627203 |  |  |  |  |  |  |  |
| EPI1171788 | EPI627464 | EPI926862 | EPI656185 | EPI930814 | EPI627082 | EPI971327 | EPI656276 |  |  |  |  |  |  |  |
| EPI1013265 | EPI566083 | EPI985842 | EPI656224 | EPI979601 | EPI627074 | EPI1100418 | EPI1090329 |  |  |  |  |  |  |  |
| EPI972303 | EPI1090719 | EPI1154770 | EPI531817 | EPI1100699 | EPI628649 | EPI1090640 | EPI580367 |  |  |  |  |  |  |  |
| EPI1013273 | EPI1090727 | EPI971185 | EPI443570 | EPI971464 | EPI628577 | EPI592233 | EPI1090257 |  |  |  |  |  |  |  |
| EPI979605 | EPI1101014 | EPI971489 | EPI498082 | EPI971456 | EPI628569 | EPI592254 | EPI628642 |  |  |  |  |  |  |  |
| EPI979597 | EPI627080 | EPI926854 | EPI447741 | EPI971448 | EPI628585 | EPI528365 | EPI583218 |  |  |  |  |  |  |  |
| EPI1013241 | EPI627088 | EPI1102938 | EPI469195 | EPI1100411 | EPI656259 | EPI628027 | EPI497853 |  |  |  |  |  |  |  |
| EPI917102 | EPI1101022 | EPI1101604 | EPI469193 | EPI971328 | EPI627042 | EPI627460 | EPI457791 |  |  |  |  |  |  |  |
| EPI1013257 | EPI1090827 | EPI1090362 | EPI469194 | EPI1100419 | EPI656451 | EPI628595 | EPI451335 |  |  |  |  |  |  |  |
| EPI919599 | EPI1102062 | EPI1100812 | EPI446964 | EPI1100643 | EPI627250 | EPI627036 | EPI628530 |  |  |  |  |  |  |  |
| EPI918736 | EPI887635 | EPI1101564 | EPI448934 | EPI1100651 | EPI654126 | EPI1090232 | EPI443046 |  |  |  |  |  |  |  |
| EPI965016 | EPI971307 | EPI1057941 | EPI439501 | EPI1100483 | EPI627210 | EPI1090184 | EPI447746 |  |  |  |  |  |  |  |
| EPI972302 | EPI1103966 | EPI1057949 | EPI568704 | EPI1100539 | EPI656443 | EPI533254 | EPI460755 |  |  |  |  |  |  |  |
| EPI1022633 | EPI627456 | EPI1101020 | EPI1102053 | EPI1101939 | EPI626994 | EPI533255 | EPI498102 |  |  |  |  |  |  |  |
| EPI1022617 | EPI941049 | EPI1100660 | EPI1101605 | EPI971512 | EPI773744 | EPI1057915 | EPI453652 |  |  |  |  |  |  |  |
| EPI1013249 | EPI628807 | EPI1101996 | EPI620147 | EPI1102019 | EPI627066 | EPI627492 | EPI497914 |  |  |  |  |  |  |  |
| EPI1018168 | EPI769577 | EPI1100412 | EPI895602 | EPI1100459 | EPI656387 | EPI1090248 | EPI464589 |  |  |  |  |  |  |  |
| EPI1022665 | EPI627104 | EPI971329 | EPI895559 | EPI1100499 | EPI627098 | EPI627276 | EPI1090361 |  |  |  |  |  |  |  |
| EPI1022649 | EPI627296 | EPI1100420 | EPI613795 | EPI1100507 | EPI627218 | EPI627484 | EPI580383 |  |  |  |  |  |  |  |
| EPI1171786 | EPI627256 | EPI971449 | EPI613788 | EPI1101563 | EPI773743 | EPI627468 | EPI580391 |  |  |  |  |  |  |  |
| EPI1013225 | EPI627312 | EPI971457 | EPI613826 | EPI1090824 | EPI585263 | EPI627476 | EPI627067 |  |  |  |  |  |  |  |
| EPI997217 | EPI627304 | EPI971465 | EPI531123 | EPI1100451 | EPI656427 | EPI566039 | EPI656364 |  |  |  |  |  |  |  |
| EPI960361 | EPI628751 | EPI1100644 | EPI620165 | EPI1090572 | EPI580388 | EPI628803 | EPI656388 |  |  |  |  |  |  |  |
| EPI1010184 | EPI627168 | EPI1100652 | EPI620126 | EPI1100443 | EPI656467 | EPI1090552 | EPI447832 |  |  |  |  |  |  |  |
| EPI919607 | EPI627224 | EPI1100540 | EPI613833 | EPI1100523 | EPI1057961 | EPI628795 | EPI613825 |  |  |  |  |  |  |  |
| EPI926817 | EPI576036 | EPI1101940 | EPI620151 | EPI1100707 | EPI627290 | EPI628827 | EPI497833 |  |  |  |  |  |  |  |
| EPI960360 | EPI627096 | EPI971521 | EPI620023 | EPI1100923 | EPI769576 | EPI1090568 | EPI627427 |  |  |  |  |  |  |  |
| EPI1022657 | EPI692626 | EPI1102020 | EPI620197 | EPI1101915 | EPI769568 | EPI1103882 | EPI1090665 |  |  |  |  |  |  |  |
| EPI1040463 | EPI692616 | EPI1100484 | EPI620158 | EPI1054138 | EPI628657 | EPI592125 | EPI1090633 |  |  |  |  |  |  |  |
| EPI1102015 | EPI627008 | EPI1102578 | EPI620116 | EPI1100915 | EPI1090542 | EPI1090648 | EPI627419 |  |  |  |  |  |  |  |
| EPI1054134 | EPI683029 | EPI971513 | EPI620123 | EPI1100947 | EPI628721 | EPI682987 | EPI627395 |  |  |  |  |  |  |  |
| EPI1010099 | EPI769569 | EPI1102586 | EPI620102 | EPI1100931 | EPI628729 | EPI1090504 | EPI627922 |  |  |  |  |  |  |  |
| EPI1022625 | EPI627240 | EPI593346 | EPI620140 | EPI1100939 | EPI627450 | EPI1090624 | EPI628170 |  |  |  |  |  |  |  |
| EPI1040479 | EPI627472 | EPI593353 | EPI620037 | EPI627860 | EPI941048 | EPI1090672 | EPI628162 |  |  |  |  |  |  |  |
| EPI1018152 | EPI566130 | EPI1103980 | EPI613774 | EPI756014 | EPI656459 | EPI443646 | EPI628210 |  |  |  |  |  |  |  |
| EPI1018144 | EPI628663 | EPI1036682 | EPI620083 | EPI566120 | EPI627186 | EPI508929 | EPI764568 |  |  |  |  |  |  |  |
| EPI1018112 | EPI628743 | EPI1102169 | EPI613819 | EPI627125 | EPI656307 | EPI515799 | EPI477414 |  |  |  |  |  |  |  |
| EPI1018250 | EPI628775 | EPI1102192 | EPI620044 | EPI627109 | EPI1057929 | EPI515450 | EPI477453 |  |  |  |  |  |  |  |
| EPI1018096 | EPI628823 | EPI1101612 | EPI620051 | EPI656342 | EPI628689 | EPI516364 | EPI764622 |  |  |  |  |  |  |  |
| EPI1022673 | EPI628567 | EPI979514 | EPI620058 | EPI627261 | EPI627234 | EPI515465 | EPI583170 |  |  |  |  |  |  |  |
| EPI1054118 | EPI628607 | EPI919596 | EPI620190 | EPI656318 | EPI580380 | EPI447757 | EPI764627 |  |  |  |  |  |  |  |
| EPI1013233 | EPI1090559 | EPI918730 | EPI620076 | EPI1103963 | EPI627378 | EPI457640 | EPI764628 |  |  |  |  |  |  |  |
| EPI1018120 | EPI628727 | EPI965013 | EPI619993 | EPI971304 | EPI1090358 | EPI509072 | EPI1090681 |  |  |  |  |  |  |  |
| EPI1018266 | EPI1090543 | EPI930812 | EPI620090 | EPI591464 | EPI773732 | EPI451257 | EPI895548 |  |  |  |  |  |  |  |
| EPI1022708 | EPI628615 | EPI1022630 | EPI620172 | EPI577209 | EPI627490 | EPI515457 | EPI1090673 |  |  |  |  |  |  |  |
| EPI1018128 | EPI628591 | EPI979602 | EPI613767 | EPI577290 | EPI628625 | EPI447771 | EPI895540 |  |  |  |  |  |  |  |
| EPI1171790 | EPI628575 | EPI1090402 | EPI620109 | EPI577289 | EPI628617 | EPI452263 | EPI895542 |  |  |  |  |  |  |  |
| EPI1100863 | EPI628583 | EPI1101620 | EPI620097 | EPI577198 | EPI628633 | EPI451334 | EPI510001 |  |  |  |  |  |  |  |
| EPI1100871 | EPI627040 | EPI1101572 | EPI620030 | EPI577295 | EPI773719 | EPI445915 | EPI443671 |  |  |  |  |  |  |  |
| EPI1100847 | EPI656489 | EPI1100788 | EPI613812 | EPI591246 | EPI656371 | EPI507150 | EPI628602 |  |  |  |  |  |  |  |
| EPI1018088 | EPI656369 | EPI1100796 | EPI613781 | EPI577212 | EPI628681 | EPI504727 | EPI566030 |  |  |  |  |  |  |  |
| EPI1018202 | EPI627232 | EPI1101596 | EPI620069 | EPI577287 | EPI656419 | EPI515473 | EPI656292 |  |  |  |  |  |  |  |
| EPI1100855 | EPI576013 | EPI1018215 | EPI620133 | EPI577285 | EPI627226 | EPI504717 | EPI580331 |  |  |  |  |  |  |  |
| EPI1022609 | EPI656465 | EPI1101636 | EPI439508 | EPI591301 | EPI627114 | EPI457624 | EPI656356 |  |  |  |  |  |  |  |
| EPI1018104 | EPI656449 | EPI1101660 | EPI620183 | EPI577213 | EPI656347 | EPI576605 | EPI1101289 |  |  |  |  |  |  |  |
| EPI1018239 | EPI1057927 | EPI872955 | EPI515786 | EPI577294 | EPI773720 | EPI510156 | EPI1101273 |  |  |  |  |  |  |  |
| EPI973363 | EPI1057959 | EPI1101644 | EPI663465 | EPI577281 | EPI656483 | EPI582940 | EPI1101249 |  |  |  |  |  |  |  |
| EPI1022697 | EPI1057967 | EPI1101628 | EPI447657 | EPI577291 | EPI773726 | EPI583047 | EPI1102623 |  |  |  |  |  |  |  |
| EPI1022641 | EPI656353 | EPI1040468 | EPI663464 | EPI577280 | EPI773750 | EPI582947 | EPI979583 |  |  |  |  |  |  |  |
| EPI1022689 | EPI656481 | EPI1100828 | EPI497900 | EPI577293 | EPI627298 | EPI582962 | EPI1101265 |  |  |  |  |  |  |  |
| EPI1018136 | EPI628623 | EPI1101580 | EPI447699 | EPI577203 | EPI971333 | EPI628251 | EPI1102631 |  |  |  |  |  |  |  |
| EPI1018176 | EPI627192 | EPI979594 | EPI1090816 | EPI577211 | EPI971341 | EPI535182 | EPI1090489 |  |  |  |  |  |  |  |
| EPI1022681 | EPI656313 | EPI1013270 | EPI590924 | EPI577298 | EPI1101288 | EPI627572 | EPI447887 |  |  |  |  |  |  |  |
| EPI1102597 | EPI576053 | EPI979610 | EPI582982 | EPI577208 | EPI1102430 | EPI627580 | EPI1100753 |  |  |  |  |  |  |  |
| EPI1040889 | EPI627264 | EPI887841 | EPI628254 | EPI591302 | EPI887605 | EPI535166 | EPI1102679 |  |  |  |  |  |  |  |
| EPI1018160 | EPI656321 | EPI1022646 | EPI583013 | EPI577283 | EPI971085 | EPI627596 | EPI1102887 |  |  |  |  |  |  |  |
| EPI628832 | EPI568234 | EPI884219 | EPI627055 | EPI577296 | EPI971101 | EPI535198 | EPI1102895 |  |  |  |  |  |  |  |
| EPI627177 | EPI656345 | EPI887977 | EPI656440 | EPI577286 | EPI1102152 | EPI627803 | EPI1102735 |  |  |  |  |  |  |  |
| EPI1090565 | EPI627112 | EPI926838 | EPI656400 | EPI577204 | EPI1102175 | EPI592628 | EPI1102879 |  |  |  |  |  |  |  |
| EPI628800 | EPI576005 | EPI926846 | EPI1022663 | EPI577199 | EPI1103936 | EPI627875 | EPI1102783 |  |  |  |  |  |  |  |
| EPI628544 | EPI627384 | EPI874335 | EPI887842 | EPI577297 | EPI1102862 | EPI627819 | EPI1102815 |  |  |  |  |  |  |  |
| EPI628560 | EPI1090359 | EPI977392 | EPI1040449 | EPI577201 | EPI1101000 | EPI627891 | EPI919593 |  |  |  |  |  |  |  |
| EPI1090589 | EPI627000 | EPI1040484 | EPI973369 | EPI577288 | EPI971397 | EPI627883 | EPI918740 |  |  |  |  |  |  |  |
| EPI1100447 | EPI656265 | EPI1101652 | EPI1018094 | EPI577217 | EPI1101032 | EPI627811 | EPI965020 |  |  |  |  |  |  |  |
| EPI1100455 | EPI627024 | EPI887833 | EPI1018142 | EPI577215 | EPI971421 | EPI627899 | EPI887630 |  |  |  |  |  |  |  |
| EPI1103895 | EPI580381 | EPI1101556 | EPI1018150 | EPI577292 | EPI971429 | EPI592168 | EPI1102057 |  |  |  |  |  |  |  |
| EPI1055416 | EPI627216 | EPI930804 | EPI1100845 | EPI577282 | EPI1102742 | EPI593156 | EPI1100441 |  |  |  |  |  |  |  |
| EPI1055400 | EPI656337 | EPI997214 | EPI1018174 | EPI577200 | EPI1102750 | EPI592655 | EPI1100449 |  |  |  |  |  |  |  |
| EPI1090525 | EPI580389 | EPI926814 | EPI1018134 | EPI577210 | EPI1102726 | EPI592602 | EPI1100521 |  |  |  |  |  |  |  |
| EPI1101095 | EPI627120 | EPI960354 | EPI1018264 | EPI577214 | EPI1102358 | EPI592621 | EPI1090577 |  |  |  |  |  |  |  |
| EPI1055408 | EPI1057935 | EPI960355 | EPI1018086 | EPI577279 | EPI1102438 | EPI592219 | EPI1100705 |  |  |  |  |  |  |  |
| EPI887740 | EPI575997 | EPI919604 | EPI1018197 | EPI577202 | EPI971365 | EPI592588 | EPI1101913 |  |  |  |  |  |  |  |
| EPI1090749 | EPI628695 | EPI1013222 | EPI1018158 | EPI577216 | EPI971357 | EPI592261 | EPI1100913 |  |  |  |  |  |  |  |
| EPI1090613 | EPI654129 | EPI1040448 | EPI1022706 | EPI577206 | EPI971349 | EPI627867 | EPI1054136 |  |  |  |  |  |  |  |
| EPI971276 | EPI656425 | EPI1090482 | EPI1054116 | EPI577284 | EPI971437 | EPI592240 | EPI1100929 |  |  |  |  |  |  |  |
| EPI1103407 | EPI656433 | EPI1101220 | EPI1102013 | EPI577193 | EPI866562 | EPI592154 | EPI1100937 |  |  |  |  |  |  |  |
| EPI682922 | EPI576063 | EPI627374 | EPI1040895 | EPI577207 | EPI887661 | EPI592550 | EPI1100921 |  |  |  |  |  |  |  |
| EPI1090621 | EPI576020 | EPI1102482 | EPI1022639 | EPI628020 | EPI1055401 | EPI628371 | EPI1100945 |  |  |  |  |  |  |  |
| EPI1100519 | EPI576029 | EPI942258 | EPI1022655 | EPI628404 | EPI1102064 | EPI627548 | EPI580342 |  |  |  |  |  |  |  |
| EPI1090549 | EPI576072 | EPI1057917 | EPI1022671 | EPI591128 | EPI1102072 | EPI535118 | EPI627243 |  |  |  |  |  |  |  |
| EPI627161 | EPI628687 | EPI1090234 | EPI1102595 | EPI1102865 | EPI1103094 | EPI528333 | EPI683115 |  |  |  |  |  |  |  |
| EPI1090501 | EPI656377 | EPI833701 | EPI1022679 | EPI1102881 | EPI1102390 | EPI528341 | EPI683117 |  |  |  |  |  |  |  |
| EPI552399 | EPI627048 | EPI1101452 | EPI1022687 | EPI1102889 | EPI887701 | EPI627540 | EPI628746 |  |  |  |  |  |  |  |
| EPI627185 | EPI656457 | EPI1090554 | EPI1022695 | EPI627029 | EPI1102462 | EPI528357 | EPI627195 |  |  |  |  |  |  |  |
| EPI682924 | EPI656473 | EPI1102970 | EPI1013231 | EPI627141 | EPI887685 | EPI627835 | EPI656300 |  |  |  |  |  |  |  |
| EPI628736 | EPI866568 | EPI1090823 | EPI1018110 | EPI627149 | EPI1103062 | EPI627412 | EPI627371 |  |  |  |  |  |  |  |
| EPI628768 | EPI628631 | EPI1100516 | EPI1018248 | EPI566135 | EPI1103038 | EPI627404 | EPI627027 |  |  |  |  |  |  |  |
| EPI628816 | EPI628639 | EPI1057957 | EPI1040477 | EPI627509 | EPI1101712 | EPI627444 | EPI1101593 |  |  |  |  |  |  |  |
| EPI628664 | EPI1090367 | EPI866566 | EPI1018243 | EPI895551 | EPI1054143 | EPI628291 | EPI1100513 |  |  |  |  |  |  |  |
| EPI1101199 | EPI1057943 | EPI628749 | EPI1018102 | EPI895549 | EPI1100968 | EPI533262 | EPI866563 |  |  |  |  |  |  |  |
| EPI1101207 | EPI1057951 | EPI1101924 | EPI1022607 | EPI895553 | EPI945064 | EPI566119 | EPI1100721 |  |  |  |  |  |  |  |
| EPI1101431 | EPI1040434 | EPI1100716 | EPI1067573 | EPI895552 | EPI971093 | EPI535190 | EPI1100737 |  |  |  |  |  |  |  |
| EPI628552 | EPI1040436 | EPI1101932 | EPI979611 | EPI895556 | EPI1101456 | EPI627532 | EPI1101561 |  |  |  |  |  |  |  |
| EPI628568 | EPI1090295 | EPI1103360 | EPI1022623 | EPI895557 | EPI1103078 | EPI583017 | EPI887702 |  |  |  |  |  |  |  |
| EPI628608 | EPI1090399 | EPI1055405 | EPI1018118 | EPI683240 | EPI1103086 | EPI528293 | EPI887846 |  |  |  |  |  |  |  |
| EPI628680 | EPI1074596 | EPI887753 | EPI1100861 | EPI683241 | EPI971109 | EPI582986 | EPI888078 |  |  |  |  |  |  |  |
| EPI628784 | EPI1090407 | EPI1090506 | EPI1100869 | EPI683242 | EPI1102982 | EPI627612 | EPI971262 |  |  |  |  |  |  |  |
| EPI1101439 | EPI1040440 | EPI1090514 | EPI1040461 | EPI942198 | EPI1054103 | EPI583024 | EPI887606 |  |  |  |  |  |  |  |
| EPI628672 | EPI1040442 | EPI971337 | EPI1100853 | EPI1090644 | EPI1101768 | EPI628139 | EPI971086 |  |  |  |  |  |  |  |
| EPI628792 | EPI1040438 | EPI971345 | EPI1010097 | EPI1055428 | EPI887621 | EPI535150 | EPI971102 |  |  |  |  |  |  |  |
| EPI627513 | EPI1040452 | EPI1103098 | EPI1018126 | EPI1090684 | EPI887653 | EPI628235 | EPI1103055 |  |  |  |  |  |  |  |
| EPI1103887 | EPI1013208 | EPI1102394 | EPI457440 | EPI1101923 | EPI1103102 | EPI627388 | EPI1054104 |  |  |  |  |  |  |  |
| EPI566138 | EPI1013192 | EPI1102466 | EPI509064 | EPI1100715 | EPI1054095 | EPI582350 | EPI887686 |  |  |  |  |  |  |  |
| EPI707987 | EPI1013168 | EPI887713 | EPI627607 | EPI1101931 | EPI979614 | EPI535174 | EPI1054096 |  |  |  |  |  |  |  |
| EPI707994 | EPI1090375 | EPI887721 | EPI576588 | EPI979577 | EPI979558 | EPI582969 | EPI1103103 |  |  |  |  |  |  |  |
| EPI708010 | EPI1013184 | EPI1102674 | EPI576589 | EPI1101299 | EPI1103046 | EPI535158 | EPI1103063 |  |  |  |  |  |  |  |
| EPI707997 | EPI1013200 | EPI1102706 | EPI532272 | EPI1101307 | EPI1102486 | EPI628043 | EPI979615 |  |  |  |  |  |  |  |
| EPI627017 | EPI1013176 | EPI971089 | EPI442719 | EPI1103939 | EPI971493 | EPI628339 | EPI1102639 |  |  |  |  |  |  |  |
| EPI707989 | EPI1090303 | EPI971105 | EPI576590 | EPI1101003 | EPI971077 | EPI628315 | EPI971094 |  |  |  |  |  |  |  |
| EPI707991 | EPI1074604 | EPI1102378 | EPI457739 | EPI1102156 | EPI1102414 | EPI628299 | EPI1101713 |  |  |  |  |  |  |  |
| EPI707986 | EPI926824 | EPI887945 | EPI1090795 | EPI971400 | EPI1101720 | EPI628059 | EPI971110 |  |  |  |  |  |  |  |
| EPI707992 | EPI919614 | EPI1103245 | EPI576621 | EPI971520 | EPI1102105 | EPI582357 | EPI1102447 |  |  |  |  |  |  |  |
| EPI707998 | EPI972277 | EPI1103074 | EPI441800 | EPI895558 | EPI971277 | EPI535134 | EPI1102199 |  |  |  |  |  |  |  |
| EPI707996 | EPI972297 | EPI1102354 | EPI447748 | EPI895550 | EPI1103054 | EPI628331 | EPI1103087 |  |  |  |  |  |  |  |
| EPI708008 | EPI972279 | EPI1102370 | EPI453478 | EPI895555 | EPI1013226 | EPI628307 | EPI1102415 |  |  |  |  |  |  |  |
| EPI707988 | EPI1013240 | EPI979618 | EPI443040 | EPI895554 | EPI1102422 | EPI583010 | EPI1102383 |  |  |  |  |  |  |  |
| EPI707990 | EPI1013256 | EPI887665 | EPI683159 | EPI887800 | EPI887645 | EPI528373 | EPI1102487 |  |  |  |  |  |  |  |
| EPI707999 | EPI965018 | EPI1103066 | EPI683157 | EPI1101099 | EPI1102638 | EPI628259 | EPI979645 |  |  |  |  |  |  |  |
| EPI708012 | EPI918738 | EPI1103106 | EPI683155 | EPI1101107 | EPI1100800 | EPI628323 | EPI887646 |  |  |  |  |  |  |  |
| EPI708011 | EPI919598 | EPI1103082 | EPI509113 | EPI712922 | EPI1102446 | EPI582908 | EPI979559 |  |  |  |  |  |  |  |
| EPI708084 | EPI1013264 | EPI1054107 | EPI576541 | EPI887736 | EPI1102990 | EPI627524 | EPI979551 |  |  |  |  |  |  |  |
| EPI707993 | EPI997159 | EPI1054147 | EPI583136 | EPI1102985 | EPI1102382 | EPI627556 | EPI1103079 |  |  |  |  |  |  |  |
| EPI1101927 | EPI1090391 | EPI887657 | EPI583143 | EPI1103009 | EPI447710 | EPI628035 | EPI887622 |  |  |  |  |  |  |  |
| EPI1100719 | EPI1022616 | EPI1054099 | EPI583094 | EPI887608 | EPI453830 | EPI535142 | EPI1100969 |  |  |  |  |  |  |  |
| EPI1101935 | EPI1022632 | EPI1102642 | EPI583150 | EPI971088 | EPI509090 | EPI542096 | EPI1100801 |  |  |  |  |  |  |  |
| EPI1102397 | EPI1013216 | EPI945068 | EPI1090579 | EPI971104 | EPI683071 | EPI627564 | EPI887654 |  |  |  |  |  |  |  |
| EPI1102469 | EPI1100830 | EPI1103042 | EPI627015 | EPI1090372 | EPI1013234 | EPI457672 | EPI1101721 |  |  |  |  |  |  |  |
| EPI887668 | EPI1022648 | EPI1102386 | EPI627511 | EPI1090364 | EPI1101928 | EPI590929 | EPI1103047 |  |  |  |  |  |  |  |
| EPI1102437 | EPI1010185 | EPI1102994 | EPI541772 | EPI1055404 | EPI1101920 | EPI583126 | EPI1101457 |  |  |  |  |  |  |  |
| EPI1102477 | EPI1010187 | EPI1102202 | EPI1103885 | EPI1103891 | EPI1100712 | EPI1103978 | EPI1103039 |  |  |  |  |  |  |  |
| EPI926865 | EPI997219 | EPI887625 | EPI531795 | EPI1101499 | EPI1090622 | EPI1036688 | EPI1102106 |  |  |  |  |  |  |  |
| EPI971100 | EPI919606 | EPI1101724 | EPI593380 | EPI887672 | EPI1090502 | EPI1102166 | EPI971078 |  |  |  |  |  |  |  |
| EPI1103061 | EPI1013224 | EPI1102110 | EPI1090451 | EPI887808 | EPI628809 | EPI1102155 | EPI1102991 |  |  |  |  |  |  |  |
| EPI971084 | EPI960364 | EPI1103058 | EPI1090837 | EPI1102585 | EPI683074 | EPI1103938 | EPI945065 |  |  |  |  |  |  |  |
| EPI971500 | EPI926816 | EPI971281 | EPI593496 | EPI887928 | EPI1101360 | EPI1102178 | EPI971278 |  |  |  |  |  |  |  |
| EPI1103013 | EPI960365 | EPI971113 | EPI593468 | EPI912955 | EPI1102542 | EPI1101002 | EPI1054144 |  |  |  |  |  |  |  |
| EPI887628 | EPI1022707 | EPI1101716 | EPI592631 | EPI887896 | EPI1101376 | EPI1100426 | EPI971494 |  |  |  |  |  |  |  |
| EPI1102421 | EPI1022624 | EPI971081 | EPI593540 | EPI912947 | EPI1101392 | EPI971423 | EPI1090561 |  |  |  |  |  |  |  |
| EPI972252 | EPI1102596 | EPI971497 | EPI582339 | EPI1101123 | EPI1101400 | EPI1102680 | EPI1057914 |  |  |  |  |  |  |  |
| EPI1102389 | EPI1022672 | EPI1100804 | EPI583107 | EPI1102257 | EPI1101384 | EPI1101586 | EPI1101769 |  |  |  |  |  |  |  |
| EPI887692 | EPI1018135 | EPI887649 | EPI1090731 | EPI1102297 | EPI1101368 | EPI971431 | EPI628538 |  |  |  |  |  |  |  |
| EPI887660 | EPI1018265 | EPI1100972 | EPI628574 | EPI1102321 | EPI1101408 | EPI1102800 | EPI1101601 |  |  |  |  |  |  |  |
| EPI1102493 | EPI1022640 | EPI1103090 | EPI628582 | EPI922140 | EPI628825 | EPI1100458 | EPI971414 |  |  |  |  |  |  |  |
| EPI971188 | EPI1022680 | EPI1102418 | EPI628590 | EPI1103843 | EPI627170 | EPI1101986 | EPI1036701 |  |  |  |  |  |  |  |
| EPI1103109 | EPI1022688 | EPI1102450 | EPI628742 | EPI1103971 | EPI1090566 | EPI1102440 | EPI1103969 |  |  |  |  |  |  |  |
| EPI926857 | EPI1022696 | EPI971097 | EPI628654 | EPI1103987 | EPI628793 | EPI1102752 | EPI1036697 |  |  |  |  |  |  |  |
| EPI1054102 | EPI1018103 | EPI1101460 | EPI627007 | EPI1102273 | EPI1074595 | EPI1100762 | EPI1103985 |  |  |  |  |  |  |  |
| EPI979641 | EPI1018087 | EPI979562 | EPI627095 | EPI887960 | EPI1090406 | EPI1102728 | EPI971302 |  |  |  |  |  |  |  |
| EPI971492 | EPI1018237 | EPI979554 | EPI627167 | EPI887848 | EPI1090294 | EPI971375 | EPI1100633 |  |  |  |  |  |  |  |
| EPI1054150 | EPI1040478 | EPI979546 | EPI627079 | EPI888080 | EPI1090398 | EPI1102744 | EPI1100553 |  |  |  |  |  |  |  |
| EPI1102941 | EPI1018201 | EPI1102490 | EPI627087 | EPI1100979 | EPI1090302 | EPI1090368 | EPI1100601 |  |  |  |  |  |  |  |
| EPI979621 | EPI1040891 | EPI1103050 | EPI535129 | EPI1100987 | EPI1074603 | EPI866564 | EPI1101809 |  |  |  |  |  |  |  |
| EPI985835 | EPI1018127 | EPI1090490 | EPI627591 | EPI1102209 | EPI1013170 | EPI926820 | EPI1100625 |  |  |  |  |  |  |  |
| EPI979557 | EPI1067570 | EPI1102402 | EPI566074 | EPI1102091 | EPI1013178 | EPI1090408 | EPI1100673 |  |  |  |  |  |  |  |
| EPI1103053 | EPI1010101 | EPI1100924 | EPI627135 | EPI1100531 | EPI1013194 | EPI1090304 | EPI1101785 |  |  |  |  |  |  |  |
| EPI887652 | EPI1013232 | EPI1100932 | EPI621125 | EPI1101139 | EPI1013202 | EPI1074605 | EPI1101817 |  |  |  |  |  |  |  |
| EPI1101463 | EPI1018159 | EPI1101916 | EPI621132 | EPI1102099 | EPI1013186 | EPI1013172 | EPI1100617 |  |  |  |  |  |  |  |
| EPI979549 | EPI1171789 | EPI1100708 | EPI628750 | EPI1103211 | EPI1013162 | EPI1013180 | EPI1101841 |  |  |  |  |  |  |  |
| EPI979565 | EPI1100854 | EPI1100940 | EPI683156 | EPI1100995 | EPI1040472 | EPI1013196 | EPI1101185 |  |  |  |  |  |  |  |
| EPI1103045 | EPI1018249 | EPI1100948 | EPI730493 | EPI1102144 | EPI919608 | EPI1013204 | EPI712920 |  |  |  |  |  |  |  |
| EPI1102116 | EPI1018111 | EPI1054139 | EPI1090683 | EPI628556 | EPI1013210 | EPI1013188 | EPI887854 |  |  |  |  |  |  |  |
| EPI1102405 | EPI1018175 | EPI1100916 | EPI1102395 | EPI1090524 | EPI1013258 | EPI1013164 | EPI887822 |  |  |  |  |  |  |  |
| EPI1103093 | EPI1100862 | EPI887905 | EPI1102467 | EPI1055396 | EPI1090390 | EPI979592 | EPI887870 |  |  |  |  |  |  |  |
| EPI1103101 | EPI1100870 | EPI887961 | EPI1103099 | EPI1101091 | EPI926818 | EPI1013268 | EPI628554 |  |  |  |  |  |  |  |
| EPI1102071 | EPI1018151 | EPI887785 | EPI887690 | EPI1103319 | EPI1013250 | EPI1090296 | EPI971198 |  |  |  |  |  |  |  |
| EPI1102079 | EPI1018143 | EPI1054123 | EPI1102069 | EPI1090748 | EPI1018267 | EPI1090400 | EPI971214 |  |  |  |  |  |  |  |
| EPI887764 | EPI973365 | EPI887897 | EPI1102077 | EPI887936 | EPI979606 | EPI1022628 | EPI971166 |  |  |  |  |  |  |  |
| EPI1103021 | EPI1018095 | EPI912945 | EPI887666 | EPI1103867 | EPI1100840 | EPI919610 | EPI971190 |  |  |  |  |  |  |  |
| EPI1102989 | EPI1022656 | EPI887929 | EPI979619 | EPI921764 | EPI1010100 | EPI1040466 | EPI971206 |  |  |  |  |  |  |  |
| EPI971284 | EPI1018119 | EPI912953 | EPI979547 | EPI1103827 | EPI1040456 | EPI1074597 | EPI887710 |  |  |  |  |  |  |  |
| EPI1103069 | EPI1054117 | EPI1101588 | EPI1103067 | EPI1090588 | EPI1040892 | EPI1022612 | EPI971174 |  |  |  |  |  |  |  |
| EPI1102453 | EPI1022608 | EPI1101972 | EPI1102987 | EPI1103400 | EPI1022650 | EPI1013212 | EPI887718 |  |  |  |  |  |  |  |
| EPI971116 | EPI1100846 | EPI1102746 | EPI1103011 | EPI979633 | EPI1022674 | EPI1100826 | EPI1100905 |  |  |  |  |  |  |  |
| EPI1054110 | EPI1040242 | EPI1102802 | EPI1103043 | EPI887784 | EPI1018145 | EPI918739 | EPI1102903 |  |  |  |  |  |  |  |
| EPI1154773 | EPI1040240 | EPI1102754 | EPI887626 | EPI887904 | EPI997218 | EPI1090392 | EPI1102911 |  |  |  |  |  |  |  |
| EPI1102205 | EPI1040462 | EPI1100764 | EPI1102419 | EPI1103811 | EPI1013218 | EPI1013260 | EPI1102655 |  |  |  |  |  |  |  |
| EPI1103085 | EPI707891 | EPI1102730 | EPI1102995 | EPI1090772 | EPI960362 | EPI965019 | EPI1102647 |  |  |  |  |  |  |  |
| EPI1102645 | EPI707893 | EPI1102986 | EPI1102203 | EPI1090612 | EPI1022682 | EPI919594 | EPI1102663 |  |  |  |  |  |  |  |
| EPI1101775 | EPI707892 | EPI1103010 | EPI1100973 | EPI1101371 | EPI1018153 | EPI1013236 | EPI1101041 |  |  |  |  |  |  |  |
| EPI1100807 | EPI707898 | EPI1102274 | EPI887658 | EPI1101395 | EPI1022634 | EPI1013252 | EPI1101057 |  |  |  |  |  |  |  |
| EPI1101719 | EPI707985 | EPI1103972 | EPI1103107 | EPI1101379 | EPI1054127 | EPI979608 | EPI1101049 |  |  |  |  |  |  |  |
| EPI1102997 | EPI707899 | EPI1103988 | EPI1103083 | EPI1101387 | EPI1022690 | EPI1022644 | EPI1101073 |  |  |  |  |  |  |  |
| EPI1101727 | EPI566075 | EPI888081 | EPI1102451 | EPI1101403 | EPI1067571 | EPI917068 | EPI1101065 |  |  |  |  |  |  |  |
| EPI1100975 | EPI627136 | EPI887849 | EPI1103059 | EPI1101363 | EPI1022701 | EPI926812 | EPI1101033 |  |  |  |  |  |  |  |
| EPI1090365 | EPI621121 | EPI979634 | EPI1100829 | EPI1101411 | EPI960363 | EPI1013220 | EPI1101081 |  |  |  |  |  |  |  |
| EPI866561 | EPI621116 | EPI1102690 | EPI945069 | EPI1101131 | EPI1018129 | EPI960366 | EPI971158 |  |  |  |  |  |  |  |
| EPI1090517 | EPI621128 | EPI1102698 | EPI971106 | EPI1102313 | EPI919600 | EPI960367 | EPI1100889 |  |  |  |  |  |  |  |
| EPI979517 | EPI628815 | EPI1102722 | EPI971082 | EPI1102553 | EPI1018169 | EPI919602 | EPI1101521 |  |  |  |  |  |  |  |
| EPI926833 | EPI1090503 | EPI971121 | EPI971498 | EPI1102929 | EPI1100848 | EPI997220 | EPI1101321 |  |  |  |  |  |  |  |
| EPI1101671 | EPI1090623 | EPI971129 | EPI1100805 | EPI1090740 | EPI973364 | EPI1090536 | EPI1101337 |  |  |  |  |  |  |  |
| EPI1101679 | EPI627176 | EPI1103320 | EPI1101773 | EPI1101419 | EPI1018089 | EPI628811 | EPI1101353 |  |  |  |  |  |  |  |
| EPI926849 | EPI628831 | EPI1102290 | EPI1100813 | EPI1103389 | EPI1018137 | EPI682985 | EPI1101329 |  |  |  |  |  |  |  |
| EPI1101031 | EPI628799 | EPI1090370 | EPI1101725 | EPI1103899 | EPI1100856 | EPI628563 | EPI1101313 |  |  |  |  |  |  |  |
| EPI1100527 | EPI1090567 | EPI1104116 | EPI887650 | EPI1090460 | EPI1100864 | EPI1101426 | EPI1101345 |  |  |  |  |  |  |  |
| EPI1102549 | EPI1090551 | EPI1104132 | EPI1102387 | EPI1102537 | EPI1022618 | EPI1101202 | EPI1102551 |  |  |  |  |  |  |  |
| EPI1103385 | EPI1100518 | EPI1104156 | EPI1101717 | EPI1022613 | EPI926810 | EPI628779 | EPI1055402 |  |  |  |  |  |  |  |
| EPI1090685 | EPI627160 | EPI971145 | EPI1102643 | EPI1018164 | EPI1018081 | EPI1101434 | EPI1090529 |  |  |  |  |  |  |  |
| EPI1100439 | EPI627016 | EPI971153 | EPI1054108 | EPI1013245 | EPI1018203 | EPI628787 | EPI1055394 |  |  |  |  |  |  |  |
| EPI1102055 | EPI627512 | EPI1102474 | EPI1054148 | EPI1102132 | EPI1054111 | EPI628675 | EPI1101089 |  |  |  |  |  |  |  |
| EPI1102039 | EPI707982 | EPI1102338 | EPI1054100 | EPI1103351 | EPI1102590 | EPI628603 | EPI1102927 |  |  |  |  |  |  |  |
| EPI1102047 | EPI707904 | EPI1102626 | EPI971098 | EPI1102051 | EPI1022666 | EPI628667 | EPI1102535 |  |  |  |  |  |  |  |
| EPI866553 | EPI707983 | EPI1101252 | EPI1101461 | EPI1102035 | EPI1018121 | EPI628547 | EPI1090465 |  |  |  |  |  |  |  |
| EPI979573 | EPI707981 | EPI1101276 | EPI971114 | EPI1102043 | EPI1018105 | EPI1101194 | EPI971334 |  |  |  |  |  |  |  |
| EPI930815 | EPI707978 | EPI1101268 | EPI1103091 | EPI1101603 | EPI1018251 | EPI971279 | EPI971342 |  |  |  |  |  |  |  |
| EPI926841 | EPI707977 | EPI979586 | EPI1102112 | EPI1102969 | EPI1018097 | EPI1102400 | EPI971358 |  |  |  |  |  |  |  |
| EPI1101543 | EPI707890 | EPI628541 | EPI1102491 | EPI1102433 | EPI1018241 | EPI566071 | EPI971438 |  |  |  |  |  |  |  |
| EPI1090509 | EPI707979 | EPI628557 | EPI1102939 | EPI1102473 | EPI1022602 | EPI627132 | EPI971350 |  |  |  |  |  |  |  |
| EPI866545 | EPI707886 | EPI1090522 | EPI979555 | EPI1102393 | EPI1090590 | EPI621117 | EPI971366 |  |  |  |  |  |  |  |
| EPI1100823 | EPI707888 | EPI1101092 | EPI1103051 | EPI1102465 | EPI712919 | EPI621122 | EPI1100641 |  |  |  |  |  |  |  |
| EPI833714 | EPI707984 | EPI1055397 | EPI926855 | EPI1103073 | EPI1103375 | EPI621129 | EPI1100649 |  |  |  |  |  |  |  |
| EPI1101479 | EPI707889 | EPI1102044 | EPI979563 | EPI1102377 | EPI628769 | EPI1101370 | EPI971510 |  |  |  |  |  |  |  |
| EPI1104023 | EPI707887 | EPI1102036 | EPI1054132 | EPI1102481 | EPI628817 | EPI1101378 | EPI1102017 |  |  |  |  |  |  |  |
| EPI1063152 | EPI707980 | EPI1102052 | EPI985841 | EPI1102353 | EPI628537 | EPI1101410 | EPI1100481 |  |  |  |  |  |  |  |
| EPI922141 | EPI566137 | EPI1102298 | EPI971186 | EPI1103244 | EPI628553 | EPI1101386 | EPI1100537 |  |  |  |  |  |  |  |
| EPI1103847 | EPI887739 | EPI1102426 | EPI971490 | EPI1102369 | EPI1103888 | EPI1103378 | EPI971462 |  |  |  |  |  |  |  |
| EPI1063154 | EPI628559 | EPI1101132 | EPI926863 | EPI1103041 | EPI1103396 | EPI1101402 | EPI971326 |  |  |  |  |  |  |  |
| EPI1103863 | EPI628543 | EPI1102258 | EPI1154771 | EPI971096 | EPI1090750 | EPI1101362 | EPI1100417 |  |  |  |  |  |  |  |
| EPI1063149 | EPI1090591 | EPI1102314 | EPI1102867 | EPI1101715 | EPI887933 | EPI1101394 | EPI971518 |  |  |  |  |  |  |  |
| EPI921554 | EPI1100446 | EPI1103892 | EPI1103941 | EPI1102641 | EPI887893 | EPI729454 | EPI1100409 |  |  |  |  |  |  |  |
| EPI1103823 | EPI1100454 | EPI1103884 | EPI1101005 | EPI1100971 | EPI912949 | EPI531804 | EPI1101937 |  |  |  |  |  |  |  |
| EPI1063155 | EPI1055407 | EPI1090746 | EPI1102159 | EPI887664 | EPI887925 | EPI729462 | EPI1055426 |  |  |  |  |  |  |  |
| EPI1103871 | EPI1103886 | EPI971273 | EPI1102182 | EPI1102067 | EPI912957 | EPI1055419 | EPI1103961 |  |  |  |  |  |  |  |
| EPI887932 | EPI1090527 | EPI1103401 | EPI1102707 | EPI1102075 | EPI942168 | EPI1055427 | EPI1102751 |  |  |  |  |  |  |  |
| EPI1063139 | EPI1055399 | EPI887609 | EPI1102443 | EPI1102417 | EPI627194 | EPI593472 | EPI1100497 |  |  |  |  |  |  |  |
| EPI887940 | EPI1101094 | EPI1102068 | EPI1101589 | EPI1102385 | EPI628761 | EPI593408 | EPI1100505 |  |  |  |  |  |  |  |
| EPI1063147 | EPI683031 | EPI1102076 | EPI1101037 | EPI1013229 | EPI656299 | EPI593551 | EPI1103913 |  |  |  |  |  |  |  |
| EPI912948 | EPI628735 | EPI1100460 | EPI1100765 | EPI887648 | EPI1102294 | EPI626996 | EPI1101297 |  |  |  |  |  |  |  |
| EPI887900 | EPI628767 | EPI1102682 | EPI1102755 | EPI1054146 | EPI1100456 | EPI656381 | EPI1101305 |  |  |  |  |  |  |  |
| EPI1063140 | EPI628551 | EPI1102157 | EPI1102747 | EPI887624 | EPI1054135 | EPI656309 | EPI1102049 |  |  |  |  |  |  |  |
| EPI1063161 | EPI1101198 | EPI1101004 | EPI971378 | EPI1102449 | EPI1100912 | EPI566079 | EPI1102033 |  |  |  |  |  |  |  |
| EPI1103260 | EPI1101430 | EPI1103940 | EPI1102363 | EPI1102993 | EPI1101472 | EPI1090200 | EPI1102041 |  |  |  |  |  |  |  |
| EPI1063150 | EPI1101206 | EPI1102181 | EPI1100757 | EPI1100811 | EPI1104016 | EPI627100 | EPI1101641 |  |  |  |  |  |  |  |
| EPI921766 | EPI628679 | EPI887737 | EPI971122 | EPI971112 | EPI1102678 | EPI627220 | EPI1101585 |  |  |  |  |  |  |  |
| EPI1103831 | EPI628791 | EPI1090586 | EPI1102723 | EPI1101723 | EPI922375 | EPI627252 | EPI1101617 |  |  |  |  |  |  |  |
| EPI1090573 | EPI628783 | EPI1102434 | EPI971130 | EPI887656 | EPI1103848 | EPI627308 | EPI887734 |  |  |  |  |  |  |  |
| EPI972241 | EPI628671 | EPI887673 | EPI1102683 | EPI1103105 | EPI1100440 | EPI769570 | EPI1102319 |  |  |  |  |  |  |  |
| EPI1101567 | EPI1101438 | EPI887809 | EPI971466 | EPI1102489 | EPI1100448 | EPI627292 | EPI1102295 |  |  |  |  |  |  |  |
| EPI1090773 | EPI942138 | EPI887689 | EPI971458 | EPI979553 | EPI1090238 | EPI566126 | EPI1103809 |  |  |  |  |  |  |  |
| EPI1102031 | EPI1101926 | EPI887761 | EPI1102779 | EPI1103049 | EPI833706 | EPI769578 | EPI1101121 |  |  |  |  |  |  |  |
| EPI972232 | EPI1100718 | EPI1103018 | EPI1102787 | EPI979561 | EPI1101448 | EPI627044 | EPI1101129 |  |  |  |  |  |  |  |
| EPI1101591 | EPI1101934 | EPI1102322 | EPI971442 | EPI1101459 | EPI1100824 | EPI656445 | EPI1102423 |  |  |  |  |  |  |  |
| EPI1101623 | EPI1103100 | EPI921759 | EPI971362 | EPI1102109 | EPI1100808 | EPI1057931 | EPI1102255 |  |  |  |  |  |  |  |
| EPI1101447 | EPI1102396 | EPI1103828 | EPI971354 | EPI1102201 | EPI1090366 | EPI656365 | EPI1102311 |  |  |  |  |  |  |  |
| EPI1101503 | EPI1102468 | EPI1090770 | EPI971370 | EPI1102425 | EPI1101600 | EPI627260 | EPI1090377 |  |  |  |  |  |  |  |
| EPI1101495 | EPI1102070 | EPI887825 | EPI1100429 | EPI1054106 | EPI942176 | EPI627108 | EPI1090593 |  |  |  |  |  |  |  |
| EPI1101471 | EPI1102078 | EPI887857 | EPI971426 | EPI1103057 | EPI917066 | EPI627212 | EPI1090689 |  |  |  |  |  |  |  |
| EPI887612 | EPI887667 | EPI887873 | EPI971434 | EPI945067 | EPI1090510 | EPI656397 | EPI1101921 |  |  |  |  |  |  |  |
| EPI971108 | EPI1102436 | EPI1103908 | EPI1090531 | EPI971080 | EPI1090518 | EPI1101626 | EPI1100713 |  |  |  |  |  |  |  |
| EPI971092 | EPI1102476 | EPI971233 | EPI1101445 | EPI971496 | EPI1102310 | EPI887839 | EPI1101929 |  |  |  |  |  |  |  |
| EPI887852 | EPI887763 | EPI971241 | EPI887802 | EPI971280 | EPI740731 | EPI1102864 | EPI979623 |  |  |  |  |  |  |  |
| EPI888084 | EPI1103020 | EPI887937 | EPI1103861 | EPI1103081 | EPI1090742 | EPI1101674 | EPI979631 |  |  |  |  |  |  |  |
| EPI961191 | EPI979620 | EPI1103924 | EPI922139 | EPI1100803 | EPI1103386 | EPI869668 | EPI1102799 |  |  |  |  |  |  |  |
| EPI1090493 | EPI1102492 | EPI1103222 | EPI1103845 | EPI887760 | EPI1055409 | EPI1101666 | EPI1102695 |  |  |  |  |  |  |  |
| EPI1101487 | EPI1103044 | EPI887729 | EPI1103869 | EPI1103017 | EPI1101120 | EPI926836 | EPI1102743 |  |  |  |  |  |  |  |
| EPI972244 | EPI1103068 | EPI1100588 | EPI887938 | EPI1103065 | EPI1103808 | EPI926844 | EPI922377 |  |  |  |  |  |  |  |
| EPI972261 | EPI887691 | EPI1101828 | EPI921762 | EPI979617 | EPI1103912 | EPI874333 | EPI1103849 |  |  |  |  |  |  |  |
| EPI1171792 | EPI979556 | EPI1103804 | EPI1103829 | EPI887688 | EPI1101128 | EPI977390 | EPI1102687 |  |  |  |  |  |  |  |
| EPI1100815 | EPI887627 | EPI1103266 | EPI921522 | EPI1103097 | EPI1102318 | EPI656437 | EPI971126 |  |  |  |  |  |  |  |
| EPI972243 | EPI945070 | EPI971249 | EPI1103821 | EPI1054098 | EPI1102254 | EPI1101282 | EPI1102287 |  |  |  |  |  |  |  |
| EPI1101607 | EPI979564 | EPI971313 | EPI887898 | EPI1102401 | EPI1101416 | EPI682982 | EPI1102703 |  |  |  |  |  |  |  |
| EPI1100839 | EPI887651 | EPI1102514 | EPI912946 | EPI1103089 | EPI1103896 | EPI682983 | EPI971118 |  |  |  |  |  |  |  |
| EPI1100703 | EPI979548 | EPI1103190 | EPI887930 | EPI1154772 | EPI1090446 | EPI1100738 | EPI1102719 |  |  |  |  |  |  |  |
| EPI1100711 | EPI971083 | EPI887777 | EPI912954 | EPI926853 | EPI1102534 | EPI1100746 | EPI1100761 |  |  |  |  |  |  |  |
| EPI1100935 | EPI1054149 | EPI1100612 | EPI979639 | EPI1102937 | EPI1090462 | EPI1100722 | EPI1090241 |  |  |  |  |  |  |  |
| EPI1100951 | EPI1102428 | EPI1102498 | EPI1102459 | EPI985840 | EPI1102550 | EPI1102896 | EPI833709 |  |  |  |  |  |  |  |
| EPI1100943 | EPI1101462 | EPI971209 | EPI1100709 | EPI971184 | EPI1103968 | EPI1102880 | EPI1101449 |  |  |  |  |  |  |  |
| EPI1100927 | EPI1102940 | EPI971217 | EPI1054140 | EPI926861 | EPI1103984 | EPI1102888 | EPI912960 |  |  |  |  |  |  |  |
| EPI1101919 | EPI926856 | EPI1100556 | EPI1100917 | EPI971488 | EPI1102926 | EPI1100754 | EPI887894 |  |  |  |  |  |  |  |
| EPI1054142 | EPI971099 | EPI1101812 | EPI971450 | EPI979513 | EPI1101296 | EPI1102848 | EPI912952 |  |  |  |  |  |  |  |
| EPI1100919 | EPI1102404 | EPI1100604 | EPI1101565 | EPI887976 | EPI1101304 | EPI1103231 | EPI1103366 |  |  |  |  |  |  |  |
| EPI1101551 | EPI971499 | EPI1101788 | EPI1100461 | EPI872954 | EPI1100408 | EPI887855 | EPI1102519 |  |  |  |  |  |  |  |
| EPI971340 | EPI971491 | EPI1101844 | EPI1101941 | EPI1101619 | EPI971325 | EPI922376 | EPI1102503 |  |  |  |  |  |  |  |
| EPI971348 | EPI926864 | EPI1100620 | EPI1100661 | EPI1101475 | EPI1100416 | EPI1103850 | EPI971502 |  |  |  |  |  |  |  |
| EPI971444 | EPI1054101 | EPI1101820 | EPI1100877 | EPI1104019 | EPI971445 | EPI887823 | EPI887798 |  |  |  |  |  |  |  |
| EPI971364 | EPI1054109 | EPI971193 | EPI1101853 | EPI1055412 | EPI971453 | EPI887871 | EPI1101097 |  |  |  |  |  |  |  |
| EPI971356 | EPI1154775 | EPI1101292 | EPI1103877 | EPI971376 | EPI971461 | EPI1101098 | EPI1101105 |  |  |  |  |  |  |  |
| EPI971372 | EPI1102420 | EPI971161 | EPI1101901 | EPI971336 | EPI1100640 | EPI1101106 | EPI1101625 |  |  |  |  |  |  |  |
| EPI971404 | EPI1103012 | EPI971177 | EPI1160155 | EPI971344 | EPI1100648 | EPI887607 | EPI971270 |  |  |  |  |  |  |  |
| EPI1102445 | EPI887659 | EPI971169 | EPI1010113 | EPI887680 | EPI971509 | EPI971087 | EPI1102335 |  |  |  |  |  |  |  |
| EPI888076 | EPI1103092 | EPI1100908 | EPI1102531 | EPI922373 | EPI1102582 | EPI971103 | EPI1103881 |  |  |  |  |  |  |  |
| EPI1102413 | EPI971115 | EPI1102906 | EPI1100693 | EPI1103851 | EPI1102574 | EPI1101298 | EPI1101113 |  |  |  |  |  |  |  |
| EPI1102365 | EPI1103060 | EPI1102914 | EPI1102283 | EPI887824 | EPI1101936 | EPI1101306 | EPI1101441 |  |  |  |  |  |  |  |
| EPI888004 | EPI1102452 | EPI971201 | EPI1102403 | EPI887856 | EPI1102016 | EPI971319 | EPI887926 |  |  |  |  |  |  |  |
| EPI1102349 | EPI972251 | EPI1100892 | EPI1102235 | EPI887872 | EPI1100480 | EPI971231 | EPI971182 |  |  |  |  |  |  |  |
| EPI1100471 | EPI1103052 | EPI1101524 | EPI1102243 | EPI1101507 | EPI1100536 | EPI971239 | EPI926859 |  |  |  |  |  |  |  |
| EPI1100479 | EPI1102113 | EPI1102658 | EPI1101893 | EPI1103915 | EPI971517 | EPI1103906 | EPI1102935 |  |  |  |  |  |  |  |
| EPI1100463 | EPI971187 | EPI1101348 | EPI1102923 | EPI1103907 | EPI1100832 | EPI1103368 | EPI1154777 |  |  |  |  |  |  |  |
| EPI1157902 | EPI985837 | EPI1101356 | EPI1100885 | EPI971232 | EPI866538 | EPI971503 | EPI971486 |  |  |  |  |  |  |  |
| EPI971412 | EPI979643 | EPI1101332 | EPI1100581 | EPI971240 | EPI1101536 | EPI1102504 | EPI926851 |  |  |  |  |  |  |  |
| EPI888028 | EPI1102988 | EPI1101340 | EPI1100493 | EPI971504 | EPI1100816 | EPI1102520 | EPI985839 |  |  |  |  |  |  |  |
| EPI1101975 | EPI1102388 | EPI1101324 | EPI1100597 | EPI1103369 | EPI1101480 | EPI1101850 | EPI1103905 |  |  |  |  |  |  |  |
| EPI971260 | EPI1054133 | EPI1101316 | EPI1100565 | EPI1102505 | EPI961188 | EPI1101242 | EPI1102130 |  |  |  |  |  |  |  |
| EPI1100959 | EPI971283 | EPI1102650 | EPI1100573 | EPI1102521 | EPI833715 | EPI1102264 | EPI1103349 |  |  |  |  |  |  |  |
| EPI971300 | EPI1102644 | EPI1102666 | EPI1100685 | EPI979625 | EPI1102024 | EPI1102280 | EPI1090369 |  |  |  |  |  |  |  |
| EPI971476 | EPI1101774 | EPI1101036 | EPI1102267 | EPI887704 | EPI1101464 | EPI1102872 | EPI979575 |  |  |  |  |  |  |  |
| EPI971484 | EPI1102996 | EPI1101052 | EPI1100669 | EPI1102337 | EPI1102048 | EPI1101226 | EPI1102065 |  |  |  |  |  |  |  |
| EPI1102253 | EPI1100974 | EPI1101076 | EPI1101909 | EPI887952 | EPI1101496 | EPI1101898 | EPI1102073 |  |  |  |  |  |  |  |
| EPI1103959 | EPI1103084 | EPI1101084 | EPI1101837 | EPI888064 | EPI1100520 | EPI1101218 | EPI1103284 |  |  |  |  |  |  |  |
| EPI1103999 | EPI1103108 | EPI1101068 | EPI1101869 | EPI1101115 | EPI1090574 | EPI1100658 | EPI1103865 |  |  |  |  |  |  |  |
| EPI1101807 | EPI1101718 | EPI1101044 | EPI1101861 | EPI1102329 | EPI1100928 | EPI1100874 | EPI1103825 |  |  |  |  |  |  |  |
| EPI1104007 | EPI1101726 | EPI1101060 | EPI1100901 | EPI1103221 | EPI1100704 | EPI1101882 | EPI921902 |  |  |  |  |  |  |  |
| EPI888060 | EPI1100806 | EPI1100444 | EPI1101877 | EPI1103265 | EPI1101912 | EPI1103874 | EPI887934 |  |  |  |  |  |  |  |
| EPI888052 | EPI1102204 | EPI1100452 | EPI1102379 | EPI1103189 | EPI1100936 | EPI1160160 | EPI1103253 |  |  |  |  |  |  |  |
| EPI888020 | EPI552401 | EPI1102133 | EPI1103989 | EPI971208 | EPI1100920 | EPI1100682 | EPI1103857 |  |  |  |  |  |  |  |
| EPI971436 | EPI627184 | EPI1103352 | EPI979579 | EPI971248 | EPI1100944 | EPI1101866 | EPI922227 |  |  |  |  |  |  |  |
| EPI888012 | EPI1101446 | EPI1022662 | EPI1101277 | EPI887776 | EPI1100432 | EPI1100898 | EPI1103841 |  |  |  |  |  |  |  |
| EPI1100431 | EPI1102548 | EPI1013246 | EPI1101269 | EPI1103923 | EPI971469 | EPI1101890 | EPI921629 |  |  |  |  |  |  |  |
| EPI971428 | EPI1090615 | EPI1018165 | EPI1101253 | EPI971200 | EPI971477 | EPI1100882 | EPI1103817 |  |  |  |  |  |  |  |
| EPI1102581 | EPI1103383 | EPI1090682 | EPI1102627 | EPI1102513 | EPI971253 | EPI1100666 | EPI971230 |  |  |  |  |  |  |  |
| EPI1101951 | EPI712925 | EPI712923 | EPI1101629 | EPI971168 | EPI1100952 | EPI1101906 | EPI1101497 |  |  |  |  |  |  |  |
| EPI1101959 | EPI1090687 | EPI979578 | EPI979603 | EPI887728 | EPI1102246 | EPI1100490 | EPI1090777 |  |  |  |  |  |  |  |
| EPI888044 | EPI1055415 | EPI1100500 | EPI1101613 | EPI971312 | EPI1101968 | EPI1100562 | EPI971238 |  |  |  |  |  |  |  |
| EPI887988 | EPI1090511 | EPI1100508 | EPI887978 | EPI971160 | EPI1103952 | EPI1100690 | EPI887670 |  |  |  |  |  |  |  |
| EPI971380 | EPI1101502 | EPI1101300 | EPI872956 | EPI1101827 | EPI1104000 | EPI1100578 | EPI887806 |  |  |  |  |  |  |  |
| EPI971388 | EPI917067 | EPI1101308 | EPI1101645 | EPI1100587 | EPI1103992 | EPI1101858 | EPI887758 |  |  |  |  |  |  |  |
| EPI971396 | EPI1171793 | EPI971505 | EPI1101581 | EPI1100611 | EPI971293 | EPI1102920 | EPI1103015 |  |  |  |  |  |  |  |
| EPI971324 | EPI1100710 | EPI1103370 | EPI979595 | EPI1102497 | EPI1101800 | EPI1101874 | EPI887662 |  |  |  |  |  |  |  |
| EPI888036 | EPI1101486 | EPI1102506 | EPI1013271 | EPI1103803 | EPI888021 | EPI1100570 | EPI1102391 |  |  |  |  |  |  |  |
| EPI971420 | EPI1101918 | EPI1102522 | EPI979515 | EPI971216 | EPI971405 | EPI1100594 | EPI1102471 |  |  |  |  |  |  |  |
| EPI1100783 | EPI1100934 | EPI1101444 | EPI926839 | EPI1100907 | EPI1102406 | EPI1101834 | EPI1103095 |  |  |  |  |  |  |  |
| EPI1104063 | EPI1100942 | EPI1101100 | EPI874336 | EPI1102905 | EPI1102342 | EPI1010118 | EPI1102463 |  |  |  |  |  |  |  |
| EPI1102885 | EPI1101494 | EPI1101108 | EPI977393 | EPI1102913 | EPI888069 | EPI1102528 | EPI1101001 |  |  |  |  |  |  |  |
| EPI1102893 | EPI930817 | EPI1102330 | EPI1100797 | EPI971192 | EPI887997 | EPI627028 | EPI1102177 |  |  |  |  |  |  |  |
| EPI1100759 | EPI926840 | EPI1101180 |  | EPI971176 | EPI971317 | EPI627012 | EPI1102154 |  |  |  |  |  |  |  |
| EPI1100727 | EPI1018258 | EPI1101172 |  | EPI1103232 | EPI1100424 | EPI627364 | EPI1102863 |  |  |  |  |  |  |  |
| EPI1102853 | EPI1101686 | EPI1103212 |  | EPI1100891 | EPI888045 | EPI627356 | EPI1103937 |  |  |  |  |  |  |  |
| EPI1100743 | EPI1102956 | EPI1100996 |  | EPI1101355 | EPI888053 | EPI769642 | EPI1040445 |  |  |  |  |  |  |  |
| EPI1100751 | EPI1102948 | EPI1101140 |  | EPI1101331 | EPI887981 | EPI560401 | EPI1100809 |  |  |  |  |  |  |  |
| EPI1102901 | EPI1102964 | EPI1100988 |  | EPI1101347 | EPI1101944 | EPI769586 | EPI1102983 |  |  |  |  |  |  |  |
| EPI1102869 | EPI1171787 | EPI1100980 |  | EPI1101323 | EPI1101952 | EPI1101498 | EPI1103007 |  |  |  |  |  |  |  |
| EPI1103943 | EPI972240 | EPI1102145 |  | EPI1101315 | EPI888037 | EPI628747 | EPI1103889 |  |  |  |  |  |  |  |
| EPI1102162 | EPI1101566 | EPI1100532 |  | EPI1101339 | EPI888005 | EPI971335 | EPI1103398 |  |  |  |  |  |  |  |
| EPI1102186 | EPI1101478 | EPI1102100 |  | EPI1101995 | EPI888013 | EPI971343 | EPI1090617 |  |  |  |  |  |  |  |
| EPI1101007 | EPI1104022 | EPI1102092 |  | EPI1101523 | EPI971373 | EPI654133 | EPI1090753 |  |  |  |  |  |  |  |
| EPI1102541 | EPI833716 | EPI1102210 |  | EPI1102657 | EPI1100464 | EPI627148 | EPI887742 |  |  |  |  |  |  |  |
| EPI1100503 | EPI1101134 | EPI887705 |  | EPI1102649 | EPI1100472 | EPI627140 | EPI1103357 |  |  |  |  |  |  |  |
| EPI1100511 | EPI1102324 | EPI979626 |  | EPI1102665 | EPI971381 | EPI942108 | EPI887878 |  |  |  |  |  |  |  |
| EPI1036698 | EPI1102046 | EPI1103916 |  | EPI1101035 | EPI971389 | EPI1100434 | EPI1103945 |  |  |  |  |  |  |  |
| EPI1103991 | EPI1101390 | EPI887865 |  | EPI1101051 | EPI888029 | EPI1100522 | EPI887862 |  |  |  |  |  |  |  |
| EPI1036694 | EPI1101422 | EPI887881 |  | EPI1101059 | EPI971413 | EPI979568 | EPI1101153 |  |  |  |  |  |  |  |
| EPI1103975 | EPI740737 | EPI887889 |  | EPI1101067 | EPI1100744 | EPI942116 | EPI887886 |  |  |  |  |  |  |  |
| EPI1102933 | EPI1101406 | EPI1101156 |  | EPI1101083 | EPI1100752 | EPI1100706 | EPI1103306 |  |  |  |  |  |  |  |
| EPI1090461 | EPI866552 | EPI1101164 |  | EPI1101043 | EPI1102894 | EPI1102552 | EPI1101145 |  |  |  |  |  |  |  |
| EPI1103983 | EPI1101542 | EPI1101148 |  | EPI1101075 | EPI1104056 | EPI1102026 | EPI887678 |  |  |  |  |  |  |  |
| EPI1036685 | EPI1101366 | EPI1103288 |  | EPI887744 | EPI1101024 | EPI1102536 | EPI887958 |  |  |  |  |  |  |  |
| EPI1102174 | EPI1101382 | EPI1102234 |  | EPI1101163 | EPI979566 | EPI1054137 | EPI1100545 |  |  |  |  |  |  |  |
| EPI1102557 | EPI1102038 | EPI1102242 |  | EPI1102713 | EPI866546 | EPI1100914 | EPI1101177 |  |  |  |  |  |  |  |
| EPI1103903 | EPI1102054 | EPI1103948 |  | EPI1100491 | EPI1101488 | EPI866540 | EPI1102599 |  |  |  |  |  |  |  |
| EPI1101423 | EPI1090519 | EPI887969 |  | EPI887912 | EPI1101544 | EPI866556 | EPI1103208 |  |  |  |  |  |  |  |
| EPI1090445 | EPI1101030 | EPI979522 |  | EPI1101155 | EPI866554 | EPI1102928 | EPI887782 |  |  |  |  |  |  |  |
| EPI1101391 | EPI1100526 | EPI1101188 |  | EPI1104147 | EPI1101560 | EPI1103388 | EPI1102089 |  |  |  |  |  |  |  |
| EPI1101415 | EPI942146 | EPI1101228 |  | EPI1102281 | EPI1102326 | EPI1100946 | EPI1102097 |  |  |  |  |  |  |  |
| EPI1101375 | EPI1090575 | EPI1101244 |  | EPI1102705 | EPI1103228 | EPI866548 | EPI1101137 |  |  |  |  |  |  |  |
| EPI1101407 | EPI1101414 | EPI1101260 |  | EPI1101835 | EPI1103240 | EPI1101466 | EPI1102142 |  |  |  |  |  |  |  |
| EPI1101399 | EPI1100702 | EPI1103310 |  | EPI1102120 | EPI1103920 | EPI1090448 | EPI1100993 |  |  |  |  |  |  |  |
| EPI1101367 | EPI866544 | EPI1101212 |  | EPI1101859 | EPI887949 | EPI1100834 | EPI1102207 |  |  |  |  |  |  |  |
| EPI1101383 | EPI1101374 | EPI979530 |  | EPI1101867 | EPI1102518 | EPI1101538 | EPI1100529 |  |  |  |  |  |  |  |
| EPI1101303 | EPI1101398 | EPI1101236 |  | EPI979529 | EPI1102350 | EPI1100938 | EPI1102188 |  |  |  |  |  |  |  |
| EPI1101311 | EPI1100822 | EPI1101124 |  | EPI1103343 | EPI1102494 | EPI1103898 | EPI1101169 |  |  |  |  |  |  |  |
| EPI971332 | EPI1090495 | EPI979538 |  | EPI1103025 | EPI971309 | EPI1101418 | EPI1100585 |  |  |  |  |  |  |  |
| EPI1100415 | EPI926832 | EPI1103344 |  | EPI1104091 | EPI1103348 | EPI1100930 | EPI1101825 |  |  |  |  |  |  |  |
| EPI1100423 | EPI869672 | EPI1101116 |  | EPI1100883 | EPI971157 | EPI1100818 | EPI1102495 |  |  |  |  |  |  |  |
| EPI971452 | EPI1101670 | EPI1103256 |  | EPI979537 | EPI887957 | EPI1101914 | EPI1100609 |  |  |  |  |  |  |  |
| EPI971460 | EPI1101678 | EPI1103860 |  | EPI1103287 | EPI1103185 | EPI961190 | EPI1103187 |  |  |  |  |  |  |  |
| EPI971468 | EPI926848 | EPI921491 |  | EPI1101891 | EPI1101824 | EPI1090744 | EPI1103229 |  |  |  |  |  |  |  |
| EPI1102589 | EPI977394 | EPI1103820 |  | EPI1102977 | EPI1100584 | EPI1101026 | EPI1102271 |  |  |  |  |  |  |  |
| EPI1157900 | EPI962899 | EPI1103201 |  | EPI971288 | EPI1102128 | EPI1101546 | EPI1103219 |  |  |  |  |  |  |  |
| EPI971524 | EPI1001899 | EPI887641 |  | EPI1101171 | EPI887941 | EPI1100922 | EPI887774 |  |  |  |  |  |  |  |
| EPI971516 | EPI874337 | EPI1102122 |  | EPI887792 | EPI887725 | EPI833717 | EPI887726 |  |  |  |  |  |  |  |
| EPI1157901 | EPI1040956 | EPI887745 |  | EPI1102633 | EPI1103800 | EPI1090496 | EPI1103801 |  |  |  |  |  |  |  |
| EPI1100543 | EPI1103902 | EPI922004 |  | EPI1104011 | EPI971213 | EPI971471 | EPI1103262 |  |  |  |  |  |  |  |
| EPI1102023 | EPI1090743 | EPI1103836 |  | EPI1010114 | EPI1102510 | EPI1103994 | EPI1103242 |  |  |  |  |  |  |  |
| EPI1100487 | EPI1103393 | EPI1103932 |  | EPI1103200 | EPI971173 | EPI1100954 | EPI1103921 |  |  |  |  |  |  |  |
| EPI1101943 | EPI887675 | EPI971225 |  | EPI1101283 | EPI1102374 | EPI1104002 | EPI1102327 |  |  |  |  |  |  |  |
| EPI1100647 | EPI887811 | EPI1103812 |  | EPI887920 | EPI1103218 | EPI971479 | EPI1102375 |  |  |  |  |  |  |  |
| EPI1100655 | EPI972231 | EPI922138 |  | EPI1102265 | EPI1100608 | EPI1101802 | EPI1102351 |  |  |  |  |  |  |  |
| EPI1103395 | EPI1101590 | EPI1103844 |  | EPI1102609 | EPI1103070 | EPI1102248 | EPI1103071 |  |  |  |  |  |  |  |
| EPI1090741 | EPI1101622 | EPI1103234 |  | EPI1102617 | EPI1102478 | EPI971295 | EPI1102479 |  |  |  |  |  |  |  |
| EPI887676 | EPI1100926 | EPI922269 |  | EPI1103001 | EPI888061 | EPI1103954 | EPI1102367 |  |  |  |  |  |  |  |
| EPI887812 | EPI1090775 | EPI1103852 |  | EPI1102681 | EPI1103904 | EPI971255 | EPI1102999 |  |  |  |  |  |  |  |
| EPI1101575 | EPI1102030 | EPI887921 |  | EPI887816 | EPI1102694 | EPI888055 | EPI1103031 |  |  |  |  |  |  |  |
| EPI1100791 | EPI1090447 | EPI1103298 |  | EPI1102801 | EPI1102502 | EPI888047 | EPI1103197 |  |  |  |  |  |  |  |
| EPI1100799 | EPI1100502 | EPI1102714 |  | EPI1101179 | EPI1103261 | EPI971383 | EPI1054120 |  |  |  |  |  |  |  |
| EPI1018257 | EPI1100510 | EPI1102794 |  | EPI1100899 | EPI979574 | EPI888039 | EPI1103317 |  |  |  |  |  |  |  |
| EPI1101687 | EPI1103974 | EPI1102770 |  | EPI887968 | EPI1102366 | EPI971391 | EPI1102855 |  |  |  |  |  |  |  |
| EPI1102949 | EPI1036695 | EPI1102778 |  | EPI1102289 | EPI971197 | EPI971415 | EPI1102839 |  |  |  |  |  |  |  |
| EPI1102957 | EPI1103990 | EPI1102858 |  | EPI1101883 | EPI1102270 | EPI888031 | EPI1102767 |  |  |  |  |  |  |  |
| EPI1102965 | EPI1101302 | EPI1102762 |  | EPI1102873 | EPI1013242 | EPI888007 | EPI1102791 |  |  |  |  |  |  |  |
| EPI1102325 | EPI1101310 | EPI1102842 |  | EPI1102921 | EPI1018161 | EPI1101970 | EPI1102759 |  |  |  |  |  |  |  |
| EPI1103815 | EPI1090463 | EPI1102834 |  | EPI1102083 | EPI1022626 | EPI888015 | EPI1102807 |  |  |  |  |  |  |  |
| EPI1102301 | EPI1102172 | EPI1102826 |  | EPI887640 | EPI887885 | EPI887983 | EPI1104065 |  |  |  |  |  |  |  |
| EPI1102261 | EPI1036687 | EPI1102738 |  | EPI1100875 | EPI887877 | EPI1101946 | EPI1104073 |  |  |  |  |  |  |  |
| EPI1102317 | EPI1103982 | EPI1102786 |  | EPI1101851 | EPI887861 | EPI1101954 | EPI1104137 |  |  |  |  |  |  |  |
| EPI1101135 | EPI1102868 | EPI1102818 |  | EPI1100659 | EPI1101144 | EPI1104058 | EPI1100769 |  |  |  |  |  |  |  |
| EPI1102429 | EPI1101006 | EPI1103328 |  | EPI1103875 | EPI971501 | EPI1100778 | EPI1101777 |  |  |  |  |  |  |  |
| EPI1103364 | EPI1103942 | EPI887681 |  | EPI1101899 | EPI1103365 | EPI888023 | EPI1101985 |  |  |  |  |  |  |  |
| EPI887804 | EPI1102160 | EPI1102610 |  | EPI1160156 | EPI971245 | EPI971407 | EPI1102567 |  |  |  |  |  |  |  |
| EPI887876 | EPI1102183 | EPI1102618 |  | EPI887752 | EPI971229 | EPI1100466 | EPI1101977 |  |  |  |  |  |  |  |
| EPI1063143 | EPI1102540 | EPI1101836 |  | EPI1104115 | EPI971237 | EPI1100474 | EPI1101993 |  |  |  |  |  |  |  |
| EPI887860 | EPI1102556 | EPI1102282 |  | EPI1104155 | EPI887773 | EPI888071 | EPI1101729 |  |  |  |  |  |  |  |
| EPI1063141 | EPI1102932 | EPI1101860 |  | EPI1104131 | EPI1101168 | EPI971399 | EPI1102009 |  |  |  |  |  |  |  |
| EPI887828 | EPI1100486 | EPI1100692 |  | EPI971144 | EPI1101176 | EPI971359 | EPI1102223 |  |  |  |  |  |  |  |
| EPI1063153 | EPI971467 | EPI1102922 |  | EPI971152 | EPI1100544 | EPI971439 | EPI1102001 |  |  |  |  |  |  |  |
| EPI922374 | EPI971459 | EPI1100884 |  | EPI922044 | EPI1102598 | EPI1102360 | EPI1104097 |  |  |  |  |  |  |  |
| EPI1103855 | EPI1100646 | EPI1101892 |  | EPI1103835 | EPI1100904 | EPI1102344 | EPI1101705 |  |  |  |  |  |  |  |
| EPI1101103 | EPI1100654 | EPI1102874 |  | EPI1101187 | EPI1102902 | EPI971367 | EPI1101793 |  |  |  |  |  |  |  |
| EPI1101111 | EPI971451 | EPI1101884 |  | EPI971264 | EPI1102910 | EPI971351 | EPI1100729 |  |  |  |  |  |  |  |
| EPI887708 | EPI971523 | EPI1100572 |  | EPI971224 | EPI971189 | EPI887999 | EPI1102559 |  |  |  |  |  |  |  |
| EPI1101127 | EPI1102022 | EPI1100564 |  | EPI887944 | EPI971165 | EPI1102408 | EPI1101689 |  |  |  |  |  |  |  |
| EPI979629 | EPI1100422 | EPI1100596 |  | EPI1101875 | EPI971205 | EPI1102856 | EPI1101745 |  |  |  |  |  |  |  |
| EPI888068 | EPI1101942 | EPI1100492 |  | EPI1100691 | EPI1101520 | EPI740733 | EPI1101697 |  |  |  |  |  |  |  |
| EPI1103077 | EPI971515 | EPI1101876 |  | EPI887768 | EPI1102646 | EPI1090688 | EPI1104105 |  |  |  |  |  |  |  |
| EPI1063148 | EPI1100414 | EPI1102530 |  | EPI1100683 | EPI1102662 | EPI979576 | EPI1100961 |  |  |  |  |  |  |  |
| EPI887956 | EPI971331 | EPI1100668 |  | EPI971120 | EPI1100632 | EPI979632 | EPI1104081 |  |  |  |  |  |  |  |
| EPI1102341 | EPI1100542 | EPI1101908 |  | EPI971128 | EPI1100600 | EPI1090528 | EPI1104121 |  |  |  |  |  |  |  |
| EPI1100983 | EPI1100838 | EPI1100580 |  | EPI1103327 | EPI1101808 | EPI1055395 | EPI971134 |  |  |  |  |  |  |  |
| EPI1100991 | EPI1100950 | EPI1101868 |  | EPI1101291 | EPI1100672 | EPI1101090 | EPI1101737 |  |  |  |  |  |  |  |
| EPI887788 | EPI1054141 | EPI1100876 |  | EPI1100547 | EPI1101784 | EPI887799 | EPI1104025 |  |  |  |  |  |  |  |
| EPI1102095 | EPI1100918 | EPI1010112 |  | EPI1102689 | EPI1100616 | EPI1101442 | EPI1036693 |  |  |  |  |  |  |  |
| EPI1102151 | EPI961189 | EPI1100684 |  | EPI1100579 | EPI1100624 | EPI1055411 | EPI971286 |  |  |  |  |  |  |  |
| EPI1102213 | EPI979572 | EPI1102266 |  | EPI1102601 | EPI1101816 | EPI1090520 | EPI1101753 |  |  |  |  |  |  |  |
| EPI1100535 | EPI972242 | EPI1100900 |  | EPI1102697 | EPI1100552 | EPI1090512 | EPI1104089 |  |  |  |  |  |  |  |
| EPI1101143 | EPI1101606 | EPI1101852 |  | EPI887696 | EPI1101840 | EPI1103399 | EPI1101761 |  |  |  |  |  |  |  |
| EPI1100999 | EPI974029 | EPI1103276 |  | EPI887712 | EPI1100888 | EPI1057955 | EPI1104049 |  |  |  |  |  |  |  |
| EPI1102104 | EPI1100814 | EPI1103026 |  | EPI887720 | EPI1102654 | EPI1090592 | EPI1101961 |  |  |  |  |  |  |  |
| EPI1102197 | EPI972260 | EPI971265 |  | EPI887616 | EPI1101352 | EPI1101490 | EPI1101513 |  |  |  |  |  |  |  |
| EPI1063164 | EPI866560 | EPI1101508 |  | EPI1103275 | EPI1101344 | EPI887703 | EPI1104033 |  |  |  |  |  |  |  |
| EPI1103217 | EPI1101550 | EPI887617 |  | EPI1103297 | EPI1101328 | EPI887959 | EPI1102215 |  |  |  |  |  |  |  |
| EPI979581 | EPI887851 | EPI1103034 |  | EPI1104107 | EPI1101336 | EPI1103342 | EPI1104145 |  |  |  |  |  |  |  |
| EPI887892 | EPI888083 | EPI1103336 |  | EPI1104139 | EPI1101312 | EPI1103358 | EPI1104009 |  |  |  |  |  |  |  |
| EPI1103919 | EPI887611 | EPI1102458 |  | EPI1054122 | EPI1101320 | EPI887879 | EPI1104041 |  |  |  |  |  |  |  |
| EPI1103347 | EPI971107 | EPI888065 |  | EPI1104099 | EPI1101048 | EPI1103334 | EPI979535 |  |  |  |  |  |  |  |
| EPI887964 | EPI971091 | EPI1102634 |  | EPI1103947 | EPI1101064 | EPI887887 | EPI1103333 |  |  |  |  |  |  |  |
| EPI887884 | EPI1101470 | EPI887793 |  | EPI1103033 | EPI1101080 | EPI887863 | EPI887950 |  |  |  |  |  |  |  |
| EPI1101151 | EPI1100438 | EPI1102978 |  | EPI921531 | EPI1101056 | EPI1103307 | EPI1102975 |  |  |  |  |  |  |  |
| EPI887868 | EPI1100470 | EPI1103868 |  | EPI1103819 | EPI1101040 | EPI1090752 | EPI1102081 |  |  |  |  |  |  |  |
| EPI1063142 | EPI1100478 | EPI887913 |  | EPI971136 | EPI1101072 | EPI887663 | EPI1103295 |  |  |  |  |  |  |  |
| EPI1102485 | EPI1100462 | EPI887993 |  | EPI1104123 | EPI1103207 | EPI1102432 | EPI1103272 |  |  |  |  |  |  |  |
| EPI1102381 | EPI1102444 | EPI887769 |  | EPI1102233 | EPI1100528 | EPI1102472 | EPI1102671 |  |  |  |  |  |  |  |
| EPI1102357 | EPI1102412 | EPI1103002 |  | EPI1102241 | EPI1101136 | EPI887783 | EPI887814 |  |  |  |  |  |  |  |
| EPI1103250 | EPI1102364 | EPI887953 |  | EPI1100763 | EPI1100976 | EPI1090776 | EPI1101161 |  |  |  |  |  |  |  |
| EPI1063159 | EPI1102348 | EPI1102084 |  | EPI1102753 | EPI1100984 | EPI1055403 | EPI979519 |  |  |  |  |  |  |  |
| EPI1102373 | EPI971323 | EPI887817 |  | EPI1102721 | EPI887781 | EPI1103890 | EPI887942 |  |  |  |  |  |  |  |
| EPI1102333 | EPI971403 | EPI887697 |  | EPI1102745 | EPI1102206 | EPI1090616 | EPI887766 |  |  |  |  |  |  |  |
| EPI1103807 | EPI888003 | EPI1101708 |  | EPI1102729 | EPI1102088 | EPI1102042 | EPI1103833 |  |  |  |  |  |  |  |
| EPI971204 | EPI888075 | EPI1101764 |  | EPI1102793 | EPI1102096 | EPI1102034 | EPI887910 |  |  |  |  |  |  |  |
| EPI971316 | EPI971411 | EPI1101756 |  | EPI1102857 | EPI1100992 | EPI1102050 | EPI888062 |  |  |  |  |  |  |  |
| EPI1102517 | EPI888027 | EPI1101748 |  | EPI1102769 | EPI1102140 | EPI887847 | EPI922084 |  |  |  |  |  |  |  |
| EPI1100911 | EPI1100958 | EPI1101740 |  | EPI1102761 | EPI922190 | EPI888079 | EPI887918 |  |  |  |  |  |  |  |
| EPI1102909 | EPI1101958 | EPI971137 |  | EPI1102809 | EPI1103840 | EPI1103970 | EPI971246 |  |  |  |  |  |  |  |
| EPI1102917 | EPI1101950 | EPI971289 |  | EPI1102825 | EPI921570 | EPI1036696 | EPI887750 |  |  |  |  |  |  |  |
| EPI887732 | EPI1102580 | EPI1104124 |  | EPI1102833 | EPI1103816 | EPI1103986 | EPI887902 |  |  |  |  |  |  |  |
| EPI971180 | EPI1102588 | EPI1104140 |  | EPI1102777 | EPI1103252 | EPI628555 | EPI1102607 |  |  |  |  |  |  |  |
| EPI971212 | EPI887987 | EPI1104148 |  | EPI1102841 | EPI1103856 | EPI1102320 | EPI1103325 |  |  |  |  |  |  |  |
| EPI971172 | EPI971379 | EPI1104092 |  | EPI1100595 | EPI887677 | EPI1102312 | EPI971142 |  |  |  |  |  |  |  |
| EPI971196 | EPI1100430 | EPI1104068 |  | EPI1100571 | EPI1102334 | EPI1101130 | EPI971222 |  |  |  |  |  |  |  |
| EPI1102277 | EPI971427 | EPI1104076 |  | EPI1100563 | EPI1102670 | EPI1100530 | EPI971150 |  |  |  |  |  |  |  |
| EPI971220 | EPI971435 | EPI1104052 |  | EPI887992 | EPI887901 | EPI1102189 | EPI1102118 |  |  |  |  |  |  |  |
| EPI1103270 | EPI888019 | EPI1104100 |  | EPI1102529 | EPI1102686 | EPI1102131 | EPI1104153 |  |  |  |  |  |  |  |
| EPI1103239 | EPI888051 | EPI1104108 |  | EPI1103255 | EPI1102702 | EPI1103350 | EPI887638 |  |  |  |  |  |  |  |
| EPI1103927 | EPI888059 | EPI1102218 |  | EPI1103859 | EPI1102710 | EPI1022660 | EPI1103929 |  |  |  |  |  |  |  |
| EPI1063158 | EPI888011 | EPI1104012 |  | EPI1102457 | EPI971117 | EPI1013244 | EPI979527 |  |  |  |  |  |  |  |
| EPI887780 | EPI888043 | EPI1102562 |  | EPI1101907 | EPI1102718 | EPI1018163 | EPI1104129 |  |  |  |  |  |  |  |
| EPI1063160 | EPI888035 | EPI1101796 |  | EPI1100667 | EPI971125 | EPI1101170 | EPI1101505 |  |  |  |  |  |  |  |
| EPI971164 | EPI971419 | EPI1104084 |  | EPI979521 | EPI1102798 | EPI1100986 | EPI1102615 |  |  |  |  |  |  |  |
| EPI1103227 | EPI971387 | EPI1101516 |  | EPI1103931 | EPI1102734 | EPI1100978 | EPI887966 |  |  |  |  |  |  |  |
| EPI1063156 | EPI971395 | EPI1104044 |  | EPI1102225 | EPI1102782 | EPI1100546 | EPI1104113 |  |  |  |  |  |  |  |
| EPI1103195 | EPI1100782 | EPI1102226 |  | EPI1102561 | EPI1102766 | EPI1101138 | EPI1103341 |  |  |  |  |  |  |  |
| EPI1101831 | EPI1104062 | EPI1104028 |  | EPI1102569 | EPI1102774 | EPI1102208 | EPI1101257 |  |  |  |  |  |  |  |
| EPI1100615 | EPI1100758 | EPI1104036 |  | EPI1101515 | EPI1102822 | EPI1100994 | EPI1102455 |  |  |  |  |  |  |  |
| EPI1100591 | EPI1102852 | EPI1100964 |  | EPI1102217 | EPI1102790 | EPI1102098 | EPI887990 |  |  |  |  |  |  |  |
| EPI1102501 | EPI1100750 | EPI1102012 |  | EPI1104043 | EPI1102814 | EPI1103210 | EPI887694 |  |  |  |  |  |  |  |
| EPI1100559 | EPI1102900 | EPI1101780 |  | EPI1104027 | EPI1102806 | EPI1102600 | EPI1101241 |  |  |  |  |  |  |  |
| EPI1101791 | EPI1100726 | EPI1100772 |  | EPI1104051 | EPI1102838 | EPI1102090 | EPI887790 |  |  |  |  |  |  |  |
| EPI1100631 | EPI1100742 | EPI1101988 |  | EPI1100963 | EPI1102830 | EPI1102256 | EPI887614 |  |  |  |  |  |  |  |
| EPI1101815 | EPI1102892 | EPI1101732 |  | EPI1104035 | EPI1102758 | EPI1103914 | EPI1102239 |  |  |  |  |  |  |  |
| EPI1100607 | EPI1102884 | EPI1101692 |  | EPI1101795 | EPI1102854 | EPI1103072 | EPI1101281 |  |  |  |  |  |  |  |
| EPI1100639 | EPI971347 | EPI1101700 |  | EPI1104083 | EPI1103340 | EPI1102480 | EPI1101233 |  |  |  |  |  |  |  |
| EPI1100679 | EPI971363 | EPI1101964 |  | EPI1101763 | EPI887613 | EPI1101154 | EPI1101217 |  |  |  |  |  |  |  |
| EPI1101847 | EPI971339 | EPI1102004 |  | EPI1101747 | EPI1101152 | EPI1102376 | EPI1102231 |  |  |  |  |  |  |  |
| EPI1100623 | EPI971443 | EPI1100732 |  | EPI1101739 | EPI1104152 | EPI1101178 | EPI1101225 |  |  |  |  |  |  |  |
| EPI1101823 | EPI971355 | EPI1101980 |  | EPI1101707 | EPI1103944 | EPI712921 | EPI1103023 |  |  |  |  |  |  |  |
| EPI1101039 | EPI971371 |  |  | EPI1101755 | EPI1101504 | EPI887671 | EPI1101209 |  |  |  |  |  |  |  |
| EPI1101055 | EPI971259 |  |  | EPI1104067 | EPI1102630 | EPI887807 | EPI1101849 |  |  |  |  |  |  |  |
| EPI1101071 | EPI971475 |  |  | EPI1104075 | EPI1103030 | EPI1103096 | EPI1101897 |  |  |  |  |  |  |  |
| EPI1101079 | EPI971299 |  |  | EPI1101779 | EPI1102454 | EPI1102066 | EPI1160161 |  |  |  |  |  |  |  |
| EPI1101087 | EPI971483 |  |  | EPI1100771 | EPI1101280 | EPI1102074 | EPI1100657 |  |  |  |  |  |  |  |
| EPI1101047 | EPI1101806 |  |  | EPI1101731 | EPI1103864 | EPI887759 | EPI1103873 |  |  |  |  |  |  |  |
| EPI1101063 | EPI1101974 |  |  | EPI1101963 | EPI887813 | EPI1103016 | EPI1101881 |  |  |  |  |  |  |  |
| EPI1101527 | EPI1102252 |  |  | EPI1101691 | EPI1101248 | EPI1103064 | EPI1102871 |  |  |  |  |  |  |  |
| EPI1102669 | EPI1104006 |  |  | EPI1101699 | EPI887989 | EPI1102984 | EPI1100665 |  |  |  |  |  |  |  |
| EPI1102653 | EPI1103998 |  |  | EPI1100731 | EPI1104112 | EPI1103008 | EPI1101833 |  |  |  |  |  |  |  |
| EPI1100895 | EPI1036699 |  |  | EPI1101979 | EPI1104128 | EPI1102384 | EPI1100561 |  |  |  |  |  |  |  |
| EPI1102661 | EPI1103958 |  |  | EPI1102003 | EPI887717 | EPI979560 | EPI1010119 |  |  |  |  |  |  |  |
| EPI1101351 | EPI707944 |  |  | EPI1101987 | EPI887637 | EPI1100802 | EPI1100897 |  |  |  |  |  |  |  |
| EPI1101335 | EPI1090735 |  |  | EPI1102011 | EPI971149 | EPI1103080 | EPI1100593 |  |  |  |  |  |  |  |
| EPI1101359 | EPI683027 |  |  |  | EPI1102080 | EPI1154776 | EPI1101905 |  |  |  |  |  |  |  |
| EPI1101327 | EPI683028 |  |  |  | EPI1103305 | EPI971183 | EPI1100873 |  |  |  |  |  |  |  |
| EPI1101319 | EPI729461 |  |  |  | EPI887917 | EPI1102640 | EPI1101857 |  |  |  |  |  |  |  |
| EPI1101343 | EPI521919 |  |  |  | EPI887789 | EPI945066 | EPI1100681 |  |  |  |  |  |  |  |
| EPI1103339 | EPI729453 |  |  |  | EPI1102998 | EPI1102107 | EPI1101865 |  |  |  |  |  |  |  |
| EPI887684 | EPI628599 |  |  |  | EPI971141 | EPI971095 | EPI1100881 |  |  |  |  |  |  |  |
| EPI979637 | EPI1090231 |  |  |  | EPI887965 | EPI1101458 | EPI1102279 |  |  |  |  |  |  |  |
| EPI1102138 | EPI627368 |  |  |  | EPI979526 | EPI979552 | EPI1100569 |  |  |  |  |  |  |  |
| EPI1103355 | EPI627360 |  |  |  | EPI887709 | EPI979544 | EPI1101873 |  |  |  |  |  |  |  |
| EPI1103911 | EPI560400 |  |  |  | EPI1103332 | EPI979616 | EPI1100689 |  |  |  |  |  |  |  |
| EPI971236 | EPI769641 |  |  |  | EPI1102974 | EPI985836 | EPI1102263 |  |  |  |  |  |  |  |
| EPI971244 | EPI1090215 |  |  |  | EPI979534 | EPI1103104 | EPI1102919 |  |  |  |  |  |  |  |
| EPI1102509 | EPI769585 |  |  |  | EPI1103928 | EPI1103048 | EPI1102527 |  |  |  |  |  |  |  |
| EPI1102525 | EPI1090223 |  |  |  | EPI1102622 | EPI1102200 | EPI1101889 |  |  |  |  |  |  |  |
| EPI971508 | EPI707971 |  |  |  | EPI1103316 | EPI887623 | EPI1100489 |  |  |  |  |  |  |  |
| EPI1103374 | EPI707956 |  |  |  | EPI1054119 | EPI1054145 | EPI1100577 |  |  |  |  |  |  |  |
| EPI1103331 | EPI592283 |  |  |  | EPI887741 | EPI1103088 |  |  |  |  |  |  |  |  |
| EPI1103315 | EPI627440 |  |  |  | EPI887693 | EPI1101714 |  |  |  |  |  |  |  |  |
| EPI1101119 | EPI566043 |  |  |  | EPI1103022 | EPI1102992 |  |  |  |  |  |  |  |  |
| EPI1010115 | EPI627336 |  |  |  | EPI1103196 | EPI1100970 |  |  |  |  |  |  |  |  |
| EPI887908 | EPI627480 |  |  |  | EPI921785 | EPI1102488 |  |  |  |  |  |  |  |  |
| EPI1063144 | EPI627496 |  |  |  | EPI1103824 | EPI887687 |  |  |  |  |  |  |  |  |
| EPI1103029 | EPI627488 |  |  |  | EPI1101264 | EPI887655 |  |  |  |  |  |  |  |  |
| EPI1101255 | EPI627280 |  |  |  | EPI979582 | EPI1102424 |  |  |  |  |  |  |  |  |
| EPI1100887 | EPI1090247 |  |  |  | EPI1101112 | EPI1054097 |  |  |  |  |  |  |  |  |
| EPI1103304 | EPI592276 |  |  |  | EPI1102606 | EPI971495 |  |  |  |  |  |  |  |  |
| EPI887700 | EPI566099 |  |  |  | EPI1102614 | EPI971079 |  |  |  |  |  |  |  |  |
| EPI1102717 | EPI627831 |  |  |  | EPI1101888 | EPI1102416 |  |  |  |  |  |  |  |  |
| EPI1063157 | EPI627911 |  |  |  | EPI1010116 | EPI887647 |  |  |  |  |  |  |  |  |
| EPI1103206 | EPI627919 |  |  |  | EPI1102526 | EPI971111 |  |  |  |  |  |  |  |  |
| EPI1101895 | EPI566107 |  |  |  | EPI1100896 | EPI926860 |  |  |  |  |  |  |  |  |
| EPI1103951 | EPI566067 |  |  |  | EPI1101832 | EPI1102448 |  |  |  |  |  |  |  |  |
| EPI1101511 | EPI627320 |  |  |  | EPI1100688 | EPI1054105 |  |  |  |  |  |  |  |  |
| EPI1100695 | EPI1090599 |  |  |  | EPI1101896 | EPI1102936 |  |  |  |  |  |  |  |  |
| EPI1102127 | EPI566051 |  |  |  | EPI1103872 | EPI1103056 |  |  |  |  |  |  |  |  |
| EPI1104103 | EPI627328 |  |  |  | EPI1160158 | EPI971487 |  |  |  |  |  |  |  |  |
| EPI1101183 | EPI566115 |  |  |  | EPI1100664 | EPI1103040 |  |  |  |  |  |  |  |  |
| EPI1101175 | EPI627504 |  |  |  | EPI1101904 | EPI926852 |  |  |  |  |  |  |  |  |
| EPI1100551 | EPI627344 |  |  |  | EPI1101848 | EPI1101722 |  |  |  |  |  |  |  |  |
| EPI1102605 | EPI1090607 |  |  |  | EPI1100560 | EPI1102720 |  |  |  |  |  |  |  |  |
| EPI971228 | EPI566059 |  |  |  | EPI1100568 | EPI887935 |  |  |  |  |  |  |  |  |
| EPI971268 | EPI627352 |  |  |  | EPI1100592 | EPI1103858 |  |  |  |  |  |  |  |  |
| EPI1101191 | EPI593169 |  |  |  | EPI1101872 | EPI922226 |  |  |  |  |  |  |  |  |
| EPI1104095 | EPI593176 |  |  |  | EPI1101880 | EPI1103842 |  |  |  |  |  |  |  |  |
| EPI1100583 | EPI1057919 |  |  |  | EPI1102870 | EPI912959 |  |  |  |  |  |  |  |  |
| EPI887796 | EPI1090199 |  |  |  | EPI1102262 | EPI887927 |  |  |  |  |  |  |  |  |
| EPI1102613 | EPI628703 |  |  |  | EPI1100488 | EPI1103254 |  |  |  |  |  |  |  |  |
| EPI1102621 | EPI628719 |  |  |  | EPI1100872 | EPI887895 |  |  |  |  |  |  |  |  |
| EPI1104151 | EPI592510 |  |  |  | EPI1100680 | EPI912951 |  |  |  |  |  |  |  |  |
| EPI1102981 | EPI592594 |  |  |  | EPI1100880 | EPI921612 |  |  |  |  |  |  |  |  |
| EPI887948 | EPI593383 |  |  |  | EPI1102918 | EPI1103818 |  |  |  |  |  |  |  |  |
| EPI1100671 | EPI592462 |  |  |  | EPI1100656 | EPI921893 |  |  |  |  |  |  |  |  |
| EPI1101911 | EPI592517 |  |  |  | EPI1100576 | EPI1103826 |  |  |  |  |  |  |  |  |
| EPI1101279 | EPI592486 |  |  |  | EPI1102278 | EPI1102712 |  |  |  |  |  |  |  |  |
| EPI887756 | EPI592246 |  |  |  | EPI1101856 | EPI1103866 |  |  |  |  |  |  |  |  |
| EPI1101887 | EPI592579 |  |  |  | EPI1101864 | EPI887735 |  |  |  |  |  |  |  |  |
| EPI1102877 | EPI707964 |  |  |  | EPI1101272 | EPI1103243 |  |  |  |  |  |  |  |  |
| EPI887620 | EPI707960 |  |  |  | EPI1100760 | EPI1102368 |  |  |  |  |  |  |  |  |
| EPI1102677 | EPI707957 |  |  |  | EPI1103271 | EPI1102352 |  |  |  |  |  |  |  |  |
| EPI1100687 | EPI707959 |  |  |  | EPI922064 | EPI1102288 |  |  |  |  |  |  |  |  |
| EPI1101967 | EPI707961 |  |  |  | EPI1103832 | EPI979624 |  |  |  |  |  |  |  |  |
| EPI1104047 | EPI707962 |  |  |  | EPI1102117 | EPI1101690 |  |  |  |  |  |  |  |  |
| EPI1104039 | EPI707963 |  |  |  | EPI1101184 | EPI1101698 |  |  |  |  |  |  |  |  |
| EPI1101735 | EPI593543 |  |  |  | EPI887765 | EPI1104034 |  |  |  |  |  |  |  |  |
| EPI1101799 | EPI703996 |  |  |  | EPI887909 | EPI1104026 |  |  |  |  |  |  |  |  |
| EPI1104087 | EPI628759 |  |  |  | EPI1102286 | EPI1104042 |  |  |  |  |  |  |  |  |
| EPI1101991 | EPI580373 |  |  |  | EPI971261 | EPI1104082 |  |  |  |  |  |  |  |  |
| EPI1100967 | EPI1090287 |  |  |  | EPI1103283 | EPI1101962 |  |  |  |  |  |  |  |  |
| EPI1104031 | EPI707973 |  |  |  | EPI971221 | EPI1101738 |  |  |  |  |  |  |  |  |
| EPI1102007 | EPI707976 |  |  |  | EPI1101160 | EPI1101746 |  |  |  |  |  |  |  |  |
| EPI1101983 | EPI692619 |  |  |  | EPI1103324 | EPI1101754 |  |  |  |  |  |  |  |  |
| EPI1102565 | EPI1102308 |  |  |  | EPI1103294 | EPI1101706 |  |  |  |  |  |  |  |  |
| EPI1102573 | EPI707972 |  |  |  | EPI1101224 | EPI1101762 |  |  |  |  |  |  |  |  |
| EPI1100775 | EPI626984 |  |  |  | EPI1101216 | EPI1102224 |  |  |  |  |  |  |  |  |
| EPI1036690 | EPI1103894 |  |  |  | EPI1101240 | EPI1104066 |  |  |  |  |  |  |  |  |
| EPI967143 | EPI1090751 |  |  |  | EPI1101256 | EPI1104074 |  |  |  |  |  |  |  |  |
| EPI1100735 | EPI971275 |  |  |  | EPI1101208 | EPI1101994 |  |  |  |  |  |  |  |  |
| EPI1101519 | EPI1103404 |  |  |  | EPI1101232 | EPI1101978 |  |  |  |  |  |  |  |  |
| EPI1102229 | EPI921814 |  |  |  | EPI887749 | EPI1102002 |  |  |  |  |  |  |  |  |
| EPI1102221 | EPI1103830 |  |  |  | EPI979518 | EPI1104050 |  |  |  |  |  |  |  |  |
| EPI1104015 | EPI887939 |  |  |  | EPI1101728 | EPI1101730 |  |  |  |  |  |  |  |  |
| EPI1101783 | EPI1103862 |  |  |  | EPI1100728 | EPI1104154 |  |  |  |  |  |  |  |  |
| EPI969331 | EPI922210 |  |  |  | EPI1104072 | EPI1104090 |  |  |  |  |  |  |  |  |
| EPI1101999 | EPI1103846 |  |  |  | EPI1101512 | EPI1100770 |  |  |  |  |  |  |  |  |
| EPI1101703 | EPI887931 |  |  |  | EPI1102566 | EPI1104130 |  |  |  |  |  |  |  |  |
| EPI1101695 | EPI1103822 |  |  |  | EPI971133 | EPI1102560 |  |  |  |  |  |  |  |  |
| EPI1101863 | EPI912950 |  |  |  | EPI1104120 | EPI1104114 |  |  |  |  |  |  |  |  |
| EPI1102629 | EPI1103259 |  |  |  | EPI1101688 | EPI971135 |  |  |  |  |  |  |  |  |
| EPI1102461 | EPI921596 |  |  |  | EPI1101696 | EPI971151 |  |  |  |  |  |  |  |  |
| EPI887724 | EPI1103870 |  |  |  | EPI1100768 | EPI1101778 |  |  |  |  |  |  |  |  |
| EPI1101239 | EPI887899 |  |  |  | EPI1100960 | EPI971143 |  |  |  |  |  |  |  |  |
| EPI1100599 | EPI912958 |  |  |  | EPI1102214 | EPI1104146 |  |  |  |  |  |  |  |  |
| EPI1102285 | EPI887859 |  |  |  | EPI1104008 | EPI1104138 |  |  |  |  |  |  |  |  |
| EPI1104119 | EPI887875 |  |  |  | EPI971285 | EPI1101514 |  |  |  |  |  |  |  |  |
| EPI1101903 | EPI1103854 |  |  |  | EPI1102558 | EPI1104098 |  |  |  |  |  |  |  |  |
| EPI1103879 | EPI1142575 |  |  |  | EPI1102222 | EPI1102216 |  |  |  |  |  |  |  |  |
| EPI1160157 | EPI887827 |  |  |  | EPI1104064 | EPI1100730 |  |  |  |  |  |  |  |  |
| EPI1102693 | EPI887803 |  |  |  | EPI1104104 | EPI971287 |  |  |  |  |  |  |  |  |
| EPI1102701 | EPI979612 |  |  |  | EPI1104136 | EPI1100962 |  |  |  |  |  |  |  |  |
| EPI1102805 | EPI1103004 |  |  |  | EPI1104088 | EPI1104106 |  |  |  |  |  |  |  |  |
| EPI1102757 | EPI1103076 |  |  |  | EPI1104144 | EPI1104122 |  |  |  |  |  |  |  |  |
| EPI971124 | EPI887947 |  |  |  | EPI1104096 | EPI1101794 |  |  |  |  |  |  |  |  |
| EPI1100767 | EPI1102300 |  |  |  | EPI1104032 | EPI1036692 |  |  |  |  |  |  |  |  |
| EPI1102749 | EPI1103248 |  |  |  | EPI1102000 | EPI1104010 |  |  |  |  |  |  |  |  |
| EPI971132 | EPI1102356 |  |  |  | EPI1101976 | EPI1102696 |  |  |  |  |  |  |  |  |
| EPI1102725 | EPI1102372 |  |  |  | EPI1102008 | EPI1090376 |  |  |  |  |  |  |  |  |
| EPI1102733 | EPI1102484 |  |  |  | EPI1101984 | EPI971127 |  |  |  |  |  |  |  |  |
| EPI972304 | EPI1102380 |  |  |  | EPI1101992 | EPI971119 |  |  |  |  |  |  |  |  |
| EPI1102685 | EPI887683 |  |  |  | EPI1101776 | EPI1102704 |  |  |  |  |  |  |  |  |
| EPI1102709 | EPI1101118 |  |  |  | EPI1104024 | EPI1102688 |  |  |  |  |  |  |  |  |
| EPI1102765 | EPI1101102 |  |  |  | EPI1101704 | EPI1101506 |  |  |  |  |  |  |  |  |
| EPI1102829 | EPI1101110 |  |  |  | EPI1104040 | EPI1100906 |  |  |  |  |  |  |  |  |
| EPI1102773 | EPI1102332 |  |  |  | EPI1104048 | EPI1102904 |  |  |  |  |  |  |  |  |
| EPI1102813 | EPI1101350 |  |  |  | EPI1104080 | EPI1102912 |  |  |  |  |  |  |  |  |
| EPI1102861 | EPI887779 |  |  |  | EPI1101744 | EPI1102328 |  |  |  |  |  |  |  |  |
| EPI1102789 | EPI971219 |  |  |  | EPI1101752 | EPI1103220 |  |  |  |  |  |  |  |  |
| EPI1102821 | EPI1102516 |  |  |  | EPI1101760 | EPI887775 |  |  |  |  |  |  |  |  |
| EPI1102781 | EPI1100558 |  |  |  | EPI1101736 | EPI971247 |  |  |  |  |  |  |  |  |
| EPI1102797 | EPI1102276 |  |  |  | EPI1101792 | EPI971167 |  |  |  |  |  |  |  |  |
| EPI1102845 | EPI971211 |  |  |  | EPI1101960 | EPI1103188 |  |  |  |  |  |  |  |  |
| EPI1102741 | EPI1103193 |  |  |  |  | EPI971175 |  |  |  |  |  |  |  |  |
| EPI1102837 | EPI1100678 |  |  |  |  | EPI1103264 |  |  |  |  |  |  |  |  |
| EPI979589 | EPI1101790 |  |  |  |  | EPI971199 |  |  |  |  |  |  |  |  |
| EPI1102293 | EPI1102660 |  |  |  |  | EPI971191 |  |  |  |  |  |  |  |  |
| EPI1101159 | EPI971203 |  |  |  |  | EPI1103922 |  |  |  |  |  |  |  |  |
| EPI1101167 | EPI1101526 |  |  |  |  | EPI1103802 |  |  |  |  |  |  |  |  |
| EPI1100879 | EPI1101334 |  |  |  |  | EPI1102512 |  |  |  |  |  |  |  |  |
| EPI1100495 | EPI1101326 |  |  |  |  | EPI971207 |  |  |  |  |  |  |  |  |
| EPI1102245 | EPI1101318 |  |  |  |  | EPI971311 |  |  |  |  |  |  |  |  |
| EPI1102237 | EPI1101342 |  |  |  |  | EPI887727 |  |  |  |  |  |  |  |  |
| EPI1104071 | EPI1101038 |  |  |  |  | EPI971215 |  |  |  |  |  |  |  |  |
| EPI1104079 | EPI971251 |  |  |  |  | EPI971159 |  |  |  |  |  |  |  |  |
| EPI1103935 | EPI971171 |  |  |  |  | EPI1102272 |  |  |  |  |  |  |  |  |
| EPI1100663 | EPI1100622 |  |  |  |  | EPI1100610 |  |  |  |  |  |  |  |  |
| EPI1101223 | EPI1100894 |  |  |  |  | EPI1102496 |  |  |  |  |  |  |  |  |
| EPI1054126 | EPI1103237 |  |  |  |  | EPI1100586 |  |  |  |  |  |  |  |  |
| EPI887924 | EPI1100590 |  |  |  |  | EPI1101826 |  |  |  |  |  |  |  |  |
| EPI1063146 | EPI1100614 |  |  |  |  | EPI1100890 |  |  |  |  |  |  |  |  |
| EPI1100575 | EPI1101830 |  |  |  |  | EPI1102648 |  |  |  |  |  |  |  |  |
| EPI1103037 | EPI1102500 |  |  |  |  | EPI1102656 |  |  |  |  |  |  |  |  |
| EPI1103005 | EPI1100638 |  |  |  |  | EPI1101522 |  |  |  |  |  |  |  |  |
| EPI971252 | EPI971163 |  |  |  |  | EPI1102664 |  |  |  |  |  |  |  |  |
| EPI1102533 | EPI1100910 |  |  |  |  | EPI1101034 |  |  |  |  |  |  |  |  |
| EPI1101751 | EPI1102908 |  |  |  |  | EPI1101050 |  |  |  |  |  |  |  |  |
| EPI1101759 | EPI1101054 |  |  |  |  | EPI1101074 |  |  |  |  |  |  |  |  |
| EPI1101743 | EPI1100606 |  |  |  |  | EPI1101066 |  |  |  |  |  |  |  |  |
| EPI1101711 | EPI971315 |  |  |  |  | EPI1101082 |  |  |  |  |  |  |  |  |
| EPI1101767 | EPI1103926 |  |  |  |  | EPI1101042 |  |  |  |  |  |  |  |  |
| EPI887644 | EPI1101070 |  |  |  |  | EPI1101058 |  |  |  |  |  |  |  |  |
| EPI1063163 | EPI1101086 |  |  |  |  | EPI1101346 |  |  |  |  |  |  |  |  |
| EPI1103293 | EPI1101822 |  |  |  |  | EPI1101330 |  |  |  |  |  |  |  |  |
| EPI887916 | EPI971179 |  |  |  |  | EPI1101354 |  |  |  |  |  |  |  |  |
| EPI1063145 | EPI1101062 |  |  |  |  | EPI1101322 |  |  |  |  |  |  |  |  |
| EPI1102087 | EPI887731 |  |  |  |  | EPI1101314 |  |  |  |  |  |  |  |  |
| EPI1101231 | EPI1102668 |  |  |  |  | EPI1101338 |  |  |  |  |  |  |  |  |
| EPI1101247 | EPI1101046 |  |  |  |  | EPI1100554 |  |  |  |  |  |  |  |  |
| EPI887820 | EPI1101814 |  |  |  |  | EPI1100634 |  |  |  |  |  |  |  |  |
| EPI1101287 | EPI1102916 |  |  |  |  | EPI1100626 |  |  |  |  |  |  |  |  |
| EPI971140 | EPI1103225 |  |  |  |  | EPI1100674 |  |  |  |  |  |  |  |  |
| EPI1104127 | EPI1100630 |  |  |  |  | EPI1101842 |  |  |  |  |  |  |  |  |
| EPI1101879 | EPI1103269 |  |  |  |  | EPI1100618 |  |  |  |  |  |  |  |  |
| EPI1104111 | EPI1101078 |  |  |  |  | EPI1101810 |  |  |  |  |  |  |  |  |
| EPI887996 | EPI1103806 |  |  |  |  | EPI1101786 |  |  |  |  |  |  |  |  |
| EPI1104159 | EPI1102652 |  |  |  |  | EPI1100602 |  |  |  |  |  |  |  |  |
| EPI1101839 | EPI1101846 |  |  |  |  | EPI1101818 |  |  |  |  |  |  |  |  |
| EPI1063162 | EPI1101358 |  |  |  |  | EPI1103946 |  |  |  |  |  |  |  |  |
| EPI1103282 | EPI971195 |  |  |  |  | EPI1054121 |  |  |  |  |  |  |  |  |
| EPI1100903 | EPI887867 |  |  |  |  | EPI1103318 |  |  |  |  |  |  |  |  |
| EPI1100567 | EPI1101150 |  |  |  |  | EPI1102232 |  |  |  |  |  |  |  |  |
| EPI887772 | EPI887883 |  |  |  |  | EPI1102240 |  |  |  |  |  |  |  |  |
| EPI1104143 | EPI1103363 |  |  |  |  | EPI887815 |  |  |  |  |  |  |  |  |
| EPI1102269 | EPI1102260 |  |  |  |  | EPI887903 |  |  |  |  |  |  |  |  |
| EPI1103323 | EPI979580 |  |  |  |  | EPI1102456 |  |  |  |  |  |  |  |  |
| EPI1103839 | EPI971507 |  |  |  |  | EPI1101122 |  |  |  |  |  |  |  |  |
| EPI887716 | EPI1103918 |  |  |  |  | EPI887615 |  |  |  |  |  |  |  |  |
| EPI887972 | EPI1103373 |  |  |  |  | EPI887767 |  |  |  |  |  |  |  |  |
| EPI979525 | EPI1102316 |  |  |  |  | EPI887967 |  |  |  |  |  |  |  |  |
| EPI971292 | EPI1102508 |  |  |  |  | EPI887991 |  |  |  |  |  |  |  |  |
| EPI979533 | EPI1102524 |  |  |  |  | EPI887943 |  |  |  |  |  |  |  |  |
| EPI1063151 | EPI1103910 |  |  |  |  | EPI979528 |  |  |  |  |  |  |  |  |
| EPI971156 | EPI971235 |  |  |  |  | EPI887951 |  |  |  |  |  |  |  |  |
| EPI1102637 | EPI971243 |  |  |  |  | EPI887719 |  |  |  |  |  |  |  |  |
| EPI922060 | EPI1013248 |  |  |  |  | EPI887919 |  |  |  |  |  |  |  |  |
| EPI979541 | EPI1102136 |  |  |  |  | EPI887711 |  |  |  |  |  |  |  |  |
| EPI1101295 | EPI1103354 |  |  |  |  | EPI1103274 |  |  |  |  |  |  |  |  |
| EPI887748 | EPI1018167 |  |  |  |  | EPI971263 |  |  |  |  |  |  |  |  |
| EPI1101215 | EPI1022664 |  |  |  |  | EPI1103000 |  |  |  |  |  |  |  |  |
| EPI971148 | EPI1101126 |  |  |  |  | EPI887695 |  |  |  |  |  |  |  |  |
| EPI1104135 | EPI887707 |  |  |  |  | EPI1103810 |  |  |  |  |  |  |  |  |
| EPI1104055 | EPI979628 |  |  |  |  | EPI1103326 |  |  |  |  |  |  |  |  |
| EPI1102925 | EPI979636 |  |  |  |  | EPI1102119 |  |  |  |  |  |  |  |  |
| EPI1101271 | EPI887955 |  |  |  |  | EPI887751 |  |  |  |  |  |  |  |  |
| EPI1101263 | EPI888067 |  |  |  |  | EPI887743 |  |  |  |  |  |  |  |  |
| EPI1101871 | EPI887963 |  |  |  |  | EPI888063 |  |  |  |  |  |  |  |  |
| EPI1101855 | EPI1101174 |  |  |  |  | EPI1103296 |  |  |  |  |  |  |  |  |
| EPI1101855 | EPI1101182 |  |  |  |  | EPI1102336 |  |  |  |  |  |  |  |  |
|  | EPI1102604 |  |  |  |  | EPI1101114 |  |  |  |  |  |  |  |  |
|  | EPI1100550 |  |  |  |  | EPI1102976 |  |  |  |  |  |  |  |  |
|  | EPI887787 |  |  |  |  | EPI1101186 |  |  |  |  |  |  |  |  |
|  | EPI1103215 |  |  |  |  | EPI1103032 |  |  |  |  |  |  |  |  |
|  | EPI1102149 |  |  |  |  | EPI1103286 |  |  |  |  |  |  |  |  |
|  | EPI1102102 |  |  |  |  | EPI1102296 |  |  |  |  |  |  |  |  |
|  | EPI1100534 |  |  |  |  | EPI887679 |  |  |  |  |  |  |  |  |
|  | EPI1102094 |  |  |  |  | EPI1102608 |  |  |  |  |  |  |  |  |
|  | EPI1101142 |  |  |  |  | EPI1102616 |  |  |  |  |  |  |  |  |
|  | EPI1100998 |  |  |  |  | EPI1103199 |  |  |  |  |  |  |  |  |
|  | EPI1102212 |  |  |  |  | EPI1102672 |  |  |  |  |  |  |  |  |
|  | EPI1102195 |  |  |  |  | EPI1101234 |  |  |  |  |  |  |  |  |
|  | EPI1100982 |  |  |  |  | EPI1101210 |  |  |  |  |  |  |  |  |
|  | EPI1100990 |  |  |  |  | EPI1101258 |  |  |  |  |  |  |  |  |
|  | EPI1102340 |  |  |  |  | EPI1101162 |  |  |  |  |  |  |  |  |
|  | EPI1103313 |  |  |  |  | EPI1102632 |  |  |  |  |  |  |  |  |
|  | EPI1101286 |  |  |  |  | EPI887639 |  |  |  |  |  |  |  |  |
|  | EPI1102284 |  |  |  |  | EPI1102082 |  |  |  |  |  |  |  |  |
|  | EPI1101214 |  |  |  |  | EPI1103024 |  |  |  |  |  |  |  |  |
|  | EPI1101238 |  |  |  |  | EPI922066 |  |  |  |  |  |  |  |  |
|  | EPI1101854 |  |  |  |  | EPI887911 |  |  |  |  |  |  |  |  |
|  | EPI887619 |  |  |  |  | EPI1103834 |  |  |  |  |  |  |  |  |
|  | EPI1103279 |  |  |  |  | EPI979536 |  |  |  |  |  |  |  |  |
|  | EPI1102924 |  |  |  |  | EPI887791 |  |  |  |  |  |  |  |  |
|  | EPI1101510 |  |  |  |  | EPI971223 |  |  |  |  |  |  |  |  |
|  | EPI1104158 |  |  |  |  | EPI979520 |  |  |  |  |  |  |  |  |
|  | EPI1101886 |  |  |  |  | EPI1103930 |  |  |  |  |  |  |  |  |
|  | EPI1102876 |  |  |  |  | EPI1102760 |  |  |  |  |  |  |  |  |
|  | EPI1010117 |  |  |  |  | EPI1102808 |  |  |  |  |  |  |  |  |
|  | EPI1104102 |  |  |  |  | EPI1102776 |  |  |  |  |  |  |  |  |
|  | EPI887995 |  |  |  |  | EPI1102736 |  |  |  |  |  |  |  |  |
|  | EPI1101910 |  |  |  |  | EPI1102792 |  |  |  |  |  |  |  |  |
|  | EPI1100670 |  |  |  |  | EPI1102768 |  |  |  |  |  |  |  |  |
|  | EPI1102980 |  |  |  |  | EPI1102784 |  |  |  |  |  |  |  |  |
|  | EPI971267 |  |  |  |  | EPI1102816 |  |  |  |  |  |  |  |  |
|  | EPI887771 |  |  |  |  | EPI1102832 |  |  |  |  |  |  |  |  |
|  | EPI1102292 |  |  |  |  | EPI1102824 |  |  |  |  |  |  |  |  |
|  | EPI1103204 |  |  |  |  | EPI1102840 |  |  |  |  |  |  |  |  |
|  | EPI887723 |  |  |  |  |  |  |  |  |  |  |  |  |  |
|  | EPI1102086 |  |  |  |  |  |  |  |  |  |  |  |  |  |
|  | EPI1101270 |  |  |  |  |  |  |  |  |  |  |  |  |  |
|  | EPI979588 |  |  |  |  |  |  |  |  |  |  |  |  |  |
|  | EPI1102628 |  |  |  |  |  |  |  |  |  |  |  |  |  |
|  | EPI1101254 |  |  |  |  |  |  |  |  |  |  |  |  |  |
|  | EPI1101278 |  |  |  |  |  |  |  |  |  |  |  |  |  |
|  | EPI1104094 |  |  |  |  |  |  |  |  |  |  |  |  |  |
|  | EPI1104134 |  |  |  |  |  |  |  |  |  |  |  |  |  |
|  | EPI1104118 |  |  |  |  |  |  |  |  |  |  |  |  |  |
|  | EPI1101902 |  |  |  |  |  |  |  |  |  |  |  |  |  |
|  | EPI1103878 |  |  |  |  |  |  |  |  |  |  |  |  |  |
|  | EPI1160159 |  |  |  |  |  |  |  |  |  |  |  |  |  |
|  | EPI1103036 |  |  |  |  |  |  |  |  |  |  |  |  |  |
|  | EPI971139 |  |  |  |  |  |  |  |  |  |  |  |  |  |
|  | EPI1104126 |  |  |  |  |  |  |  |  |  |  |  |  |  |
|  | EPI1101190 |  |  |  |  |  |  |  |  |  |  |  |  |  |
|  | EPI1103330 |  |  |  |  |  |  |  |  |  |  |  |  |  |
|  | EPI1101166 |  |  |  |  |  |  |  |  |  |  |  |  |  |
|  | EPI1103291 |  |  |  |  |  |  |  |  |  |  |  |  |  |
|  | EPI887699 |  |  |  |  |  |  |  |  |  |  |  |  |  |
|  | EPI1103322 |  |  |  |  |  |  |  |  |  |  |  |  |  |
|  | EPI1100878 |  |  |  |  |  |  |  |  |  |  |  |  |  |
|  | EPI1100582 |  |  |  |  |  |  |  |  |  |  |  |  |  |
|  | EPI1100494 |  |  |  |  |  |  |  |  |  |  |  |  |  |
|  | EPI1100694 |  |  |  |  |  |  |  |  |  |  |  |  |  |
|  | EPI1104110 |  |  |  |  |  |  |  |  |  |  |  |  |  |
|  | EPI1104142 |  |  |  |  |  |  |  |  |  |  |  |  |  |
|  | EPI1102236 |  |  |  |  |  |  |  |  |  |  |  |  |  |
|  | EPI1102244 |  |  |  |  |  |  |  |  |  |  |  |  |  |
|  | EPI1104070 |  |  |  |  |  |  |  |  |  |  |  |  |  |
|  | EPI1104078 |  |  |  |  |  |  |  |  |  |  |  |  |  |
|  | EPI979540 |  |  |  |  |  |  |  |  |  |  |  |  |  |
|  | EPI1102532 |  |  |  |  |  |  |  |  |  |  |  |  |  |
|  | EPI1054125 |  |  |  |  |  |  |  |  |  |  |  |  |  |
|  | EPI1102756 |  |  |  |  |  |  |  |  |  |  |  |  |  |
|  | EPI1102692 |  |  |  |  |  |  |  |  |  |  |  |  |  |
|  | EPI1102700 |  |  |  |  |  |  |  |  |  |  |  |  |  |
|  | EPI1102716 |  |  |  |  |  |  |  |  |  |  |  |  |  |
|  | EPI1102748 |  |  |  |  |  |  |  |  |  |  |  |  |  |
|  | EPI971123 |  |  |  |  |  |  |  |  |  |  |  |  |  |
|  | EPI1100766 |  |  |  |  |  |  |  |  |  |  |  |  |  |
|  | EPI1102724 |  |  |  |  |  |  |  |  |  |  |  |  |  |
|  | EPI971131 |  |  |  |  |  |  |  |  |  |  |  |  |  |
|  | EPI1102732 |  |  |  |  |  |  |  |  |  |  |  |  |  |
|  | EPI1102684 |  |  |  |  |  |  |  |  |  |  |  |  |  |
|  | EPI972305 |  |  |  |  |  |  |  |  |  |  |  |  |  |
|  | EPI1102804 |  |  |  |  |  |  |  |  |  |  |  |  |  |
|  | EPI1102708 |  |  |  |  |  |  |  |  |  |  |  |  |  |
|  | EPI1102780 |  |  |  |  |  |  |  |  |  |  |  |  |  |
|  | EPI1102860 |  |  |  |  |  |  |  |  |  |  |  |  |  |
|  | EPI1102772 |  |  |  |  |  |  |  |  |  |  |  |  |  |
|  | EPI1102828 |  |  |  |  |  |  |  |  |  |  |  |  |  |
|  | EPI1102836 |  |  |  |  |  |  |  |  |  |  |  |  |  |
|  | EPI1102844 |  |  |  |  |  |  |  |  |  |  |  |  |  |
|  | EPI1102764 |  |  |  |  |  |  |  |  |  |  |  |  |  |
|  | EPI1102812 |  |  |  |  |  |  |  |  |  |  |  |  |  |
|  | EPI1102796 |  |  |  |  |  |  |  |  |  |  |  |  |  |
|  | EPI1102820 |  |  |  |  |  |  |  |  |  |  |  |  |  |
|  | EPI1102740 |  |  |  |  |  |  |  |  |  |  |  |  |  |
|  | EPI1102788 |  |  |  |  |  |  |  |  |  |  |  |  |  |
|  | EPI887795 |  |  |  |  |  |  |  |  |  |  |  |  |  |
|  | EPI887715 |  |  |  |  |  |  |  |  |  |  |  |  |  |
|  | EPI1102125 |  |  |  |  |  |  |  |  |  |  |  |  |  |
|  | EPI1103028 |  |  |  |  |  |  |  |  |  |  |  |  |  |
|  | EPI887747 |  |  |  |  |  |  |  |  |  |  |  |  |  |
|  | EPI1103814 |  |  |  |  |  |  |  |  |  |  |  |  |  |
|  | EPI971227 |  |  |  |  |  |  |  |  |  |  |  |  |  |
|  | EPI1100662 |  |  |  |  |  |  |  |  |  |  |  |  |  |
|  | EPI1101262 |  |  |  |  |  |  |  |  |  |  |  |  |  |
|  | EPI1102006 |  |  |  |  |  |  |  |  |  |  |  |  |  |
|  | EPI1102228 |  |  |  |  |  |  |  |  |  |  |  |  |  |
|  | EPI969330 |  |  |  |  |  |  |  |  |  |  |  |  |  |
|  | EPI1102014 |  |  |  |  |  |  |  |  |  |  |  |  |  |
|  | EPI1102564 |  |  |  |  |  |  |  |  |  |  |  |  |  |
|  | EPI1102572 |  |  |  |  |  |  |  |  |  |  |  |  |  |
|  | EPI1104046 |  |  |  |  |  |  |  |  |  |  |  |  |  |
|  | EPI1101998 |  |  |  |  |  |  |  |  |  |  |  |  |  |
|  | EPI1104030 |  |  |  |  |  |  |  |  |  |  |  |  |  |
|  | EPI1101734 |  |  |  |  |  |  |  |  |  |  |  |  |  |
|  | EPI1102220 |  |  |  |  |  |  |  |  |  |  |  |  |  |
|  | EPI1104014 |  |  |  |  |  |  |  |  |  |  |  |  |  |
|  | EPI1101710 |  |  |  |  |  |  |  |  |  |  |  |  |  |
|  | EPI1101766 |  |  |  |  |  |  |  |  |  |  |  |  |  |
|  | EPI1101758 |  |  |  |  |  |  |  |  |  |  |  |  |  |
|  | EPI1101750 |  |  |  |  |  |  |  |  |  |  |  |  |  |
|  | EPI1101742 |  |  |  |  |  |  |  |  |  |  |  |  |  |
|  | EPI1104054 |  |  |  |  |  |  |  |  |  |  |  |  |  |
|  | EPI1101982 |  |  |  |  |  |  |  |  |  |  |  |  |  |
|  | EPI1036691 |  |  |  |  |  |  |  |  |  |  |  |  |  |
|  | EPI1101782 |  |  |  |  |  |  |  |  |  |  |  |  |  |
|  | EPI1101798 |  |  |  |  |  |  |  |  |  |  |  |  |  |
|  | EPI1100966 |  |  |  |  |  |  |  |  |  |  |  |  |  |
|  | EPI1104038 |  |  |  |  |  |  |  |  |  |  |  |  |  |
|  | EPI1100734 |  |  |  |  |  |  |  |  |  |  |  |  |  |
|  | EPI1100774 |  |  |  |  |  |  |  |  |  |  |  |  |  |
|  | EPI1101966 |  |  |  |  |  |  |  |  |  |  |  |  |  |
|  | EPI1101518 |  |  |  |  |  |  |  |  |  |  |  |  |  |
|  | EPI1101694 |  |  |  |  |  |  |  |  |  |  |  |  |  |
|  | EPI1104086 |  |  |  |  |  |  |  |  |  |  |  |  |  |
|  | EPI1101702 |  |  |  |  |  |  |  |  |  |  |  |  |  |
|  | EPI967144 |  |  |  |  |  |  |  |  |  |  |  |  |  |
|  | EPI1101990 |  |  |  |  |  |  |  |  |  |  |  |  |  |
|  | EPI1100886 |  |  |  |  |  |  |  |  |  |  |  |  |  |
|  | EPI979532 |  |  |  |  |  |  |  |  |  |  |  |  |  |
|  | EPI1103338 |  |  |  |  |  |  |  |  |  |  |  |  |  |
|  | EPI1102612 |  |  |  |  |  |  |  |  |  |  |  |  |  |
|  | EPI1102620 |  |  |  |  |  |  |  |  |  |  |  |  |  |
|  | EPI1101246 |  |  |  |  |  |  |  |  |  |  |  |  |  |
|  | EPI1101222 |  |  |  |  |  |  |  |  |  |  |  |  |  |
|  | EPI1101294 |  |  |  |  |  |  |  |  |  |  |  |  |  |
|  | EPI887755 |  |  |  |  |  |  |  |  |  |  |  |  |  |
|  | EPI1101894 |  |  |  |  |  |  |  |  |  |  |  |  |  |
|  | EPI887643 |  |  |  |  |  |  |  |  |  |  |  |  |  |
|  | EPI887891 |  |  |  |  |  |  |  |  |  |  |  |  |  |
|  | EPI1100574 |  |  |  |  |  |  |  |  |  |  |  |  |  |
|  | EPI1103934 |  |  |  |  |  |  |  |  |  |  |  |  |  |
|  | EPI1101862 |  |  |  |  |  |  |  |  |  |  |  |  |  |
|  | EPI1103301 |  |  |  |  |  |  |  |  |  |  |  |  |  |
|  | EPI1103346 |  |  |  |  |  |  |  |  |  |  |  |  |  |
|  | EPI1104150 |  |  |  |  |  |  |  |  |  |  |  |  |  |
|  | EPI1102268 |  |  |  |  |  |  |  |  |  |  |  |  |  |
|  | EPI887971 |  |  |  |  |  |  |  |  |  |  |  |  |  |
|  | EPI887923 |  |  |  |  |  |  |  |  |  |  |  |  |  |
|  | EPI1100566 |  |  |  |  |  |  |  |  |  |  |  |  |  |
|  | EPI1101838 |  |  |  |  |  |  |  |  |  |  |  |  |  |
|  | EPI971147 |  |  |  |  |  |  |  |  |  |  |  |  |  |
|  | EPI1102636 |  |  |  |  |  |  |  |  |  |  |  |  |  |
|  | EPI1100598 |  |  |  |  |  |  |  |  |  |  |  |  |  |
|  | EPI887907 |  |  |  |  |  |  |  |  |  |  |  |  |  |
|  | EPI971155 |  |  |  |  |  |  |  |  |  |  |  |  |  |
|  | EPI1101870 |  |  |  |  |  |  |  |  |  |  |  |  |  |
|  | EPI1103950 |  |  |  |  |  |  |  |  |  |  |  |  |  |
|  | EPI1102460 |  |  |  |  |  |  |  |  |  |  |  |  |  |
|  | EPI1101158 |  |  |  |  |  |  |  |  |  |  |  |  |  |
|  | EPI1101230 |  |  |  |  |  |  |  |  |  |  |  |  |  |
|  | EPI922065 |  |  |  |  |  |  |  |  |  |  |  |  |  |
|  | EPI887915 |  |  |  |  |  |  |  |  |  |  |  |  |  |
|  | EPI1100902 |  |  |  |  |  |  |  |  |  |  |  |  |  |
|  | EPI971291 |  |  |  |  |  |  |  |  |  |  |  |  |  |
|  | EPI887819 |  |  |  |  |  |  |  |  |  |  |  |  |  |
|  | EPI1100686 |  |  |  |  |  |  |  |  |  |  |  |  |  |
|  | EPI1101878 |  |  |  |  |  |  |  |  |  |  |  |  |  |
|  | EPI1102676 |  |  |  |  |  |  |  |  |  |  |  |  |  |
|  | EPI979524 |  |  |  |  |  |  |  |  |  |  |  |  |  |
|  | EPI1103838 |  |  |  |  |  |  |  |  |  |  |  |  |  |
