# Supplementary figures and images for: Exploring utility of genomic epidemiology to trace origins of highly pathogenic influenza A/H7N9 in Guangdong
Source: Virus Evol. 2020 Dec 18;6(2):veaa097. doi: 10.1093/ve/veaa097 (PMC7758296; doi:10.1093/ve/veaa097)

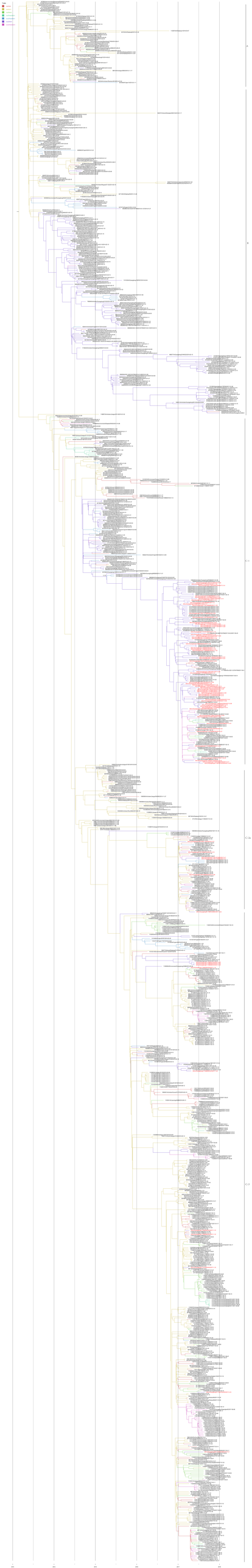

Supplement: veaa097_Supplementary_Data [file veaa097_supplementary_data.zip › S Fig 3 MCC.tre.pdf]

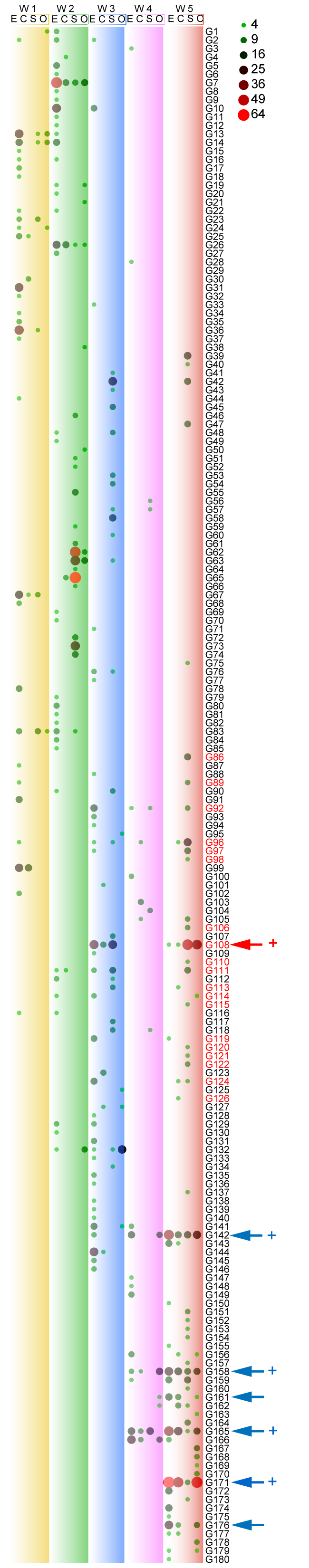

Supplement: veaa097_Supplementary_Data [file veaa097_supplementary_data.zip › S Figure 2.tif]
