# Supplementary material for: Exploring utility of genomic epidemiology to trace origins of highly pathogenic influenza A/H7N9 in Guangdong
Source: Virus Evol. 2020 Dec 18;6(2):veaa097. doi: 10.1093/ve/veaa097 (PMC7758296; doi:10.1093/ve/veaa097)
Supplement: veaa097_Supplementary_Data [file S figure 1.pdf › S figure 1.pdf]

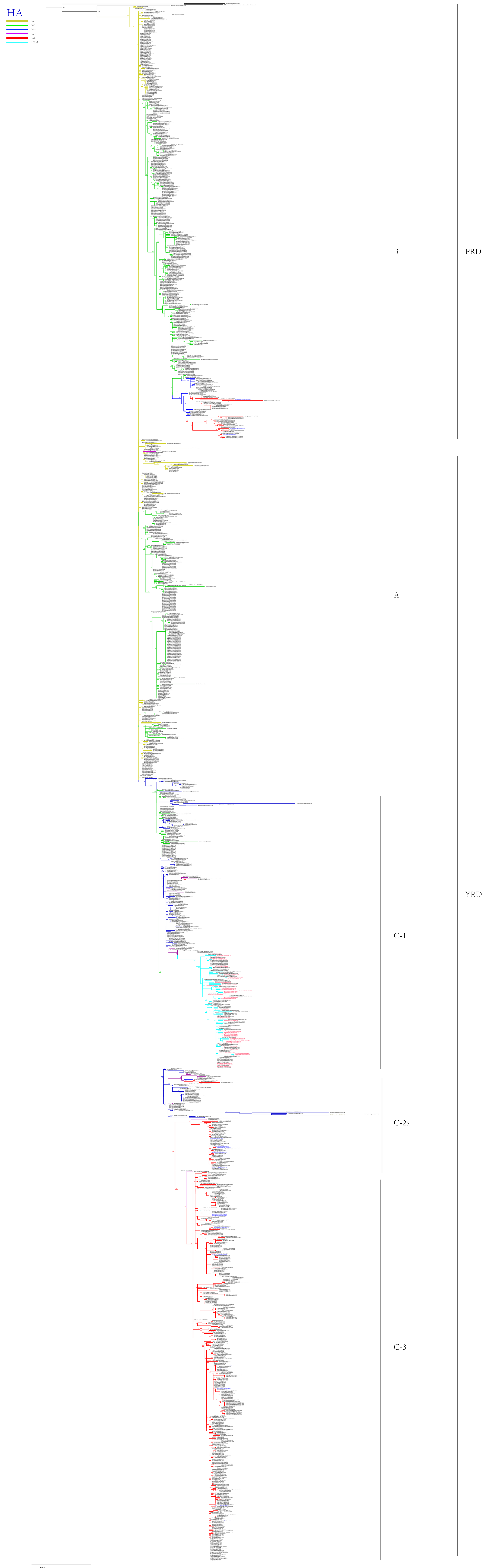
HA
W1
W2
W3
W4
W5
HPAI
B
PRD
A
YRD
C-1
C-2a
C-3
0.03

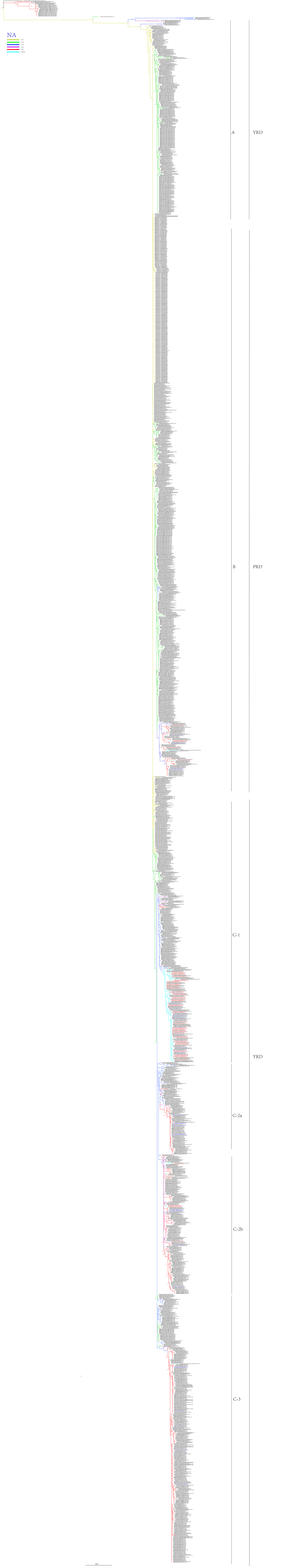
NA
A
YRD
B
PRD
C-1
YRD
C-2a
C-2b
C-3

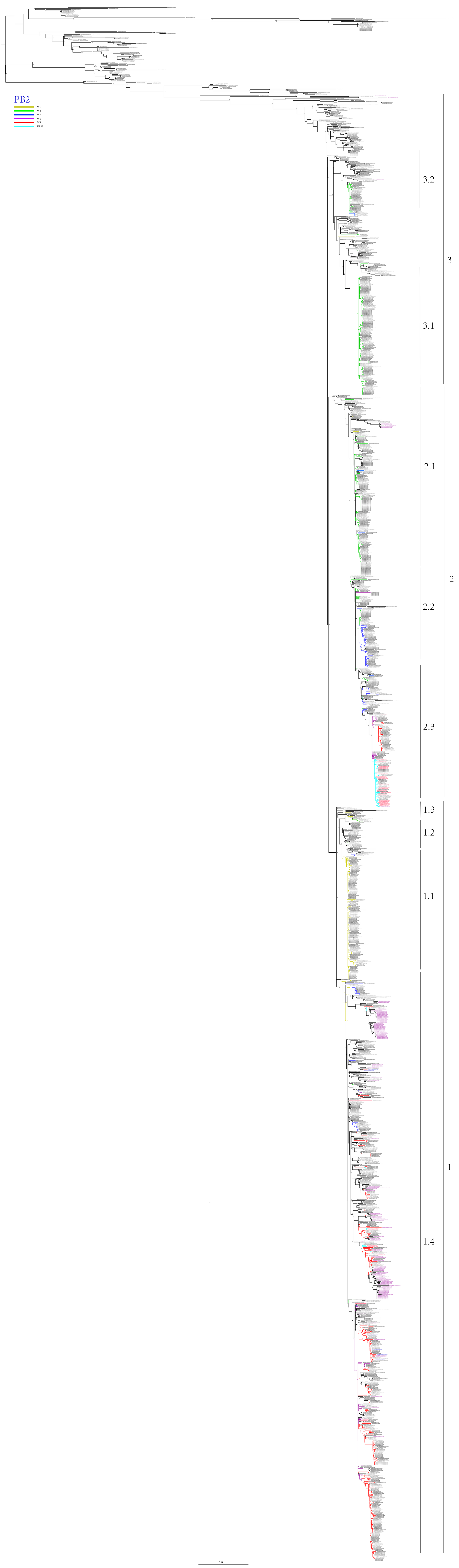

PB2
W1
W2
W3
W4
W5
HPAI
3.2
3.1
3
2.1
2.2
2.3
2
1.3
1.2
1.1
1.4
1

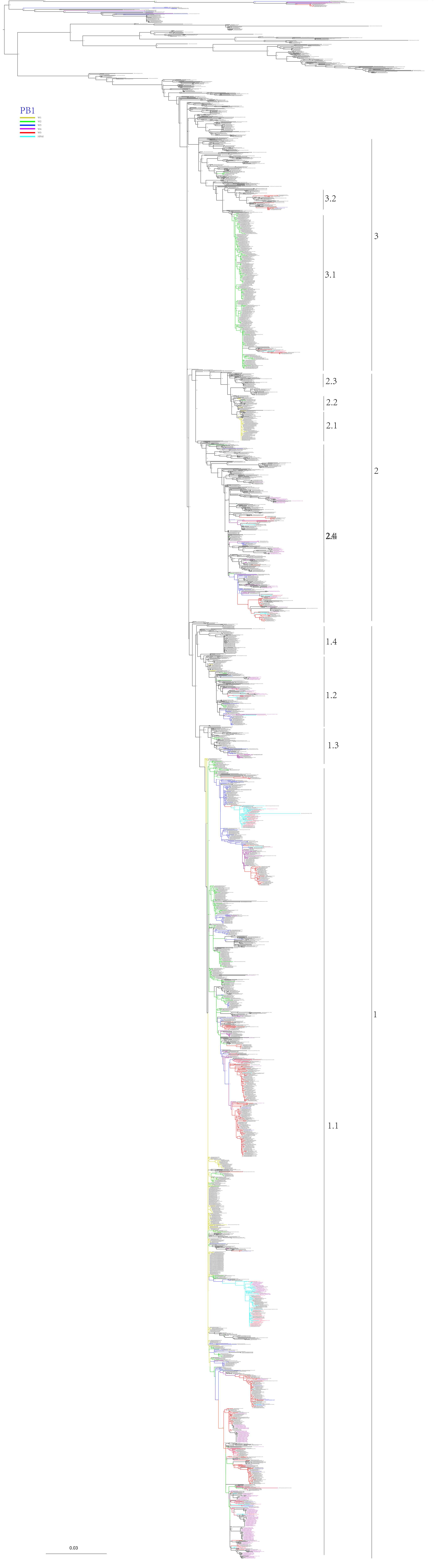
PB1
W1
W2
W3
W4
W5
HPAI
3.2
3.1
3
2.3
2.2
2.1
2.4
2
1.4
1.2
1.3
1.1
1
0.03

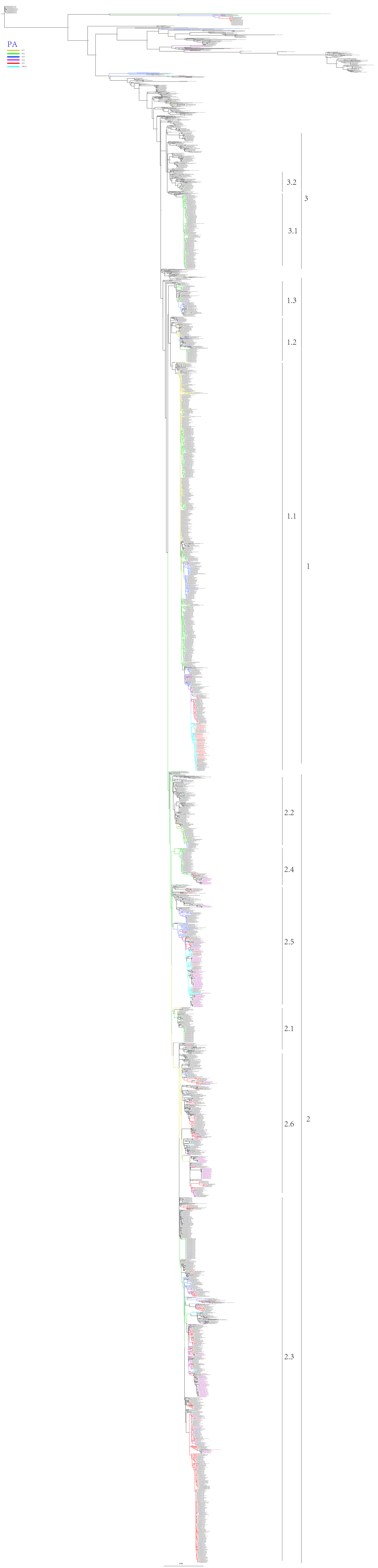
PA
3.2
3.1
3
1.3
1.2
1.1
1
2.2
2.4
2.5
2.1
2.6
2
2.3

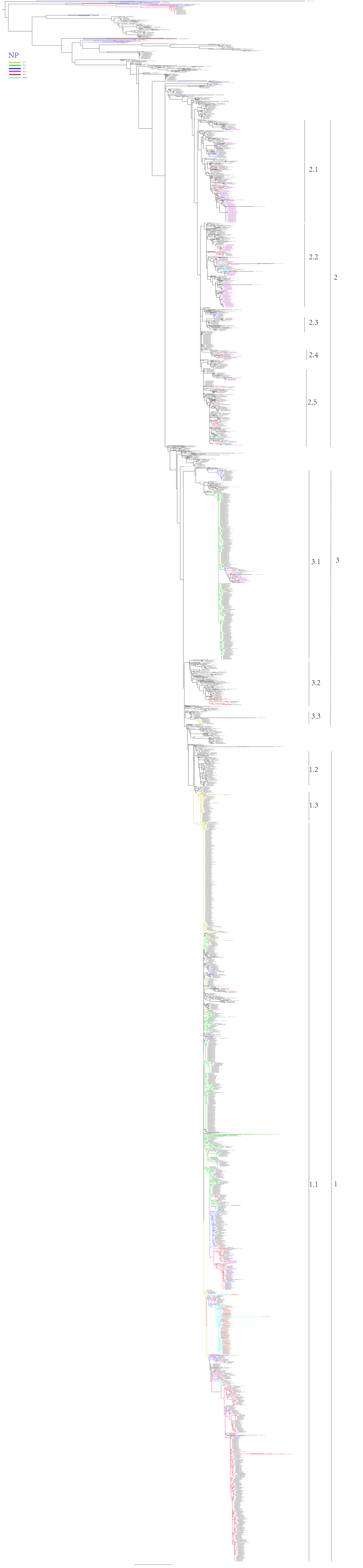
NP
2.1
2.2
2
2.3
2.4
2.5
3.1
3
3.2
3.3
1.2
1.3
1.1
1

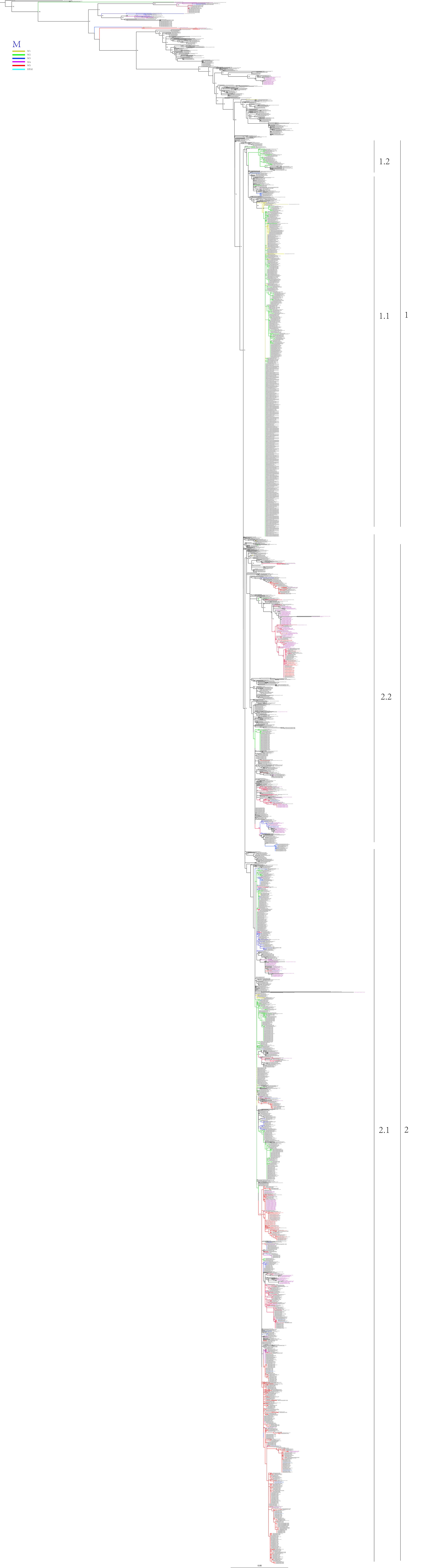
M
W1
W2
W3
W4
W5
HPAI
1.2
1.1
1
2.2
2.1
2
0.05

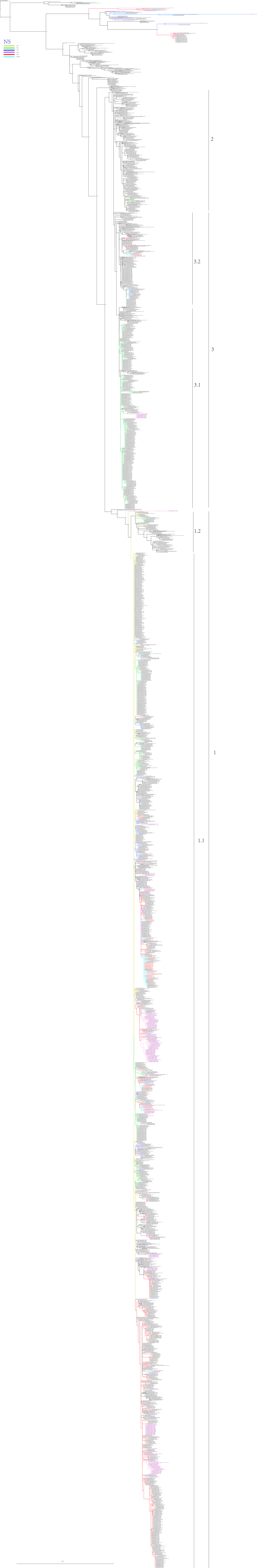
NS
2
3.2
3
3.1
1.2
1
1.1
